# Emergent Ferroelectric Nematic and Heliconical Ferroelectric Nematic States in an Achiral “Straight” Polar Rod Mesogen

**DOI:** 10.1002/advs.202405718

**Published:** 2024-08-05

**Authors:** Hiroya Nishikawa, Daichi Okada, Dennis Kwaria, Atsuko Nihonyanagi, Motonobu Kuwayama, Manabu Hoshino, Fumito Araoka

**Affiliations:** ^1^ RIKEN Center for Emergent Matter Science (CEMS) 2‐1 Hirosawa, Wako Saitama 351‐0198 Japan; ^2^ Faculty of Electrical Engineering and Electronics Kyoto Institute of Technology Matsugasaki, Sakyo‐ku Kyoto 606‐8585 Japan; ^3^ Present address: Graduate School of Medicine, and General Medical Education and Research Center Teikyo University 2‐11‐1, Kaga, Itabashi‐ku Tokyo 173‐8605 Japan

**Keywords:** ferroelectric nematic, ferroelectric smectic C, heliconical ferroelectric nematic, polarization, SHG

## Abstract

Ferroelectric nematic liquid crystals (N_F_LCs) are distinguished by their remarkable polarization characteristics and diverse physical phenomena, sparking significant interest and excitement within the scientific community. To date, over 150 N_F_LC molecules are developed; however, there are no reports regarding straight linear polar molecules with a parallel alignment of the permanent dipole moment and the molecular axis. The straight polar mesogen **nBOE** exhibits an enantiotropic N_F_ phase with a wide temperature window (up to 100 K) despite having a longer alkyl chain (up to *n* = 6) than the critical alkyl chain length of conventional models. Interestingly, **nBOE** with a medium‐length alkyl chain displays an exotic phase sequence of N_F_–*^HC^* N_F_–SmX_F_ during the elimination of positional displacement among adjacent molecules. Furthermore, the reflective color modulation of the *^HC^* N_F_LC over the entire VIS‐NIR spectral regime by ultralow *E*‐field (up to 0.14 V µm^−1^) is demonstrated.

## Graphical abstract

A new class of helielectric phase (*^HC^* N_F_) is discovered, which appears in an achiral straight polar rod molecule. These careful studies suggest an effect of positional displacements between adjacent molecules on the emergence of an exotic phase sequence. Notably, the *^HC^* N_F_ phase exhibits effective tuning of the reflective color over the entire visible spectral range by an ultralow electric field.

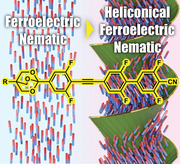

## 1 Introduction

**Figure 1:**
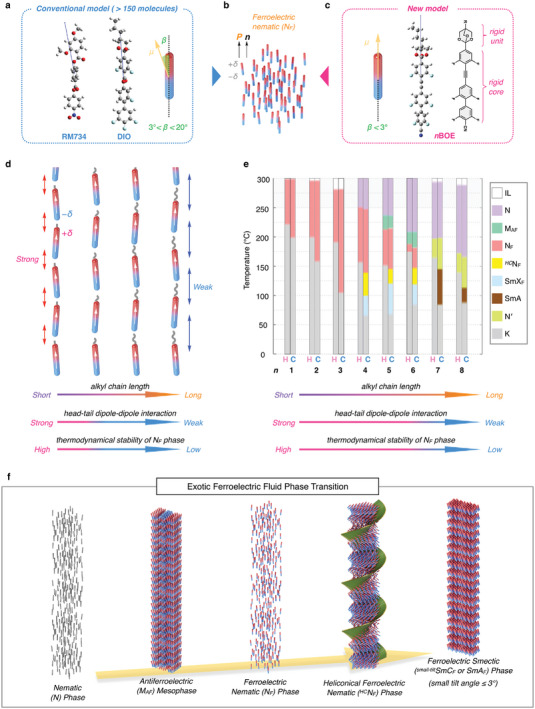
A schematic illustration of a ferroelectric nematic (N_F_) phase (b) induced by conventional (RM734, DIO) (a) and straight rod mesogen (**BOE**) (d). *β* denotes the angle between dipole moment and a direction of long axis for the molecule. c) A plausible model to induce the N_F_ phase. e) Phase transition behavior of **nBOE** (*n* = 1–8). Gray‐colored area represents decomposition temperature that investigated by thermogravimetric analysis. Abbrev.: IL = isotropic liquid, N = nematic, M_AF_ = antiferroelectric mesophase, N_F_ = ferroelectric nematic, *^HC^* N_F_ = heliconical ferroelectric nematic, SmX_F_ = small tilt ferroelectric smectic C or ferroelectric smectic A, SmA = smectic A, N′ = cybotactic nematic, K = crystal. f) A schematic illustration of an exotic ferroelectric phase transition of **BOE** system. The unique interactions in **BOE** lead to not only N_F_ phase but also heliconical ferroelectric nematic (*^HC^* N_F_) and ferroelectric smectic (SmX_F_) phases.

Giant fluid ferroelectricity emerges in a new class of matter states called the ferroelectric nematic (N_F_) phase,^[^ ^1^, ^2^, ^3^ ^]^ which is described by a long‐range polar orientational order. Thus, the N_F_ phase has a global C_∞_ *_v_* symmetry since the macroscopic polarization aligns along the director (**Figure** **1**b). Usually, the N_F_ phase can be formed by a liquid crystalline (LC) rod‐like molecules with high dipole moment (>9 Debye),^[^ ^4^ ^]^ exhibiting giant polarization behavior, that is, as apparent dielectric permittivity (<≈10k),^[^ ^5^ ^]^ polarization density (>≈4 µC cm^−2^),^[^ ^5^ ^]^ and NLO coefficient (<≈10 pm V^−1^)^[^ ^5^ ^]^ and unique physical properties such as topology,^[^ ^6^, ^7^ ^]^ instability,^[^ ^8^, ^9^ ^]^ fiber,^[^ ^10^, ^11^ ^]^ thermomotor,^[^ ^12^ ^]^ superscreening^[^ ^13^ ^]^ as well as N_F_–isotropic liquid critical end point.^[^ ^14^ ^]^ These outstanding characteristics have led to exponential growth in state‐of‐the‐art ferroelectric research. In particular, research dedicated to a deeper understanding of the relationship between the molecular structure and N_F_ phases is flourishing, with over 150 types of N_F_LC molecules developed to date.^[^ ^4^ ^]^ The structure of N_F_LC molecules is highly delicate and difficult to tailor without inspiring the backbone of the archetypal N_F_LC molecules (i.e., DIO,^[^ ^1^ ^]^ RM734,^[^ ^2^ ^]^ and UUQU‐4‐N^[^ ^15^ ^]^).

However, some studies have provided valuable insights into molecular design. Mandle et al.^[^ ^16^ ^]^ and Chen et al.^[^ ^3^ ^]^ independently reported that a strong dipole–dipole interaction along the director triggers the emergence of the N_F_ phase with the aid of molecular dynamics simulations. Madhusudana introduced a model describing a molecule (a rod entity) in the N_F_ phase by a longitudinal surface charge density wave.^[^ ^17^ ^]^ In this model, the adjacent molecules orient in a syn‐polar fashion with a molecular offset such that the amplitude of the charge density waves is minimized (thus reducing the electrostatic energy), further indicating that this polar arrangement emerges in a high‐density state. Notably, the model with an alternating charge distribution along the molecular axis was consistent with N_F_LC molecules. Cruickshank et al. obtained good feedback on the validity of Madhusudana's model within RM734 families.^[^ ^18^, ^19^, ^20^ ^]^ Nacke et al. discussed Mandle's and Madhusudana's model in the N_F_ phase for AUUQU‐2‐N using synchrotron‐based X‐ray diffraction (XRD) studies.^[^ ^21^ ^]^ A smectic C‐type long‐range correlation is suggested over the entire temperature range of the N_F_ phase for AUUQU‐2‐N. In their proposed model, the molecules packed in a layer are slightly displaced from each other, forming a polar structure (a similar model reported in our previous study^[^ ^22^ ^]^). More recently, Marchenko et al. directly visualized synpolar molecular ordering with a molecular offset in a monolayer on an Au(111) surface.^[^ ^23^ ^]^

Much effort has been made to develop N_F_LCs, but the dipole moment of N_F_LC generics (>150 types) forms an angle between 10° and 25° with the long axis (Figure 1a). This property is attributable to the molecular structure consisting of a polar linker (e.g., COO, CF_2_O) and a polar end unit (e.g., 1,3‐dioxane, ester^[^ ^24^ ^]^), which both have dipole moments that deviate from the molecular axis. Furthermore, the common characteristics of the N_F_ phase in conventional models include being thermodynamically metastable and significantly destabilized (or vanishing) as the length of the alkyl chain increases owing to the reduction of the head‐tail dipole‐dipole interaction (Figure 1d). It is of paramount importance to develop a molecular design that can overcome the universally observed drawbacks.

In this paper, we present a new model, **nBOE** (*n* = 1–8), with a hard‐rod polar molecule motif, in which the direction of the dipole moment and the molecular axis are in an approximately perfect parallel alignment (Figure 1c). Notably, we found that the **nBOE** variants exhibited not only an enantiotropic N_F_ phase with a wide temperature window but also an exotic ferroelectric fluid phase transition via N_F_, heliconical N_F_ (*^HC^* N_F_), and ferroelectric smectic (SmX_F_) phases (Figure 1e,f). Note that this ferroelectric smectic phase could be either small tilt ferroelectric smectic C or ferroelectric smectic A, but is defined here as SmX_F_. Recently, new polar helical phases (N_TBF_^[^ ^25^ ^]^ and ${\mathrm{SmC}}_{p}^{H}$ ^[^ ^26^ ^]^) have been found in similar achiral molecules. However, we have opened the way for exploring novel helielectric phases in ferroelectric fluid libraries of rigid rod mesogen, along with new helielectric phases in even just “straight” polar rods.

## 2 Results and Discussion

### 2.1 Molecular Structure of BOE Series

**Figure 2:**
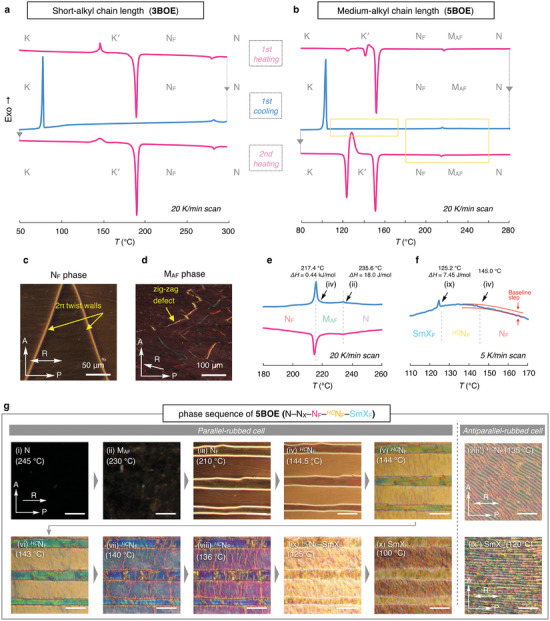
DSC studies. DSC curves of **3BOE** (a) and **5BOE** (b,e,f,). Scan rate: 20 K min^−1^ (a,b,e), 5 K min^−1^ (f). e,f) Enlarged DSC curves for **5BOE** in regions indicated by yellow boxes in the panel (b). c,d,g) POM images of various LC phases taken under cross polarizers in the parallel‐ and antiparallel rubbed cells. Areas designated by Roman numerals (i–x, viii′ and ix′) corresponds to the observations in DSC and POM studies. g) scale bar: 50 µm.

The chemical structures of the **BOE** series are shown in Figure 1c. Compared to the common motifs (RM734, DIO, and UUQU‐4‐N), the structure of **BOE** does not bear a flexible linker such as ester (COO) or difluoromethoxy (CF_2_O) units; instead, it incorporates a rigid diphenyl alkyne unit, well‐known as tolan. Furthermore, to increase the whole dipole moment (*µ*) and to prevent deviation of the dipole from the molecular axis, we end‐capped the tolan unit using a bicycloorthoester unit, designing the **BOE** molecules. In such a straight, hard rod molecule, the dipole moment may direct along the long axis of the molecule. As expected, the **nBOE** series (*n* = 1–8) showed a small *β* angle ranging between 0.25° and 4.7° owing to the hard‐rod molecular design. The optimized structures of **nBOE** obtained using DFT calculations are displayed in Figure S14a (Supporting Information). The calculated *µ* and *β* as a function of the number of carbon atoms (n) for **nBOE** are shown in Figure S14b,c and Table S1 (Supporting Information). **1BOE–3BOE** (short‐alkyl chain group) showed a negligibly small *β* (<0.3°), indicating the dipole moment and the molecular axis are perfectly parallelly aligned. With increasing alkyl chain length, *β* also increased to between 1° and 3° for **4BOE**–**6BOE** (medium‐alkyl chain group). **7BOE** and **8BOE** (long‐alkyl chain group) still showed small angles of *β* = 3.4° and 4.7° respectively. Lengthening the alkyl chain slightly increased the *µ* value, marking ca. 15 Debye for all the **BOE** series. Figures 1e and **2**a,b,e,f show the phase transition behaviors and DSC curves of **nBOE**, respectively. For short‐alkyl chain groups (*n* = 1–3), the N_F_ phase appeared at the melting point of the pristine crystal, and the N_F_ phase emerged again from the upper phase [isotropic liquid (IL) or nematic (N)], indicating enantiotropic N_F_LC behavior in these compounds. The N_F_ phase is usually characterized by 2*π*‐twist walls (Figure 2c).^[^ ^27^ ^]^ Medium‐alkyl chain groups (*n* = 4–6) were also found to be enantiotropic N_F_LCs. In addition, in the case of *n* = 5,6, the antiferroelectric mesophase (M_AF_) was observed. This phase was characterized by polarized optical microscope (POM) images with the typical zig‐zag texture (Figure 2d) and antiferroelectric polarization switching. The M_AF_ phase has a periodic structure of the antiferroelectric domain consisting of small polar regions (Figure 1f). The plausible structures and the corresponding nomenclature of this phase (i.e., N_s_ and SmZ_A_) are proposed by Mertelj et al.^[^ ^28^, ^29^ ^]^ and Chen et al.^[^ ^30^ ^]^ On the other hand, the N_F_ phase was excluded for longer alkyl chain groups (*n* = 7–8); instead, apolar phases (N′ and SmA) appeared. With increasing molecular length, the N phases appeared as a highest temperature mesophase. The N and N′ phases have similar characteristics observed by POM (for bare glass and non‐rubbed PI cells, Figure S8, Supporting Information), DR and XRD, but are distinguished with a weak first‐order N–N′ transition, suggesting that the N′ phase may be analogous to the N phase. The blocky‐like texture in a planar (annealed PMMA) anchoring and the presence of SmA in the phase sequence suggest the possibility of a cybotactic nematic phase with a local SmA ordering (see also Note S3, Supporting Information). Notably, **nBOE** (*n* = 1–5) exhibited a broadened temperature window for the N_F_ phase (≈50–100 K) during heating. Interestingly, **nBOE** induces the N_F_ phase even with long alkyl chains, which significantly differs from conventional N_F_LCs. For instance, the N_F_ phases exhibited in RM734 and DIO (hereafter referred to as **nRM** and **nDIO**, respectively) are thermodynamically metastable (see Figure S15, Supporting Information). Irrespective of the alkyl chain length, **nRM** exhibited a monotropic N_F_ phase, which barely emerged for *n* = 2 (**2RM**). By contrast, **nDIO** with short alkyl chains (*n* = 1–2) is an enantiotropic N_F_ phase, but the temperature windows are quite narrow (<7 K) upon heating. The metastable N_F_ phase was observed narrowly within **4DIO**. In sharp contrast, the N_F_ phase remained alive in **nBOE** up to *n* = 6. Unexpectedly, two extra ferroelectric phases, heliconical ferroelectric nematic (*^HC^* N_F_) and ferroelectric smectic (SmX_F_), emerged below the N_F_ regime for **nBOE** (*n* = 4–6) (Figure 1e).

### 2.2 Phase Transition Behavior and Unique Polarized Optical Microscope Textures

The common DSC features of **nBOE** (*n* = 4–6) within the N_F_–*^HC^* N_F_–SmX_F_ regime were a baseline step (N_F_–*^HC^* N_F_) and a distinct exothermal peak (*^HC^* N_F_–SmX_F_), as shown in Figure 2f and Figures S16–S18 (Supporting Information). Figure 2g displays the unique texture change through the unique cascade phase changes of **5BOE**. During cooling from the N_F_ phase, the reddish texture gradually appeared at 145 °C (panel (iv) in Figure 2f,g) in a parallel‐rubbed cell. Further cooling led to a gradual color change (blue shift) in the POM texture. However, in this state, vivid blue reflection can be observed when viewed by naked eyes, indicating that a specific helical structure exists in the *^HC^* N_F_ phase (complete images are shown in Figure S19a, Supporting Information). Notably, this texture of the *^HC^* N_F_ phase differed from the planar texture with defects (a.k.a., oily streaks) of the helicoidal ferroelectric nematic, *^HD^* N_F_ (o.k.a., chiral ferroelectric nematic, N_F_*) phase in the rubbed cell (Figure S20, Supporting Information).^[^ ^31^, ^32^ ^]^ At ≈125 °C, a drastic change in texture with strong light scattering occurred (panel (ix) in Figure 2f,g). The textural difference between *^HC^* N_F_ and SmX_F_ phases was more remarkable in an antiparallel‐rubbed cell (panels (viii′) and (ix′) in Figure 2g; Figure S19b, Supporting Information). In the *^HC^* N_F_ phase, a striped texture tilted at a certain angle relative to the rubbing direction appeared. Similarly, a striped texture emerged in the SmX_F_ phase; however, unlike in the *^HC^* N_F_ phase, the orientation of the stripes was parallel to the rubbing direction. For the *^HC^* N_F_ phase, similar behavior is usually observed in twist‐bend nematic (N_TB_) materials, including a second‐order‐like N–N_TB_ transition and a striped texture.^[^ ^33^ ^]^ The details of the *^HC^* N_F_ and SmX_F_ phases are discussed in Section 2.5 and Note S2 (Supporting Information), respectively.

### 2.3 Ferroelectric Behavior of the N_F_, *^HC^* N_F_, and SmX_F_ Phases

**Figure 3:**
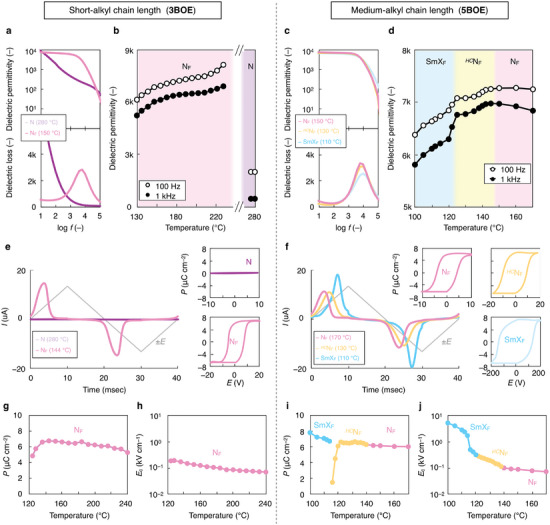
Polarization behavior. Dielectric spectra (**3BOE**: a, **5BOE**: c) and dielectric permittivity versus temperature (**3BOE**: b, **5BOE**: d). Polarization reversal current profile (**3BOE**: e, **5BOE**: f). Insets represent the corresponding *P*–*E* hysteresis curves. Polarization density versus temperature (**3BOE**: g, **5BOE**: i). Coercive electric field (*E*_c_) versus temperature (**3BOE**: h, **5BOE**: j).

To evaluate the ferroelectricity of the three polar phases (N_F_, *^HC^* N_F_, and SmX_F_) for **nBOE**, we performed dielectric relaxation (DR), polarization reversal current (or *P*–*E* hysteresis), and second harmonic generation (SHG) measurements. **Figure** **3**a–d shows the results of the DR studies for **3BOE** and **5BOE**. For both cases, the giant dielectric permittivity (*ε*′) of ≈6k–8k (**3BOE**) and ≈6.8k (**5BOE**) was observed in the N_F_ regime, corresponding to its magnitude for typical N_F_LCs. For **5BOE**, with decreasing temperature, the *ε*′ value in the N_F_ phase experienced a minor increase, followed by a gradual decrease (down to ≈6.5k) upon entering the *^HC^* N_F_ regime. The *ε*′ value dropped to ≈6.0k at the *^HC^* N_F_–SmX_F_ phase transition temperature, continuing to reduce *ε*′ toward the low‐temperature side (Figure 3d). The relaxation frequency was slightly increased via the N_F_→*^HC^* N_F_→SmX_F_ phase transition (Figure 3c; Figure S21, Supporting Information).

For the polarization reversal current measurement, no current peak was observed in the N phase for **3BOE**, whereas a distinct peak appeared in the N_F_ phase for **3BOE** and **5BOE** (Figure 3e,f). As shown in the corresponding *P–E* hysteresis (insets in Figure 3e,f), a parallelogram hysteresis loop, commonly observed in ferroelectrics, was obtained for the N_F_, *^HC^* N_F_, and SmX_F_ phases. The temperature dependence of *P* is shown in Figure 3g (**3BOE**) and Figure 3i (**5BOE**). The average spontaneous polarization (*P*_ave_) in the N_F_ regime was nearly the same ≈6.1 µC cm^−2^ for **3BOE** and **5BOE**. For **5BOE**, in the *^HC^* N_F_ and SmX_F_ regime, large *P*_ave_ values of ∼6.5 and ≈6.8 µC cm^−2^ were observed, respectively. As a first approximation, the polar ordering, <P1>, in the N_F_ phase for **nBOE** (*n* = 1–6) was estimated using the equation:^[^ ^34^ ^]^

(1) $$<P1>\;\approx P_{\mathrm{av}}/P_{\max}$$

(2) $$P_{\max}=\mu \left(\rho /M\right)N_{A}$$

where <P1>, *P*_max_, *µ*, *M*, and *N*_A_ are the polar order parameter, maximum electron polarization, permanent dipole moment, molar mass, and Avogadro constant, respectively. The parameter *P*_max_ implies that all the molecular dipoles are aligned perfectly in one direction along **n**). The obtained <P1> values are listed in Table S2 (Supporting Information). Although the BOE series with a relatively long alkyl chain showed a relatively high <P1> ≈0.8, **1BOE** showed the lowest <P1> ≈0.6. This result suggests a relatively long alkyl chain offers a suitable molecular alignment for favorable dipole–dipole interactions. Notably, the three phases exhibited extremely small coercive *E*‐field (*E*_c_) of 0.01, 0.1, and 1 kV cm^−1^ order in the N_F_, *^HC^* N_F_, and SmX_F_ phases, respectively (Figure 3h,j). Moreover, the *^HC^* N_F_ phase inherited its unique property, with its *E*_c_ being one order of magnitude lower than that of a similarly reported helielectric (N_TBF_) phase.^[^ ^25^ ^]^ Thus, these results indicate that the tolan‐based polar molecule can dramatically reduce the *E*_c_ of various ferroelectric fluids.

Strong SHG activities in the N_F_, *^HC^* N_F_, and SmX_F_ phases were observed in **3BOE** and **5BOE** (further details provided in Note S1, Supporting Information). Therefore, the DR, *P*–*E* hysteresis, and SHG studies demonstrate that the N_F_, *^HC^* N_F_, and SmX_F_ phases exhibit remarkable ferroelectric behavior. Similar behavior for DR, *P*–*E*, and SHG properties were observed for **4BOE** and **6BOE** (Figures S22, S23, and S5, Supporting Information). Figure S24 (Supporting Information) shows the complete data on the polarization reversal current. By combining these studies, three mesophases (N, N′, and SmA) exhibited in **7BOE** and **8BOE** (long‐alkyl chain groups) were found to be paraelectric LC phases.

### 2.4 Structure Characterization Based on X‐Ray Diffraction Analysis

**Figure 4:**
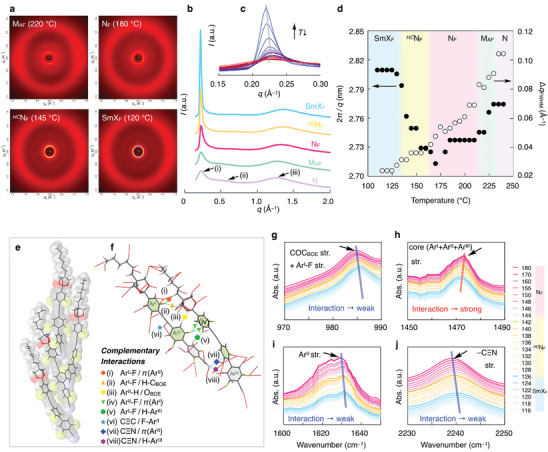
XRD and FTIR spectra studies. 2D XRD (a) and 1D XRD (b,c) pattern in various phases for **6BOE** (non‐aligned samples). d) 2*π* *q* ^−1^ and FWHM versus Temperature for **6BOE**. Structures of local cluster (e) and adjacent dimer (f) extracted from SC‐XRD data for **6BOE**. g–j) FTIR absorbance spectra for **5BOE**. For the panel (b), the recorded temperature in N, N_F_, *^HC^* N_F_, and SmX_F_ phases are 240, 220, 180, 145, and 120 °C, respectively.

XRD measurements were performed to characterize the LC structures, particularly the polar ordering for **nBOE** (*n* = 1–6). Figure S25 (Supporting Information) shows the 1D XRD patterns of **2BOE** and **3BOE**. For both compounds, within the N_F_ range, the position of relatively sharp diffraction peak at small angles may correspond to the molecular length. We characterized the LC phases of **6BOE** based on XRD analysis. **Figure** **4**a–c shows 2D and 1D X‐ray diffractograms obtained for non‐aligned samples in various phases, respectively. For all LC phases, three distinct peaks were observed from small to wide angle, for instance (e.g., *q* = 2.77, 1.26, 0.48 Å^−1^ in the N phase). The 1D XRD profiles of N, M_AF_, N_F_, *^HC^* N_F_ and SmX_F_ phases are shown in Figure 4b. With decreasing temperature, the position of peak (iii) shifted to the large *q* side owing to face‐to‐face molecular stacking, suggesting that the stacking distance changed. By contrast, the relative intensity and full width at half maximum (FWHM) of phases significantly increased and decreased, respectively, upon cooling. In particular, within the SmX_F_ range, this trend was more remarkable. Thus, this indicates that increase of correlation length and formation of a long‐range positional order within a smectic layer. Notably, the value of 2*π* *q* ^−1^ was decreased from the N to N_F_ phases, whereas in the *^HC^* N_F_ regime, the value of 2*π* *q* ^−1^ was increased, and was saturated in the SmX_F_ phases (Figure 4c,d). Note that in the previous report regarding the polar SmC phases,^[^ ^25^, ^26^ ^]^ the *d*‐spacing (2*π*/*q*) decreases with decrease temperature, indicating the presence of molecular tilt in a layer. In the contrary, in the SmX_F_ phase for **nBOE** (*n* = 4–6), the *d* value was constant, yet this is usually characterized as SmA_F_ phase. However, two distinct domains with opposite polarization properties were observed in the thinner rubbed cell (Note S2, Supporting Information). Thus, we propose that the smectic phase for **BOE** is characterized the small tilted ferroelectric smectic C phase (SmC_F_) but resemble as SmA_F_ phase. Similar XRD data set was obtained for **4BOE** and **5BOE** (Figures S26–S28, Supporting Information). The *d*‐spacing in the SmX_F_ phase for **4BOE** and **5BOE** was nearly according to the molecular length (*L*_m_), whereas **6BOE** exhibited a slightly smaller *d* value (*d* < *L*_m_), which may be due to the folded alkyl chain. Assuming that *d* = *L*_m_ in the SmX_F_ phase, dimerization with molecular displacement may occur in the *^HC^* N_F_ and N_F_ phases. Indeed, the single crystal XRD (SC‐XRD) results for **6BOE** indicate the presence of a cluster with a molecular offset (Figure 4e; Figure S29, Supporting Information). In the cluster, the syn‐polar arrangement with a molecular offset seems to be generated via multiple complementary interactions such as hydrogen bonding, fluorine/*π*, and CN/*π* (Figure 4f).

Figure 4g–j shows the changes in the Fourier transform infrared (FTIR) absorbance spectra related to specific stretching vibrations (see also Note S4, Supporting Information). With decreasing temperature, the peak position due to the stretching vibrations of C─O─C_BOE_, Ar^I^─F, Ar^II^, and C≡N continuously shifted to high wavenumber. By contrast, the vibrational peak of the core mesogen (A^I^+A^II^+A^III^) shifted to a lower wavenumber. By combining the XRD and FTIR data, we propose the following model. i) head‐and‐tail and complementary interactions such as hydrogen bonding, fluorine/*π*, and CN/*π* produce the syn‐polar fashion with a molecular offset, introducing the N_F_ phase, ii) the molecular offset level becomes more significant by the complementary interactions, leading to the emergence of the *^HC^* N_F_ phase, iii) noticeable contribution of the complementary interactions leads to a predominance of face‐to‐face communication with eliminating the molecular offset level, thereby transitioning to the SmX_F_ phase. For (i), we believe that the complementary interactions also effectively stabilize the N_F_ phase, suggesting that the N_F_ phase remains alive even in **6BOE** with a long alkyl chain. For (ii), the **nBOE** (*n* = 4,5) replaced the ─CN group with either ─F or ─NO_2_ groups (i.e., **nBOE‐F** and **nBOE‐NO_2_**) did not induce the *^HC^* N_F_ phase in either case (Note S5, Supporting Information). This result suggests that specific interactions via the ─CN group are crucial for inducing the *^HC^* N_F_ phase.

### 2.5 Unique Characteristics of the *^HC^* N_F_ Phase

**Figure 5:**
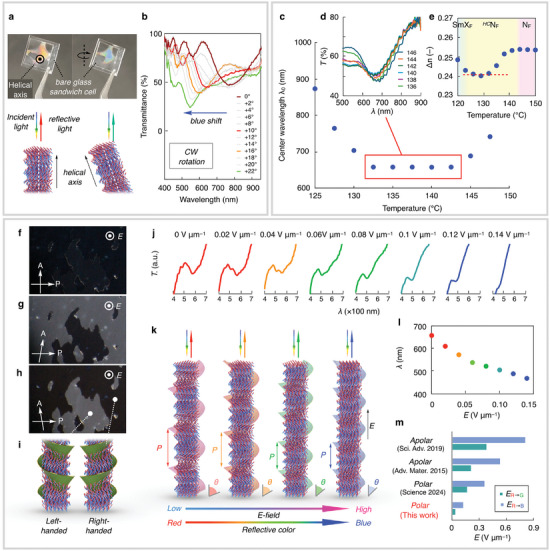
Unique properties of *^HC^* N_F_ phase emerging in **5BOE**. a) Real photos of *^HC^* N_F_ phase (≈145 °C) By rotating the cell, the reflection color changed from orange to blue. b) Rotation angle dependence of reflection spectra in the *^HC^* N_F_ phase (145 °C). c) A schematic illustration of helicoidal structure of *^HC^* N_F_ and estimated color of reflective light. d) Center wavelength (*λ*) of reflection band versus Temperature. e) Birefringence versus temperature, POM images during an applied *E*‐field (0.25 V µm^−1^) under crossed (f) and decrossed polarizers (g,h). i) left‐/right‐handed heliconical ferroelectric nematic structure. *E*‐field driven color modulation of a *^HC^* N_F_LC film (10 µm). j) and the corresponding peak shift (l). k) Schematic illustration of ferroelectric heliconical structure and their structure change under *E*‐field. m) Comparison of required *E*‐field to change in reflective color from red to green or to blue. In the panel (m), examples of conventional apolar and latest polar heliconical system are shown.

As shown in Section 2.2, the *^HC^* N_F_ phase showed a reflective color, suggesting the presence of a helical structure, but it may be distinct from the typical *^HD^* N_F_ phase owing to its unusual POM texture. One possibility is that the molecules were oblique to the helical axis in the *^HC^* N_F_ phase, resulting in an oblique helicoidal or heliconical structure. When **nBOE** (*n* = 4–6) was injected into the bare glass sandwich cell, the reflective color was visible to the naked eye. In the case of **5BOE**, the alignment was more uniform than those of the others; therefore, unless otherwise noted, the investigation regarding the helical structure of the *^HC^* N_F_ phase was performed using **5BOE**. A relatively uniform texture is observed in the POM image of the bare glass cell; however, no oily streak texture is observed (Figure S20, Supporting Information). When the cell was observed straight on, an orange reflective color was observed, whereas a blue shift in color became apparent from an oblique angle (**Figure** **5**a). Figure 5b shows the reflection spectra as a function of the cell rotation (oblique) angle. When the cell rotated counterclockwise, a blue shift from the red reflective color was observed. Similarly, the clockwise rotation showed a blue shift in the structural color (Figure S30a, Supporting Information). Therefore, despite the rotation direction, the constant peak displacement indicated that the helical axis of the *^HC^* N_F_ phase stands normal to the cell plane (Figure 5a). Figure 5c,d, and Figure S28b (Supporting Information) show the temperature‐dependent spectral changes in the bare glass cell. Interestingly, unique spectral changes occurred sequentially in the *^HC^* N_F_ regime: i) a blue shift to ≈680 nm, ii) no peak shift at 680 nm (Figure 5d), iii) a redshift over 900 nm (outside the measurement range). The almost constant birefringence within the *^HC^* N_F_ regime (Figure 5e; Figure S31, Supporting Information) coincides with the result for (ii).

Next, we investigated the electric field (*E*‐field) response of the helical structure in the *^HC^* N_F_ phase. In the planar state of the cholesteric LCs, the strong *E*‐field (several V µm^−1^) along the helical axis allows homeotropic alignment of LC because the elastic free energy is dominated by the electric free energy collapsing the helical structure. Similarly, a vertical direct current *E*‐field was applied to the *^HC^* N_F_ phase in the ITO‐coated glass cell. With a small *E*‐field of 0.25 V µm^−1^, the birefringence nearly disappeared (Figure 5f), suggesting the homeotropically aligned polar heliconical structure along the *E*‐field. Notably, by slightly decrossing the polarizers to the scarcely observed domain boundaries, the black/white contrast between adjacent domains was reversed (Figure 5g,h). This result indicates that left‐/right‐handed helical structures coexist and chiral symmetry breaking occurred spontaneously (Figure 5i). As the intensity of the *E*‐field progressively increased, the spectral width narrowed, accompanied by a simultaneous shift of its position toward the shorter‐wavelength side, confirming the selective reflective colors over a wide range of wavelengths (Figure 5j,l). This color‐change mechanism in the *^HC^* N_F_ phase is probably identical to that observed in heliconical nematics.^[^ ^35^ ^]^ Thus, as shown in the model (Figure 5k), in the heliconical nematic, the director is tilted with some angle *θ* < *π*/2 with respect to the helical axis. By applying the *E*‐field along the axis, the director is reoriented with decreasing *θ*, changing the helical pitch (*P*) without reorienting the helical axis. Typically, a heliconical nematic can be created by blending a chiral dopant to induce chirality in the host LCs (twist‐bend nematic and typical nematic). However, it is noteworthy that the *^HC^* N_F_ phase is the helielectric version of the heliconical nematic phase so that its characteristics differ dramatically from those of the heliconical nematics: i) the *^HC^* N_F_ phase is generated spontaneously because of the coupling between polar and chiral symmetry breaking, and ii) the ultralow *E*‐field‐driven multicolor modulation is due to the coupling of polarization and voltage. The exceptional *E*‐field response to the helical pitch modulation is remarkably smaller (up to 0.14 V µm^−1^) than that of the reported system (Figure 5m).^[^ ^25^, ^35^, ^36^ ^]^

## 3 Conclusion

In conclusion, we developed just “straight” polar rod mesogens, **nBOE** (*n* = 1–8), in which the dipole moment aligned nearly perfectly parallel to the molecular axis. Unlike the characteristics of the N_F_ phase, which emerges in a library of over 150 types of molecules, the enantiotropic N_F_ phase was observed even in **nBOE** molecules with long alkyl chains (up to *n* = 6). For **nBOE** (*n* = 4–6) with medium‐length alkyl chains, we discovered emergent *^HC^* N_F_ and SmX_F_ phases with heliconical structures and small molecular tile angles, respectively. The DR, *P*–*E*, and SHG studies evidenced the ferroelectricity of the N_F_, *^HC^* N_F_, and SmX_F_ phases owing to its giant dielectric permittivity (6k–8k), large spontaneous polarization (4.6–6.5 µC cm^−2^), and high SHG activation. The findings from XRD and spectra analysis elucidated that the exotic phase sequence (N_F_→*^HC^* N_F_→SmX_F_) proceeds via a mechanism that eliminates the offset level between adjacent molecules due to alterations in the strength of complementary interactions. Additionally, we demonstrated ultralow *E*‐field‐driven color tunability across the entire VIS‐NIR spectral range of the *^HC^* N_F_ phase. We believe that the straight polar rod model can be utilized as a novel strategy for the emergence of spontaneous polar and chiral symmetry breaking, unlocking novel helielectric phases in polar fluid materials.

## Conflict of Interest

The authors declare no conflict of interest.

## Author Contributions

H.N. conceived the project and designed the experiments. F.A. co‐designed the work and constructed the optical and electrical setups for SHG. H.N. performed all the experiments. D.O. constructed the optical setups and performed the optical experiments. F.A. supported XRD measurements. D.K. partially performed *P*–*E* hysteresis/DR studies. H.N. and D.K. partially synthesized compounds. A.N. synthesized all compounds. M.K. co‐designed a synthetic strategy and measured HRMS. M.H. measured and analyzed single crystal XRD. H.N. and F.A. analyzed data and discussed the results. H.N. and F.A. wrote the manuscript, and all authors approved the final manuscript.

## Supporting information

Supporting Information

Supporting cif

## Data Availability

The data that support the findings of this study are available in the supplementary material of this article.
