# Supplementary material for: Emergent Ferroelectric Nematic and Heliconical Ferroelectric Nematic States in an Achiral “Straight” Polar Rod Mesogen
Source: Adv Sci (Weinh). 2024 Aug 5;11(39):2405718. doi: 10.1002/advs.202405718 (PMC11633337; doi:10.1002/advs.202405718)
Supplement: Supplementary file 1 — Supporting Information [file ADVS-11-2405718-s002.pdf]

ADVANCED SCIENCE
Open Access

Supporting Information

for *Adv. Sci.*, DOI 10.1002/advs.202405718

Emergent Ferroelectric Nematic and Heliconical Ferroelectric Nematic States in an Achiral "Straight" Polar Rod Mesogen

Hiroya Nishikawa, Daichi Okada, Dennis Kwaria, Atsuko Nihonyanagi, Motonobu Kuwayama, Manabu Hoshino and Fumito Araoka**

Supplementary Information

Emergent Ferroelectric Nematic and Heliconical Ferroelectric Nematic States in an Achiral "Straight" Polar Rod Mesogen

Hiroya Nishikawa, Daichi Okada, Dennis Kwaria, Atsuko Nihonyanagi, Motonobu Kuwayama, Manabu Hoshino and Fumito Araoka**

*To whom correspondence should be addressed.

E-mail: hiroya.nishikawa@riken.jp (H.N.) and fumito.araoka@riken.jp (F.A.)

Table of contents

Methods

1. **General and materials** S2
2. **Synthesis and characterization** S6

Supporting Notes (Notes S1–S5) S15

Supporting Figures (Figures S1–S31) S20

Supporting Tables (Tables S1 and S2) S51

Supporting References S53

Methods

1. General and materials

Nuclear magnetic resonance (NMR) spectroscopy: ^{1}H, ^{13}C, and ^{19}F NMR spectra were recorded on JNM-ECZ500 (JEOL) operating at 500 MHz, 126 MHz, and 471 MHz for ^{1}H [^{1}H{^{19}F}], ^{13}C{^{1}H} [^{13}C{^{1}H,^{19}F}] and ^{19}F [^{19}F{^{1}H}] NMR, respectively, using the TMS (trimethylsilane) as an internal standard for ^{1}H NMR and the deuterated solvent for ^{13}C NMR. The absolute values of the coupling constants are given in Hz, regardless of their signs. Signal multiplicities were abbreviated by s (singlet), d (doublet), t (triplet), q (quartet), quint (quintet), sext (sextet), and dd (double–doublet), respectively.

High-resolution mass (HRMS) spectroscopy: The quadrupole time-of-flight high-resolution mass spectrometry (QTOF-HRMS) was performed on COMPACT (BRUKER). The calibration was carried out using LC/MS tuning mix, for APCI/APPI (Agilent Technologies).

Density Functional Theory (DFT) Calculation: Calculations were performed using the Chem3D (pro, 22.2.0.3300) and Gaussian 16 (G16, C.01) softwares (installed at the RIKEN Hokusai GreatWave Supercomputing facility) for MM2 and DFT calculations, respectively. GaussView 6 (6.0.16) software was used to visually analyze the calculation results. Positions of hydrogens of molecules were optimized using the B3LYP/6-31G++ level Gaussian 16 program package. [S1] Dipole moments of molecules were calculated using b3lyp/6-311+g(d,p) and B3LYP-gd3bj which were added for empirical dispersion corrections to the standard B3LYP. The calculation method is as follows: opt=tight freq b3lyp/6-311+g(d,p) geom=connectivity empiricaldispersion=gd3bj int=ultrafine.

Polarized optical microscopy: Polarized optical microscopy was performed on a polarizing microscope (Eclipse LV100 POL, Nikon) with a hot stage (HSC402, INSTEC) on the rotation stage. Unless otherwise noted, the sample temperature was controlled using the INSTEC temperature controller and a liquid nitrogen cooling system pump (mk2000 and LN2-P/LN2-D2, INSTEC).

Differential scanning calorimetry (DSC). Differential scanning calorimetry was performed on a calorimeter (DSC1, Mettler-Toledo). Rate, 5 and 20 K min^{-1}. Cooling/heating profiles were recorded and analyzed using the Mettler-Toledo STARe software system.

Dielectric spectroscopy. Dielectric relaxation spectroscopy was performed ranging between 1 Hz and 1 MHz using an impedance/gain-phase analyzer (SI 1260, Solartron Metrology) and a dielectric interface (SI 1296, Solartron Metrology). Prior to starting the measurement of the LC sample, the capacitance of the empty cell was determined as a reference.

***P–E* hysteresis measurement.** *P–E* hysteresis measurements were performed in the temperature range of the N_F phase under a triangular-wave electric field (10 kV cm^{-1}, 200 Hz) using a ferroelectricity evaluation system (FCE 10, TOYO Corporation), which is composed of an arbitrary waveform generator (2411B), an IV/QV amplifier (model 6252) and a simultaneous A/D USB device (DT9832).

SHG measurement. The SHG investigation was carried out using a Q-switched DPSS Nd: YAG laser (FQS-400-1-Y-1064, Elforlight) at λ = 1064 nm with a 5 ns pulse width (pulse energy: 400 μJ). The primary beam was incident on the LC cell followed by the detection of the SHG signal. The electric field was applied normally to the LC cell. The optical setup is shown in **Figure S1**. The experimental details are mentioned in **Supplementary Note 1**.

Wide-angle X-ray scattering (WAXD) measurement. Two-dimensional WAXD measurement was carried out using the NANOPIX system (Rigaku). The samples held in a Cu holder (diameter: 2.0 mm, thickness: 2 mm) were measured at a constant temperature using a temperature controller (mk2000B, INSTEC) and a hot stage. The measurement set up is shown in **Figure S2**. The scattering vector q ($q = 4\pi\sin\theta\ \lambda^{-1}$; 2θ and λ = scattering angle and wavelength of an incident X-ray beam [1.54 Å (NANOPIX)] and position of an incident X-ray beam on the detector were calibrated using several orders of layer diffractions from silver behenate (d = 58.380 Å). The sample-to-detector distances were 95.4 mm (NANOPIX), where acquired scattering 2D images were integrated along the Debye–Scherrer ring by using software (Igor Pro with Nika-plugin), affording the corresponding one-dimensional profiles.

Single crystal X-ray scattering (SC-XRD) measurement.

A single crystal of **6BOE** with dimensions of 0.29 × 0.09 × 0.04 mm was used for SC-XRD measurement. Diffraction data were collected using Rigaku Synergy-i single crystal X-ray diffractometer equipped with a sealed-tube CuKα (λ = 1.54184 Å) X-ray source and a pixel array type detector. The single crystal was mounted on the goniometer head of the diffractometer and cooled to 100 K by a cold nitrogen stream from Oxford Cryosystem Cryostream 800 for measurement. Collected data were integrated, corrected (including the

Lorentzian-polarization and the absorption correction), and scaled using the program *CrysAlisPro*.[S2] All crystal structures were solved using the program *SHELXT*[S3] and refined (against $|F^2|$)using the program *SHELXL*.[S4] Crystal system: monoclinic. Space group $P2_1/c$. Unit cell parameters: a = 29.5406(2) Å, b = 25.3902(2) Å, c = 14.49345(13) Å, β = 91.1515(7)°, V = 10868.49(15) Å^3. Calculated Density: 1.431 g cm^{-1}. $2\theta_{max}$: 134.16°. Scan mode: ω scans. No. of reflections: 72370 (measured) and 28833 (independent). μ: 1.026 mm^{-1}. T_{min}, T_{max}: 0.510, 1.000. No. of parameters: 1518. Z = 16. R ($F^2 > 2\sigma(F^2)$) = 0.0771, wR (all reflections) = 0.1808. and Residual electron density: 0.872(max), −0.257(min). CCDC reference number: 2356894.

Reflection Spectra measurement. The reflection spectra were recorded using a fiber optic spectrometer (USB4000, Ocean optics). The rotation angle dependence of reflection spectra was recorded using the optical setup as shown in **Figure S3**. For experiments on reflection color tuning, we employed a multifunction generator (WF1973, nf) and a microscope spectroscopic method using the polarizing microscope (Eclipse LV100 POL, Nikon) quipped with the fiber optic spectrometer (USB4000, Ocean optics).

Birefringence measurement. Birefringence (Δn) measurement was performed using the microscope spectroscopic method using the polarizing microscope (Eclipse LV100 POL, Nikon) quipped with the fiber optic spectrometer (USB4000, Ocean optics). The transmittance of light under para-polarization conditions was observed. The obtained transmittance data was fitted by combination of eq. (1) and Cauchy's eq. (2),

$$I = I_0 cos^2\left(\frac{\pi d \Delta n(\lambda)}{\lambda}\right) \tag{1}$$

$$\Delta n(\lambda) = A + B/\lambda^2 + C/\lambda^4 \tag{2}$$

, where I and λ are intensity of transmitted light, wavelength, respectively while, A, B and C are Cauchy's coefficients. In this paper, we adopted $\Delta n(550)$.

FTIR spectra measurement. FTIR absorbance spectra were recorded using AIM-9000 FTIR microscope systems (SHIMADZU) with simultaneous observation of light microscope images. The LC sample was injected in a BaF_2 cell. The corresponding FTIR spectra was simulated by DFT calculation as mentioned above.

Information of used liquid crystalline (LC) cells:

Bare glass cell (EHC):

- Experiments: POM (thickness: 10.0 μm) and spectra studies (thickness: 5.0, 10.0 μm)

Bare glass cell (homemade):

- Sandwich-type film using two cover glasses
- Experiments: Spectra studies (thickness: 10.0 μm)

Antiparallel-rubbed cell (EHC):

- PI-coated type
- Alignment layer: LX-1400
- Experiments: POM studies (thickness: 2.0, 5.0 μm)

Parallel-rubbed cell (EHC):

- PI-coated type
- Alignment layer: LX-1400
- Experiments: POM studies (thickness: 2.0, 5.0 μm), birefringence measurement (thickness: 2.0 μm).

ITO glass cell (EHC):

- ITO-coated type, electrode area: 5 × 10 mm
- Experiments: POM (thickness: 10.0 μm) and DR (thickness: 9.0 μm) studies; reflection color tuning (thickness: 10.0 μm).

IPS cell (EHC):

- PI-coated type, electrode distance: 500 μm, electrode length: 18 mm
- Alignment layer: LX-1400
- Rubbing condition: antiparallel
- Experiments: *PE* hysteresis (thickness: 5.0 μm)

IPS cell (homemade):

- Au-coated type (Cr bottom layer: 5 nm; Au top layer: 50 nm), electrode distance: 1 mm, electrode length: 13 mm, electrode width, 2 mm
- Alignment layer: AL1254
- Rubbing condition: antiparallel (rubbing depth: 100 μm, rubbing times: 10)
- Experiments: SHG (thickness: 5.0 μm) studies

2. Synthesis of nBOE (n = 1–8).

2.1. Synthetic route

nBOE (n = 1–8) used in this paper were synthesized by following pathway (**Scheme S1**). The blue and green colored pathway indicate solution chemical (SC) and mechanochemical (MC) synthesis, respectively. The detail of MC synthesis has been reported in our previous papers.[S5,S6]

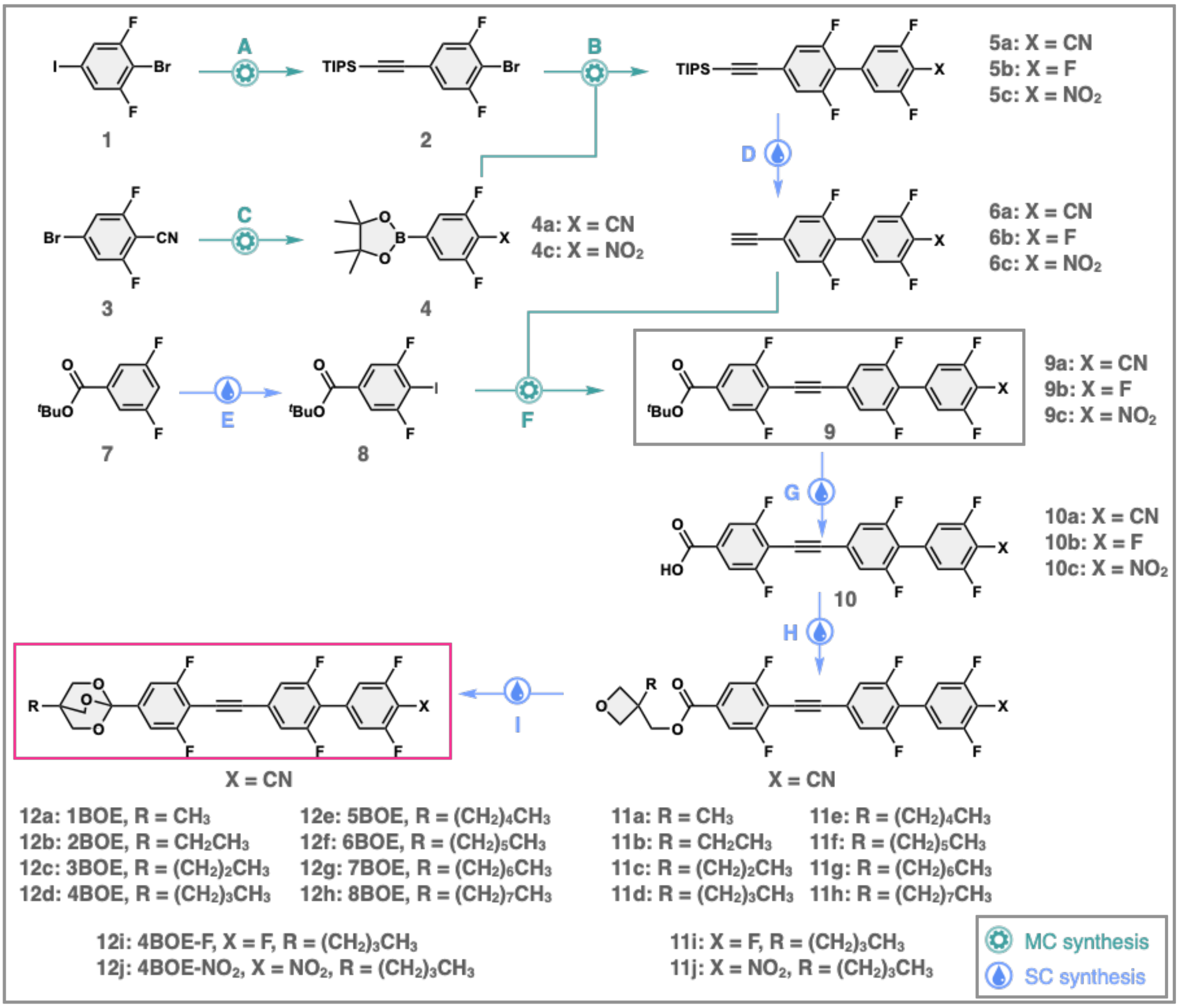

Scheme S1 Synthetic pathway of **nBOE** (n = 1–8), **4BOE-F** and **4BOE-NO₂**. A) MC-Sonogashira coupling, B) MC-Suzuki coupling, C) MC-Miyaura coupling, D) deprotection of TIPS group with TBAF, E) Iodization, F) MC-Sonogashira coupling, G) deprotection of ^{t}BuO group, H) esterification, I) orthoesterification.

2.2. SC/MC Synthesis

2.2.1. ***Synthesis of ((4-bromo-3,5-difluorophenyl)ethynyl)triisopropylsilane (2)***

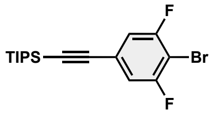

Compound **2** was synthesized by following the procedure described in our previous paper.[S5]

2.2.2. ***Synthesis of compounds (4a–c)***

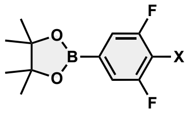

Compounds **4a–c** were synthesized by following the procedure described in our previous paper.[S5]

2,6-difluoro-4-(4,4,5,5-tetramethyl-1,3,2-dioxaborolan-2-yl)benzonitrile (4a)

The $^{1}H\{^{19}F\}$-NMR spectrum is in accordance with the literature.[S5]

2-(3,5-Difluoro-4-nitrophenyl)-4,4,5,5-tetramethyl-1,3,2-dioxaborolane (4c)

$^{1}H\{^{19}F\}$-NMR (500 MHz, $CDCl_3$): δ 7.48 (s, 2H), 1.35 (s, 12H)

$^{19}F\{^{1}H\}$-NMR (471 MHz, $CDCl_3$): δ −120.1

2.2.3. ***Synthesis of compounds (5a–c)***

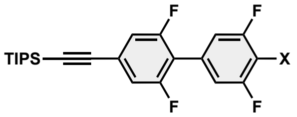

Compounds **5a–c** were synthesized by following the procedure described in our previous paper.[S5]

2',3,5,6'-Tetrafluoro-4'-((triisopropylsilyl)ethynyl)-[1,1'-biphenyl]-4-carbonitrile (5a)

The $^{1}H\{^{19}F\}$-NMR spectrum is in accordance with the literature.[S5]

Triisopropyl((2,3',4',5',6-pentafluoro-[1,1'-biphenyl]-4-yl)ethynyl)silane (5b)

$^{1}H\{^{19}F\}$-NMR (500 MHz, $CDCl_3$): δ 7.11-7.10 (m, 4H), 1.14-1.13 (m, 18H)

$^{19}F\{^{1}H\}$-NMR (471 MHz, $CDCl_3$): δ −114.4, −134.3 (d, J = 18.4 Hz), −159.7 (d, J = 20.3Hz)

Triisopropyl((2,3',5',6-tetrafluoro-4'-nitro-[1,1'-biphenyl]-4-yl)ethynyl)silane (5c)

$^{1}H\{^{19}F\}$-NMR (500 MHz, $CDCl_3$): δ 7.24 (s, 2H), 7.14 (s, 2H), 1.15-1.12 (m, 18H)

$^{19}F\{^{1}H\}$-NMR (471 MHz, $CDCl_3$): δ −113.9, −118.4

2.2.4. ***General procedure of compounds (6a–c)***

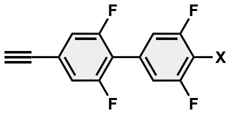

Compounds **6a–c** were synthesized by following the procedure described in our previous paper.[S5]

4'-Ethynyl-2',3,5,6'-tetrafluoro-[1,1'-biphenyl]-4-carbonitrile (6a)

The $^{1}H\{^{19}F\}$-NMR spectrum is in accordance with the literature.[S5]

4-Ethynyl-2,3',4',5',6-pentafluoro-1,1'-biphenyl (6b)

$^{1}H\{^{19}F\}$-NMR (500 MHz, $CDCl_3$): δ 7.13-7.11 (m, 4H), 3.22 (s, 1H)

$^{19}F\{^{1}H\}$-NMR (471 MHz, $CDCl_3$): δ −113.8, −132.6, −134.1 (d, J = 18.4 Hz), −159.4 (dd, J = 20.3 Hz)

4-Ethynyl-2,3',5',6-tetrafluoro-4'-nitro-1,1'-biphenyl (6c)

$^{1}H\{^{19}F\}$-NMR (500 MHz, $CDCl_3$): δ 7.25 (s, 2H), 7.17 (s, 2H), 3.28 (s, 1H)

$^{19}F\{^{1}H\}$-NMR (471 MHz, $CDCl_3$): δ −113.2, −118.3

2.2.5. *Synthesis of tert-butyl 3,5-difluoro-4-iodobenzoate (8)*

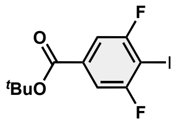

LDA was prepared by adding n-BuLi (27.6 mL, 43 mmol, 1.56 M in hexane) to a solution of diisopropylamine (6.0 mL, 43 mmol) in 200 mL of THF at –78 °C under Ar. The solution was stirred 30 min at 0 °C and then cooled again to –78 °C. To the LDA solution was added a solution of tert-butyl 3,5-difluorobenzoate (7.7 g, 35.8 mmol) in THF (10 mL), and the mixture was stirred for 30 min. Iodine (10 g, 39.4 mmol) was then added to the mixture. After 5 min stirring, the reaction was warmed to 0 °C and stirred for 1.5 h. The mixture was poured into ice water and extracted with EtOAc and hexane (1:1), the organic layer was dried over Na_2SO_4. The solution was filtered through a short pad of silica gel and the short pad was further washed with mixed solvent (hexane / CH_2Cl_2 =1:1). The solution was concentrated and dried under reduced pressure to yield a brown solid (9.8 g, 28.7 mmol, 80.2%)

$^{1}H\{^{19}F\}$-NMR (500 MHz, $CDCl_3$): δ 7.47 (s, 2H), 1.59 (s, 9H)

$^{19}F\{^{1}H\}$-NMR (471 MHz, $CDCl_3$): δ −91.2

Synthesis of compounds (9a–c)

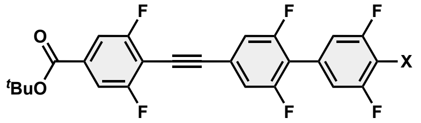

Compounds **9a–c** were synthesized by following the procedure described in our previous paper.[S5].

tert-Butyl 4-((4'-cyano-2,3',5',6-tetrafluoro-[1,1'-biphenyl]-4-yl)ethynyl)-3,5-difluorobenzoate (9a)

$^{1}H\{^{19}F\}$-NMR (500 MHz, $CDCl_3$): δ 7.57 (s, 2H), 7.28 (s, 2H), 7.23 (s, 2H), 1.60 (s, 9H)

$^{19}F\{^{1}H\}$-NMR (471 MHz, $CDCl_3$): δ −103.4, −105.8, −112.9

tert-Butyl 3,5-difluoro-4-((2,3',4',5',6-pentafluoro-[1,1'-biphenyl]-4-yl)ethynyl)benzoate (9b)

$^{1}H\{^{19}F\}$-NMR (500 MHz, $CDCl_3$): δ 7.57 (s, 2H), 7.24 (s, 2H), 7.15 (d, J = 6.2 Hz, 2H), 1.60 (s, 9H)

$^{19}F\{^{1}H\}$-NMR (471 MHz, $CDCl_3$): δ −105.9, −113.5, −134.1 (d, J = 22 Hz), −159.3 (dd, J = 20.3 Hz)

tert-Butyl 3,5-difluoro-4-((2,3',5',6-tetrafluoro-4'-nitro-[1,1'-biphenyl]-4-yl)ethynyl)benzoate (9c)

$^{1}H\{^{19}F\}$-NMR (500 MHz, $CDCl_3$): δ 7.57 (s, 2H), 7.29 (s, 2H), 7.28 (s, 2H), 1.61 (s, 9H)

$^{19}F\{^{1}H\}$-NMR (471 MHz, $CDCl_3$): δ −105.8, −113.0, −118.2

2.2.6. ***Synthesis of compounds (10a–c)***

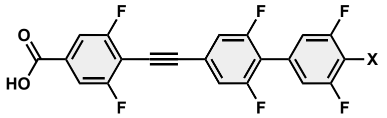

Example: To a solution of *tert*-butyl ester (1.57 g, 3.22 mmol) in CH_2Cl_2 (18 mL) was added TFA (6 mL) at 0 °C under Ar. After stirring for 1h, the resulting solution was warmed to r.t. and further stirred for 3.5 h. The reaction was quenched by the addition of H_2O and extracted with CH_2Cl_2. The combined organic layer was dried over Na_2SO_4 and concentrated in vacuo afforded **10a** as a pale brown solid (1.34 g, 3.11 mmol, 96.6 %). The other compounds were synthesized according to above procedure.

4-((4'-Cyano-2,3',5',6-tetrafluoro-[1,1'-biphenyl]-4-yl)ethynyl)-3,5-difluorobenzoic acid (10a)

$^{1}H\{^{19}F\}$-NMR (500 MHz, acetone-*d*6): δ 7.73 (s, 2H), 7.61 (s, 2H), 7.53 (s, 2H)

$^{19}F\{^{1}H\}$-NMR (471 MHz, acetone-*d*6): δ −106.5, −107.2, −113.9

3,5-Difluoro-4-((2,3',4',5',6-pentafluoro-[1,1'-biphenyl]-4-yl)ethynyl)benzoic acid (10b)

$^{1}H\{^{19}F\}$-NMR (500 MHz, acetone-*d*6): δ 7.72 (s, 2H), 7.47-7.46 (m, 4H)

$^{19}F\{^{1}H\}$-NMR (471 MHz, acetone-*d*6): δ −107.3, −114.3, −136.3 (d, J = 18.4 Hz), −161.9 (d, J = 20.3 Hz)

3,5-Difluoro-4-((2,3',5',6-tetrafluoro-4'-nitro-[1,1'-biphenyl]-4-yl)ethynyl)benzoic acid (10c)

$^{1}H\{^{19}F\}$-NMR (500 MHz, acetone-*d*6): δ 7.73 (s, 2H), 7.67 (s, 2H), 7.53 (s, 2H)

$^{19}F\{^{1}H\}$-NMR (471 MHz, acetone-*d*6): δ −107.2, −113.9, −120.7

2.2.7. ***Synthesis of compounds (11a–j)***

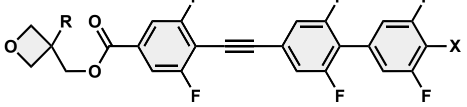

Example: To a solution of carboxylic acid (620 mg, 1.44 mmol) in CH_2Cl_2 (17 mL) were added 3-pentyl-3-oxetanemethanol (266 mg, 1.68 mmol), EDAC-HCl (322 mg, 1.68 mmol), and DMAP (51.3 mg, 0.42 mmol) at 0 °C under Ar. After 0.5 h stirring, the solution was warmed to r.t. and further stirred for 2 h. The reaction was quenched by the addition of H_2O and extracted with CH_2Cl_2. The combined organic layer was dried over Na_2SO_4

and concentrated. The crude products were purified by column chromatography on silica gel (hexane/ CH_2Cl_2, 1:4 to 0:100) afforded **11e** as a white solid (360 mg, 0.64 mmol, 99%). The other compounds were synthesized according to above procedure.

(3-Methyloxetan-3-yl)methyl 3,5-difluoro-4-((2,3',5',6-tetrafluoro-4'-nitro-[1,1'-biphenyl]-4-yl)ethynyl)benzoate (11a)

$^{1}H\{^{19}F\}$-NMR (500 MHz, $CDCl_3$): δ 7.65 (s, 2H), 7.29 (s, 2H), 7.23 (s, 2H), 4.56 (dd, J = 62.6, 6.0 Hz, 4H), 4.45 (s, 2H), 1.44 (s, 3H)

$^{19}F\{^{1}H\}$-NMR (471 MHz, $CDCl_3$): δ −103.4, −105.0, −112.8

(3-Ethyloxetan-3-yl)methyl 3,5-difluoro-4-((2,3',5',6-tetrafluoro-4'-nitro-[1,1'-biphenyl]-4-yl)ethynyl)benzoate (11b)

$^{1}H\{^{19}F\}$-NMR (500 MHz, $CDCl_3$): δ 7.64 (s, 2H), 7.29 (s, 2H), 7.23 (s, 2H), 4.54 (dd, J = 28.8, 6.2 Hz, 4H), 4.50 (s, 2H), 1.85 (q, J = 7.4 Hz, 2H), 0.98 (t, J = 7.5 Hz, 3H)

$^{19}F\{^{1}H\}$-NMR (471 MHz, $CDCl_3$): δ −103.4, −104.9, −112.9

(3-Propyloxetan-3-yl)methyl 4-((4'-cyano-2,3',5',6-tetrafluoro-[1,1'-biphenyl]-4-yl)ethynyl)-3,5-difluorobenzoate (11c)

$^{1}H\{^{19}F\}$-NMR (500 MHz, $CDCl_3$): δ 7.64 (s, 2H), 7.29 (s, 2H), 7.23 (s, 2H), 4.55 (dd, J = 21.8, 6.0 Hz, 4H), 4.50 (s, 2H), 1.80-1.77 (m, 2H), 1.41-1.33 (m, 2H), 0.99 (t, J = 7.5 Hz, 3H)

$^{19}F\{^{1}H\}$-NMR (471 MHz, $CDCl_3$): δ −103.4, −104.9, −112.7

(3-Butyloxetan-3-yl)methyl 4-((4'-cyano-2,3',5',6-tetrafluoro-[1,1'-biphenyl]-4-yl)ethynyl)-3,5-difluorobenzoate (11d)

$^{1}H\{^{19}F\}$-NMR (500 MHz, $CDCl_3$): δ 7.64 (s, 2H), 7.29 (s, 2H), 7.23 (s, 2H), 4.57 (d, J = 6.2 Hz, 2H), 4.52 (d, J = 6.2 Hz, 2H), 4.49 (s, 2H), 1.82-1.79 (m, 2H), 1.42-1.30 (m, 4H), 0.94 (t, J = 7.1 Hz, 3H)

$^{19}F\{^{1}H\}$-NMR (471 MHz, $CDCl_3$): δ −103.4, −104.9, −112.7

(3-Pentyloxetan-3-yl)methyl 4-((4'-cyano-2,3',5',6-tetrafluoro-[1,1'-biphenyl]-4-yl)ethynyl)-3,5-difluorobenzoate (11e)

$^{1}H\{^{19}F\}$-NMR (500 MHz, $CDCl_3$): δ 7.64 (s, 2H), 7.29 (s, 2H), 7.23 (s, 2H), 4.54 (dd, J = 27.0, 6.0 Hz, 4H), 4.49 (s, 2H), 1.79 (m, 2H), 1.37-1.25 (m, 6H), 0.91 (t, J = 6.8 Hz, 3H)

$^{19}F\{^{1}H\}$-NMR (471 MHz, $CDCl_3$): δ −103.3, −104.9, −112.7

(3-Hexyloxetan-3-yl)methyl 4-((4'-cyano-2,3',5',6-tetrafluoro-[1,1'-biphenyl]-4-yl)ethynyl)-3,5-difluorobenzoate (11f)

$^{1}H\{^{19}F\}$-NMR (500 MHz, $CDCl_3$): δ 7.64 (s, 2H), 7.29 (s, 2H), 7.23 (s, 2H), 4.57-4.49 (m, 6H), 1.81-1.78 (m, 2H), 1.32 (q, J = 5.9 Hz, 8H), 0.89 (t, J = 6.8 Hz, 3H)

$^{19}F\{^{1}H\}$-NMR (471 MHz, $CDCl_3$): δ −103.3, −104.9, −112.7

(3-Heptyloxetan-3-yl)methyl 4-((4'-cyano-2,3',5',6-tetrafluoro-[1,1'-biphenyl]-4-yl)ethynyl)-3,5-difluorobenzoate (11g)

$^{1}H\{^{19}F\}$-NMR (500 MHz, $CDCl_3$): δ 7.64 (s, 2H), 7.29 (s, 2H), 7.23 (s, 2H), 4.57-4.49 (m, 6H), 1.79 (d, J = 15.9 Hz, 2H), 1.32-1.23 (m, 12H), 0.90-0.87 (m, 3H)

$^{19}F\{^{1}H\}$-NMR (471 MHz, $CDCl_3$): δ −103.3, −104.9, −112.8

(3-Octyloxetan-3-yl)methyl 4-((4'-cyano-2,3',5',6-tetrafluoro-[1,1'-biphenyl]-4-yl)ethynyl)-3,5-difluorobenzoate (11h)

$^{1}H\{^{19}F\}$-NMR (500 MHz, $CDCl_3$): δ 7.64 (s, 2H), 7.29 (s, 2H), 7.23 (s, 2H), 4.54 (dd, J = 26.5, 6.0 Hz, 3H), 4.49 (s, 2H), 1.81-1.78 (m, J = 7.6 Hz, 2H), 1.33-1.27 (m, 12H), 0.88 (t, J = 6.8 Hz, 3H)

$^{19}F\{^{1}H\}$-NMR (471 MHz, $CDCl_3$): δ −103.3, −104.9, −112.7

(3-Butyloxetan-3-yl)methyl 3,5-difluoro-4-((2,3',4',5',6-pentafluoro-[1,1'-biphenyl]-4-yl)ethynyl)benzoate (11i)

$^{1}H\{^{19}F\}$-NMR (500 MHz, $CDCl_3$): δ 7.64 (s, 2H), 7.25 (s, 2H), 7.15 (d, J = 6.0 Hz, 2H), 4.54 (dd, J = 27.0, 6.0 Hz, 3H), 4.49 (s, 2H), 1.82-1.79 (m, 2H), 1.41-1.28 (m, 4H), 0.94 (t, J = 7.3 Hz, 3H)

$^{19}F\{^{1}H\}$-NMR (471 MHz, $CDCl_3$): δ −105.0, −113.4, −134.0 (d, J = 18.4 Hz), −159.2 (d, J = 20.3 Hz)

(3-Butyloxetan-3-yl)methyl 3,5-difluoro-4-((2,3',5',6-tetrafluoro-4'-nitro-[1,1'-biphenyl]-4-yl)ethynyl)benzoate (11j)

^{1}H-NMR (500 MHz, $CDCl_3$): δ 7.65 (d, J = 7.0 Hz, 2H), 7.30-7.26 (m, 4H), 4.54 (dd, J = 26.8, 6.3 Hz, 4H), 4.49 (s, 2H), 1.82-1.79 (m, 2H), 1.41-1.28 (m, 4H), 0.94 (t, J = 7.3 Hz, 3H)

$^{19}F\{^{1}H\}$-NMR (471 MHz, $CDCl_3$): δ −104.9, −112.9, −118.2

2.2.8. *Synthesis of nBOE (12a–12h), 4BOE-F (12i) and 4BOE-NO₂ (12j)*

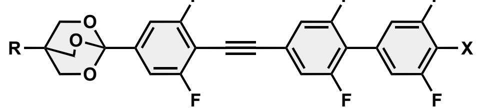

Example: To a solution of ester (663 mg, 1.19 mmol) in CH_2Cl_2 (6 mL) was added BF_3-OEt_2 (14.7 µL, 0.12 mmol) at 0 °C under Ar. After 1.5 h stirring, the

solution was warmed to r.t. and further stirred for 1.5 h. The reaction was quenched by the addition of Et_3N (828 μL, 5.94 mmol) and H_2O, and extracted with CH_2Cl_2. The combined organic layer was dried over Na_2SO_4 and concentrated. The residue was purified by a neutral alumina column chromatography on silica gel (hexane / $CHCl_3$ 1:4 to 1:1) afforded **11d** as a white solid (512 mg, 0.92 mmol, 77%). The other compounds were synthesized according to above procedure. All compounds were recrystallized twice from hexane/CH_2Cl_2.

4'-((2,6-Difluoro-4-(4-methyl-2,6,7-trioxabicyclo[2.2.2]octan-1-yl)phenyl)ethynyl)-2',3,5,6'-tetrafluoro-[1,1'-biphenyl]-4-carbonitrile (12a)

The $^{1}H\{^{19}F\}$-NMR spectrum is in accordance with the literature.[S5]

4'-((2,6-Difluoro-4-(4-ethyl-2,6,7-trioxabicyclo[2.2.2]octan-1-yl)phenyl)ethynyl)-2',3,5,6'-tetrafluoro-[1,1'-biphenyl]-4-carbonitrile (12b)

The $^{1}H\{^{19}F\}$-NMR spectrum is in accordance with the literature.[S5]

4'-((2,6-Difluoro-4-(4-propyl-2,6,7-trioxabicyclo[2.2.2]octan-1-yl)phenyl)ethynyl)-2',3,5,6'-tetrafluoro-[1,1'-biphenyl]-4-carbonitrile, 3BOE (12c)

$^{1}H\{^{19}F\}$-NMR (500 MHz, $CDCl_3$): 7.25 (s, 2H), 7.24 (s, 2H), 7.23 (s, 2H), 4.11 (s, 6H), 1.31-1.22 (m, 4H), 0.94 (t, J = 6.7 Hz, 3H)

$^{19}F\{^{1}H\}$-NMR (471 MHz, $CDCl_3$): δ −103.5, −106.3, −113.2

$^{13}C\{^{1}H,^{19}F\}$-NMR (126 MHz, $CDCl_3$): 162.8, 162.5, 159.1, 141.2, 136.6, 125.9, 115.6, 115.5, 114.2, 109.4, 108.9, 106.3, 101.6, 96.0, 92.3, 80.0, 72.1, 33.5, 31.9, 16.6, 14.7

QTOF-HRMS (*m/z*, $[M+H]^+$) calc for 544.1348; found, 544.1370

4'-((2,6-Difluoro-4-(4-butyl-2,6,7-trioxabicyclo[2.2.2]octan-1-yl)phenyl)ethynyl)-2',3,5,6'-tetrafluoro-[1,1'-biphenyl]-4-carbonitrile, 4BOE (12d)

$^{1}H\{^{19}F\}$-NMR (500 MHz, $CDCl_3$): 7.25 (s, 2H), 7.24 (s, 2H), 7.23 (s, 2H), 4.11 (s, 6H), 1.35-1.19 (m, 6H), 0.92 (t, J = 7.2 Hz, 3H)

$^{19}F\{^{1}H\}$-NMR (471 MHz, $CDCl_3$): δ −103.5, −106.3, −113.2

$^{13}C\{^{1}H,^{19}F\}$-NMR (126 MHz, $CDCl_3$): 162.8, 162.5, 159.1, 141.2, 136.6, 125.9, 115.6, 115.5, 114.2, 109.4, 109.0, 106.3, 101.6, 96.0, 92.3, 80.1, 72.1, 33.4, 29.4, 25.3, 23.3, 13.8

QTOF-HRMS (*m/z*, $[M+H]^+$) calc for 558.1504; found, 558.1502.

4'-((2,6-Difluoro-4-(4-pentyl-2,6,7-trioxabicyclo[2.2.2]octan-1-yl)phenyl)ethynyl)-2',3,5,6'-tetrafluoro-[1,1'-biphenyl]-4-carbonitrile, 5BOE (12e)

$^{1}H\{^{19}F\}$-NMR (500 MHz, $CDCl_3$): 7.25 (s, 2H), 7.24 (s, 2H), 7.23 (s, 2H), 4.11 (s, 6H), 1.35-1.24 (m, 8H), 0.90 (t, J = 7.1 Hz, 3H)

$^{19}F\{^1H\}$-NMR (471 MHz, $CDCl_3$): δ −103.5, −106.3, −113.2

$^{13}C\{^1H,^{19}F\}$-NMR (126 MHz, $CDCl_3$): 162.8, 162.5, 159.1, 141.2, 136.6, 125.9, 115.6, 115.5, 114.2, 109.4, 109.0, 106.3, 101.6, 96.0, 92.3, 80.1, 72.1, 33.5, 32.3, 29.6, 22.8, 22.3, 13.9

QTOF-HRMS (*m*/*z*, $[M+H]^+$) calc for 572.1660; found, 572.1669

4'-((2,6-Difluoro-4-(4-hexyl-2,6,7-trioxabicyclo[2.2.2]octan-1-yl)phenyl)ethynyl)-2',3,5,6'-tetrafluoro-[1,1'-biphenyl]-4-carbonitrile, 6BOE (12f)

$^1H\{^{19}F\}$-NMR (500 MHz, $CDCl_3$): 7.25 (s, 2H), 7.24 (s, 2H), 7.23 (s, 2H), 4.10 (s, 6H), 1.32-1.21 (m, 10H), 0.89 (t, J = 6.8 Hz, 3H)

$^{19}F\{^1H\}$-NMR (471 MHz, $CDCl_3$): δ −103.5, −106.3, −113.2

$^{13}C\{^1H,^{19}F\}$-NMR (126 MHz, $CDCl_3$): 162.8, 162.5, 159.1, 141.2, 136.6, 125.9, 115.6, 115.5, 114.2, 109.4, 109.0, 106.3, 101.6, 96.0, 92.3, 80.1, 72.1, 33.5, 31.5, 29.8, 29.7, 23.1, 22.5, 14.0

QTOF-HRMS (*m*/*z*, $[M+H]^+$) calc for 586.1817; found, 586.1810

4'-((2,6-Difluoro-4-(4-heptyl-2,6,7-trioxabicyclo[2.2.2]octan-1-yl)phenyl)ethynyl)-2',3,5,6'-tetrafluoro-[1,1'-biphenyl]-4-carbonitrile, 7BOE (12g)

$^1H\{^{19}F\}$-NMR (500 MHz, $CDCl_3$): 7.25 (s, 2H), 7.24 (s, 2H), 7.23 (s, 2H), 4.10 (s, 6H), 1.32-1.24 (m, 12H), 0.89 (t, J = 6.9 Hz, 3H)

$^{19}F\{^1H\}$-NMR (471 MHz, $CDCl_3$): δ −103.5, −106.3, −113.2

$^{13}C\{^1H,^{19}F\}$-NMR (126 MHz, $CDCl_3$): 162.8, 162.5, 159.1, 141.2, 136.6, 125.9, 115.6, 115.5, 114.2, 109.4, 109.0, 106.3, 101.6, 96.0, 92.3, 80.1, 72.1, 33.5, 31.7, 30.1, 29.7, 29.0, 23.2, 22.6, 14.1

QTOF-HRMS (*m*/*z*, $[M+H]^+$) calc for 600.1973; found, 600.1970

4'-((2,6-Difluoro-4-(4-octyl-2,6,7-trioxabicyclo[2.2.2]octan-1-yl)phenyl)ethynyl)-2',3,5,6'-tetrafluoro-[1,1'-biphenyl]-4-carbonitrile, 8BOE (12h)

$^1H\{^{19}F\}$-NMR (500 MHz, $CDCl_3$): 7.25 (s, 2H), 7.24 (s, 2H), 7.23 (s, 2H), 4.10 (s, 6H), 1.32-1.24 (m, 14H), 0.89 (t, J = 6.9 Hz, 3H)

$^{19}F\{^1H\}$-NMR (471 MHz, $CDCl_3$): δ −103.4, −106.3, −113.2

$^{13}C\{^1H,^{19}F\}$-NMR (126 MHz, $CDCl_3$): 162.8, 162.5, 159.1, 141.2, 136.6, 125.9, 115.6, 115.5, 114.2, 109.4, 108.9, 106.3, 101.6, 96.0, 92.3, 80.1, 72.1, 33.5, 31.8, 30.2, 29.7, 29.3, 29.2, 23.2, 22.6, 14.1

QTOF-HRMS (*m*/*z*, $[M+H]^+$) calc for 614.2130; found, 614.2141

4-Butyl-1-(3,5-difluoro-4-((2,3',4',5',6-pentafluoro-[1,1'-biphenyl]-4-yl)ethynyl)phenyl)-2,6,7-trioxabicyclo[2.2.2]octane, 4BOE-F (12i)

$^{1}H\{^{19}F\}$-NMR (500 MHz, $CDCl_3$): δ 7.22 (d, J = 9.3 Hz, 4H), 7.14 (d, J = 6.2 Hz, 2H), 4.10 (s, 6H), 1.35-1.17 (m, 6H), 0.92 (t, J = 7.3 Hz, 3H)

$^{19}F\{^{1}H\}$-NMR (471 MHz, $CDCl_3$): δ −106.5, −113.8, −134.2 (d, J = 18.4 Hz), −159.5 (dd, J = 20.3 Hz)

$^{13}C\{^{1}H,^{19}F\}$-NMR (126 MHz, $CDCl_3$): $^{13}C\{^{1}H\}$-NMR (126 MHz, $CDCl_3$): δ 162.5, 159.3, 151.0, 140.9, 139.8, 124.5, 124.2, 116.6, 115.3, 114.8, 109.4, 106.4, 101.8, 96.4, 79.2, 72.1, 33.4, 29.4, 25.3, 23.3, 13.8

QTOF-HRMS (*m*/*z*, $[M+H]^+$) calc for 551.1457; found, 551.1455

4-Butyl-1-(3,5-difluoro-4-((2,3',5',6-tetrafluoro-4'-nitro-[1,1'-biphenyl]-4-yl)ethynyl)phenyl)-2,6,7-trioxabicyclo[2.2.2]octane, 4BOE-F (12j)

$^{1}H\{^{19}F\}$-NMR (500 MHz, $CDCl_3$): δ 7.27 (s, 2H), 7.26 (s, 2H), 7.24 (s, 2H), 4.11 (s, 6H), 1.35-1.19 (m, 6H), 0.92 (t, J = 7.2 Hz, 3H)

$^{19}F\{^{1}H\}$-NMR (471 MHz, $CDCl_3$): δ −106.3, −113.3, −118.3

$^{13}C\{^{1}H,^{19}F\}$-NMR (126 MHz, $CDCl_3$): δ 162.5, 159.2, 154.3, 141.2, 134.2, 129.0, 125.9, 115.5, 115.3, 115.0, 109.4, 106.3, 101.6, 96.0, 80.0, 77.2, 77.0, 76.7, 72.1, 33.4, 29.4, 25.3, 23.3, 13.8

QTOF-HRMS (*m*/*z*, $[M+H]^+$) calc for 578.1402; found, 578.1405

Supporting Notes (Notes S1–S5)

Supporting Note 1 | SHG measurements.

SHG is a powerful tool that provides detailed studies of polarization switching on surfaces and in bulk systems without being affected by ionic migration. Figure S1 shows the optical setup. In this experiment, we used a laser pulse with a repetition frequency synchronized with the AC triangular wave voltage frequency. This method prevents significant damage to the sample due to ion accumulation on the electrode surface when a direct current (DC) *E*-field is used. **Figure S4**a and b shows the SH intensity as a function of the waveform phase. In this setup, the 90° and 270° phases correspond to the maximum applied *E*-field with positive and negative polarities, respectively. To obtain a temperature-dependent SHG profile for **nBOE**, we first measured the SHG responses in various LC phases. For example, as to **4BOE**, the SHG profiles of N_F (180 °C), $^{HC}N_F$ (125 °C), and SmX_F (115 °C) were measured in advance. For **8BOE**, the SHG profiles of N (200 °C), N′ (125 °C) and SmA (115 °C) were recorded. We then measured the temperature dependence of the SHG intensity by fixing the phase that showed the maximum intensity at each phase. For **6BOE** and **8BOE**, the fixed phases were 150° and 90°, respectively. As shown in **Figure S5**a–c, strong SH activity was observed in three phases (N_F, $^{HC}N_F$, and SmX_F), indicating the presence of a polar structure. Similarly, the SHG activity of **3BOE** was detected (Figure S5d). The data for **1BOE** and **2BOE** were reported in our previous paper[S5]). Furthermore, **8BOE** also exhibited SH activity throughout various phases (N, N′, SmA). However, as seen in Figure S5e, the SH intensity increased and decreased with the absolute value of the *E*-field, suggesting that this response implies paraelectric behavior.

Supporting Note 2 | Characterization of ferroelectric smectic phase for nBOE (n = 4–6).
According to the XRD results, the *d*-spacing within the SmX_F regime is according to the molecular length of **nBOE** (n = 4–6), implying that this smectic phase may possess SmA order rather than SmC. However, in the antiparallel-rubbed cell (5 μm), the SmX_F phase showed a fine stripe along the rubbing direction (**Figure S6**). This result is unlike the typical SmA_F phase [S7] in which a uniform director alignment parallel to the rubbing direction is allowed. Let us now discuss the plausible structure of the SmX_F phase. Based on the X-ray diffraction pattern, this phase can be categorized as a smectic LC phase. Figure S6 shows the POM image of various phases for **5BOE** in an antiparallel-rubbed cell (gap: 2 μm). In the SmX_F phase, we found the extinction position (Figure S6e); however, the coexistence of the two domains was visualized upon the rotation of the stage by ±5° within either domain. By this manipulation, the birefringence colors (orange and blue) were inverted in the domains (Figure S6f and 6g). Thus, in the two domains, the molecules within a layer are oriented at a finite angle with different signs relative to the normal direction of the layer. An optical tilt angle (θ_{opt}) in the SmX_F phase was determined using a microscopic method (**Figure S7**), resulting in θ_{opt} ~2–3°. Thus, the combined data demonstrate that the third mesophase exhibited for **nBOE** (n = 4–6) is the SmX_F phase with a small tilt angle (≤ 3°).

Supporting Note 3 | Characterization of 7BOE and 8BOE.

The POM textures of N and N′ phases of **7BOE** and **8BOE** were similar to each other, both in the bare glass and non-rubbed PI cells (**Figure** S8, Supporting Information). The change in the birefringence color at the N–N′ phase transition was only subtle. In the rubbed PI cell, a blocky and blocky striped textures were observed in the N′ and SmA phases, in which birefringence vanishes at the extinction position (**Figure S9**a, Supporting Information). In a planar (annealed PMMA) cell, a typical Schlieren texture, a broken Schlieren texture, and a blocky texture were observed in the N, N′, and SmA phases, respectively (Figure S9b, Supporting Information). The DSC curves for **7BOE** and **8BOE** showed a small exothermal peak with ΔH = 12.9 and 14.2 J mol^{-1} at ~193 and ~175 °C, respectively, at the N–N′ phase transition, indicating the weak 1st order phase transition (**Figure S10**, Supporting Information). **7BOE** has an additional exothermal peak emerged at 134.8 °C (ΔH = 0.11 kJ mol^{-1}) owing to the N′–SmA phase transition. These two peaks are also observed upon heating, indicating that the three phases exhibit enantiotropic properties. Similarly, **8BOE** displayed two transition peaks at 175.2 °C and ~80 °C, but its SmA phase was metastable. Compared to the 1D X-ray diffractogram of N, N′ and SmA phases for **7BOE**, that for **8BOE** showed a small angle peak becoming shaper and more intense with decreasing temperature. The fact indicates the emergence of long-range order and increase in the correlation length in the N′ and SmA phases. DR and *P–E* studies didn't exhibit any ferroelectric signals in the N, N′, and SmA phases (**Figure S11**, Supporting Information). Thus, these phases are considered to be just paraelectric. Summarizing the data above, we concluded that the N′ phase is assigned as a cybotactic nematic phase of a short-range ordered structure with the smectic A-type correlation.

Supporting Note 4 | FTIR spectra measurement.

Figure S12 shows the computational and experimental FTIR spectra of **5BOE**. Figure S12a shows a discrepancy between the peak position (ν_{FTIR} or ν_{DFT}) on the characteristic spectra, attributed to the computational results considering the gas phase. Nevertheless, no significant differences in the peak intensities or relative peak positions were observed. Thus, the respective vibration modes were characterized by calibrating the peak shifts. For example, the peak at ν_{FTIR} = 1630 cm^{-1} (ν_{DFT} = 1670 cm^{-1}) was identified predominantly as a C–C stretching vibration of the second aromatic ring (Ar^{II}).

Supporting Note 5 | Control materials: nBOE-F and nBOE-NO_2.

Figure S13a displays the phase transition behavior for **4BOE**, **4BOE-F** and **4BOE-NO_2**. All compounds exhibited the enantiotropic N_F phase with a broaden temperature window. As shown in Figure S13a and S13b, three types of compounds showed the similar trend with respect to dipole moment and β angle as a function of n, and almost same corresponding magnitude (see also Table S1). Nevertheless, the ${}^{HC}N_F$ phase was absent in **4BOE-F** and **4BOE-NO_2**, indicating that an interaction with a –CN group contributes emerging the ${}^{HC}N_F$ phase.

Supporting Figures (Figures S1–S31)

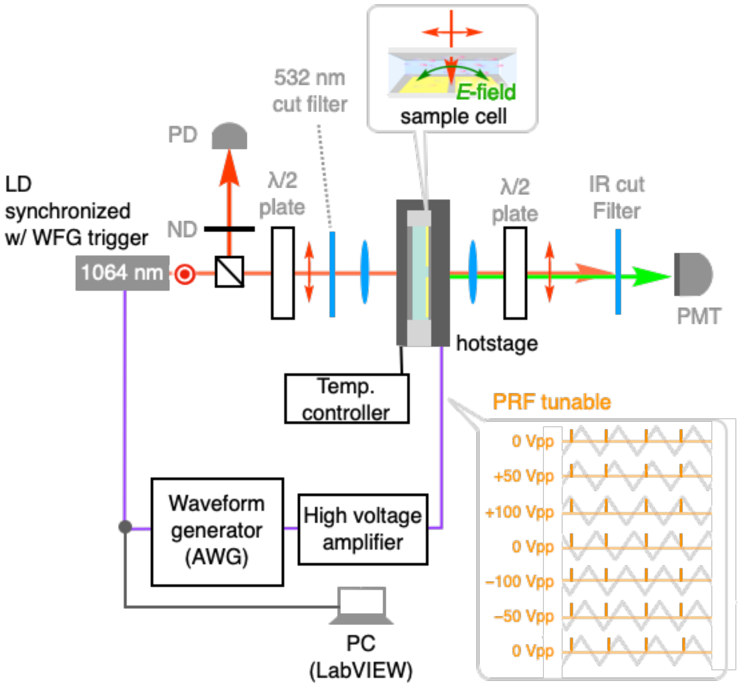

Figure S1 Optical setup for SHG studies. The electric field (*E*-field) was applied normally to the sample cell (thickness: 5 μm).

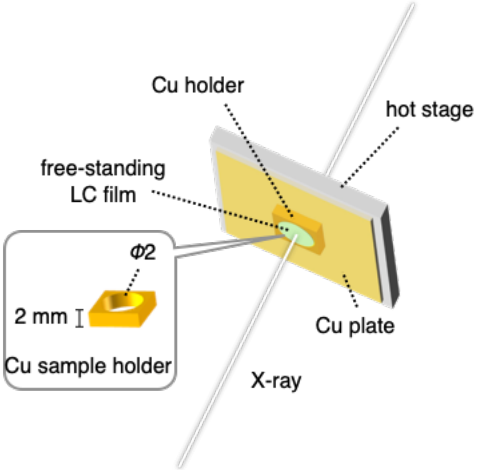

Figure S2 Measurement setup for XRD studies.

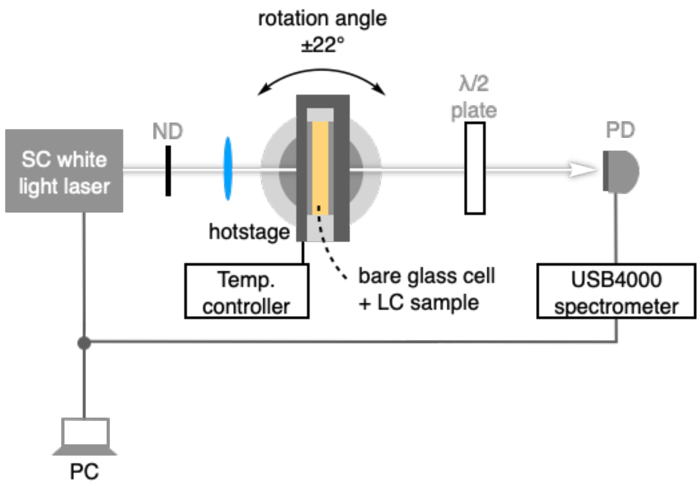

Figure S3 Optical setup for Spectra studies. Cell thickness: 5 μm.

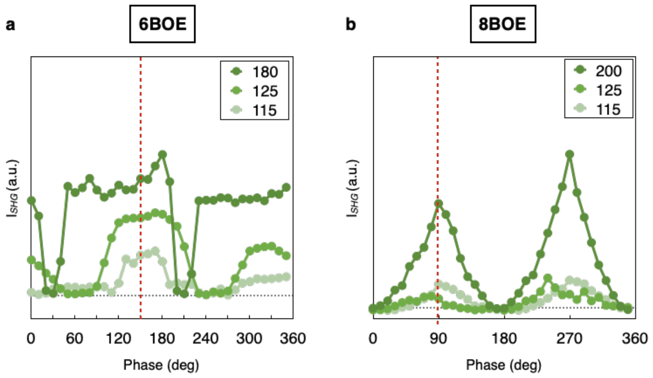

Figure S4 SHG Intensity vs phase (related to the magnitude of *E*-field) in various temperature for **6BOE** (a) and **8BOE** (b).

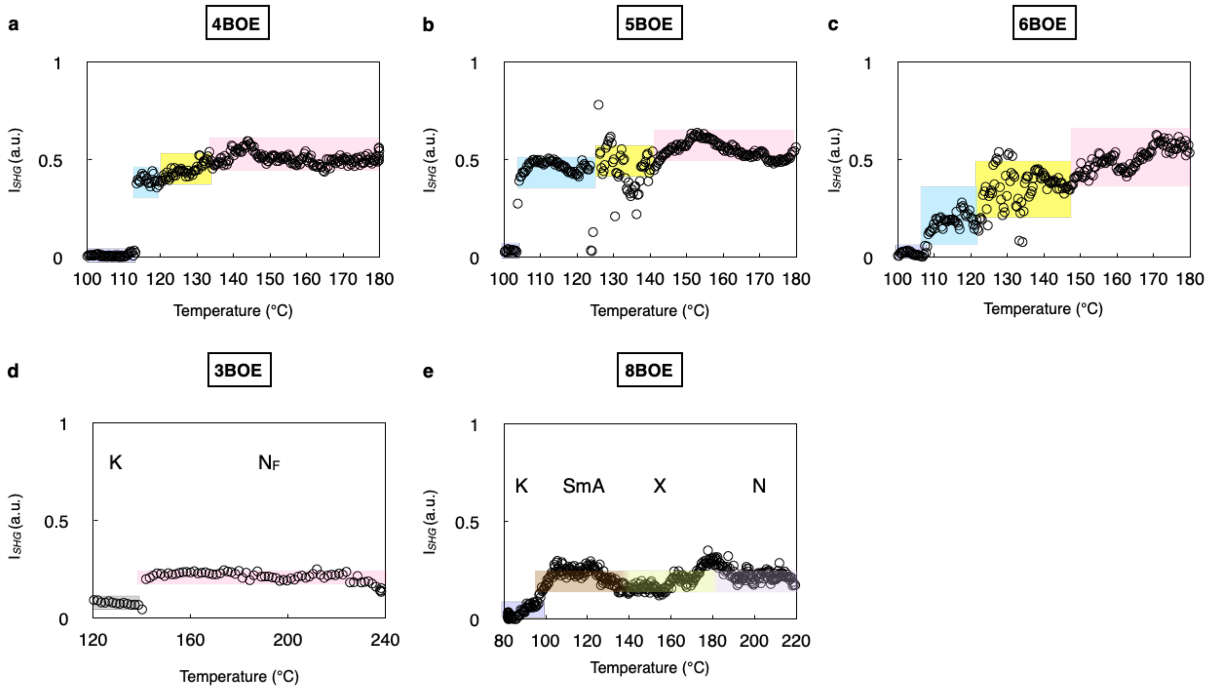

Figure S5 SHG vs temperature for **nBOE** (n = 3–6, 8).

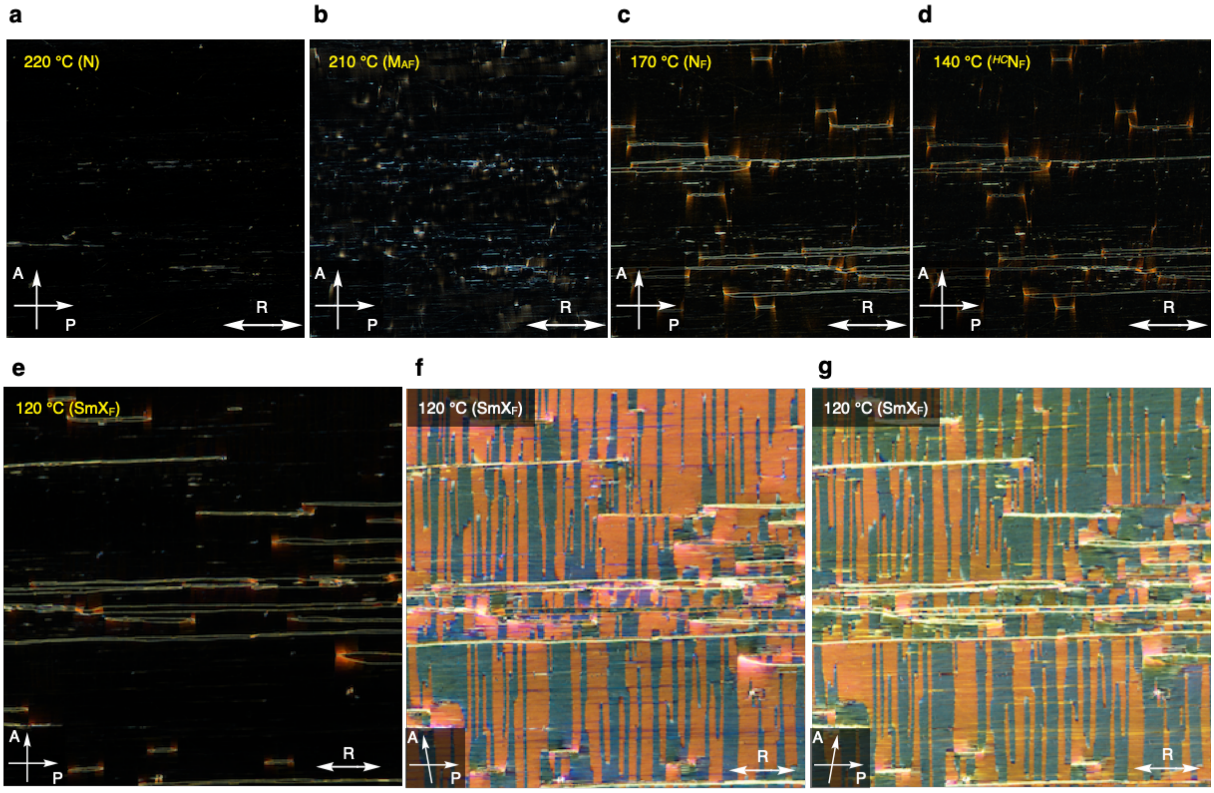

Figure S6 POM images of the N (a), M_{AF} (b), N_F (c), $^{HC}N_F$ (d) and SmX_F (e) phases for **5BOE** in the anti-parallel rubbed cell (2 μm). The texture changes upon the rotation of an analyzer by + 5° (f) and −5° (g).

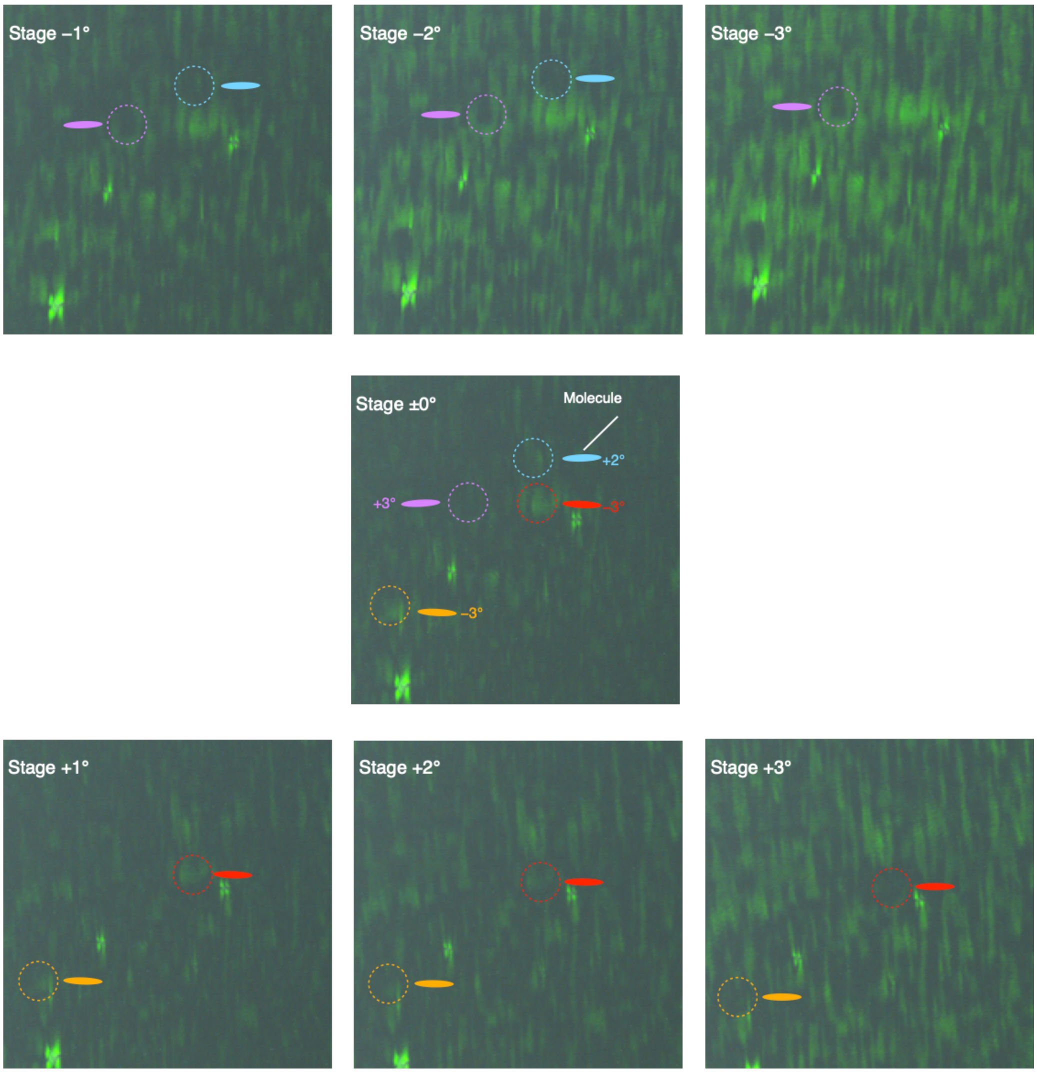

Figure S7 Determination of an optical tilt angle in the SmX_F phase (110 °C, 2 μm thicker rubbed cell) using a microscopic method (green band path filter: 532 nm).

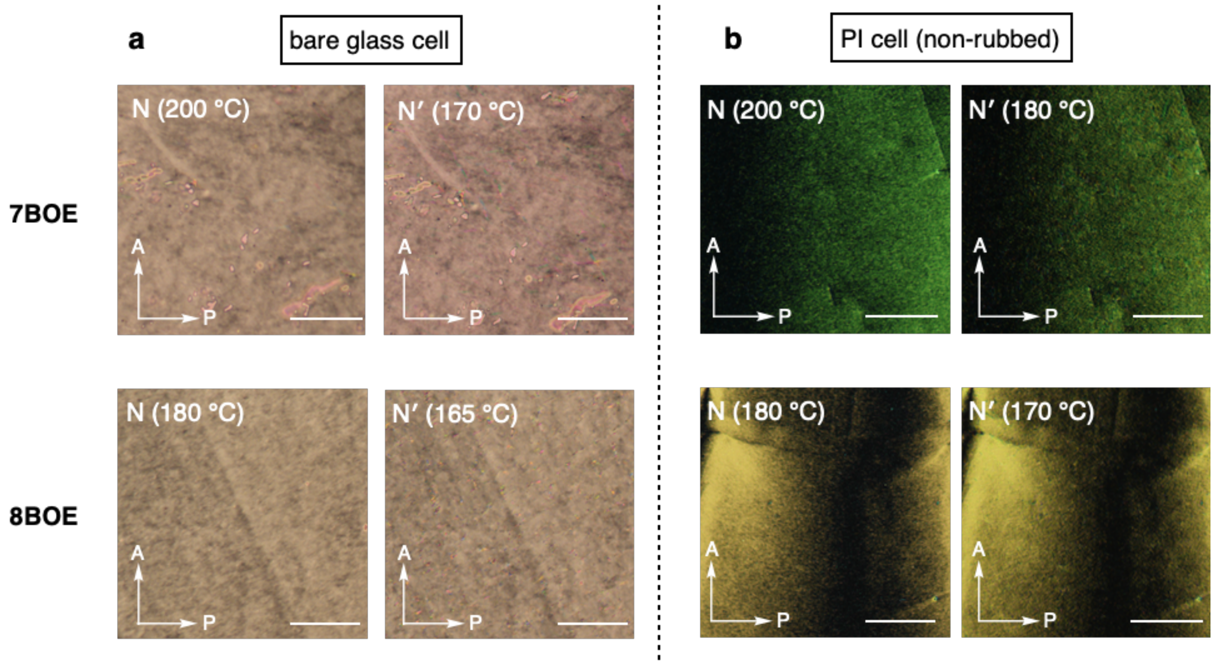

Figure S8 Extra POM images for **7BOE** (a) and **8BOE** (b) in the bare glass (upper) and non-rubbed polyimide (bottom) cells. Scale bar: 100 μm.

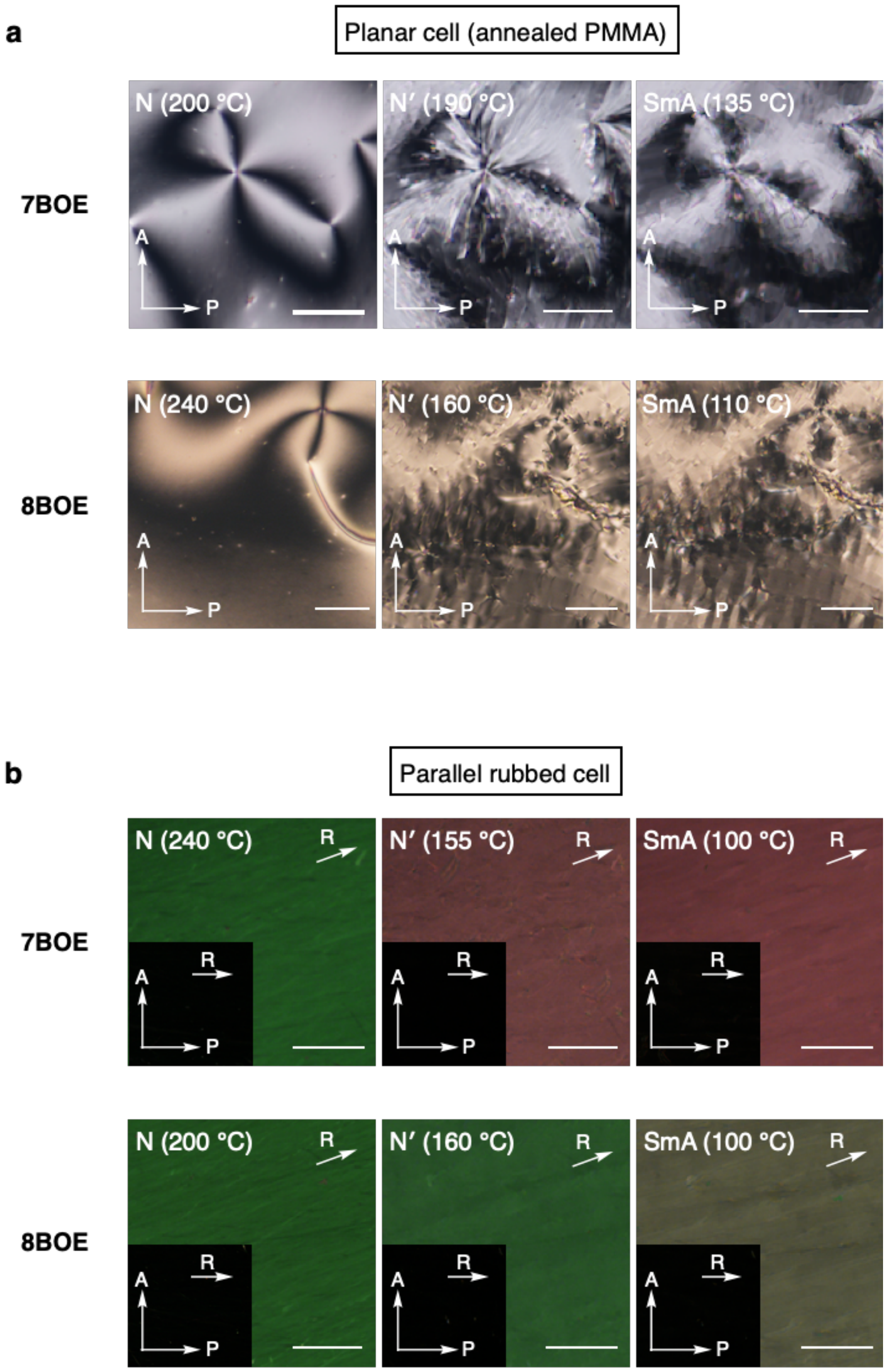

Figure S9 Extra POM images for **7BOE** and **8BOE** in the planar (upper) and antiparallel rubbed (bottom) cells. Scale bar: 100 μm.

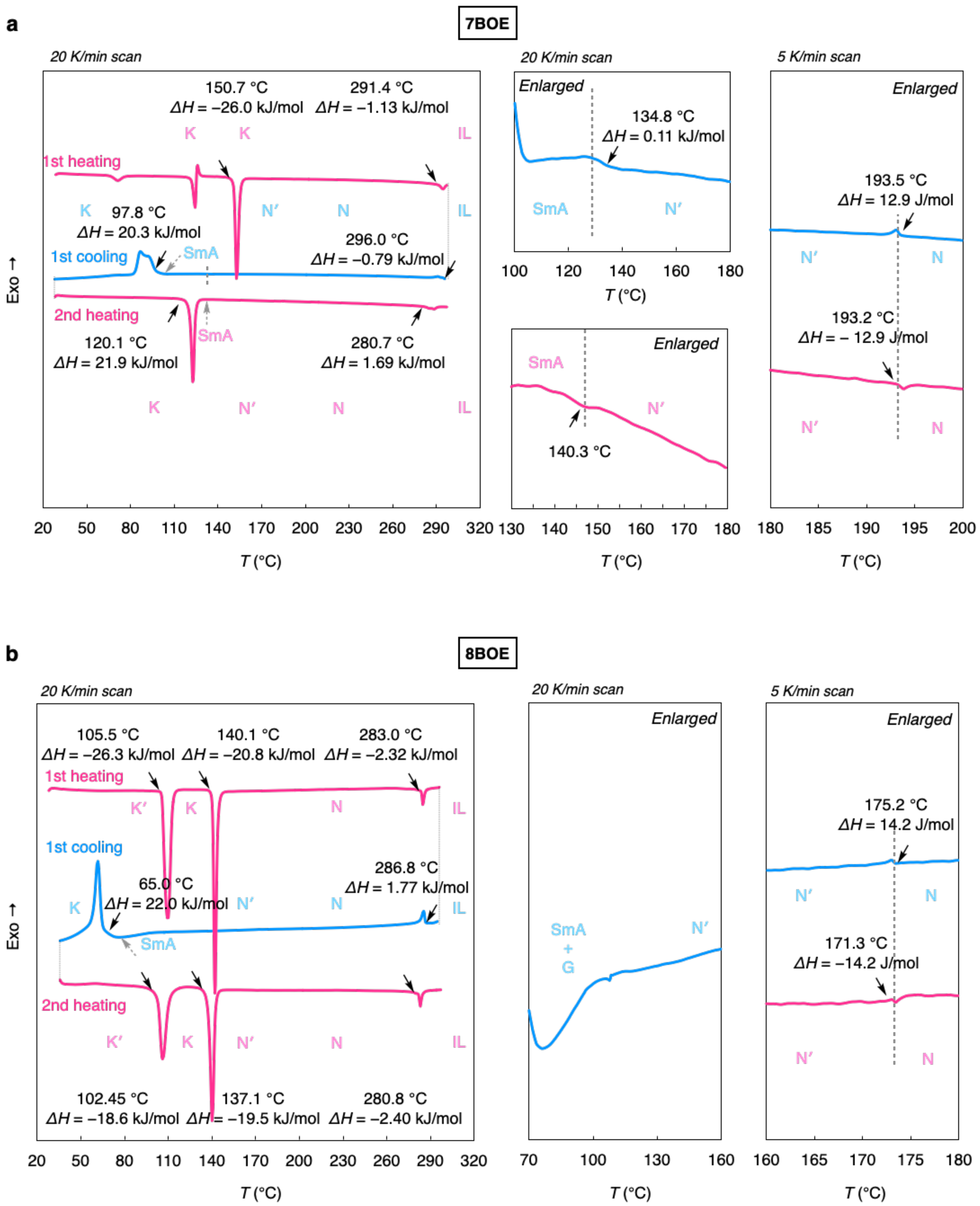

Figure S10 DSC curves for **7BOE** (a) and **8BOE** (b). Scan rate 5 and 20 K min^{-1}.

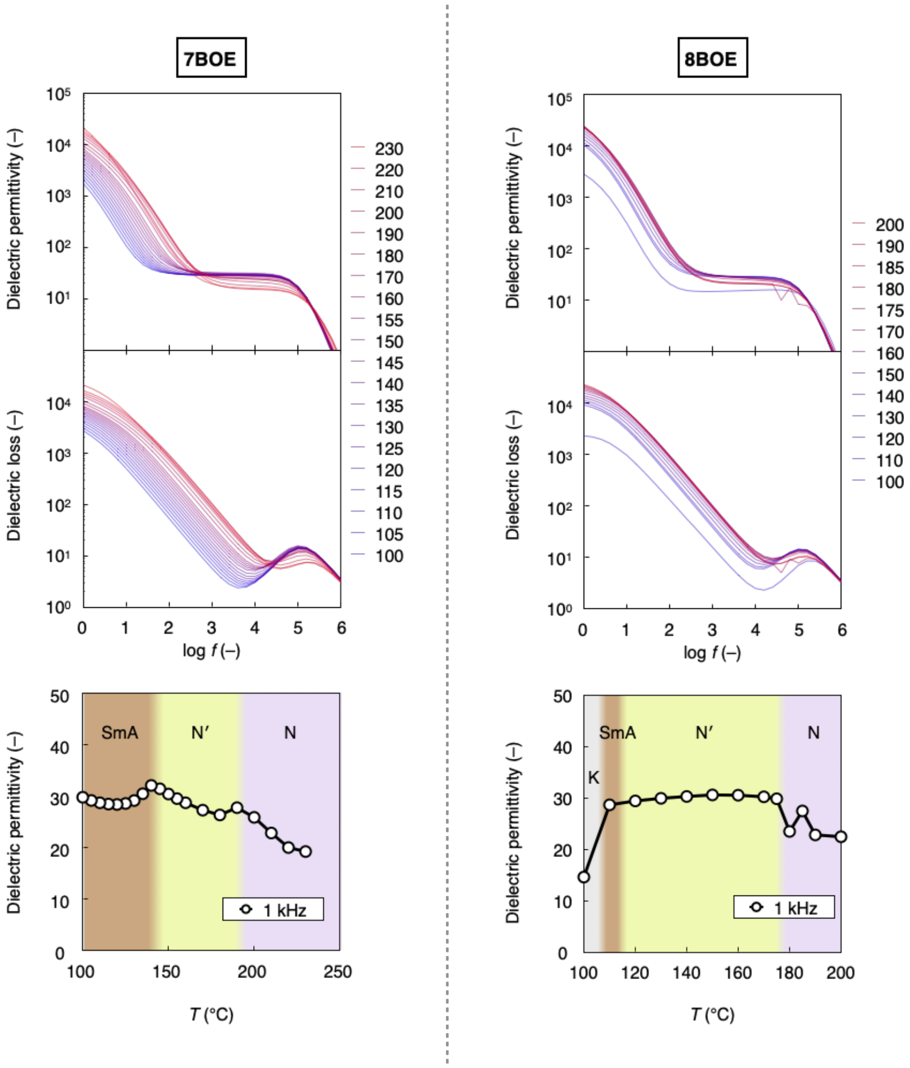

Figure S11 Extra DR spectra for **7BOE** and **8BOE**.

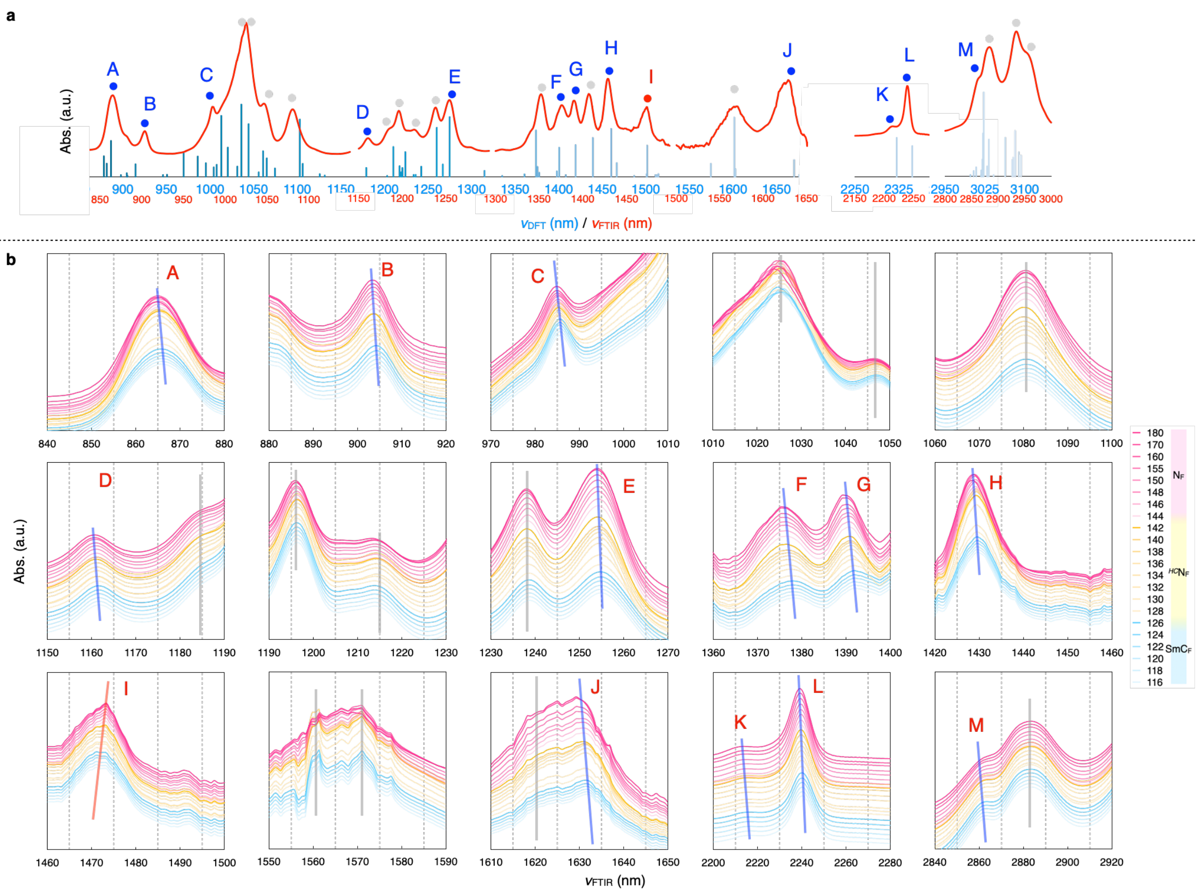

Figure S12 (a) Computational and Experimental FTIR spectra for **5BOE**. (b) Highlighted FTIR spectra during N_F–$^{HC}N_F$–SmX_F phase transition. The change in peak position is represented by colored straight lines: magenta (low v), blue (high v) and gray (no change).

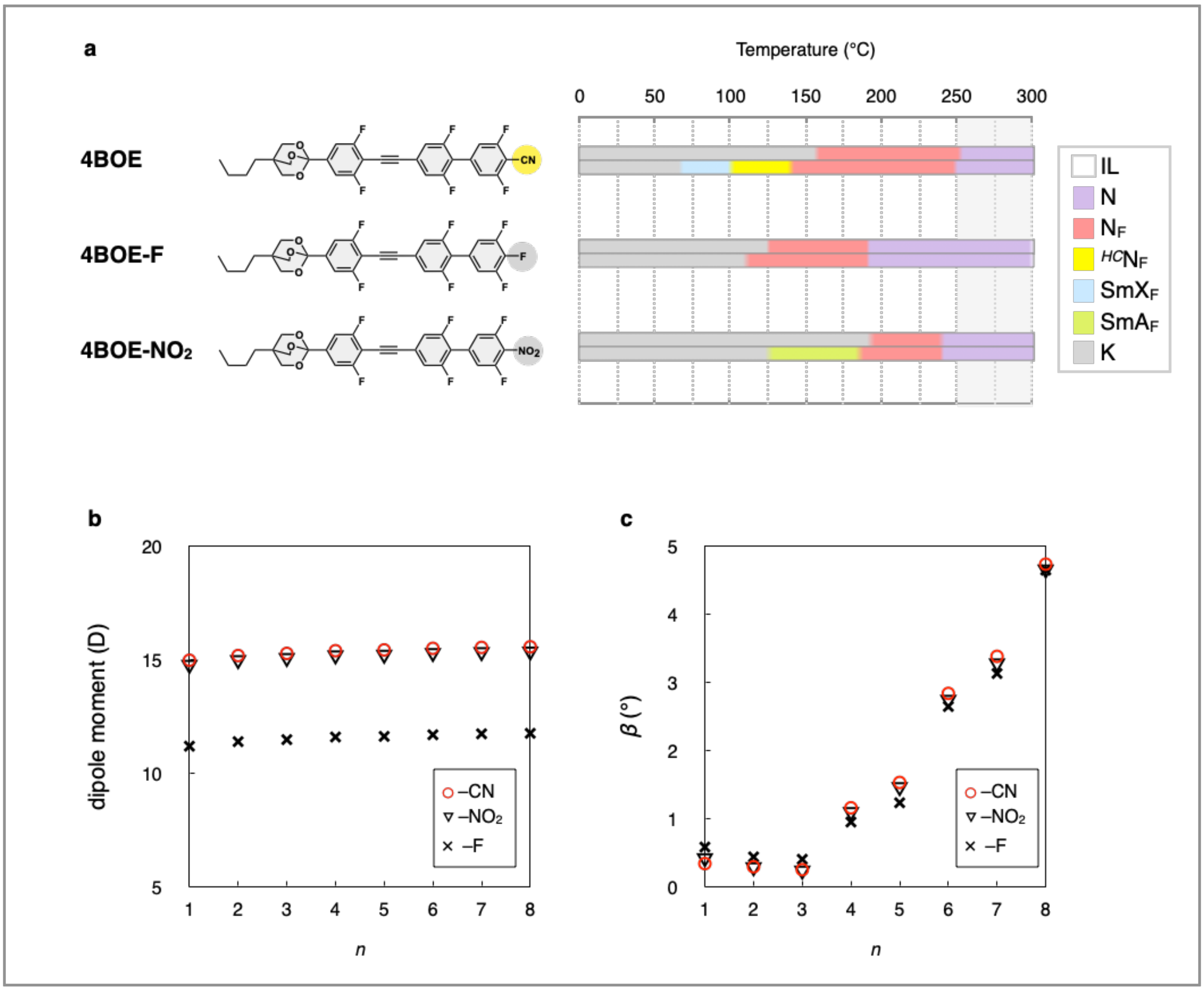

Figure S13 Comparison of phase transition behavior (a), dipole moment (b) and β angle (c) vs carbon number of alkyl chain (n) for **4BOE**, **4BOE-F** and **4BOE-NO$_2$**.

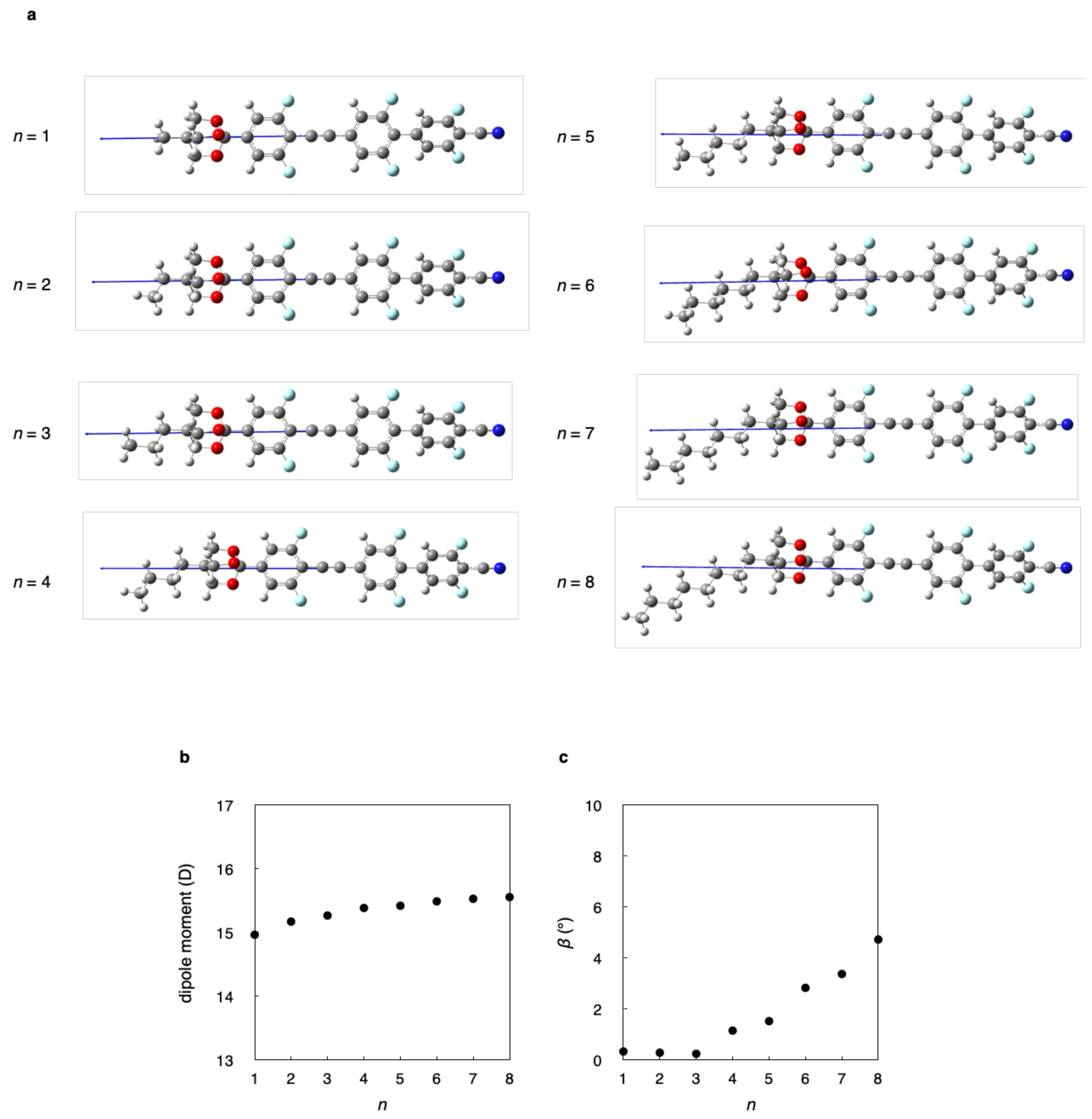

Figure S14 (a) Optimized structures of **nBOE** (n = 1–8). Dipole moment (a) and β angle (b) vs carbon number of alkyl chain (n).

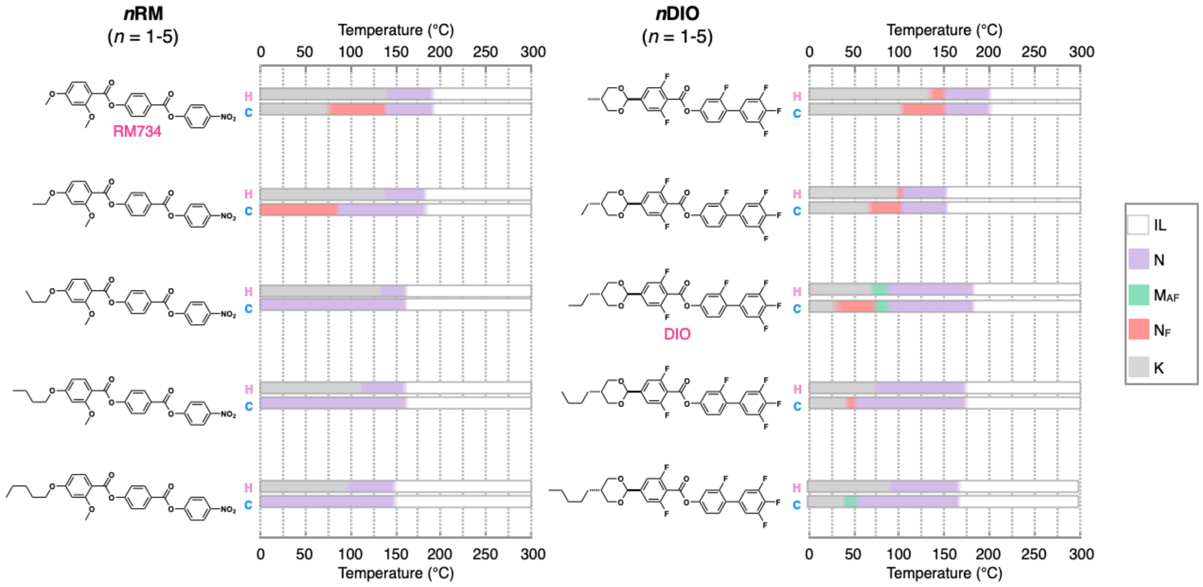

Figure S15 Phase transition behavior for **nRM** (n = 1–5) and **nDIO** (n = 1–5). Data of **nRM** (n = 1–5) were extracted from ref. S8. For data of **nDIO** (n = 2,3,4) were extracted from ref. S9, S10 and S11, respectively. The phase transition temperature for **nDIO** (n = 1,5), which were synthesized in our laboratory, was decided by DSC studies.

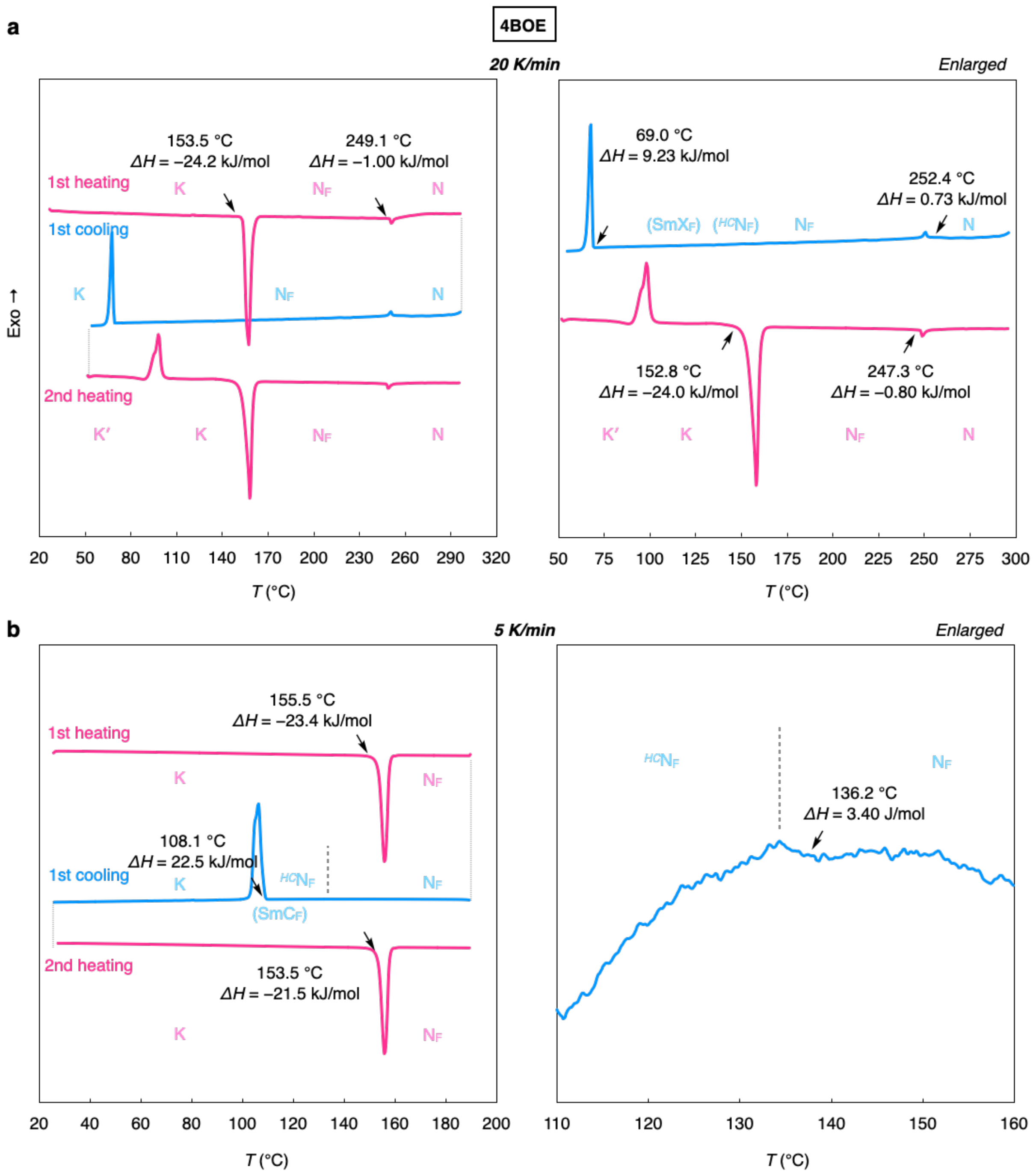

Figure S16 DSC curves for **4BOE**. Scan rate 20 K min^{-1} (a) and 5 K min^{-1} (b).

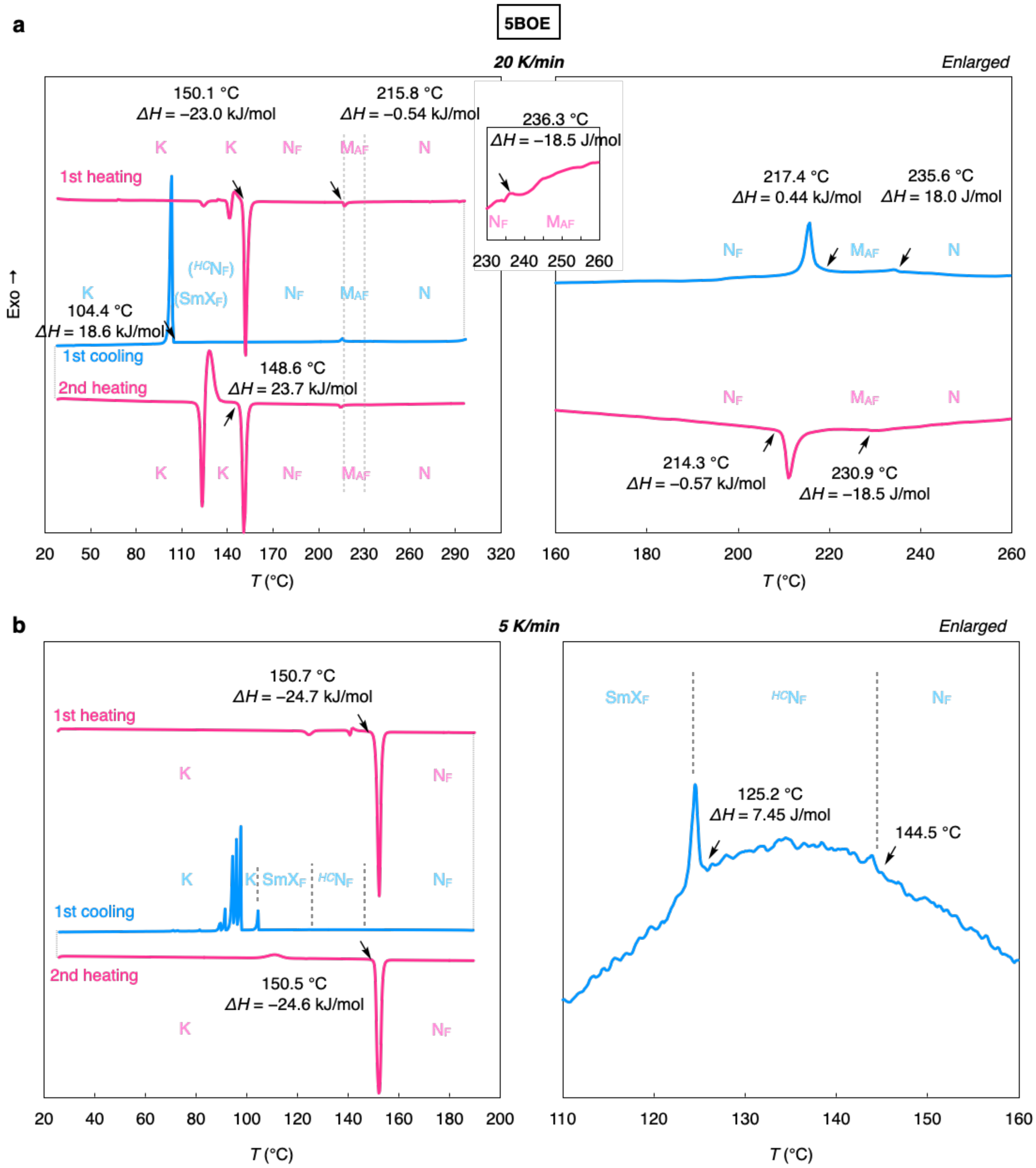

Figure S17 DSC curves for **5BOE**. Scan rate 20 K min^{-1} (a) and 5 K min^{-1} (b).

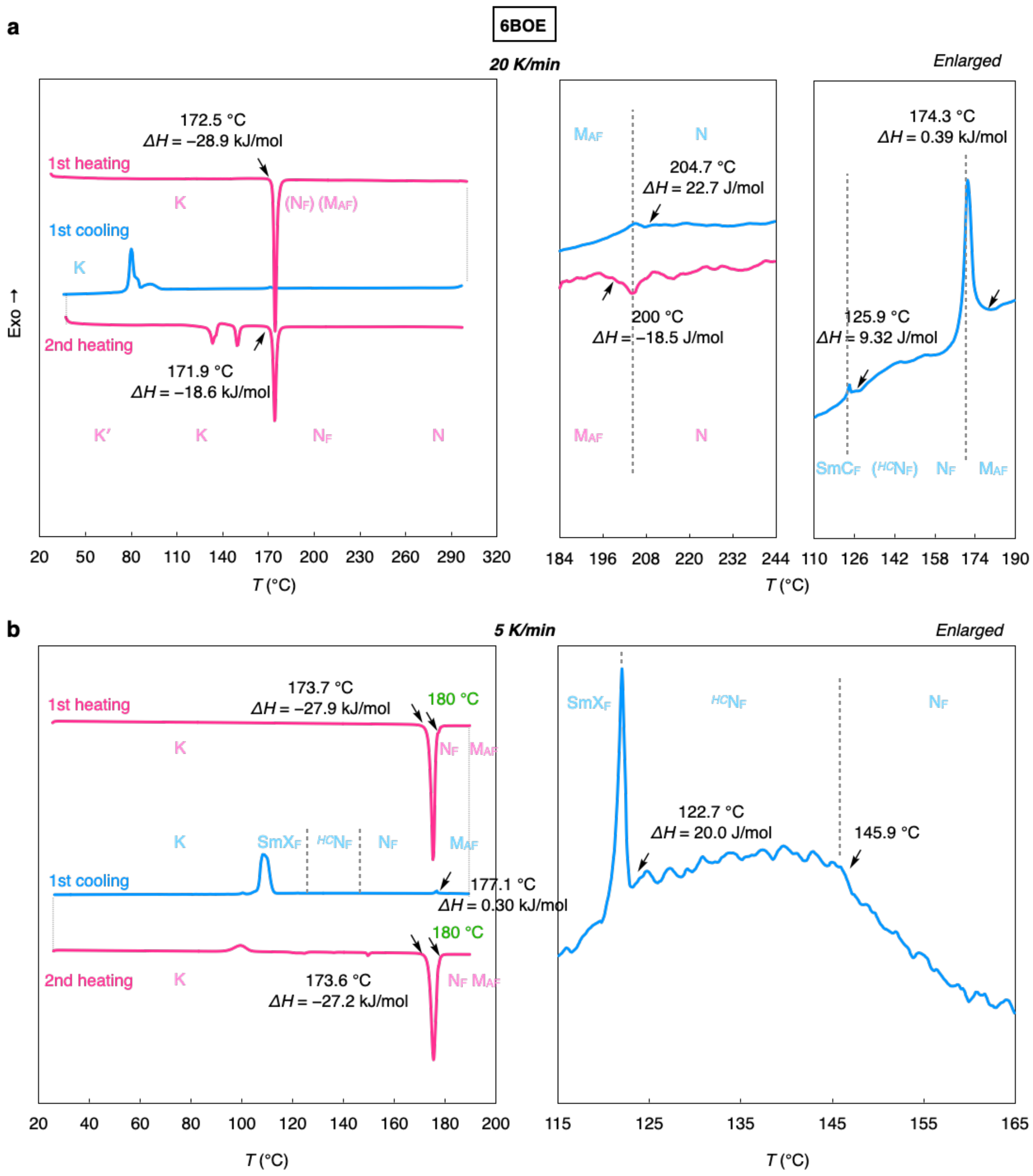

Figure S18 DSC curves for **6BOE**. Scan rate 20 K min^{-1} (a) and 5 K min^{-1} (b).

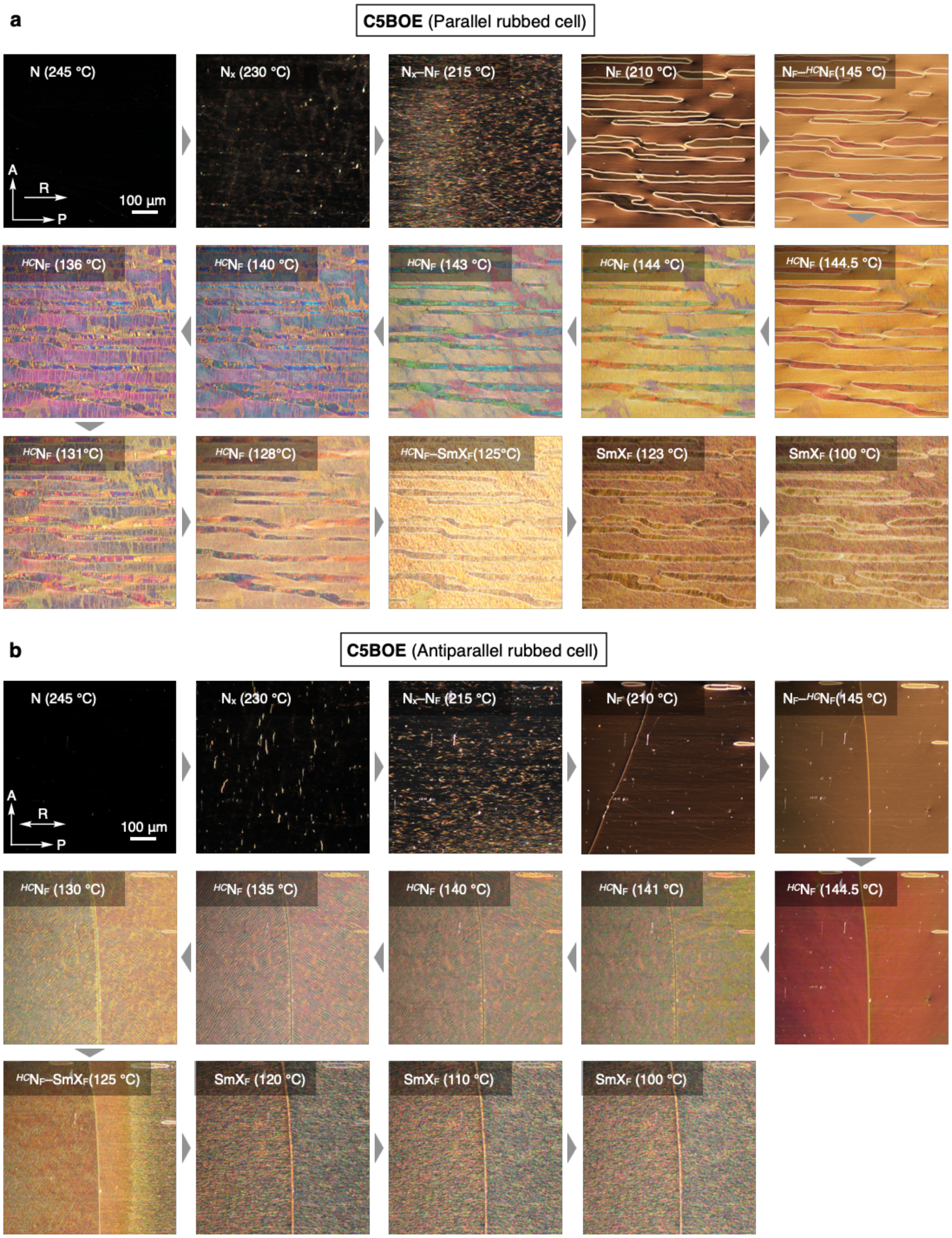

Figure S19 Extra POM images for **5BOE** in the parallel- (a) and antiparallel- (b) cells. Scale bar: 100 μm.

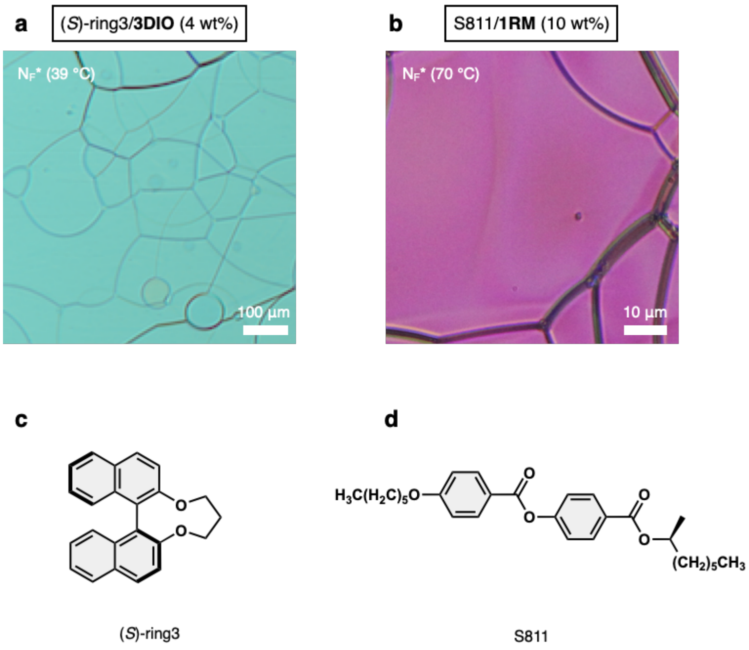

Figure S20 POM images of the ferroelectric cholesteric (N_F*) phase in two cases: (a) (*S*)-ring/**3DIO** (4 wt%), (b) S811/**1RM** (10 wt%). Chemical structures of the corresponding chiral dopant are shown in the panel (c) and (d).

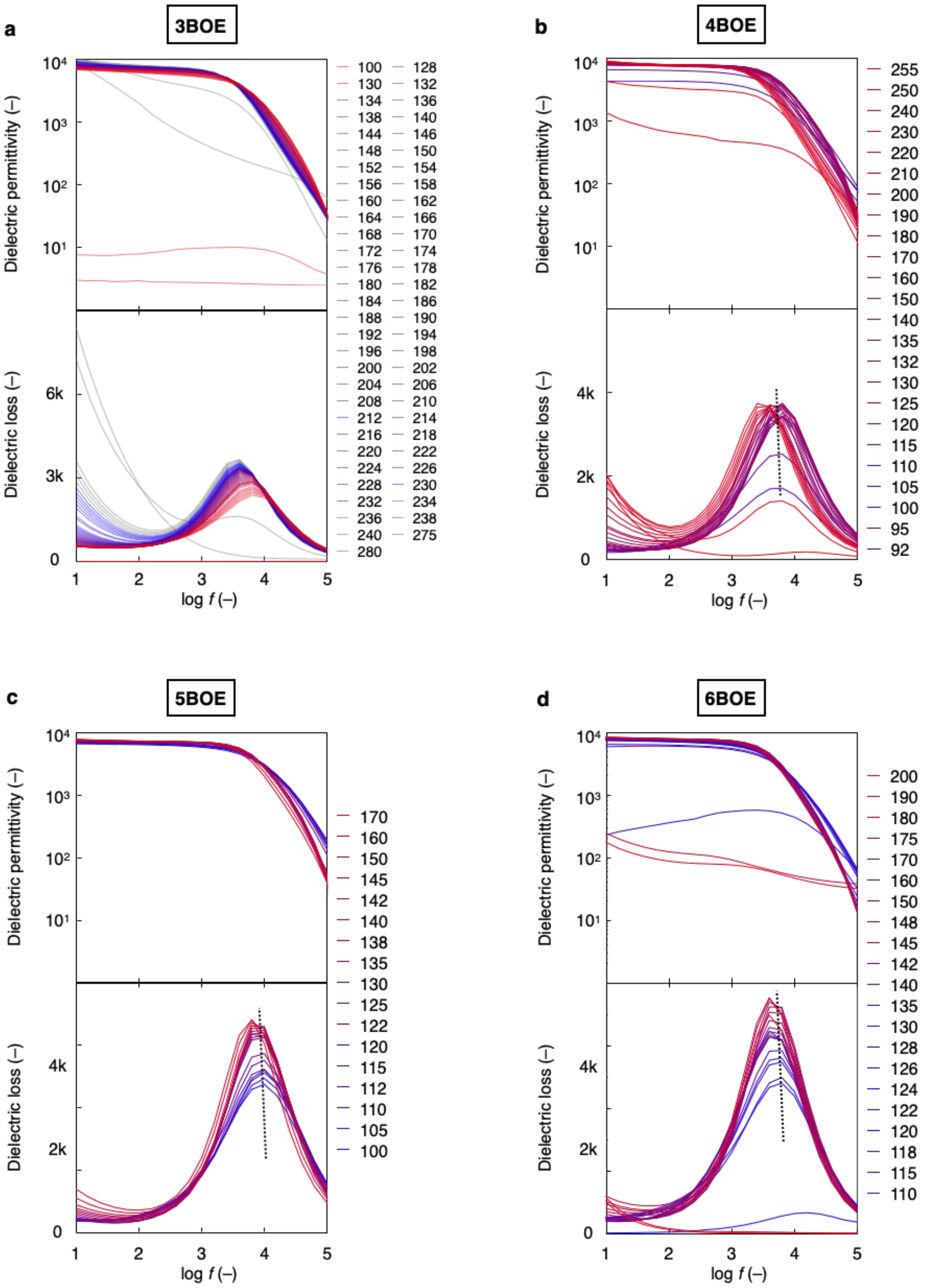

Figure S21 Extra DR spectra for **nBOE** (n = 3–6).

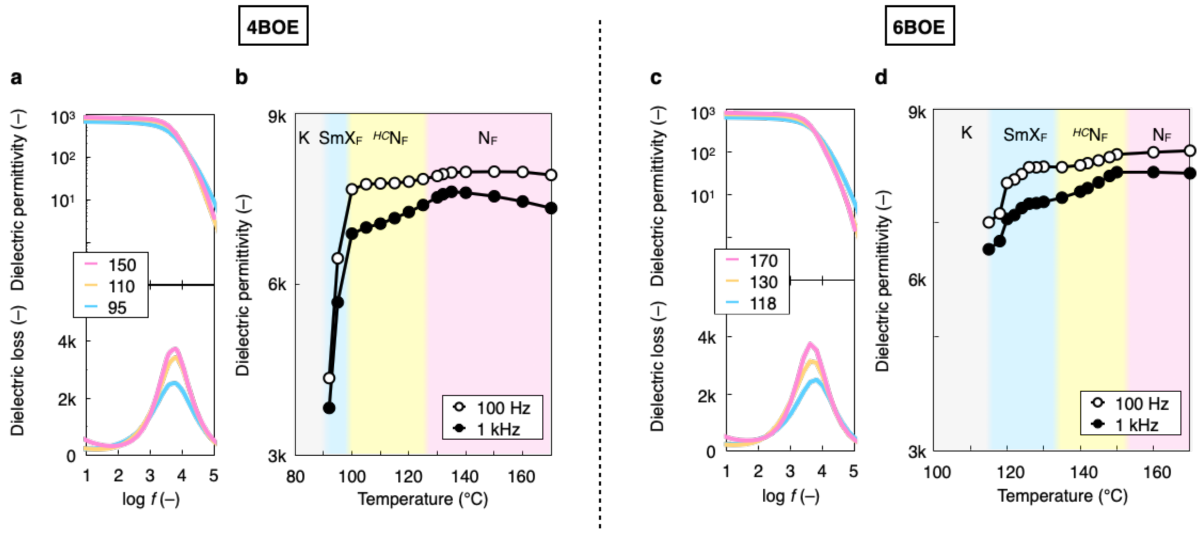

Figure S22 DR spectra and temperature-dependent dielectric permittivity for **4BOE** and **6BOE**.

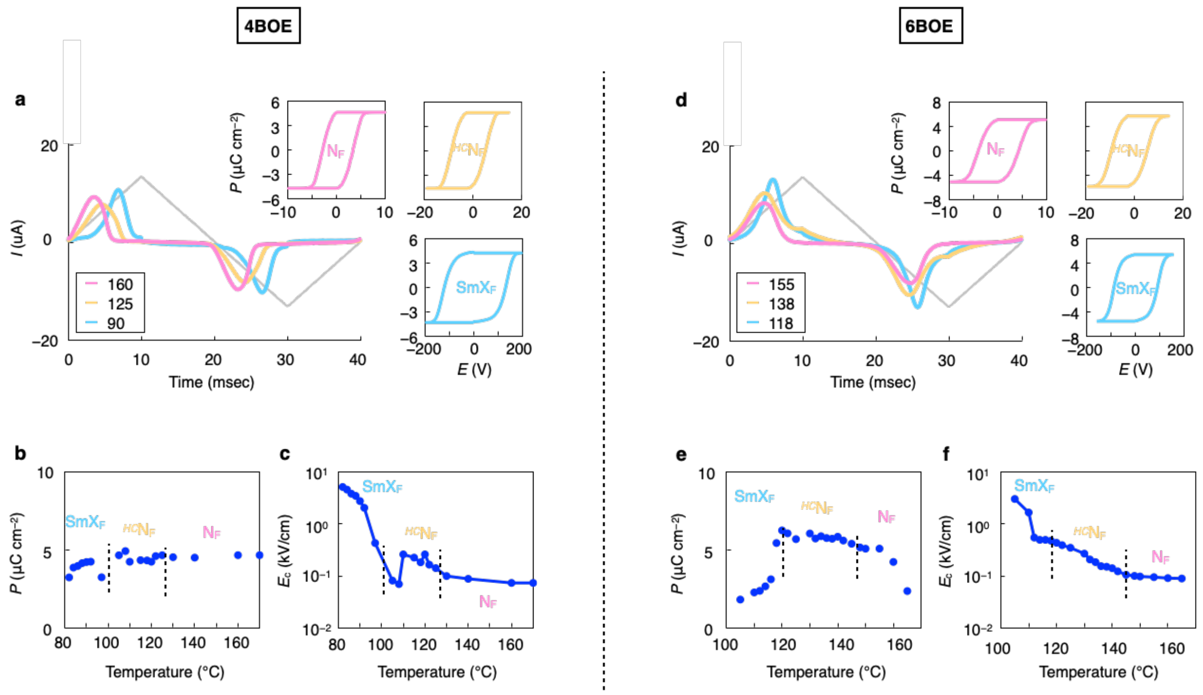

Figure S23 Polarization behavior for **4BOE** and **6BOE**. (a,d) *I* vs time. Insets represent the corresponding *P*-*E* hysteresis loop. (b,e) *P* vs temperature. (c,f) E_c vs temperature.

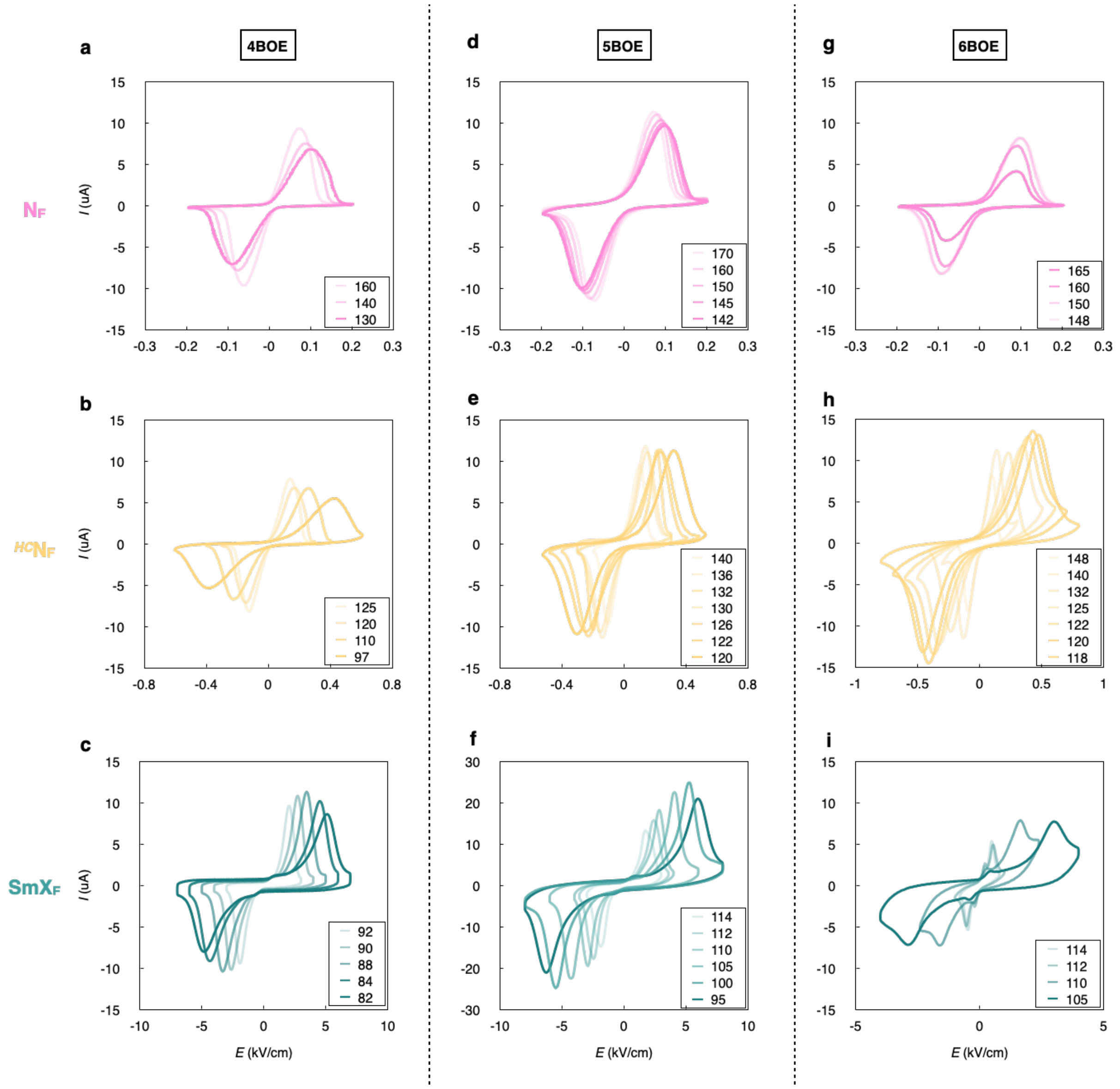

Figure S24 Extra polarization reversal current data for **nBOE** (n = 4–6).

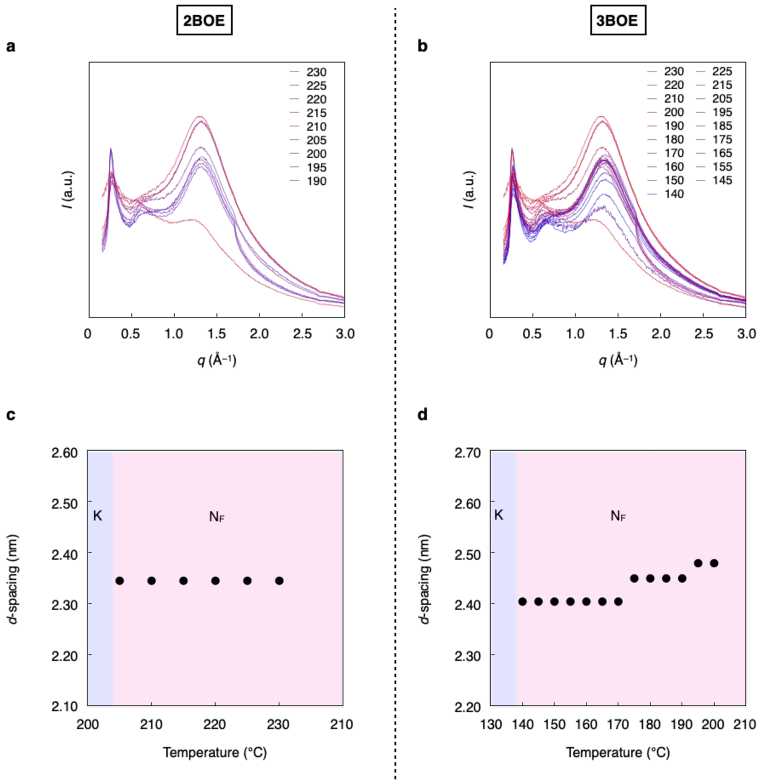

Figure S25 Temperature dependent 1D XRD pattern and *d*-spacing for **2BOE** (a,c) and **3BOE** (b,d).

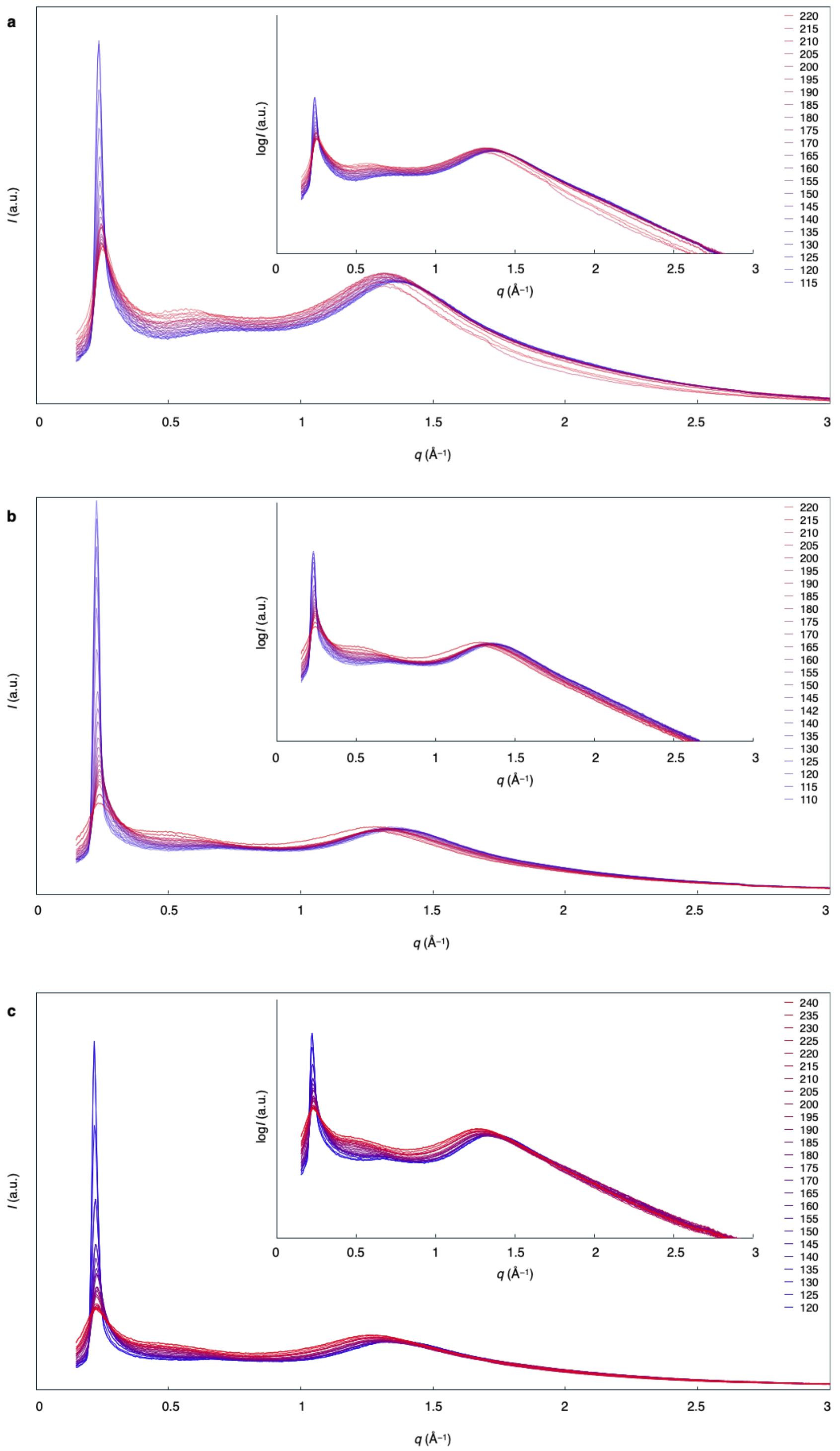

Figure S26 Temperature-dependent 1D XRD pattern for **4BOE** (a), **5BOE** (b) and **6BOE** (c).

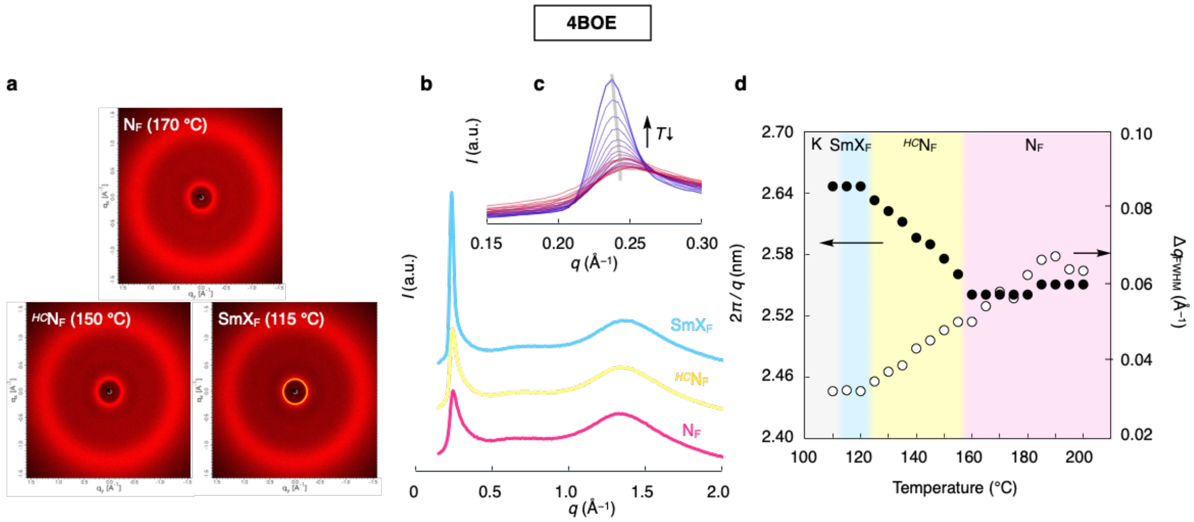

Figure S27 XRD studies for **4BOE**. 2D XRD (a) and 1D XRD (b,c) pattern in various phases. (d) *d*-spacing and FWHM vs Temperature. For the panel (b), the recorded temperature in N_F, $^{HC}N_F$ and SmX_F phases are 170, 150 and 115 °C, respectively.

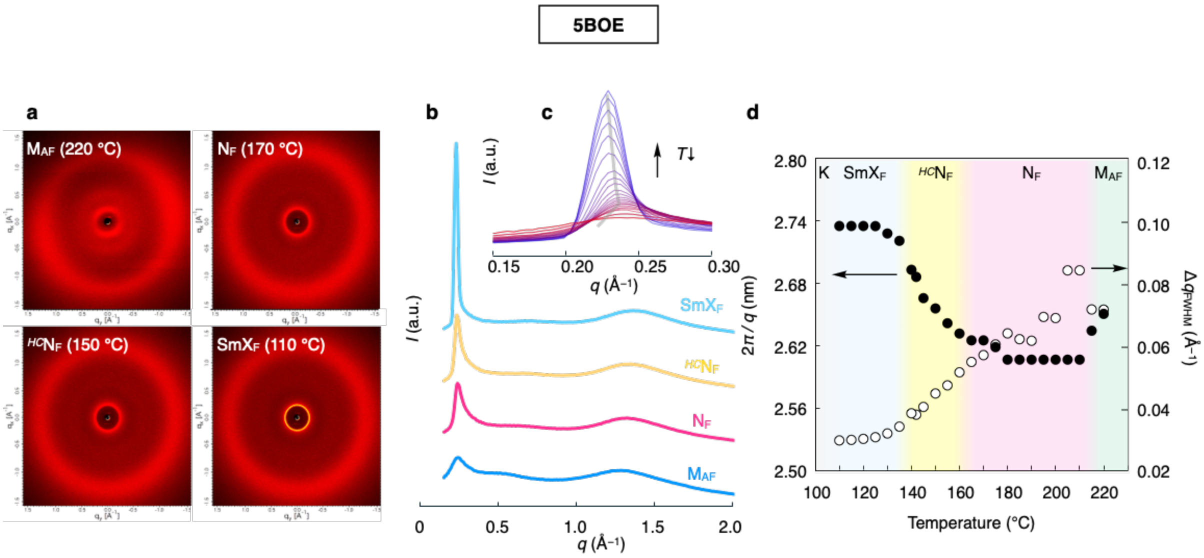

Figure S28 XRD studies for **5BOE**. 2D XRD (a) and 1D XRD (b,c) pattern in various phases. (d) *d*-spacing and FWHM vs Temperature. For the panel (b), the recorded temperature in M_{AF}, N_F, ${}^{HC}N_F$ and SmX_F phases are 220, 170, 150 and 110 °C, respectively.

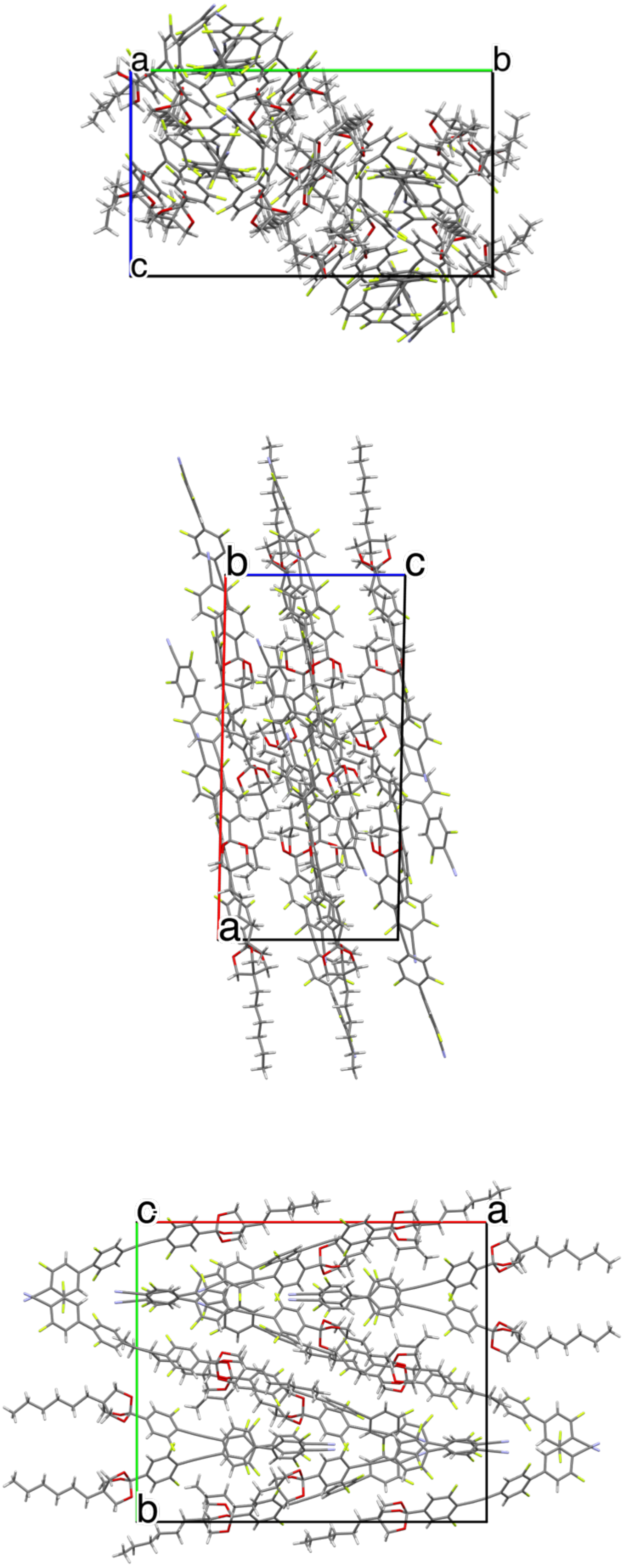

Figure S29 SC-XRD data viewed along the a-, b-, and c-axis for **6BOE**: crystal twinning, crystal system: monoclinic, space group: $P2_{1/c}$, cell length: **a** = 29.5406(2) Å, **b** = 25.3902(2) Å, **c** = 14.49345(13) Å, cell angle: **α** = 90°, **β** = 91.1515(7)°, **γ** = 90°, cell volume: **V** = 10868.5 Å^3, **Z** = 16, **Z′** = 4, R-factor = 7.71%.

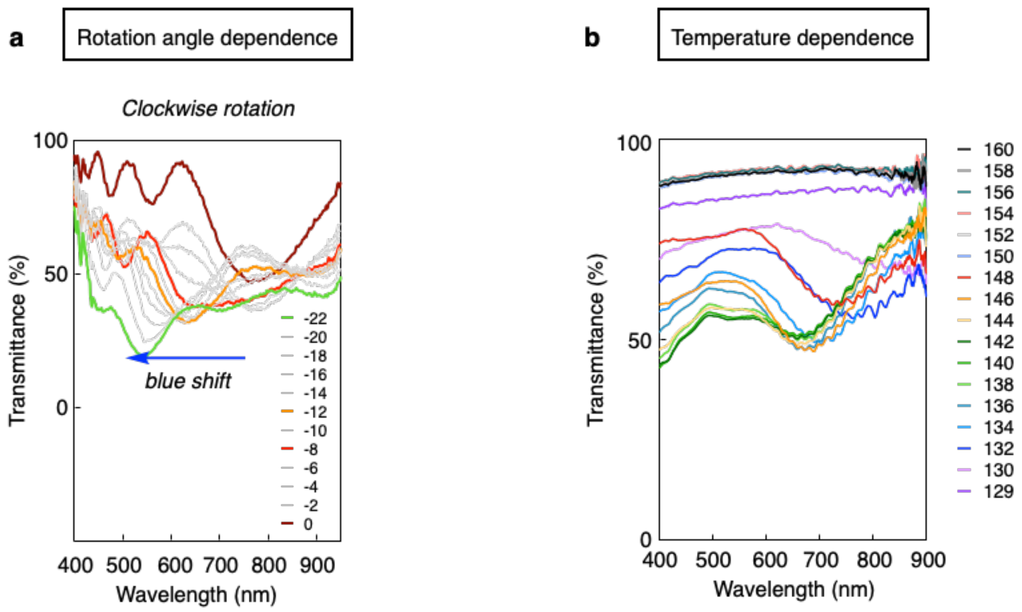

Figure S30 Extra spectra data for 5BOE. (a) Rotation angle dependence ($^{HC}N_F$ phase at 145 °C), (b) temperature dependence (cooling rate: 1 K min^{-1}).

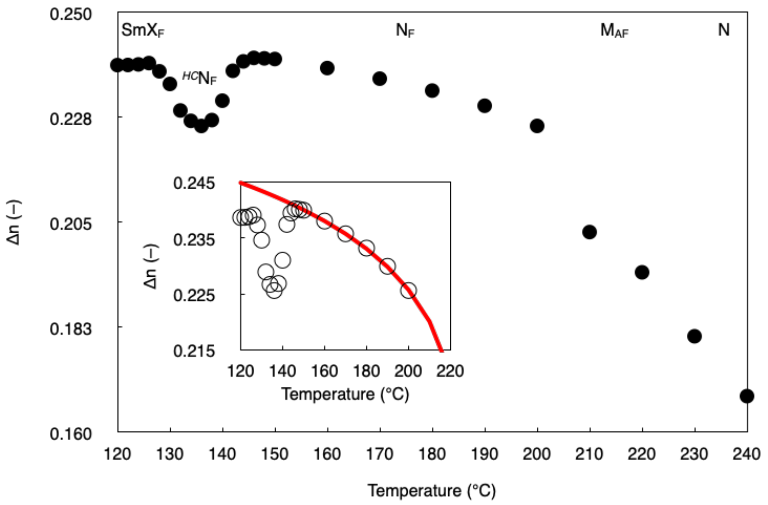

Figure S31 Temperature dependence of 5BOE. Inset: fitting curve calculated within N_F regime. The $\Delta n(N_F)$ was extrapolated to the lower temperature range by supposing a power-law temperature dependence: $\Delta n(N_F)= \Delta n_0(T_c-T)^{\gamma}$, where $\Delta n0$, T_c, and γ are the fitting parameters.

Supporting Tables (Tables S1 and S2)

Table S1 The molecular parameters of energy-minimized conformations calculated by MM2/DFT for **BOE** variants.

Entry	Vector X	Vector Y	Vector Z	μ (D)	β (deg)†
**1BOE**	14.967	0.037	-0.080	14.967	0.339
**2BOE**	15.173	0.061	-0.048	15.173	0.293
**3BOE**	15.269	0.062	-0.023	15.269	0.249
**4BOE**	-15.385	-0.254	0.179	15.388	1.158
**5BOE**	-15.417	-0.320	0.259	15.423	1.529
**6BOE**	-15.482	-0.642	0.420	15.491	2.836
**7BOE**	-15.472	-0.808	0.427	15.532	3.379
**8BOE**	-15.505	-1.155	0.560	15.558	4.732
**1BOE-NO_2**	14.678	0.010	-0.099	14.678	0.389
**2BOE-NO_2**	14.885	0.028	-0.061	14.885	0.257
**3BOE-NO_2**	14.978	0.036	-0.041	14.978	0.207
**4BOE-NO_2**	15.091	0.266	0.090	15.093	1.066
**5BOE-NO_2**	15.116	0.343	0.158	15.121	1.432
**6BOE-NO_2**	-15.191	-0.665	0.275	15.197	2.713
**7BOE-NO_2**	-15.180	-0.807	0.301	15.238	3.247
**8BOE-NO_2**	-15.213	-1.179	0.352	15.263	4.624
**1BOE-F**	11.184	0.011	-0.113	11.184	0.582
**2BOE-F**	11.385	0.004	-0.087	11.385	0.437
**3BOE-F**	11.473	-0.003	-0.081	11.473	0.402
**4BOE-F**	-11.587	-0.190	0.027	11.589	0.949
**5BOE-F**	-11.613	-0.239	0.073	11.615	1.232
**6BOE-F**	-11.683	-0.506	0.186	11.687	2.644
**7BOE-F**	-11.675	-0.605	0.204	11.729	3.128
**8BOE-F**	-11.712	-0.915	0.262	11.751	4.645

† an angle between the permanent dipole moment (μ) and long molecular axis. $\beta = \sin^{-1}\left(\frac{\sqrt{Y^2+Z^2}}{\sqrt{X^2+Y^2+Z^2}}\right)$.

Table S2 The polarization density and estimated polar order parameter <P1> for **nBOE**.

Entry	P_s (D)	<P1>††
**1BOE**	4.6†	0.609
**2BOE**	5.8†	0.778
**3BOE**	6.5	0.890
**4BOE**	4.7	0.649
**5BOE**	6.1	0.869
**6BOE**	5.2	0.756

† extracted from S2, †† density (ρ) was set to be 1.3 g cm^{-3}

Supporting References

S1 M. J. Frisch, G. W. Trucks, H. B. Schlegel, G. E. Scuseria, M. A. Robb, J. R. Cheeseman, G. Scalmani, V. Barone, B. Mennucci, G. A. Petersson, H. Nakatsuji, M. Caricato, X. Li, H. P. Hratchian, A. F. Izmaylov, J. Bloino, G. Zheng, J. L. Sonnenberg, M. Hada et al. Gaussian 09, revision E.01; Gaussian, Inc.: Wallingford, CT, 2009.

S2 CrysAlisPro Software system version 1.171.43.90: Rigaku Oxford Diffraction; Rigaku Corporation, Wroclaw: Poland, 2023.

S3 G. M. Sheldrick, *Acta Crystallogr. Sect. A* **2015**, 71, 3–8.

S4 G. M. Sheldrick, *Acta Crystallogr. Sect. C* **2015**, 71, 3–8.

S5 H. Nishikawa et al., *J. Mater. Chem. C.* **2023**, 11, 12525–12542.

S6 H. Nishikawa et al., *Liq. Cryst.* **2024**, https://doi.org/10.1080/02678292.2024.2319629.

S7 H. Kikuchi et al., *Adv. Sci.* **2022**, 9, 2202048.

S8 R. Mandle et al., *Chem. Eur. J.* **2017**, 23. 14554.

S9 H. Nishikawa, *Doctoral thesis*, **2018**, pp.236.

S10 H. Nishikawa et al., *Adv. Mater.* **2017**, 29, 1702354.

S11 H. Nishikawa et al., *Commun. Mater.*, **2022**, 3, 89.

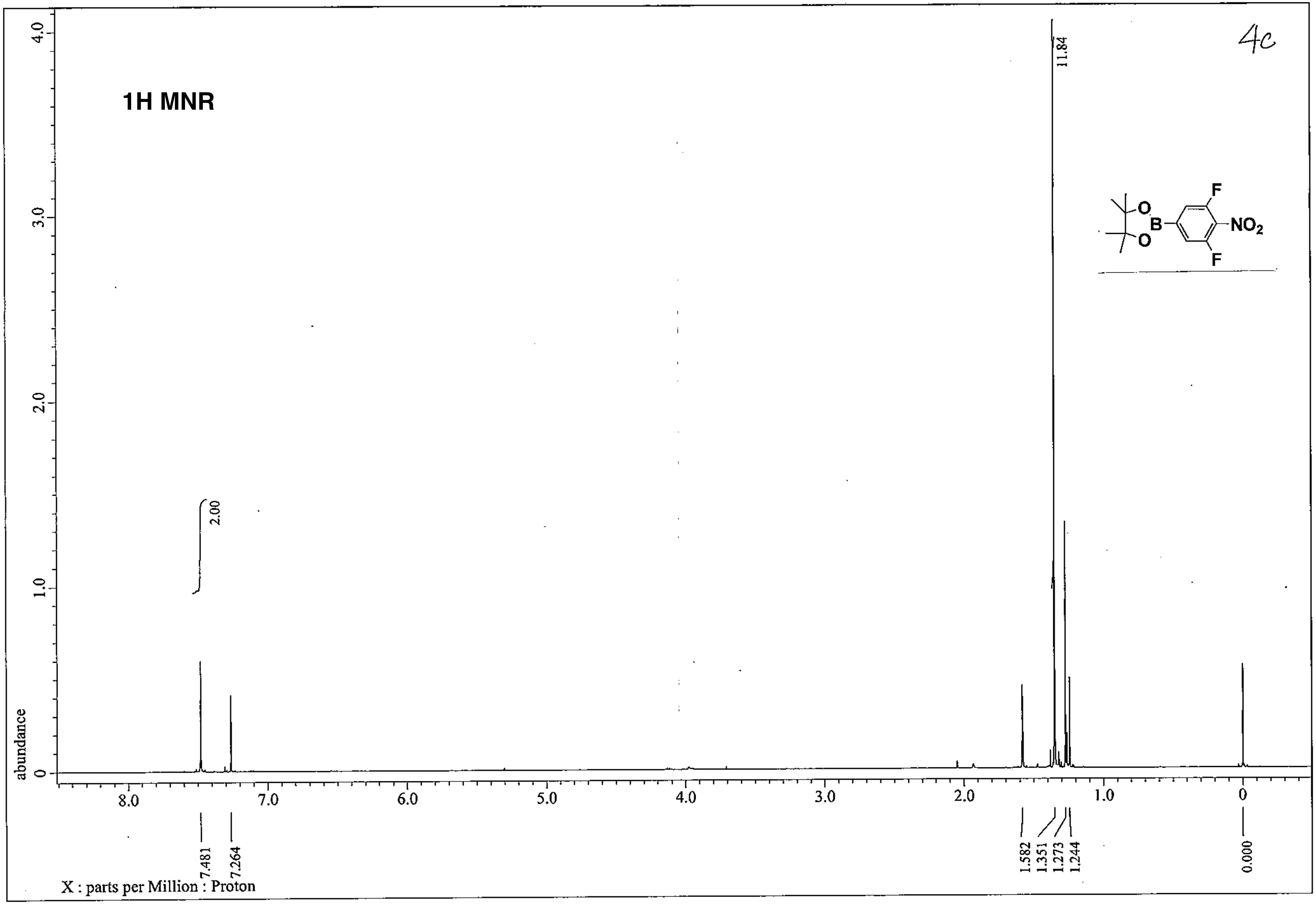

4c
1H MNR
11.84
2.00
abundance
4.0
3.0
2.0
1.0
0
8.0
7.0
6.0
5.0
4.0
3.0
2.0
1.0
0
7.481
7.264
1.582
1.351
1.273
1.244
0.000
X : parts per Million : Proton

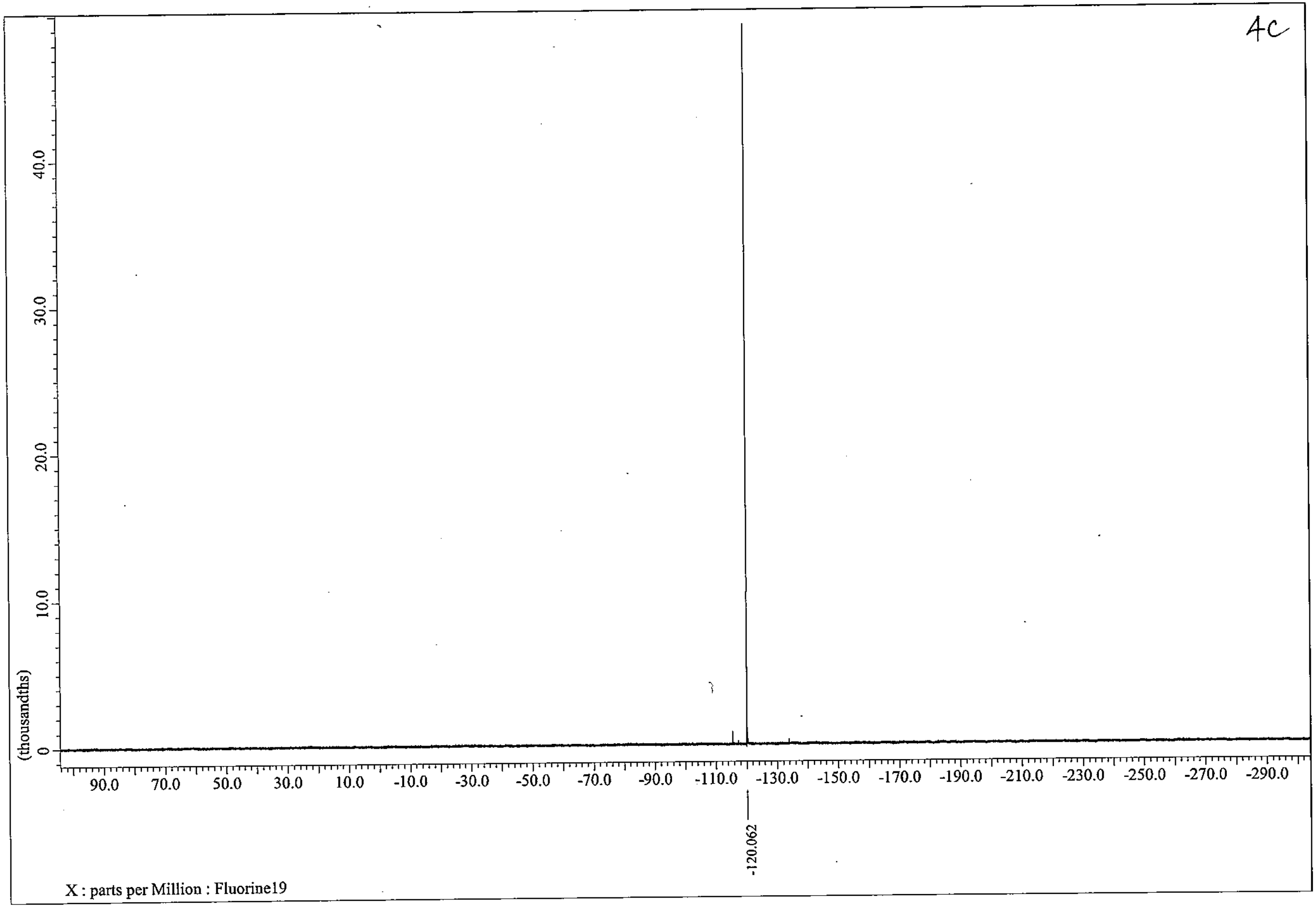
4c
(thousandths)
0
10.0
20.0
30.0
40.0
90.0
70.0
50.0
30.0
10.0
-10.0
-30.0
-50.0
-70.0
-90.0
-110.0
-130.0
-150.0
-170.0
-190.0
-210.0
-230.0
-250.0
-270.0
-290.0
-120.062
X : parts per Million : Fluorine19

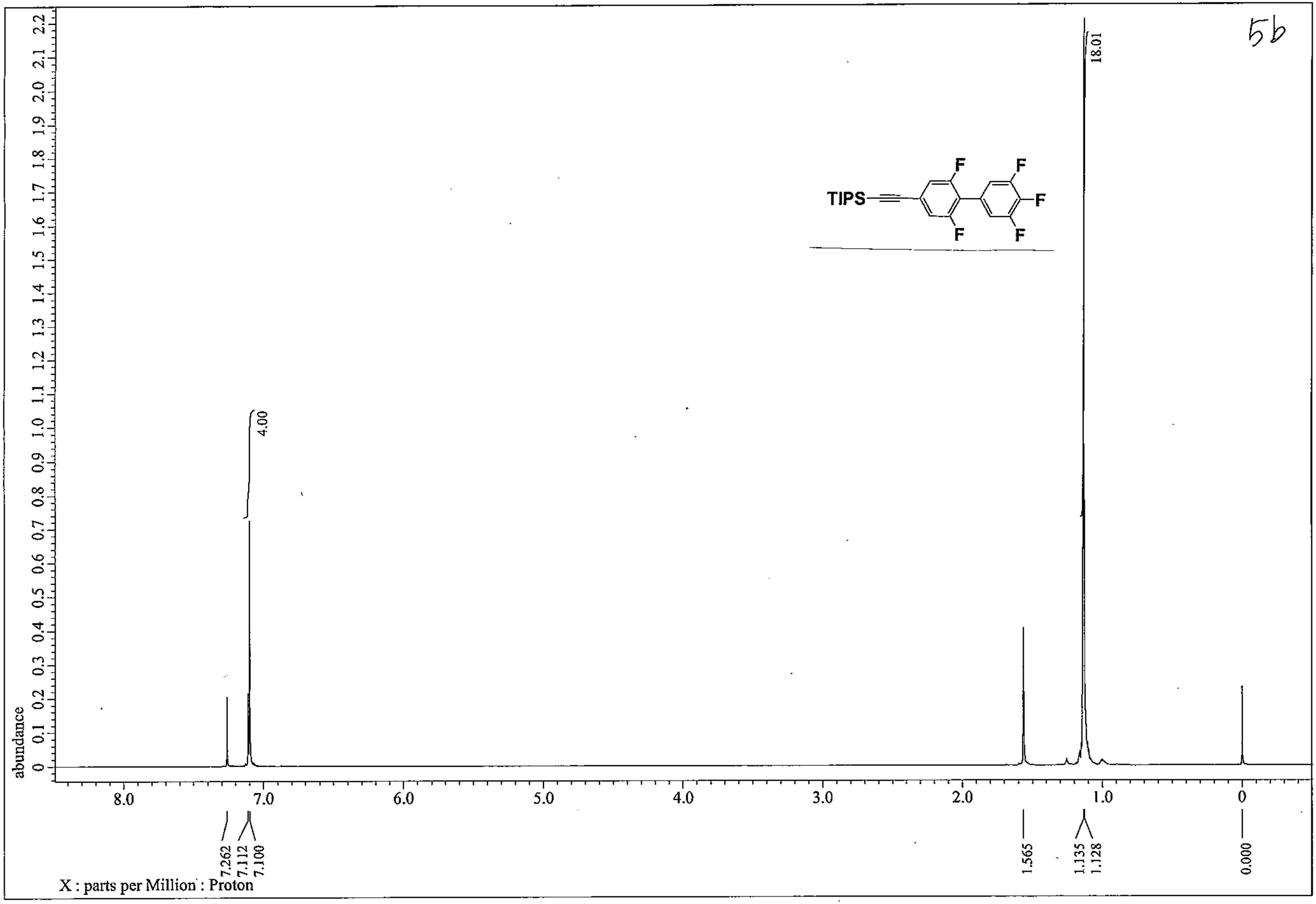

5b
TIPS
F
F
F
F
F
18.01
4.00
abundance
X : parts per Million : Proton
7.262
7.112
7.100
1.565
1.135
1.128
0.000

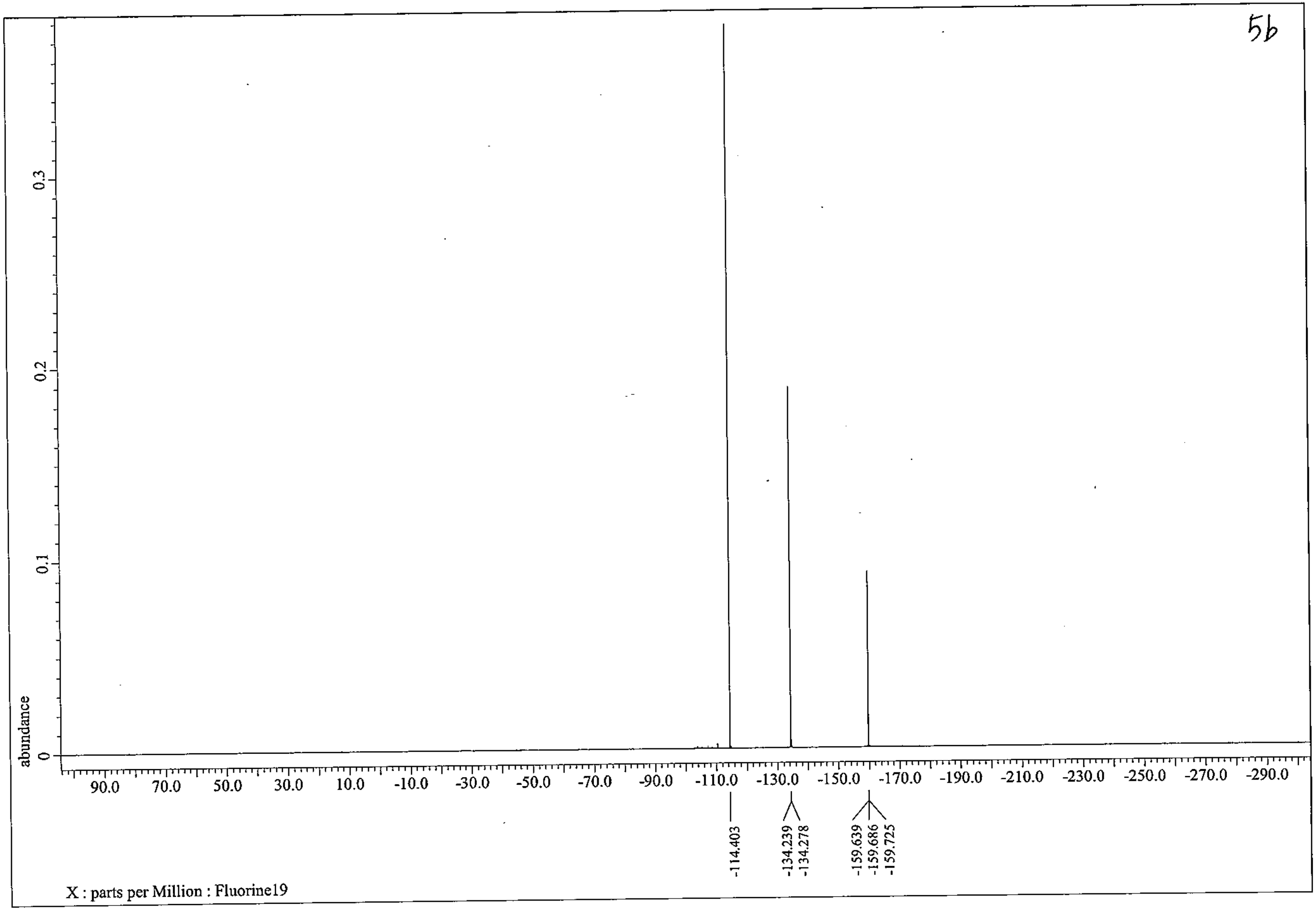
5b
abundance
0.3
0.2
0.1
0
90.0
70.0
50.0
30.0
10.0
-10.0
-30.0
-50.0
-70.0
-90.0
-110.0
-130.0
-150.0
-170.0
-190.0
-210.0
-230.0
-250.0
-270.0
-290.0
-114.403
-134.239
-134.278
-159.639
-159.686
-159.725
X : parts per Million : Fluorine19

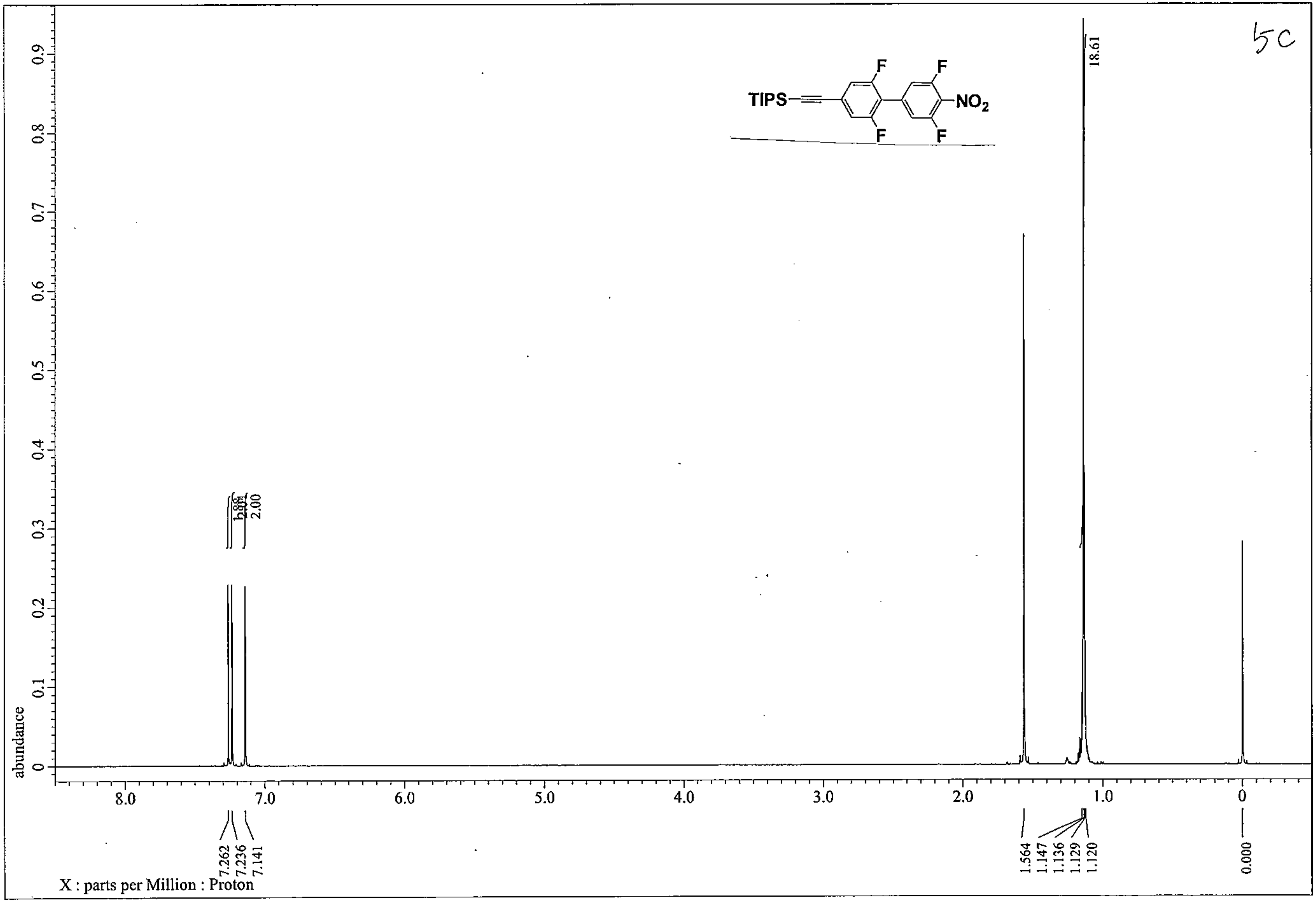
5c
TIPS
F
F
F
F
NO2
18.61
1.88
2.01
2.00
abundance
0.9
0.8
0.7
0.6
0.5
0.4
0.3
0.2
0.1
0
8.0
7.0
6.0
5.0
4.0
3.0
2.0
1.0
0
7.262
7.236
7.141
1.564
1.147
1.136
1.129
1.120
0.000
X : parts per Million : Proton

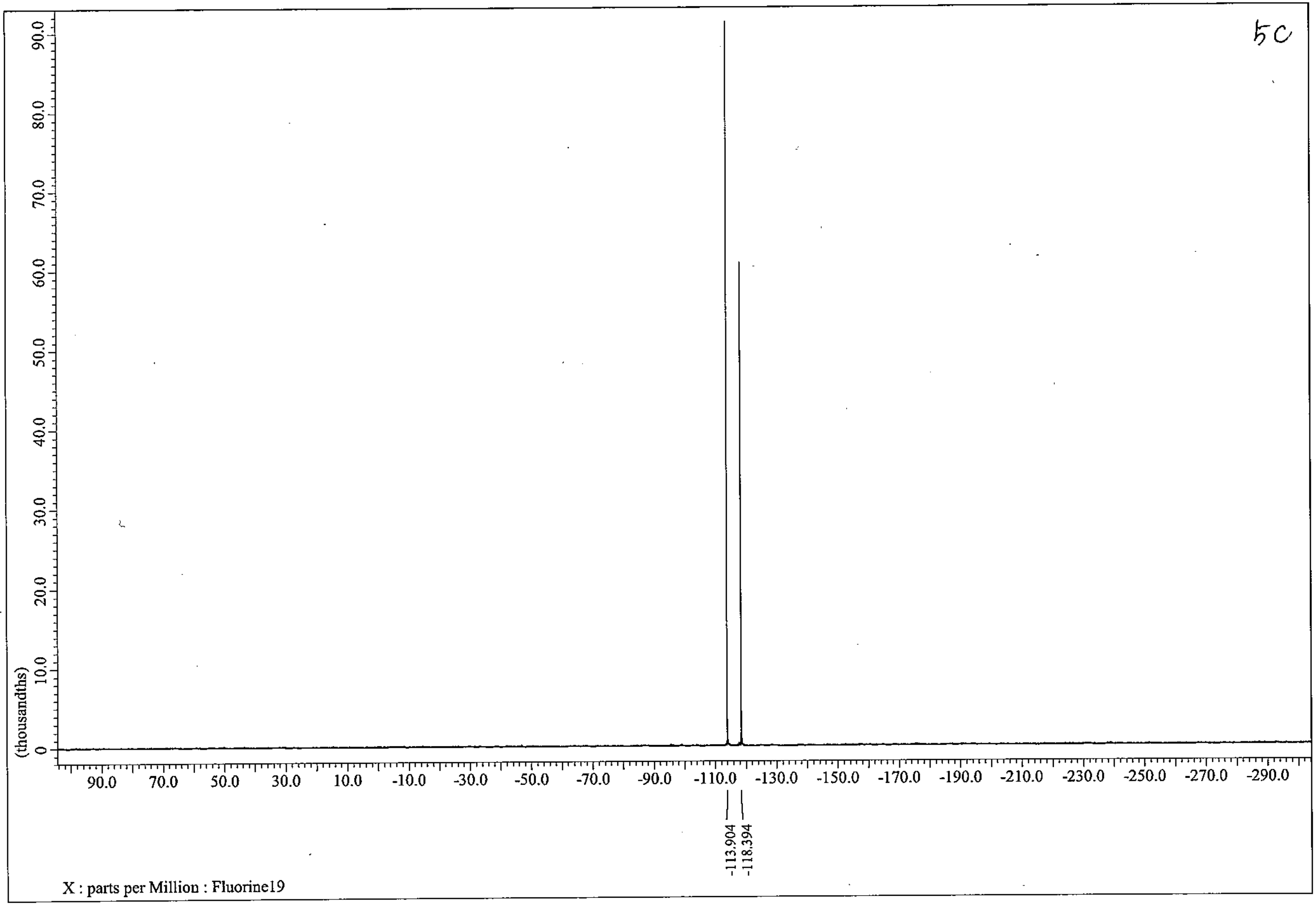
5c
(thousandths)
90.0
80.0
70.0
60.0
50.0
40.0
30.0
20.0
10.0
0
90.0
70.0
50.0
30.0
10.0
-10.0
-30.0
-50.0
-70.0
-90.0
-110.0
-130.0
-150.0
-170.0
-190.0
-210.0
-230.0
-250.0
-270.0
-290.0
-113.904
-118.394
X : parts per Million : Fluorine19

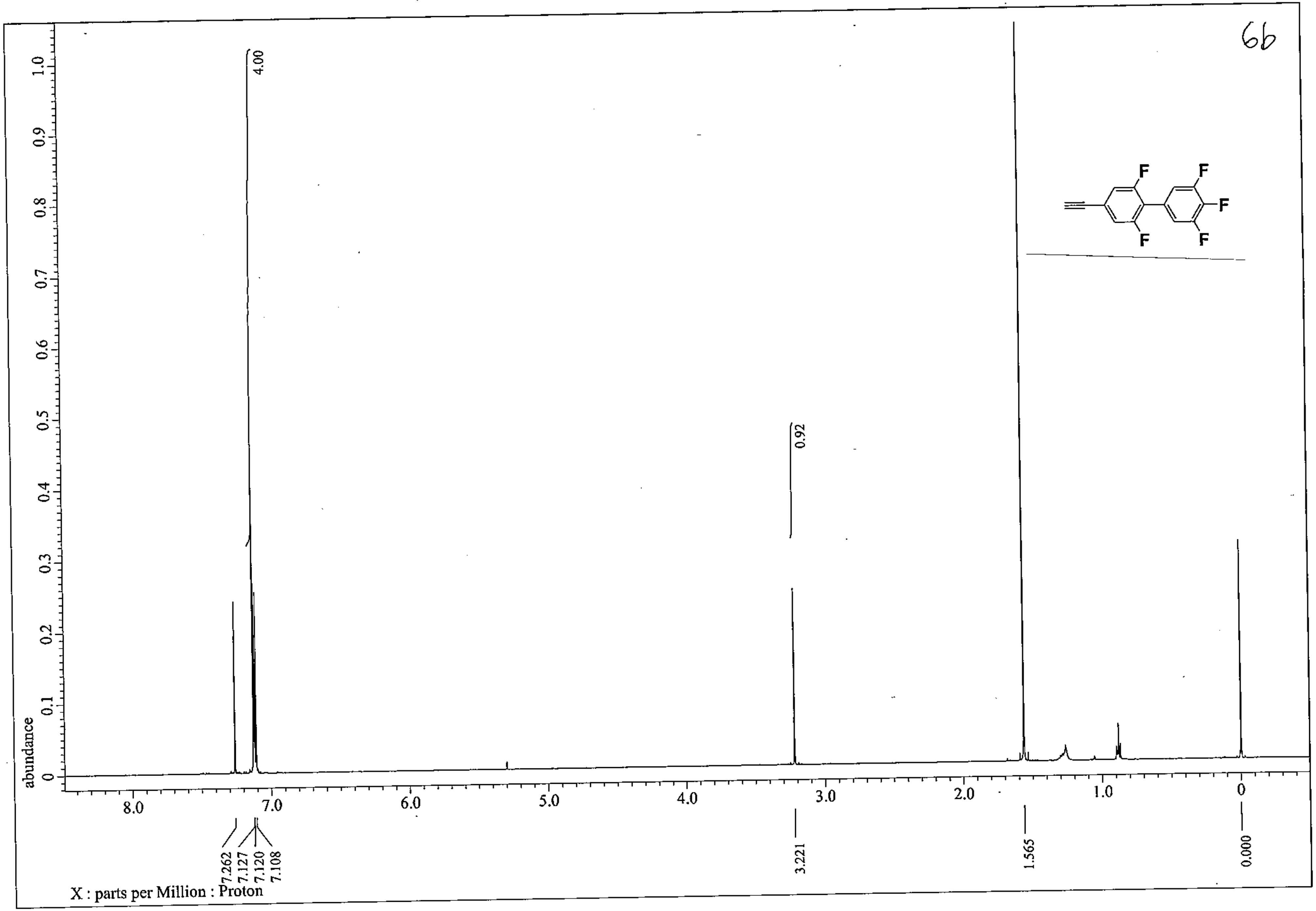
6b
F
F
F
F
F
4.00
0.92
abundance
0
0.1
0.2
0.3
0.4
0.5
0.6
0.7
0.8
0.9
1.0
8.0
7.0
6.0
5.0
4.0
3.0
2.0
1.0
0
7.262
7.127
7.120
7.108
3.221
1.565
0.000
X : parts per Million : Proton

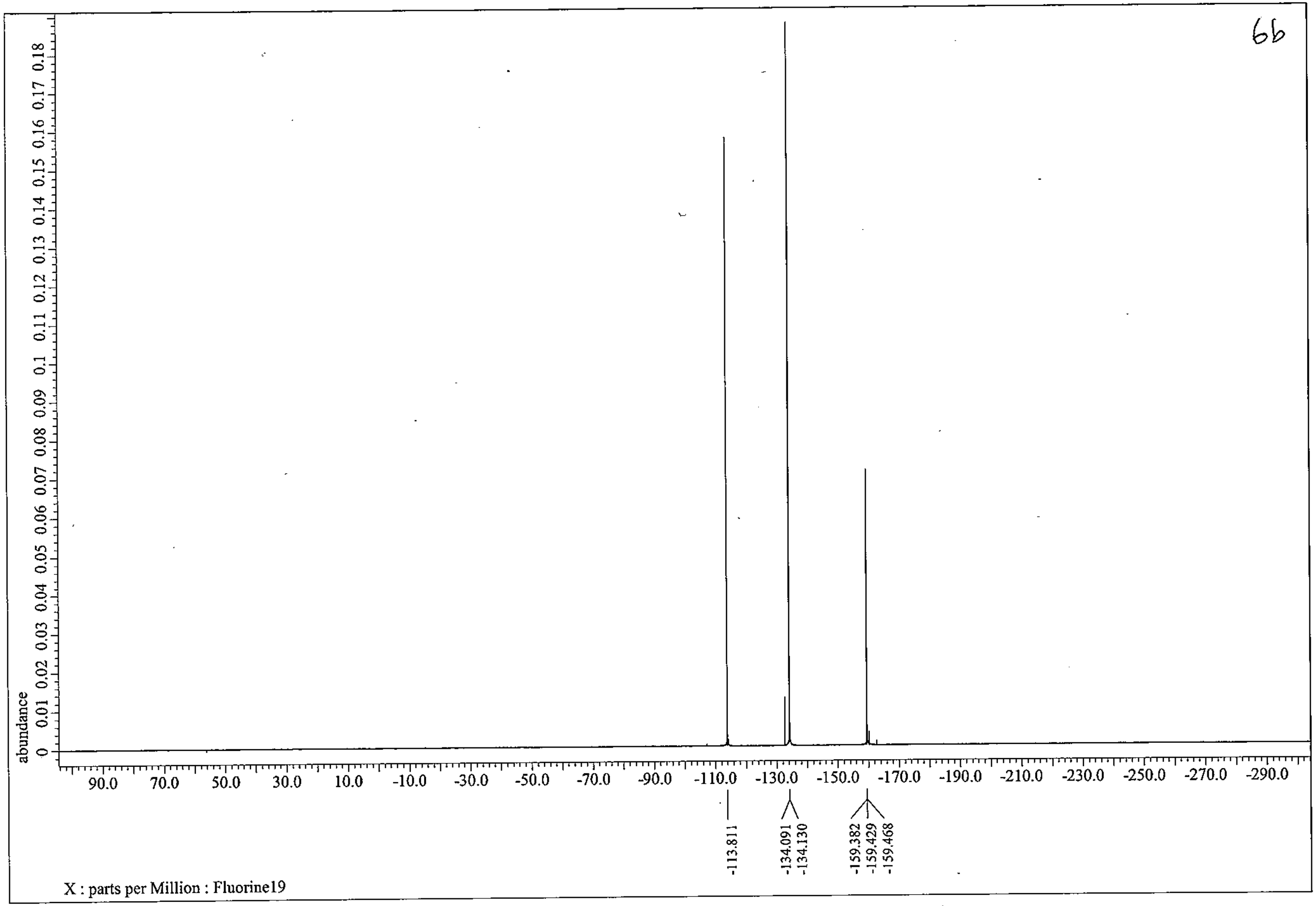
6b
abundance
0 0.01 0.02 0.03 0.04 0.05 0.06 0.07 0.08 0.09 0.1 0.11 0.12 0.13 0.14 0.15 0.16 0.17 0.18
90.0 70.0 50.0 30.0 10.0 -10.0 -30.0 -50.0 -70.0 -90.0 -110.0 -130.0 -150.0 -170.0 -190.0 -210.0 -230.0 -250.0 -270.0 -290.0
-113.811
-134.091
-134.130
-159.382
-159.429
-159.468
X : parts per Million : Fluorine19

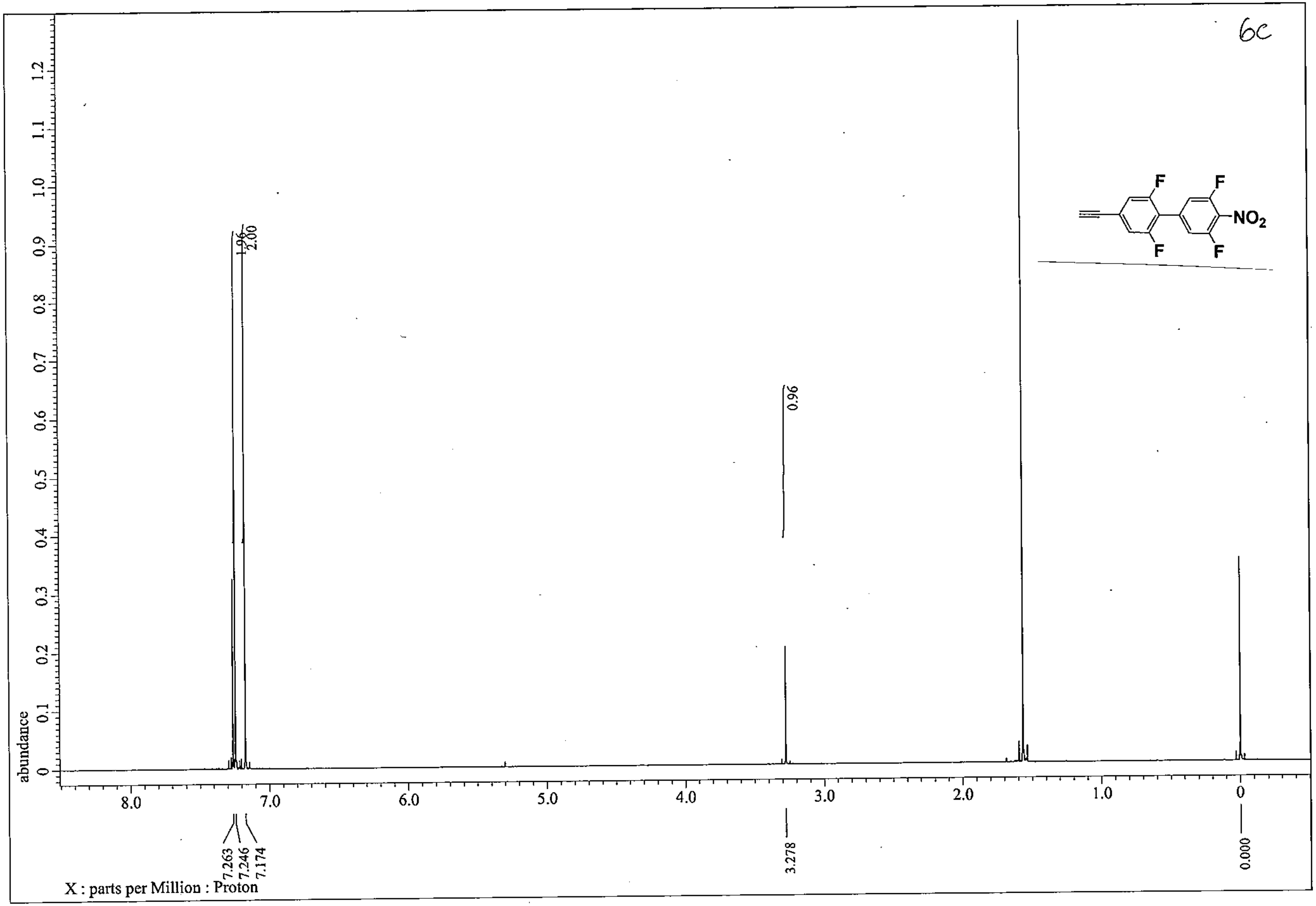
6c
F
F
NO2
F
F
abundance
0
0.1
0.2
0.3
0.4
0.5
0.6
0.7
0.8
0.9
1.0
1.1
1.2
1.96
2.00
0.96
8.0
7.0
6.0
5.0
4.0
3.0
2.0
1.0
0
7.263
7.246
7.174
3.278
0.000
X : parts per Million : Proton

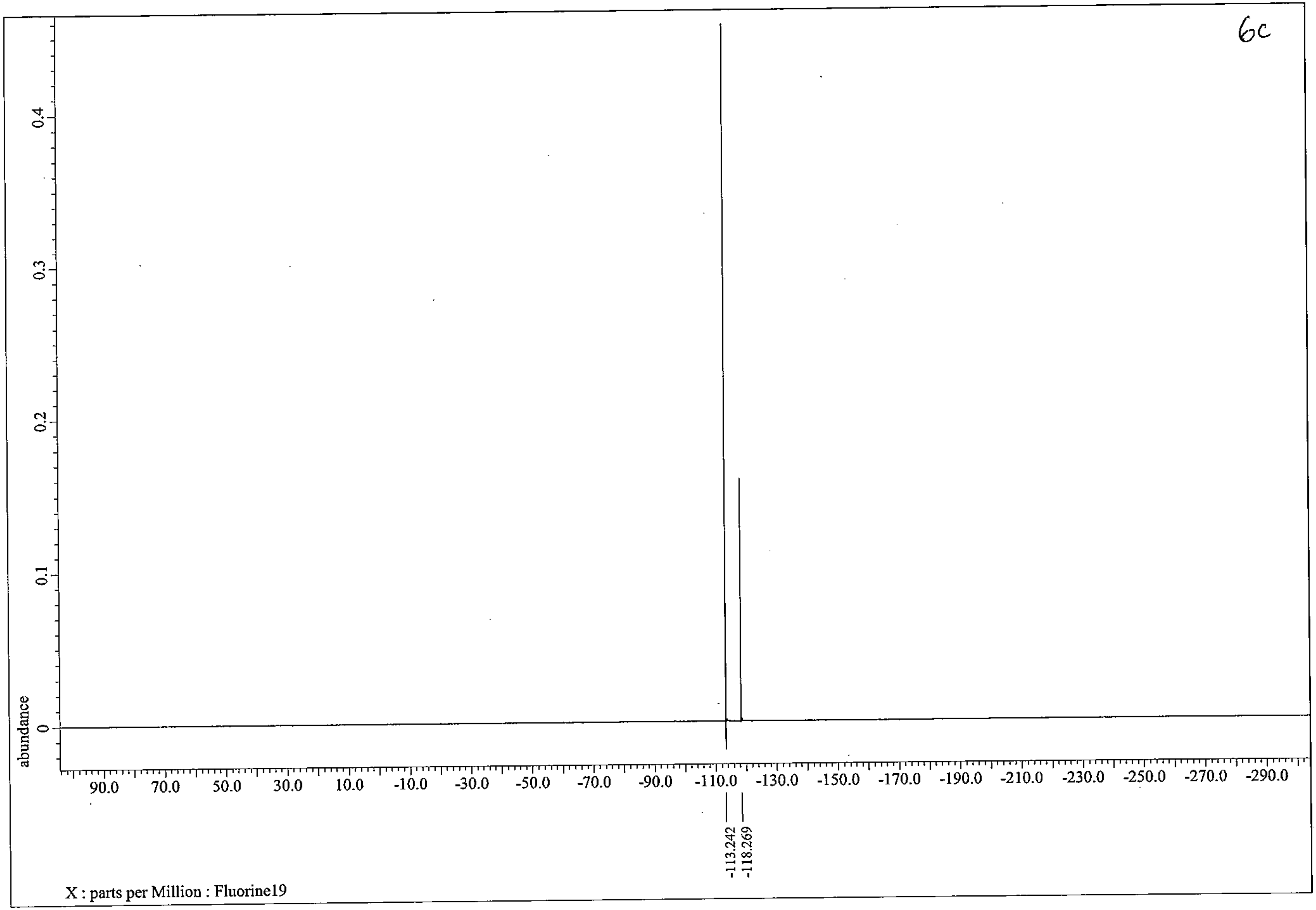

6c
abundance
0.4
0.3
0.2
0.1
0
90.0
70.0
50.0
30.0
10.0
-10.0
-30.0
-50.0
-70.0
-90.0
-110.0
-130.0
-150.0
-170.0
-190.0
-210.0
-230.0
-250.0
-270.0
-290.0
-113.242
-118.269
X : parts per Million : Fluorine19

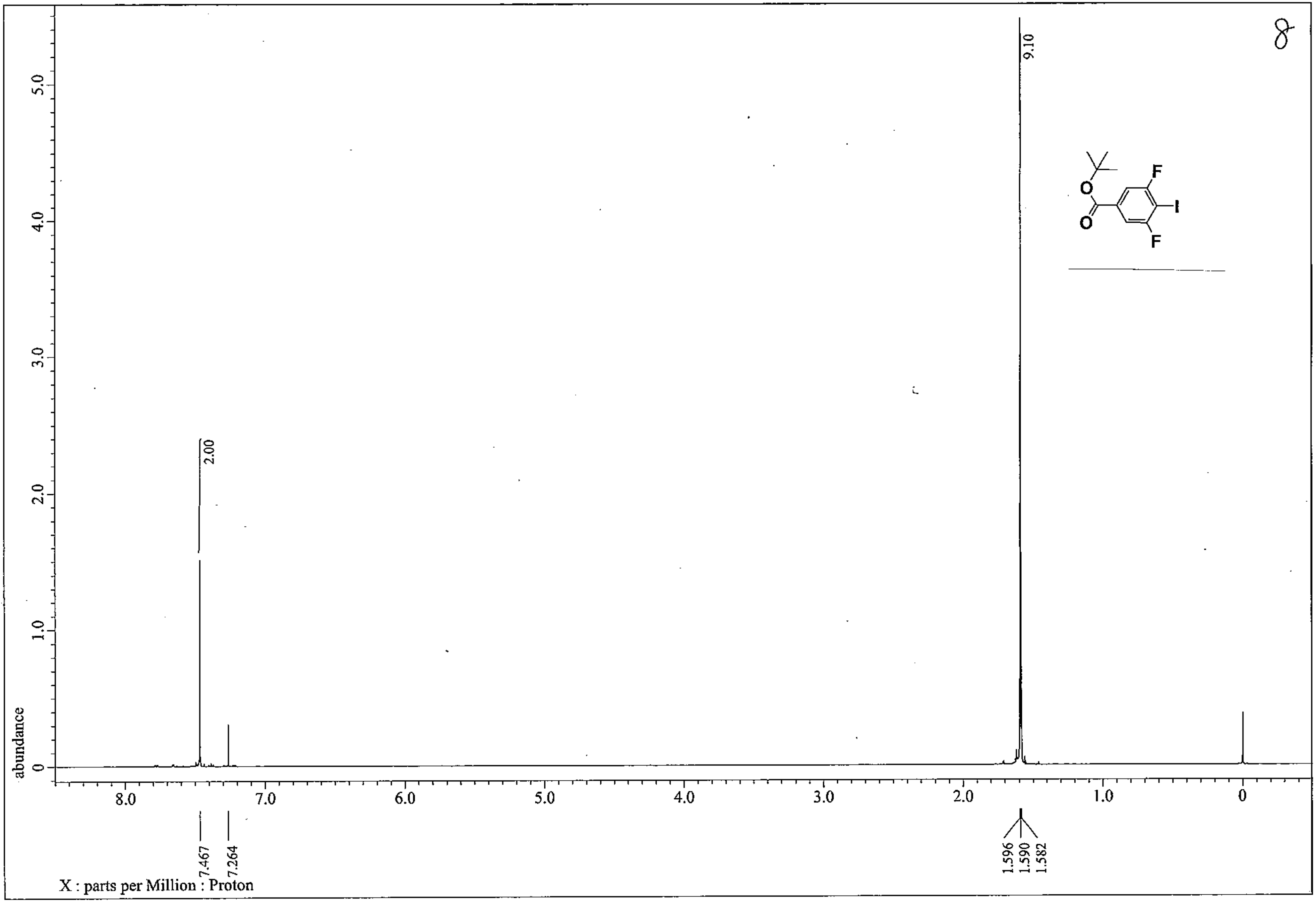

8
9.10
2.00
5.0
4.0
3.0
2.0
1.0
0
abundance
8.0
7.0
6.0
5.0
4.0
3.0
2.0
1.0
0
7.467
7.264
1.596
1.590
1.582
X : parts per Million : Proton
F
I
F
O
O

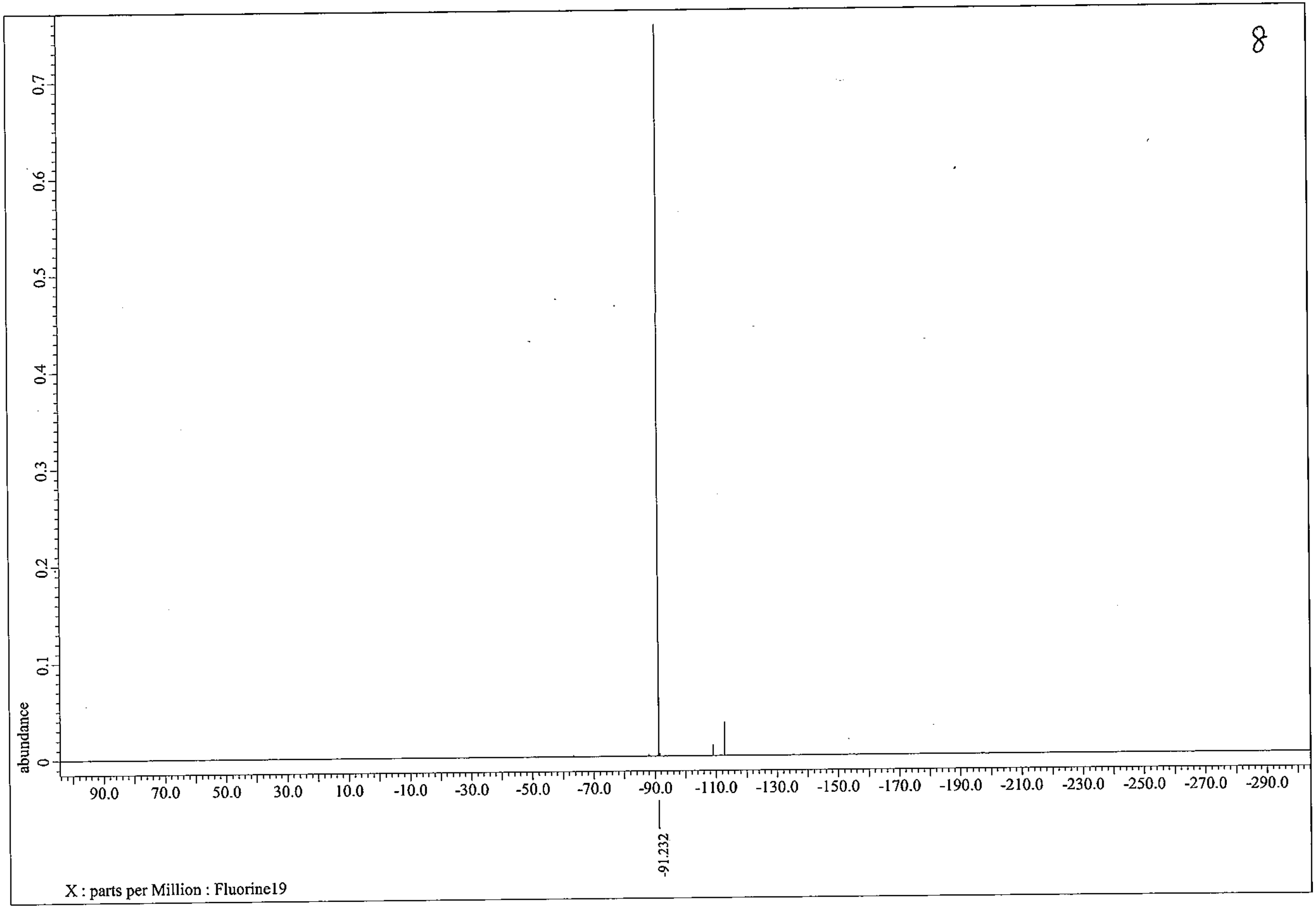
8
abundance
0
0.1
0.2
0.3
0.4
0.5
0.6
0.7
90.0
70.0
50.0
30.0
10.0
-10.0
-30.0
-50.0
-70.0
-90.0
-110.0
-130.0
-150.0
-170.0
-190.0
-210.0
-230.0
-250.0
-270.0
-290.0
-91.232
X : parts per Million : Fluorine19

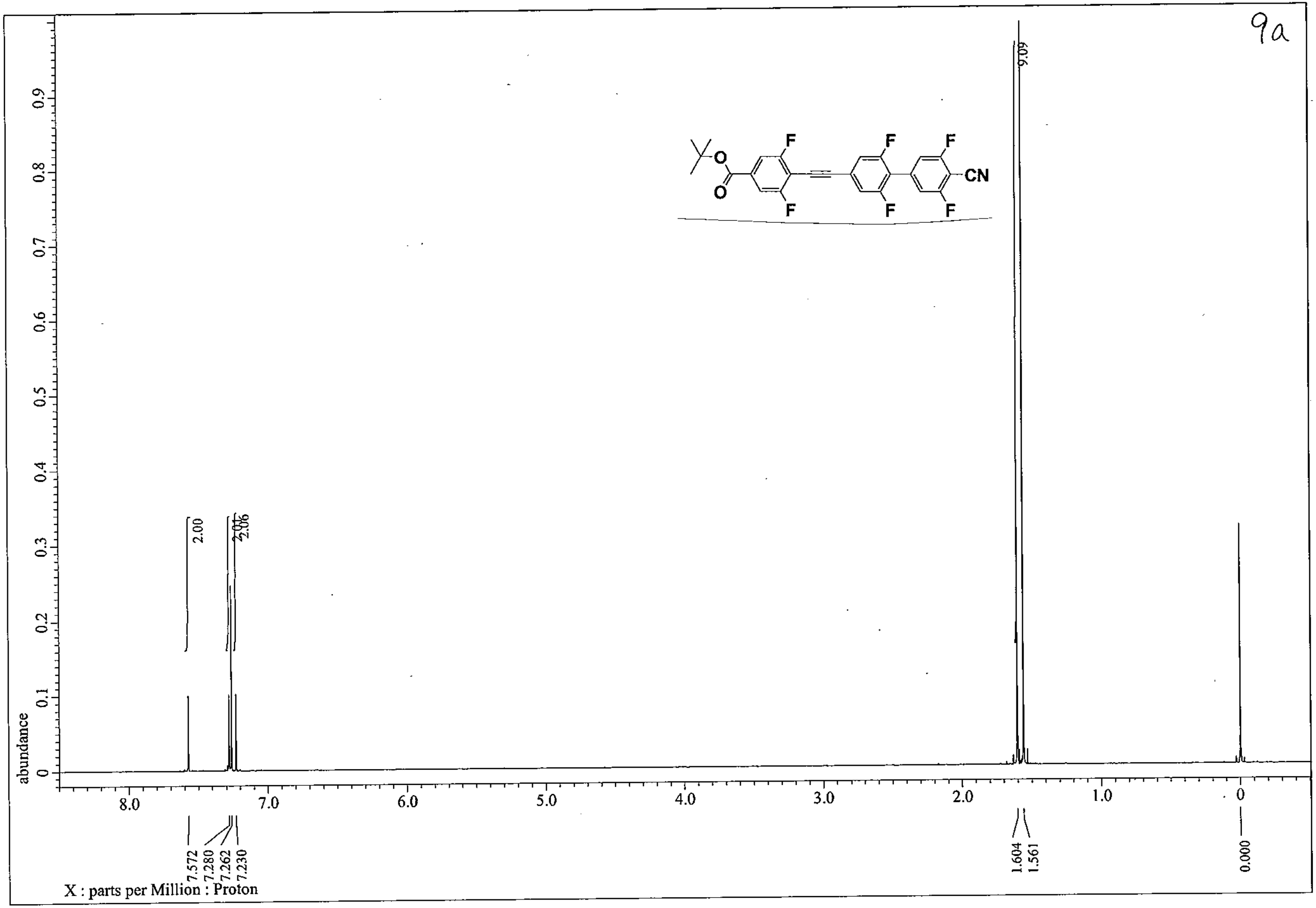
9a
CN
F
F
F
F
F
F
O
O
9.09
2.00
2.01
2.06
abundance
0
0.1
0.2
0.3
0.4
0.5
0.6
0.7
0.8
0.9
8.0
7.0
6.0
5.0
4.0
3.0
2.0
1.0
0
7.572
7.280
7.262
7.230
1.604
1.561
0.000
X : parts per Million : Proton

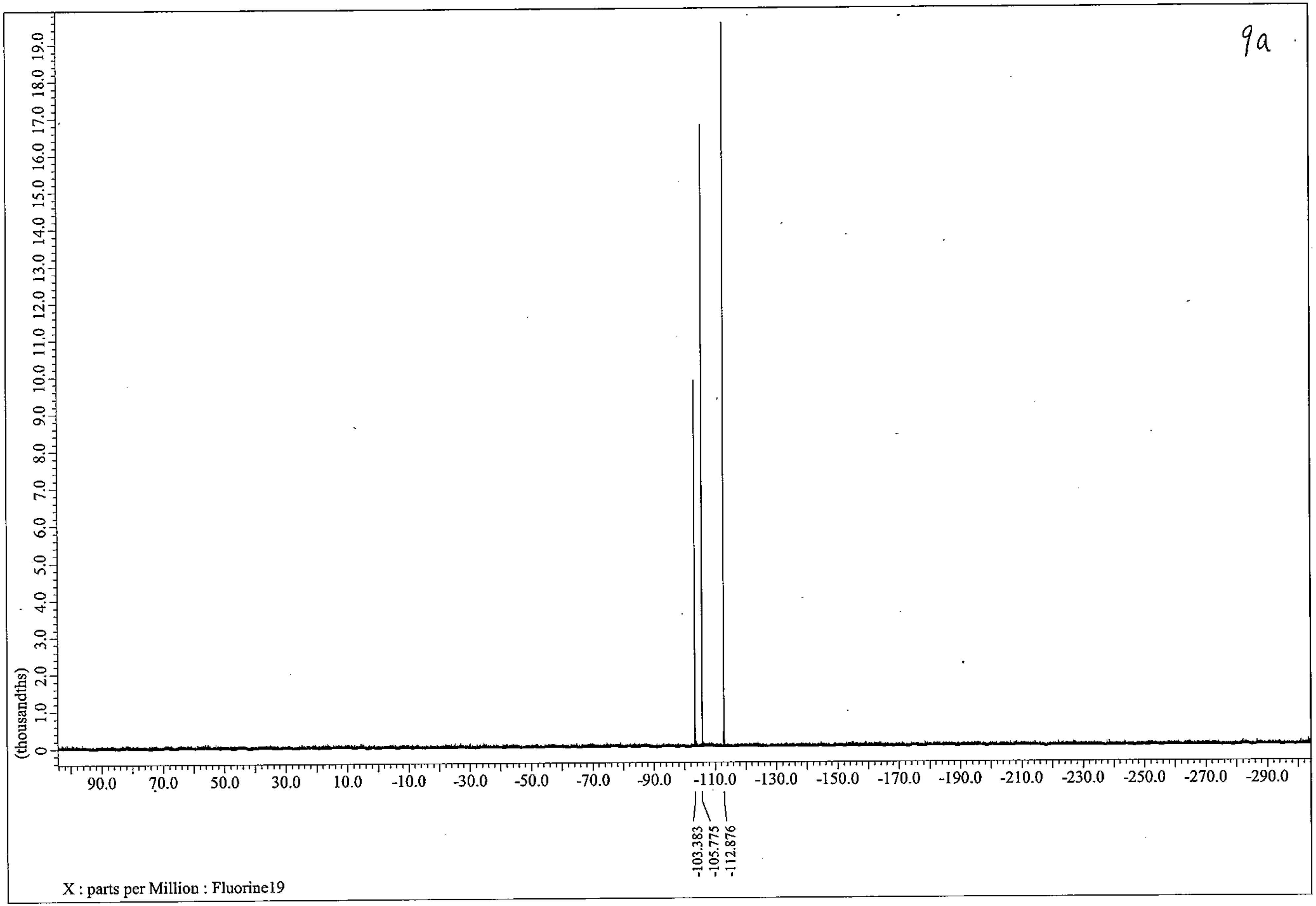
9a
(thousandths)
0
1.0
2.0
3.0
4.0
5.0
6.0
7.0
8.0
9.0
10.0
11.0
12.0
13.0
14.0
15.0
16.0
17.0
18.0
19.0
90.0
70.0
50.0
30.0
10.0
-10.0
-30.0
-50.0
-70.0
-90.0
-110.0
-130.0
-150.0
-170.0
-190.0
-210.0
-230.0
-250.0
-270.0
-290.0
-103.383
-105.775
-112.876
X : parts per Million : Fluorine19

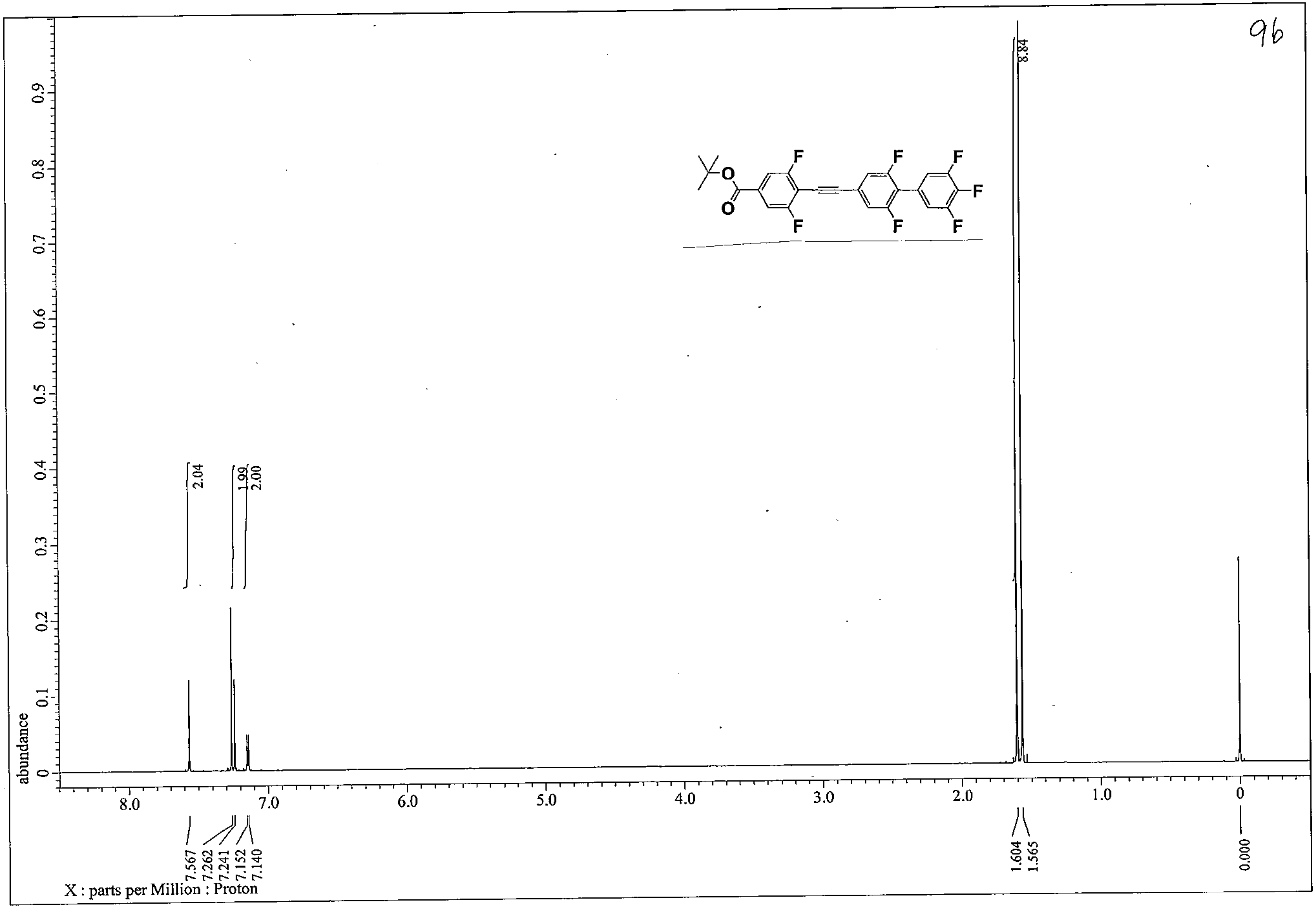

96
abundance
0
0.1
0.2
0.3
0.4
0.5
0.6
0.7
0.8
0.9
2.04
1.99
2.00
8.84
F
O
O
8.0
7.0
6.0
5.0
4.0
3.0
2.0
1.0
0
7.567
7.262
7.241
7.152
7.140
1.604
1.565
0.000
X : parts per Million : Proton

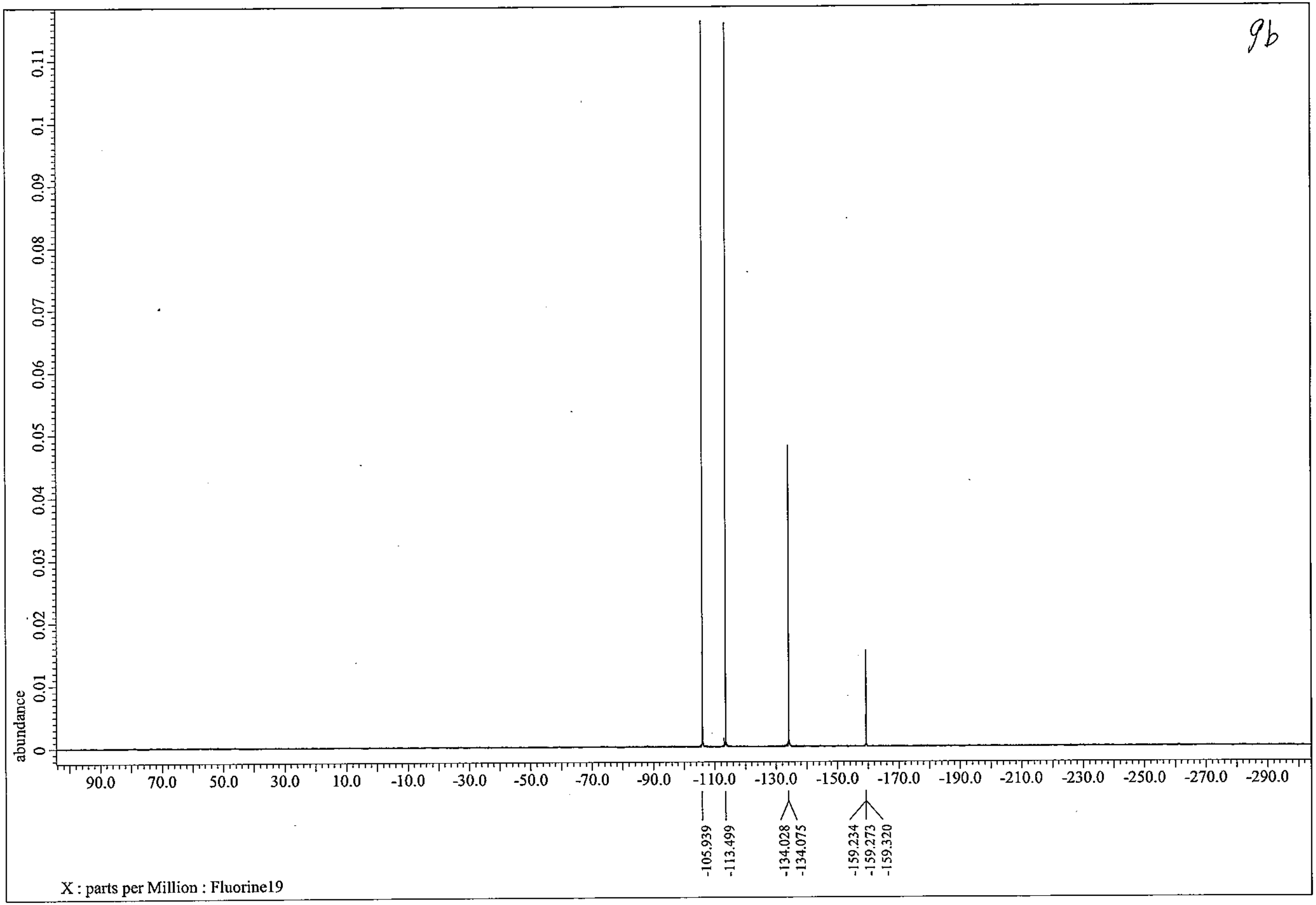

9b
abundance
0
0.01
0.02
0.03
0.04
0.05
0.06
0.07
0.08
0.09
0.1
0.11
90.0
70.0
50.0
30.0
10.0
-10.0
-30.0
-50.0
-70.0
-90.0
-110.0
-130.0
-150.0
-170.0
-190.0
-210.0
-230.0
-250.0
-270.0
-290.0
-105.939
-113.499
-134.028
-134.075
-159.234
-159.273
-159.320
X : parts per Million : Fluorine19

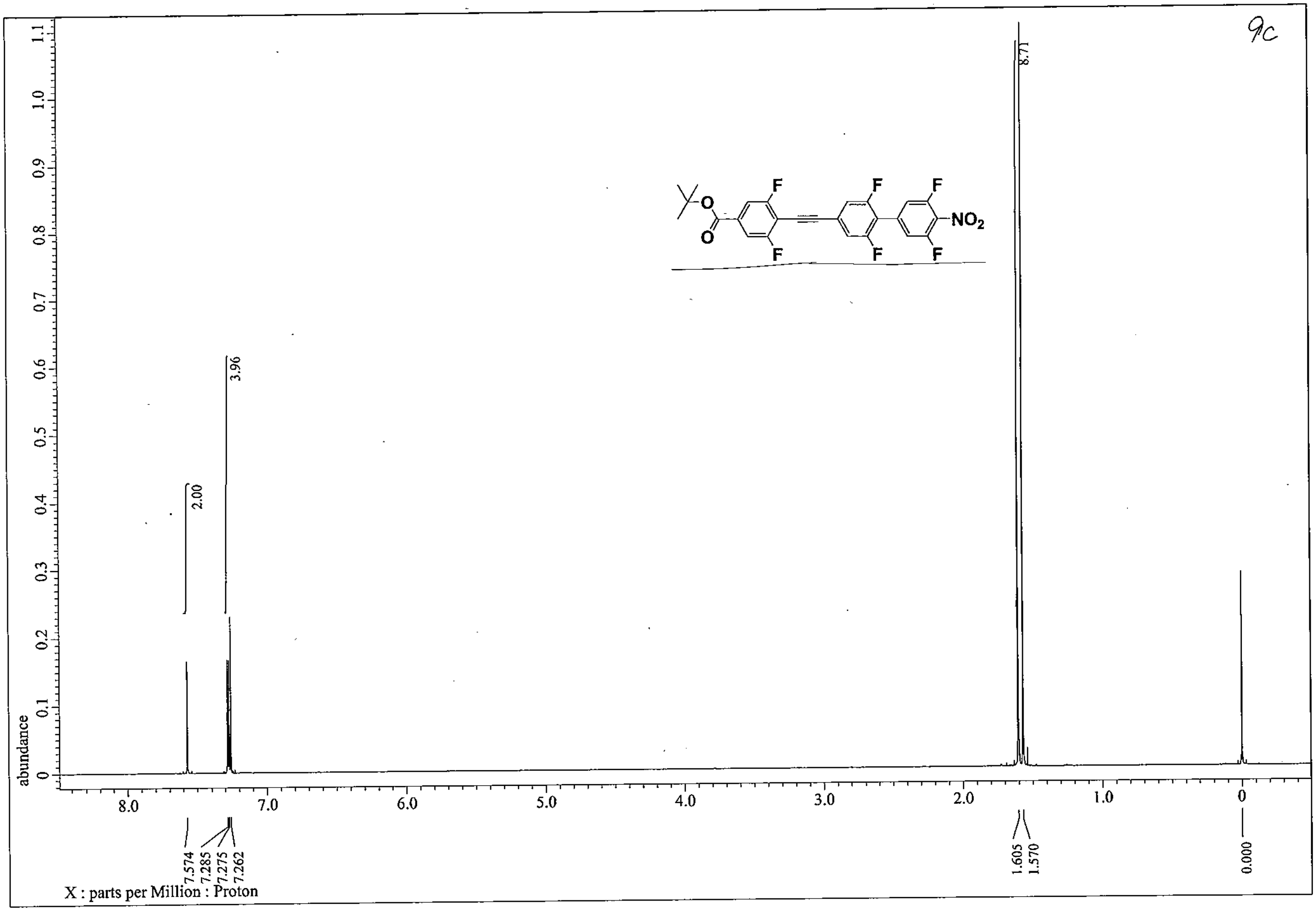
9c
NO2
F
abundance
X : parts per Million : Proton
8.0
7.0
6.0
5.0
4.0
3.0
2.0
1.0
0
2.00
3.96
8.71
7.574
7.285
7.275
7.262
1.605
1.570
0.000

9c.

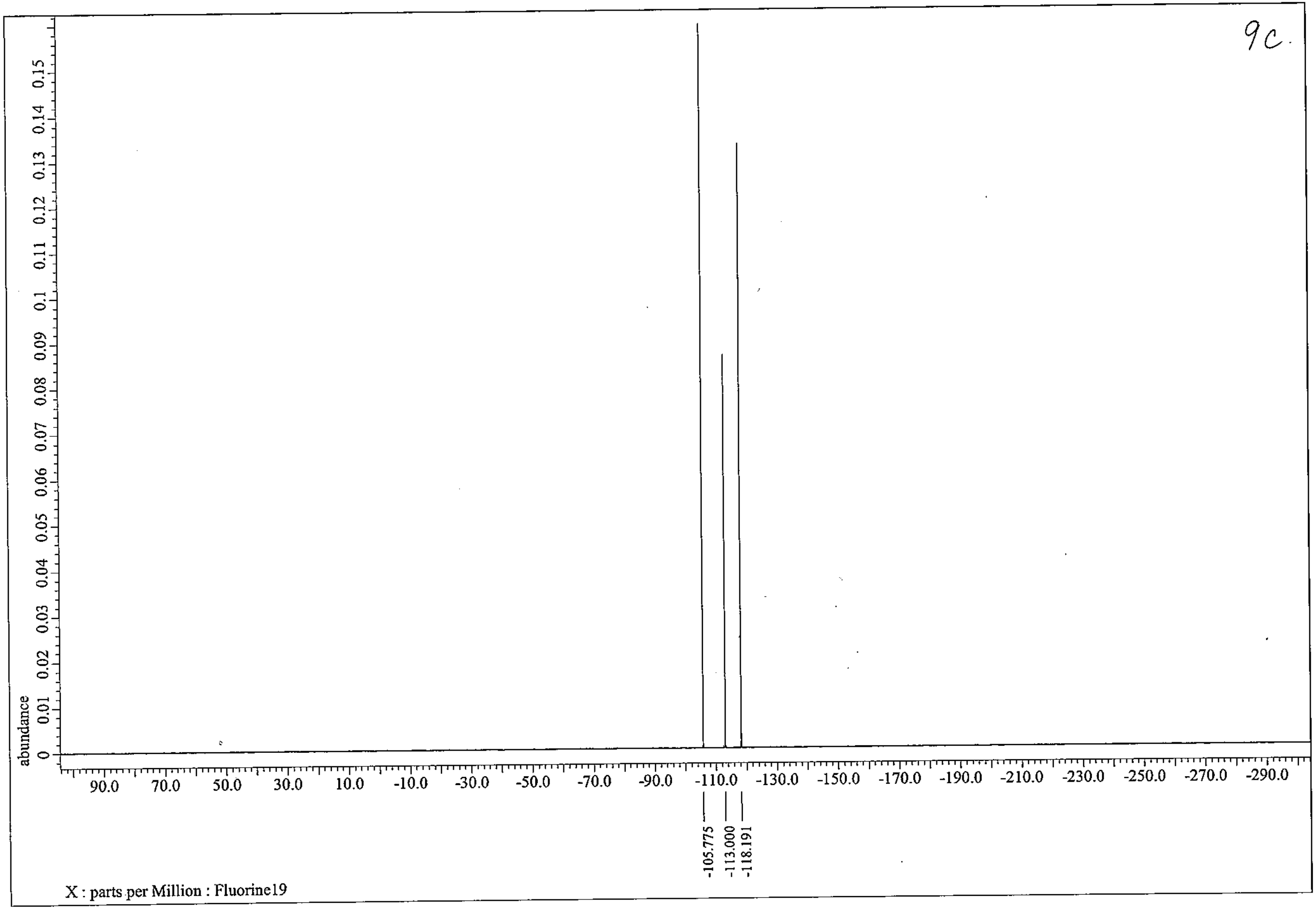

abundance
0
0.01
0.02
0.03
0.04
0.05
0.06
0.07
0.08
0.09
0.1
0.11
0.12
0.13
0.14
0.15
90.0 70.0 50.0 30.0 10.0 -10.0 -30.0 -50.0 -70.0 -90.0 -110.0 -130.0 -150.0 -170.0 -190.0 -210.0 -230.0 -250.0 -270.0 -290.0
-105.775
-113.000
-118.191
X : parts per Million : Fluorine19

10a

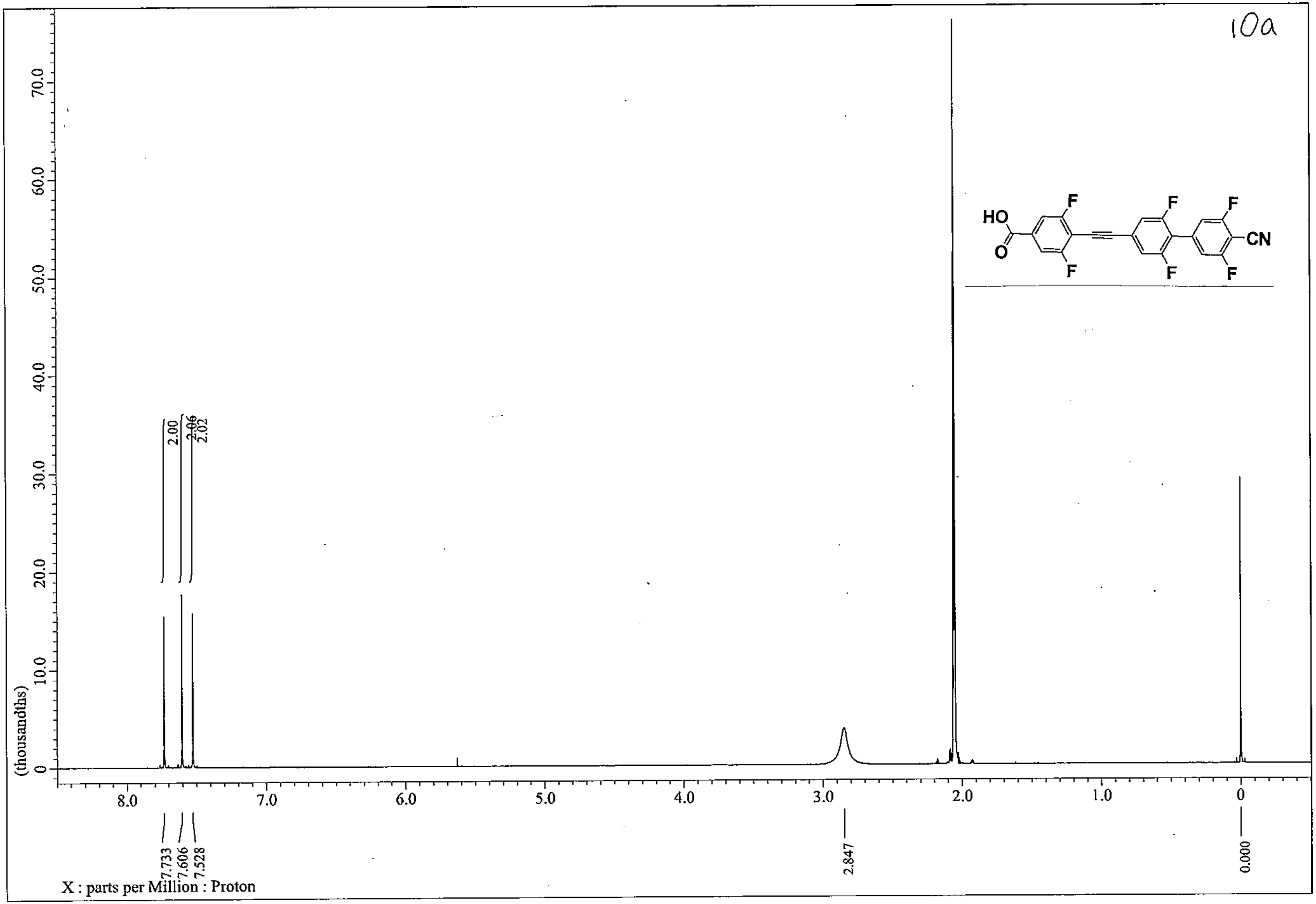
HO
O
F
F
F
F
F
F
CN
2.00
2.06
2.02
7.733
7.606
7.528
2.847
0.000
(thousandths)
X : parts per Million : Proton

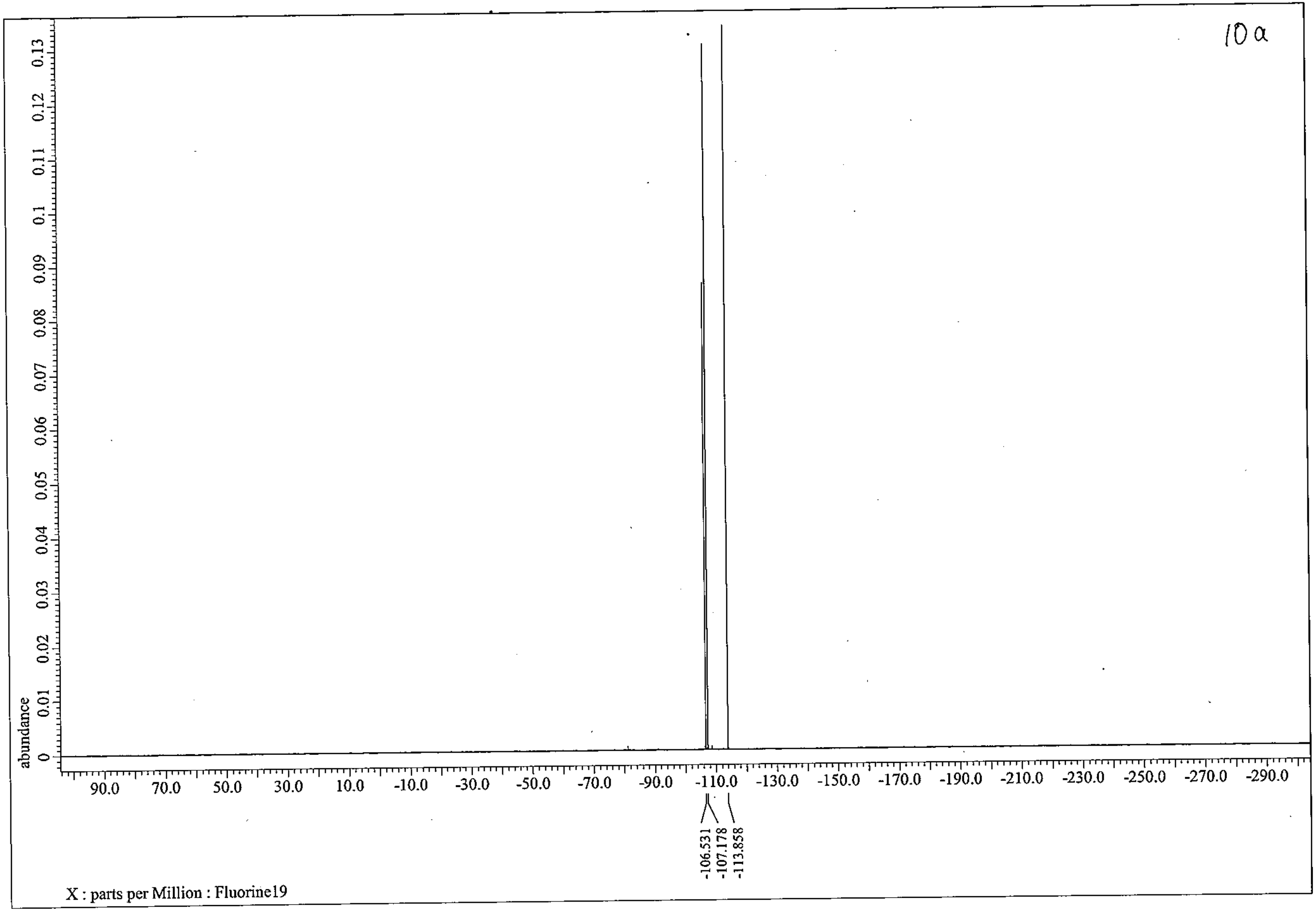
10a
abundance
0.13
0.12
0.11
0.1
0.09
0.08
0.07
0.06
0.05
0.04
0.03
0.02
0.01
0
90.0
70.0
50.0
30.0
10.0
-10.0
-30.0
-50.0
-70.0
-90.0
-110.0
-130.0
-150.0
-170.0
-190.0
-210.0
-230.0
-250.0
-270.0
-290.0
-106.531
-107.178
-113.858
X : parts per Million : Fluorine19

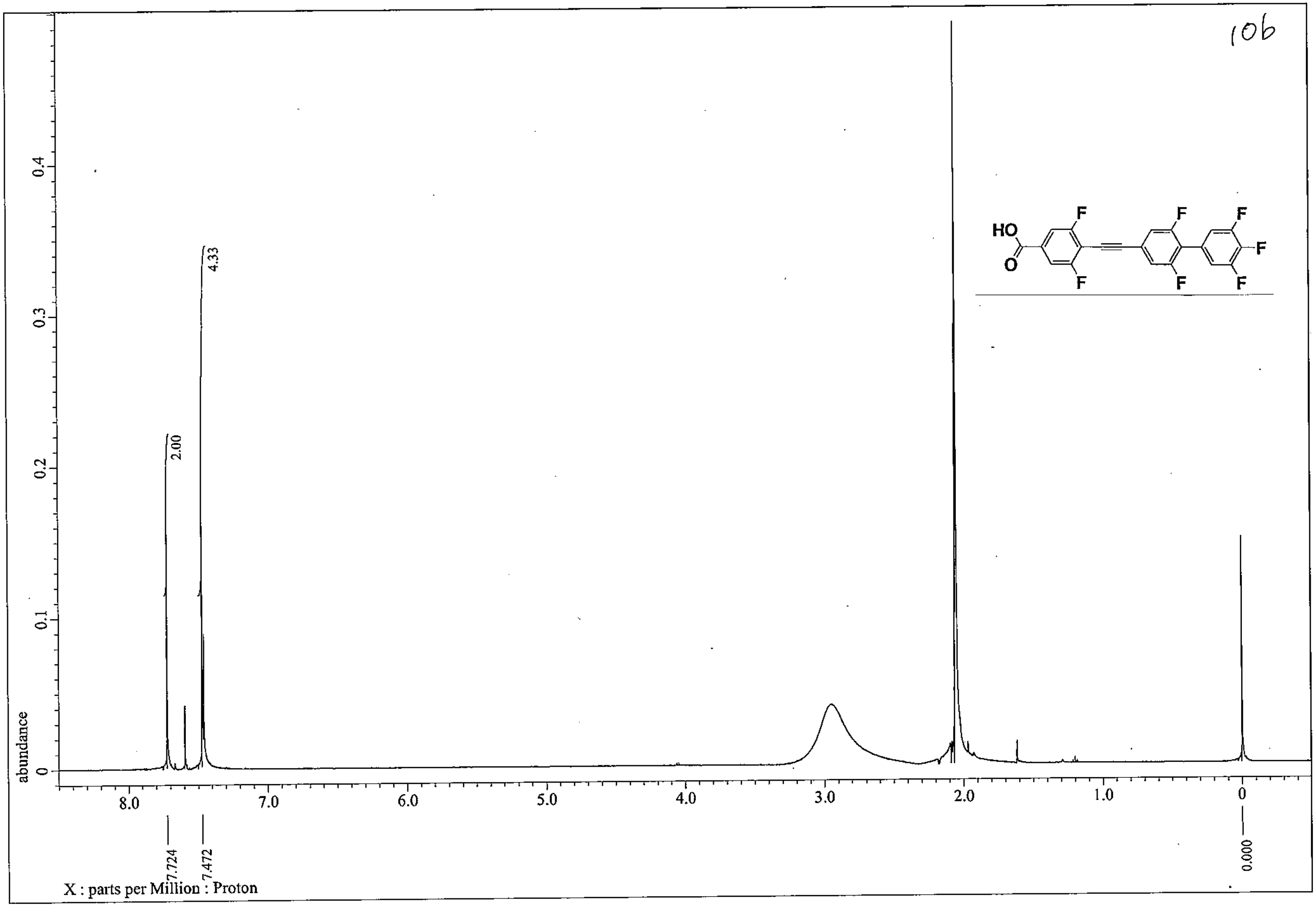
10b
HO
O
F
F
F
F
F
F
F
0.4
0.3
0.2
0.1
0
abundance
2.00
4.33
8.0
7.0
6.0
5.0
4.0
3.0
2.0
1.0
0
7.724
7.472
0.000
X : parts per Million : Proton

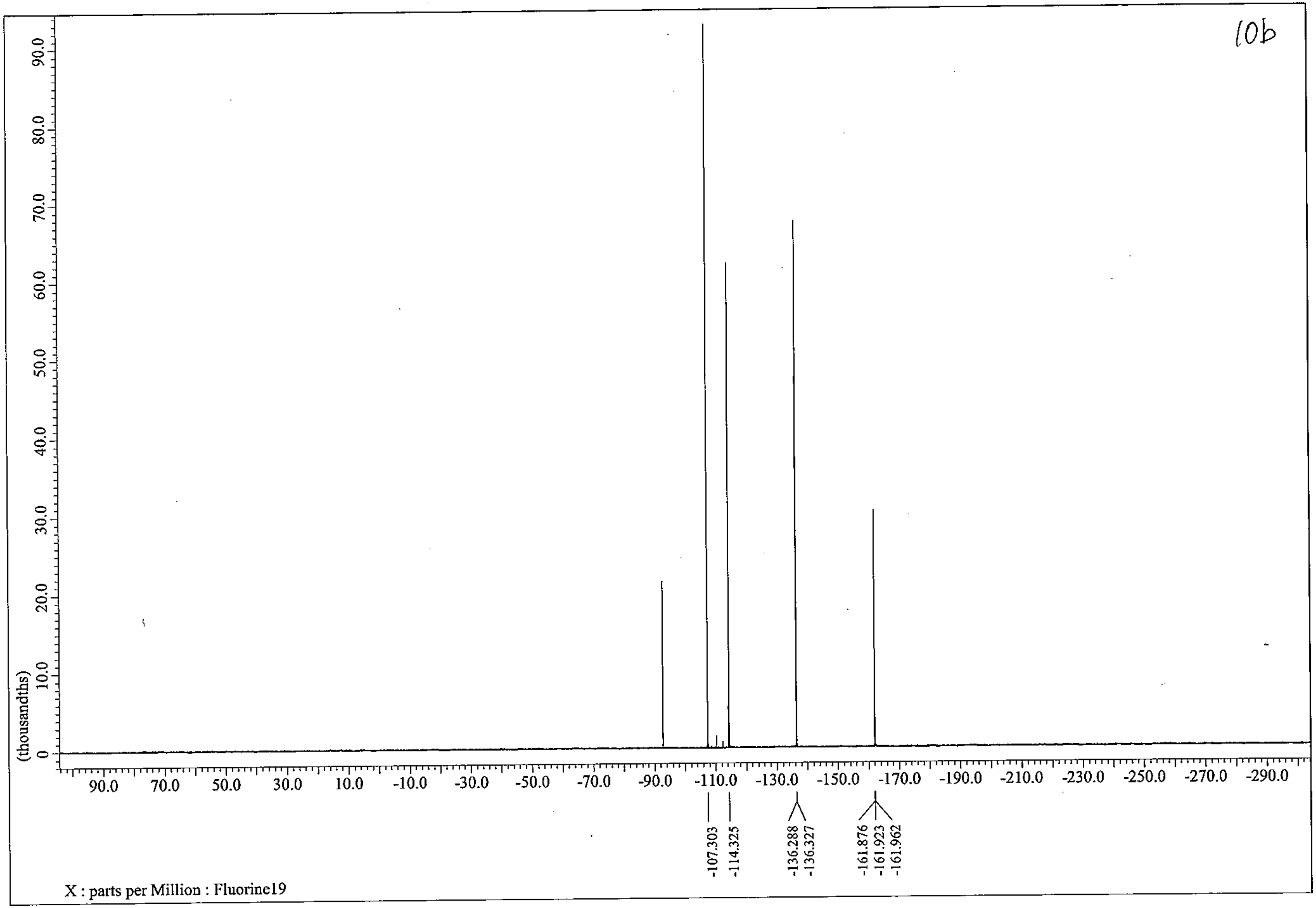

10b
(thousandths)
0
10.0
20.0
30.0
40.0
50.0
60.0
70.0
80.0
90.0
90.0
70.0
50.0
30.0
10.0
-10.0
-30.0
-50.0
-70.0
-90.0
-110.0
-130.0
-150.0
-170.0
-190.0
-210.0
-230.0
-250.0
-270.0
-290.0
-107.303
-114.325
-136.288
-136.327
-161.876
-161.923
-161.962
X : parts per Million : Fluorine19

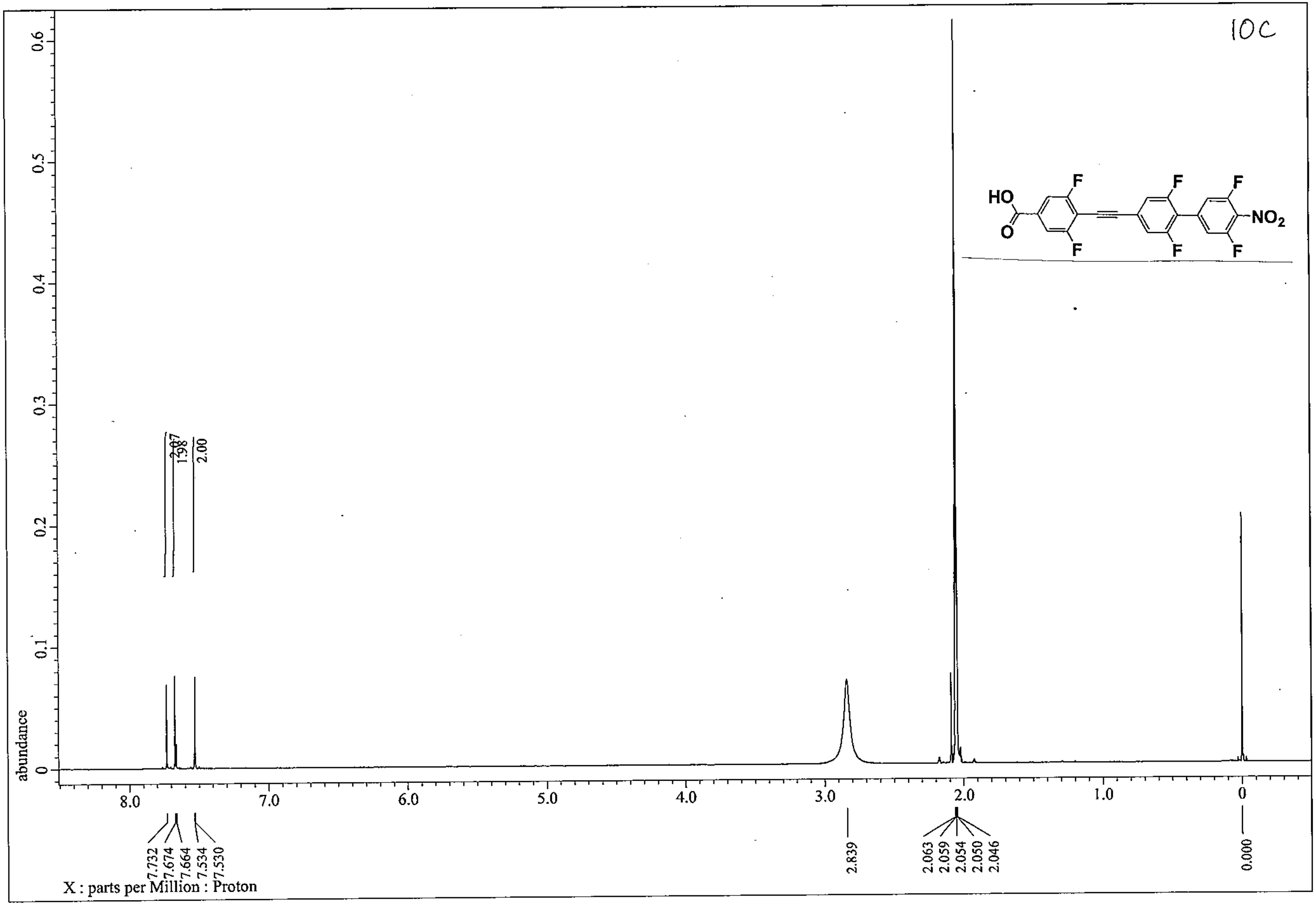
10C
HO
O
F
F
F
F
F
F
NO2
2.07
1.98
2.00
abundance
0
0.1
0.2
0.3
0.4
0.5
0.6
8.0
7.0
6.0
5.0
4.0
3.0
2.0
1.0
0
7.732
7.674
7.664
7.534
7.530
2.839
2.063
2.059
2.054
2.050
2.046
0.000
X : parts per Million : Proton

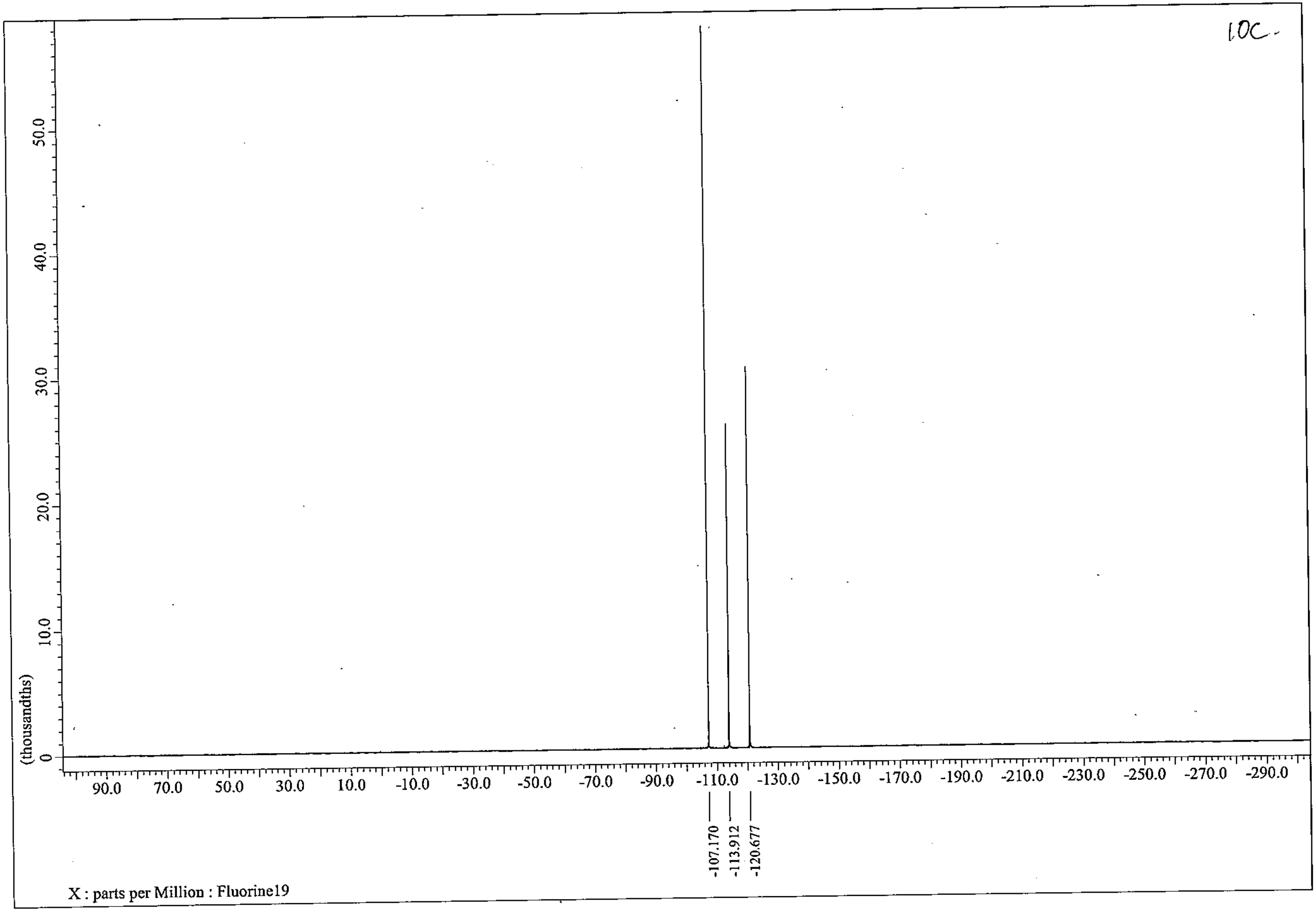
10C
(thousandths)
50.0
40.0
30.0
20.0
10.0
0
90.0
70.0
50.0
30.0
10.0
-10.0
-30.0
-50.0
-70.0
-90.0
-110.0
-130.0
-150.0
-170.0
-190.0
-210.0
-230.0
-250.0
-270.0
-290.0
-107.170
-113.912
-120.677
X : parts per Million : Fluorine19

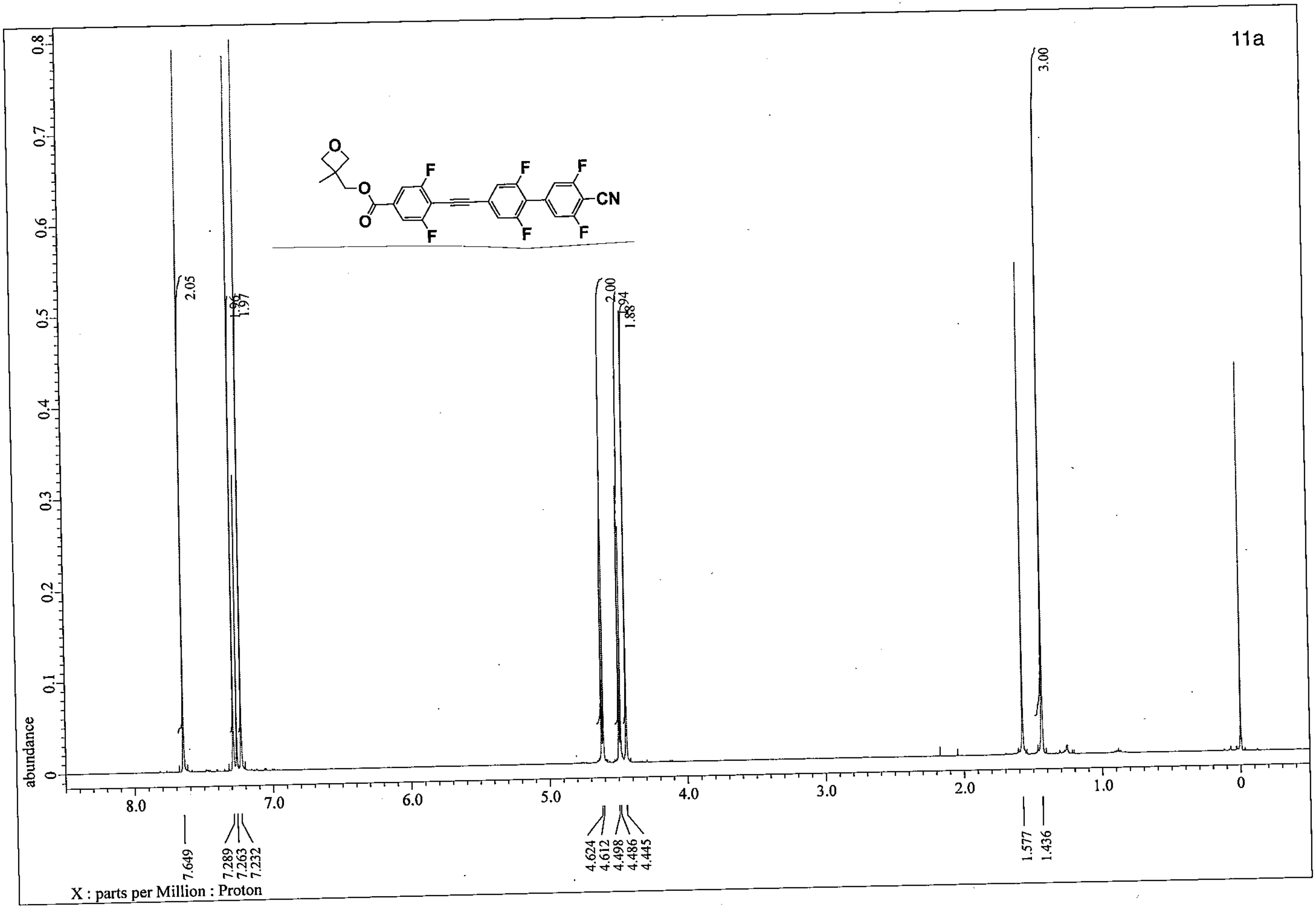
11a
F
F
F
F
F
F
O
O
O
CN
abundance
X : parts per Million : Proton
2.05
1.96
1.97
2.00
1.94
1.88
3.00
7.649
7.289
7.263
7.232
4.624
4.612
4.498
4.486
4.445
1.577
1.436
8.0
7.0
6.0
5.0
4.0
3.0
2.0
1.0
0
0.8
0.7
0.6
0.5
0.4
0.3
0.2
0.1
0

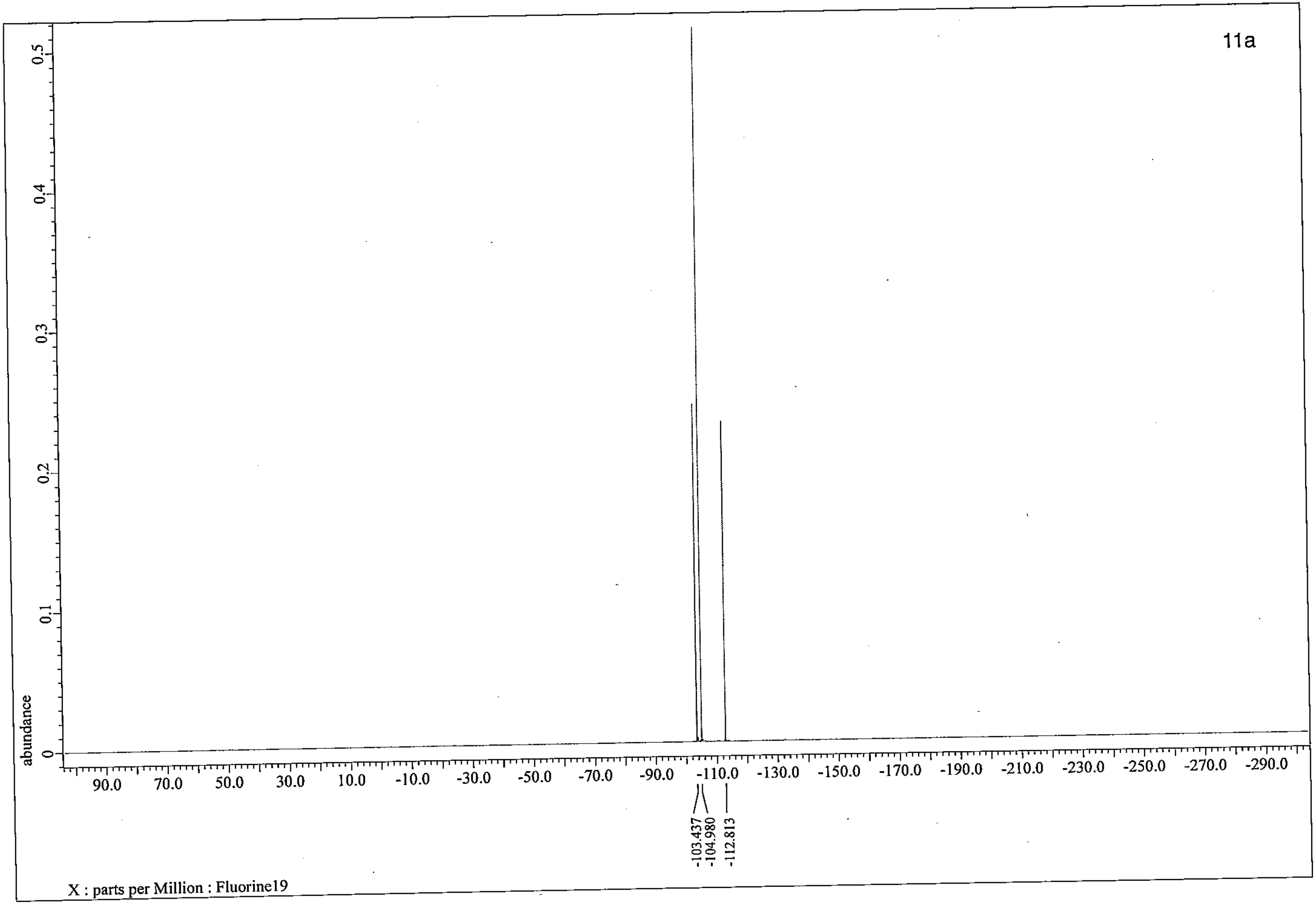

11a
abundance
0
0.1
0.2
0.3
0.4
0.5
90.0
70.0
50.0
30.0
10.0
-10.0
-30.0
-50.0
-70.0
-90.0
-110.0
-130.0
-150.0
-170.0
-190.0
-210.0
-230.0
-250.0
-270.0
-290.0
-103.437
-104.980
-112.813
X : parts per Million : Fluorine19

11b

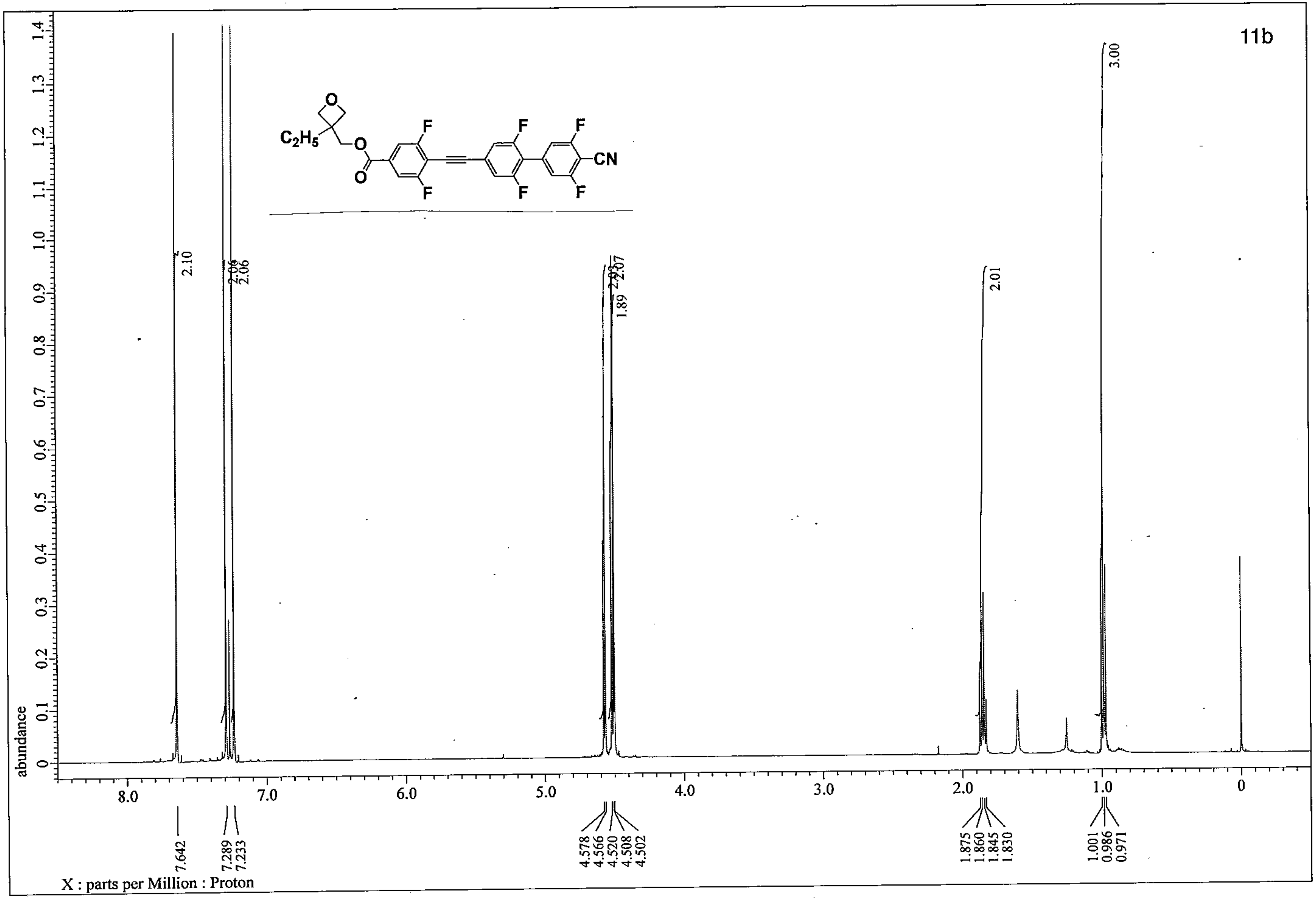

C_2H_5
O
F
CN
abundance
X : parts per Million : Proton
2.10
2.06
2.06
2.03
2.07
1.89
2.01
3.00
7.642
7.289
7.233
4.578
4.566
4.520
4.508
4.502
1.875
1.860
1.845
1.830
1.001
0.986
0.971

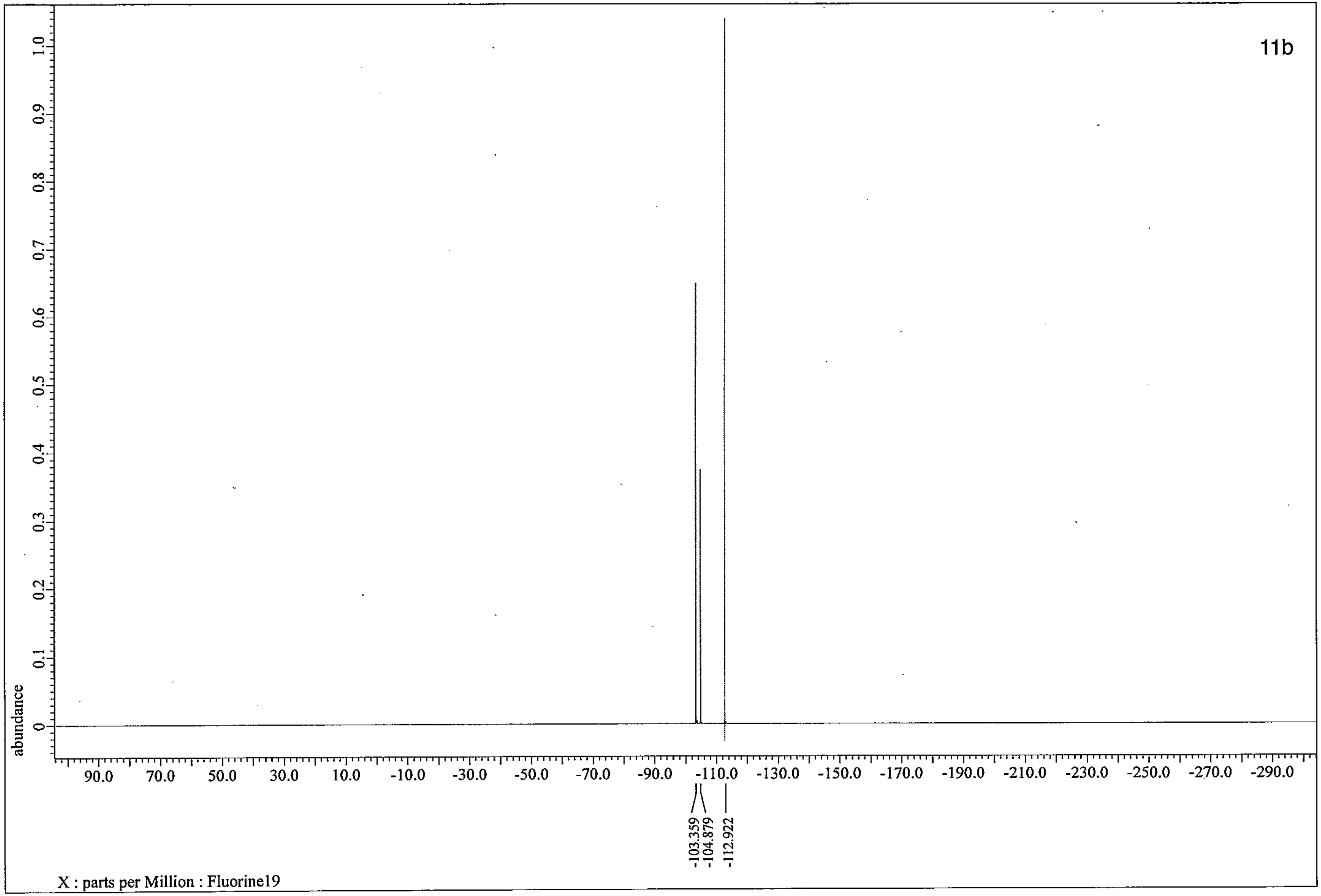
11b
abundance
0
0.1
0.2
0.3
0.4
0.5
0.6
0.7
0.8
0.9
1.0
90.0
70.0
50.0
30.0
10.0
-10.0
-30.0
-50.0
-70.0
-90.0
-110.0
-130.0
-150.0
-170.0
-190.0
-210.0
-230.0
-250.0
-270.0
-290.0
-103.359
-104.879
-112.922
X : parts per Million : Fluorine19

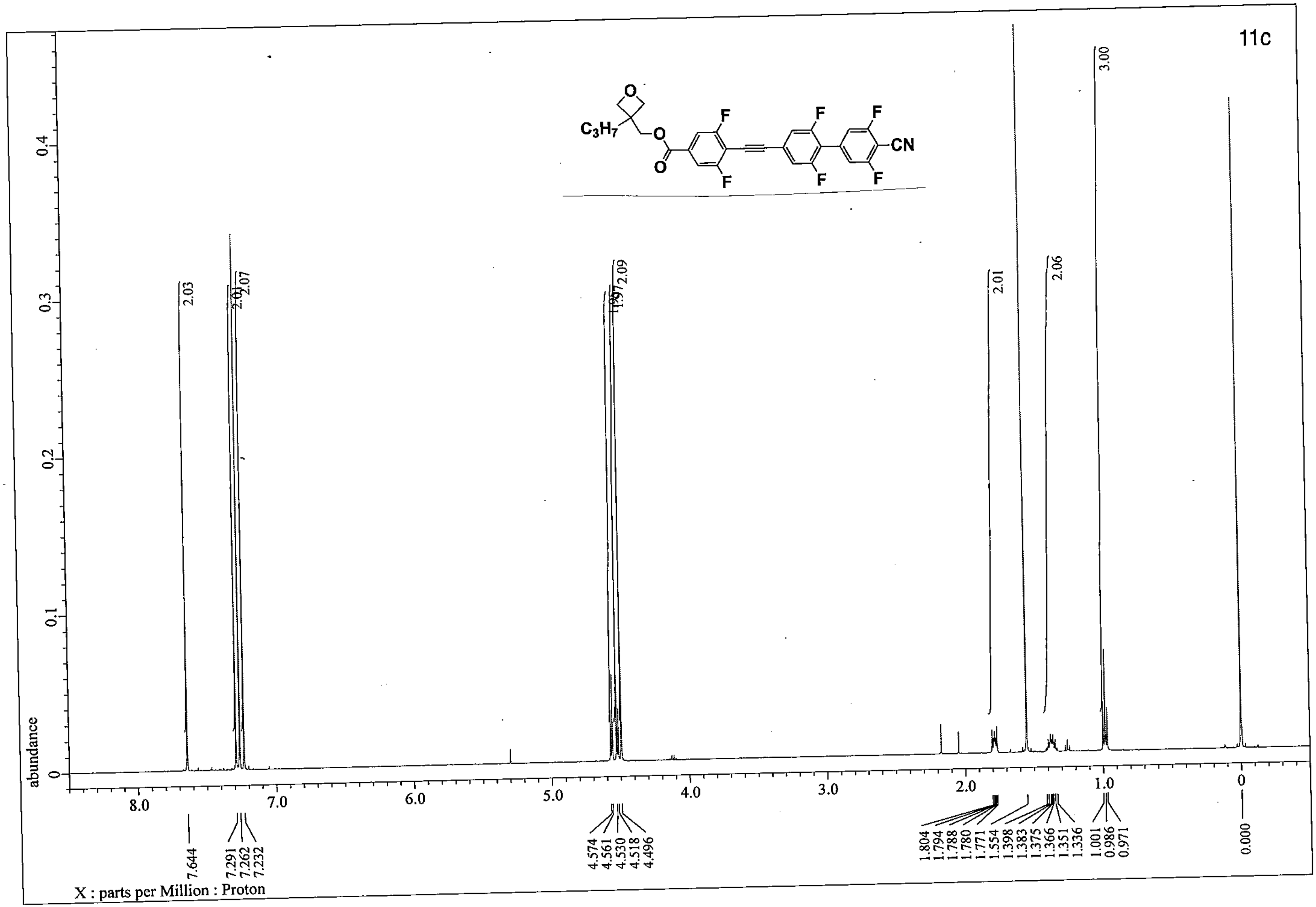

11c
C3H7
O
F
CN
abundance
0.4
0.3
0.2
0.1
0
2.03
2.01
2.07
1.97
1.97
2.09
2.01
2.06
3.00
8.0
7.0
6.0
5.0
4.0
3.0
2.0
1.0
0
7.644
7.291
7.262
7.232
4.574
4.561
4.530
4.518
4.496
1.804
1.794
1.788
1.780
1.771
1.554
1.398
1.383
1.375
1.366
1.351
1.336
1.001
0.986
0.971
0.000
X : parts per Million : Proton

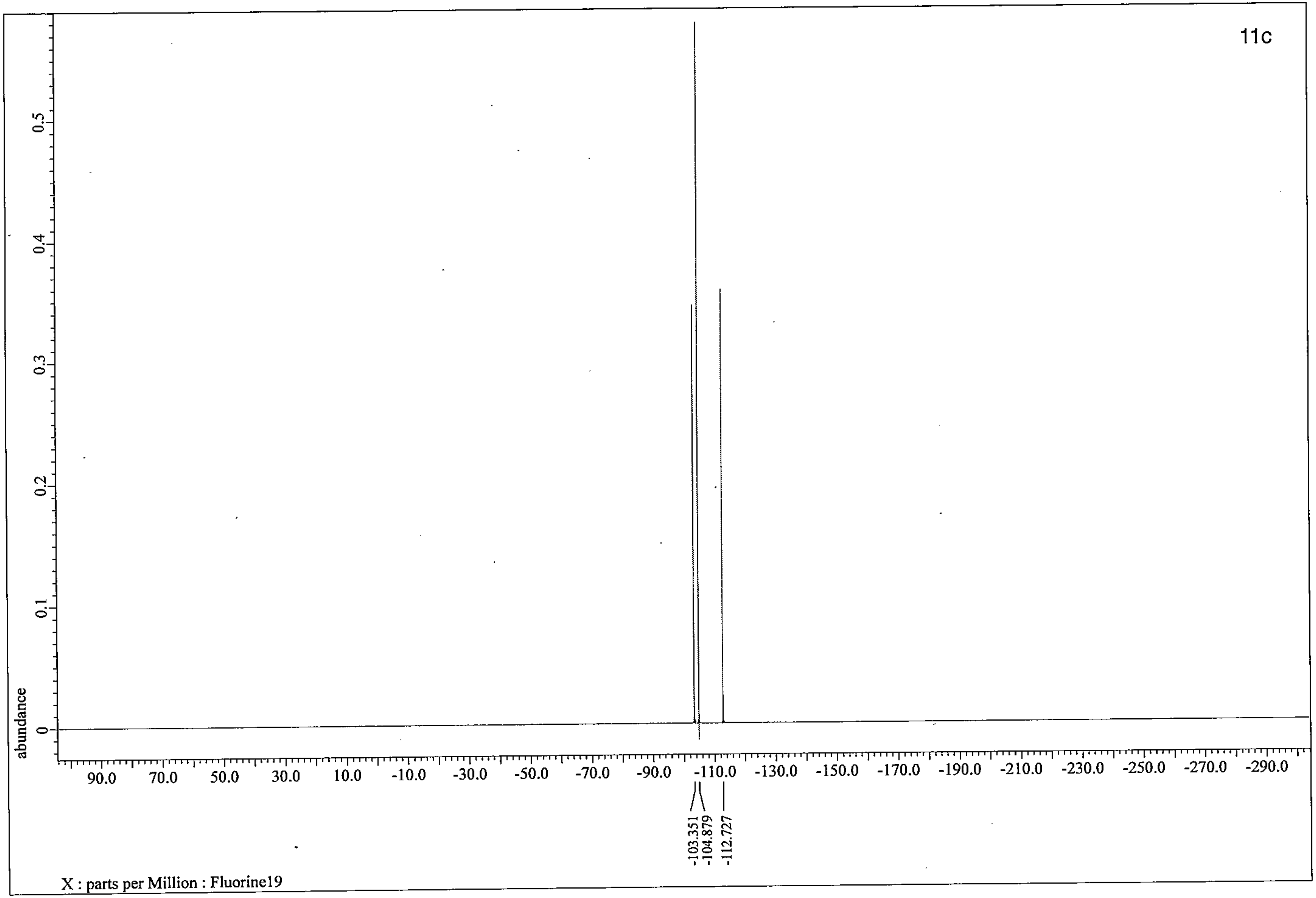
11c
abundance
0
0.1
0.2
0.3
0.4
0.5
90.0 70.0 50.0 30.0 10.0 -10.0 -30.0 -50.0 -70.0 -90.0 -110.0 -130.0 -150.0 -170.0 -190.0 -210.0 -230.0 -250.0 -270.0 -290.0
-103.351
-104.879
-112.727
X : parts per Million : Fluorine19

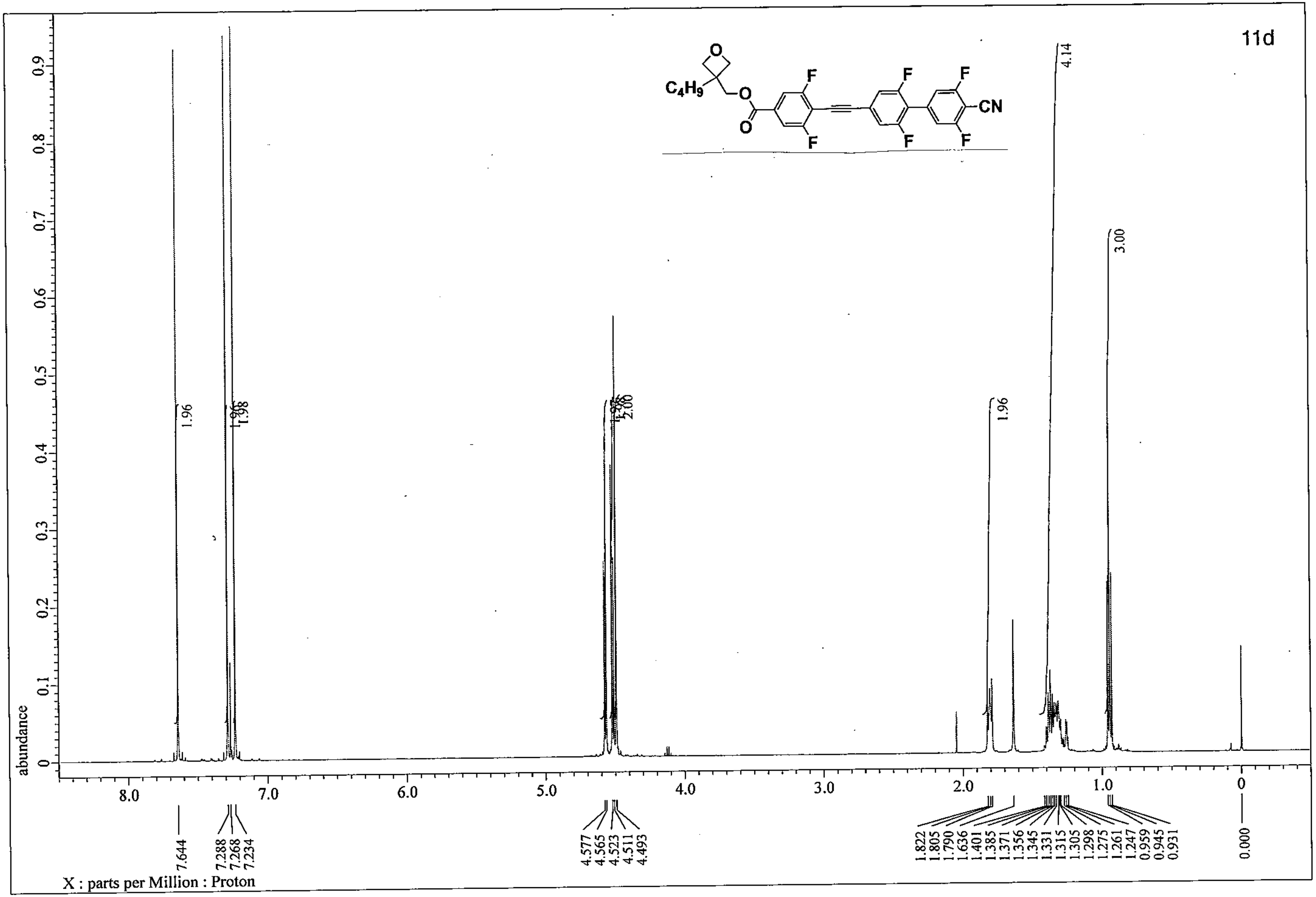

11d
C4H9
O
F
CN
abundance
0.9
0.8
0.7
0.6
0.5
0.4
0.3
0.2
0.1
0
1.96
1.96
1.98
2.00
1.96
4.14
3.00
8.0
7.0
6.0
5.0
4.0
3.0
2.0
1.0
0
7.644
7.288
7.268
7.234
4.577
4.565
4.523
4.511
4.493
1.822
1.805
1.790
1.636
1.401
1.385
1.371
1.356
1.345
1.331
1.315
1.305
1.298
1.275
1.261
1.247
0.959
0.945
0.931
0.000
X : parts per Million : Proton

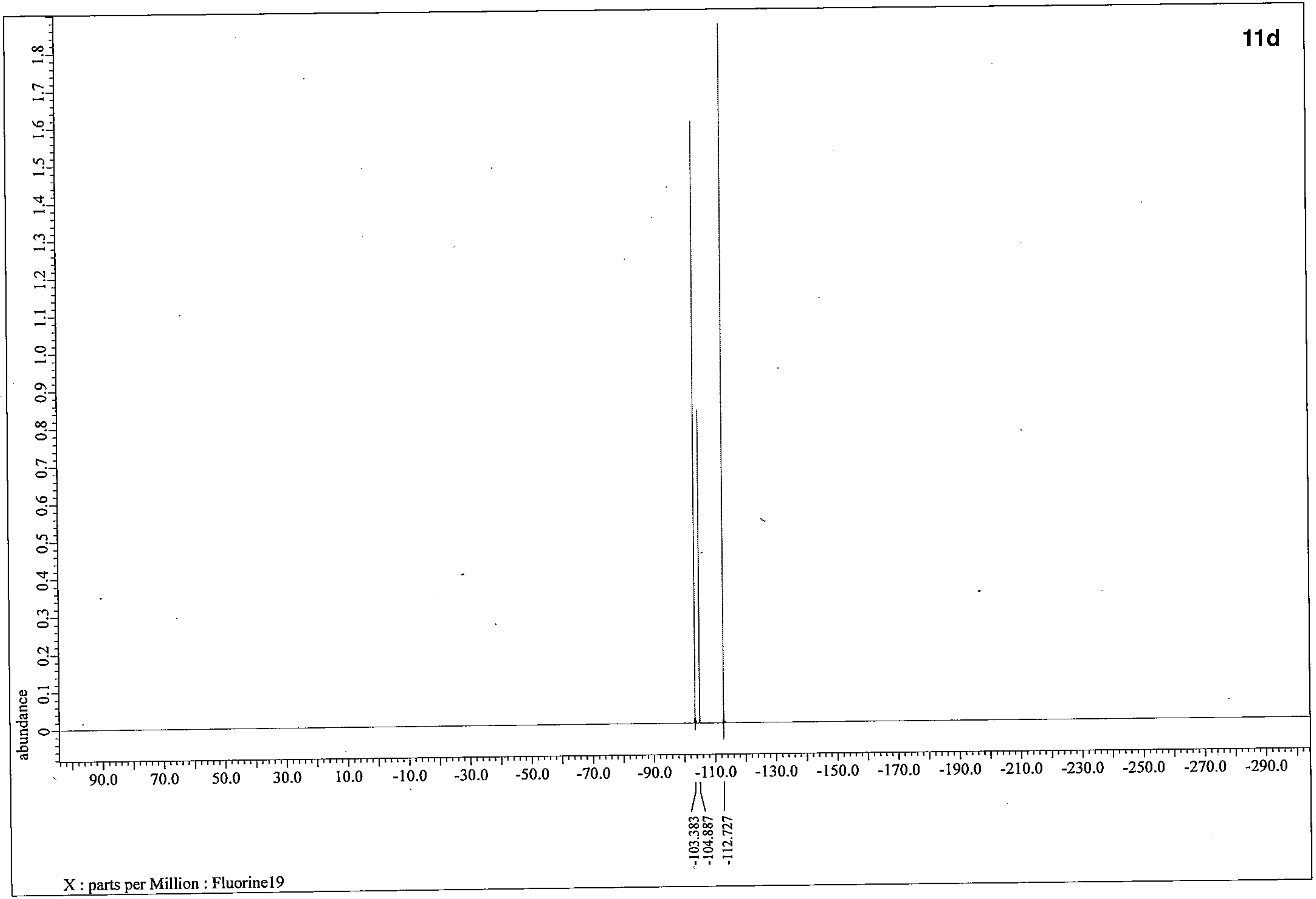
11d
abundance
0
0.1
0.2
0.3
0.4
0.5
0.6
0.7
0.8
0.9
1.0
1.1
1.2
1.3
1.4
1.5
1.6
1.7
1.8
90.0
70.0
50.0
30.0
10.0
-10.0
-30.0
-50.0
-70.0
-90.0
-110.0
-130.0
-150.0
-170.0
-190.0
-210.0
-230.0
-250.0
-270.0
-290.0
-103.383
-104.887
-112.727
X : parts per Million : Fluorine19

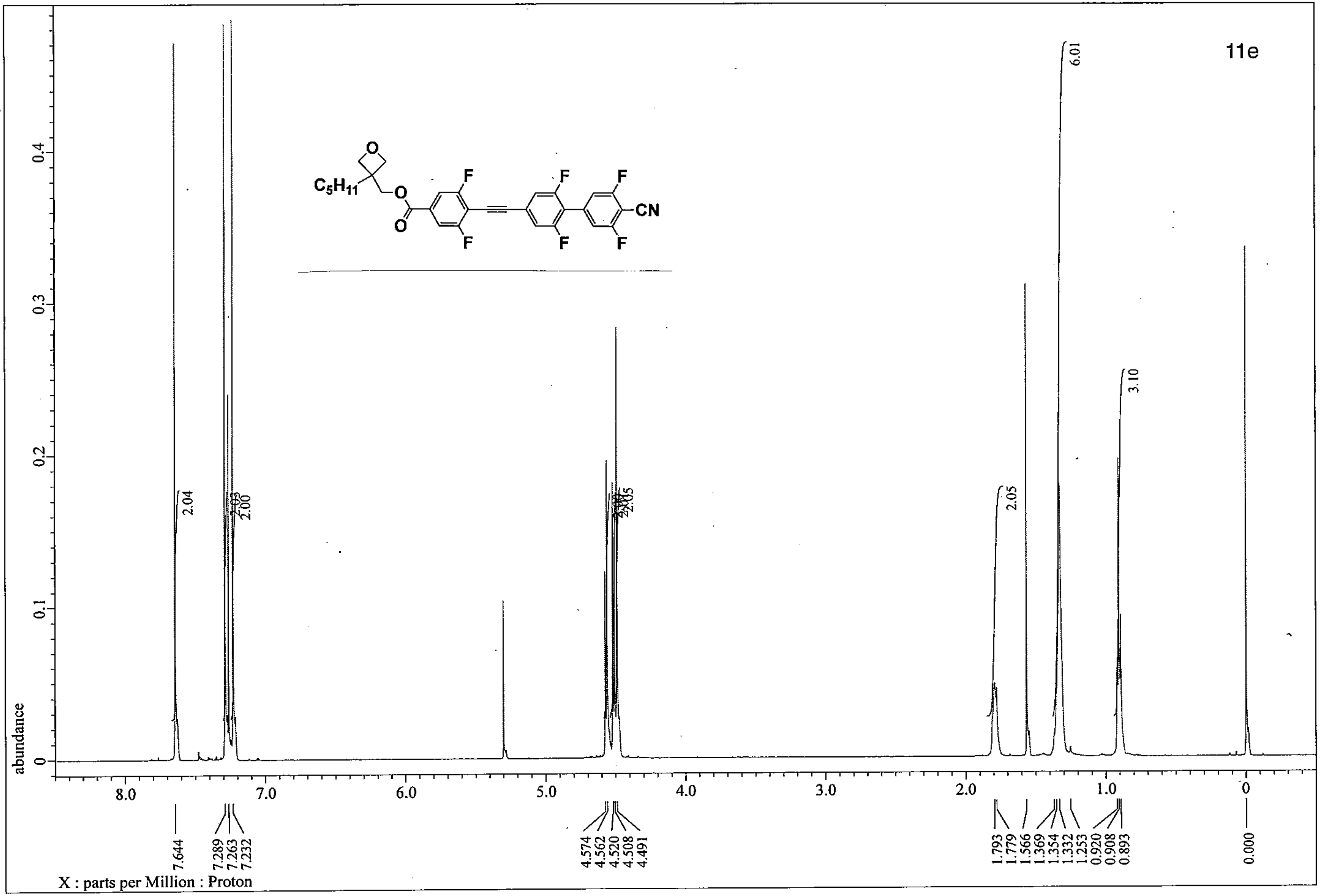
11e
C_5H_{11}
O
F
CN
abundance
0.4
0.3
0.2
0.1
0
2.04
2.03
2.00
2.05
6.01
3.10
8.0
7.0
6.0
5.0
4.0
3.0
2.0
1.0
0
7.644
7.289
7.263
7.232
4.574
4.562
4.520
4.508
4.491
1.793
1.779
1.566
1.369
1.354
1.332
1.253
0.920
0.908
0.893
0.000
X : parts per Million : Proton

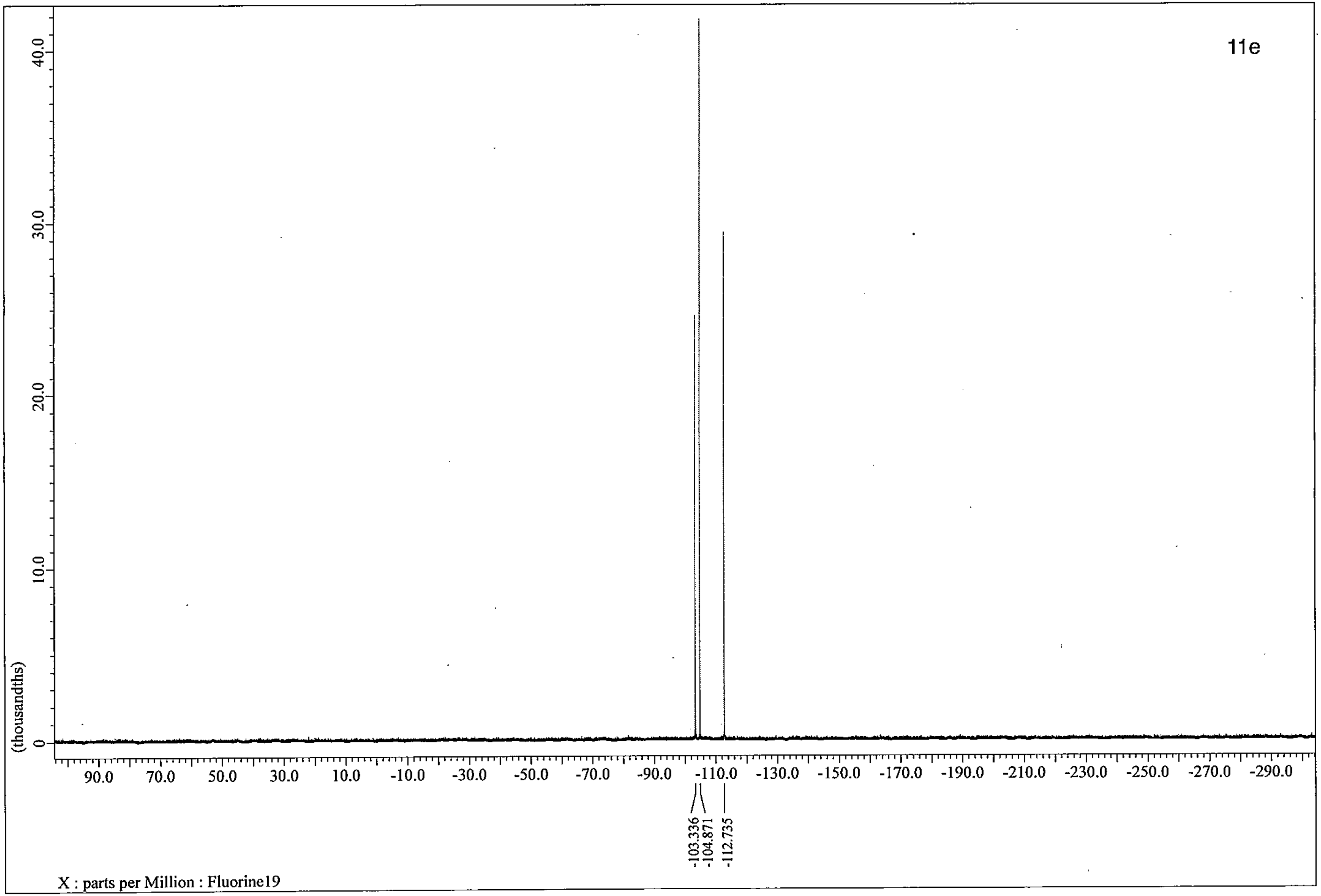
11e
(thousandths)
40.0
30.0
20.0
10.0
0
90.0
70.0
50.0
30.0
10.0
-10.0
-30.0
-50.0
-70.0
-90.0
-110.0
-130.0
-150.0
-170.0
-190.0
-210.0
-230.0
-250.0
-270.0
-290.0
-103.336
-104.871
-112.735
X : parts per Million : Fluorine19

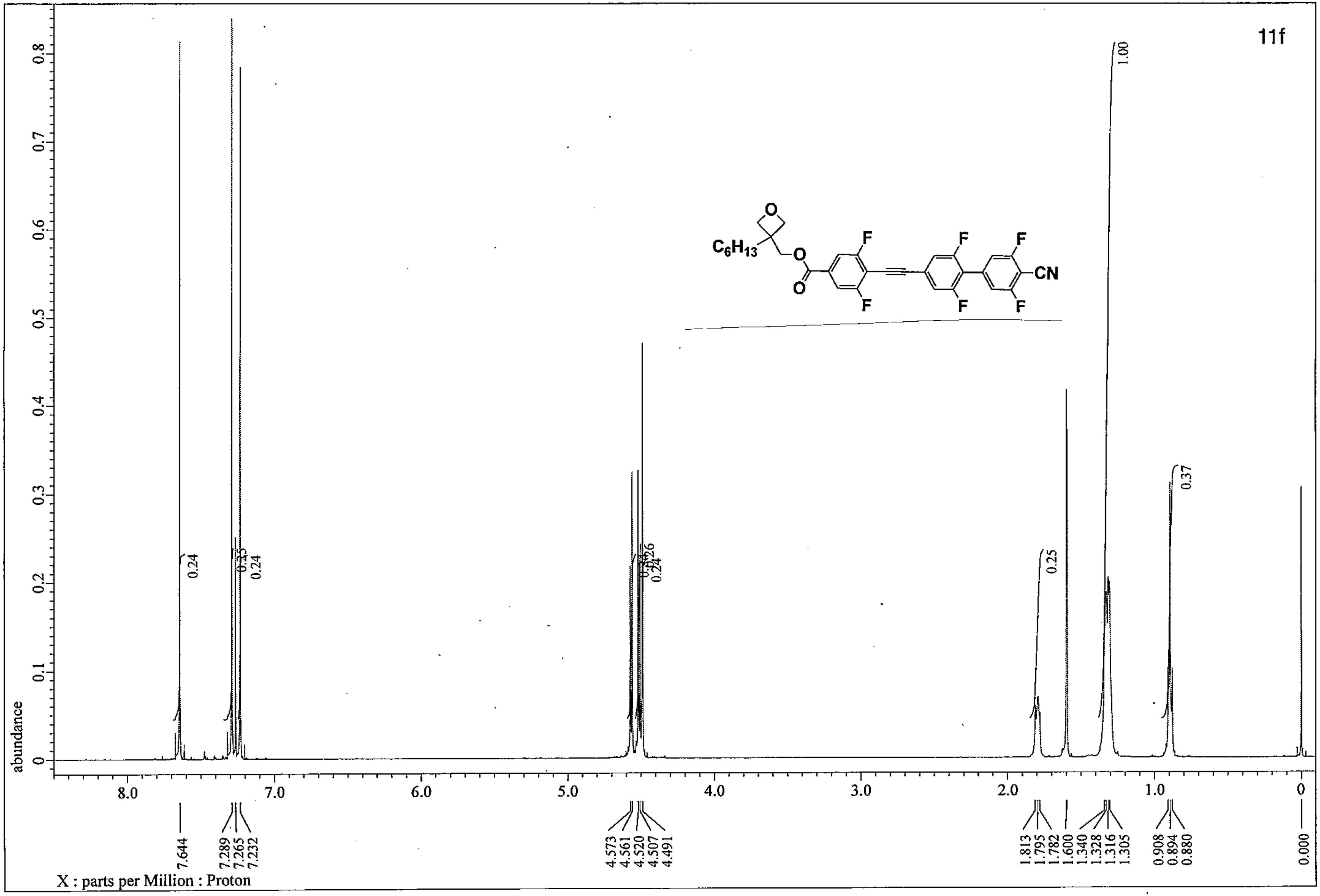

11f
C6H13
O
F
CN
abundance
X : parts per Million : Proton
8.0
7.0
6.0
5.0
4.0
3.0
2.0
1.0
0
0.24
0.25
0.24
0.26
0.24
0.25
1.00
0.37
7.644
7.289
7.265
7.232
4.573
4.561
4.520
4.507
4.491
1.813
1.795
1.782
1.600
1.340
1.328
1.316
1.305
0.908
0.894
0.880
0.000

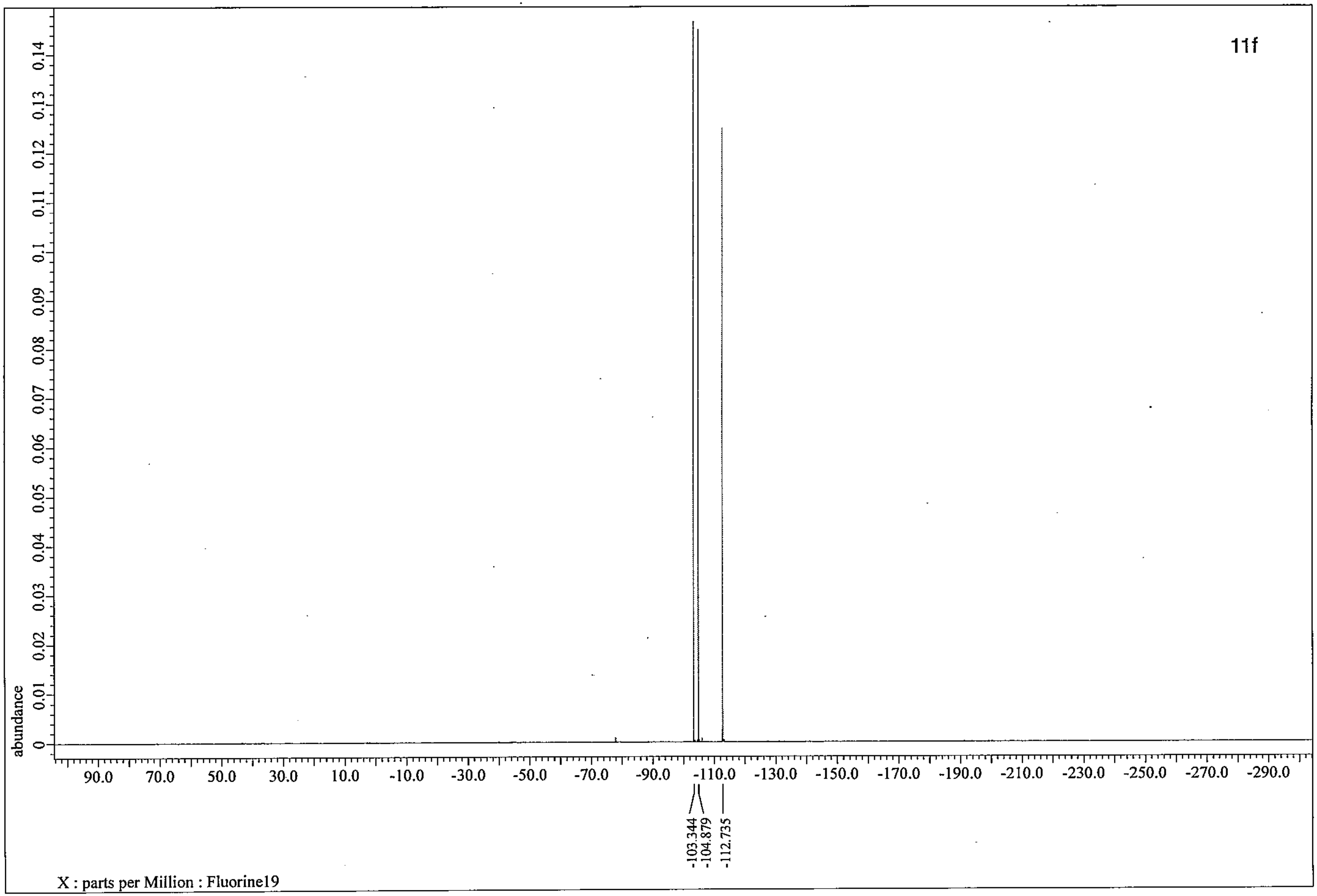
11f
abundance
0
0.01
0.02
0.03
0.04
0.05
0.06
0.07
0.08
0.09
0.1
0.11
0.12
0.13
0.14
90.0
70.0
50.0
30.0
10.0
-10.0
-30.0
-50.0
-70.0
-90.0
-110.0
-130.0
-150.0
-170.0
-190.0
-210.0
-230.0
-250.0
-270.0
-290.0
-103.344
-104.879
-112.735
X : parts per Million : Fluorine19

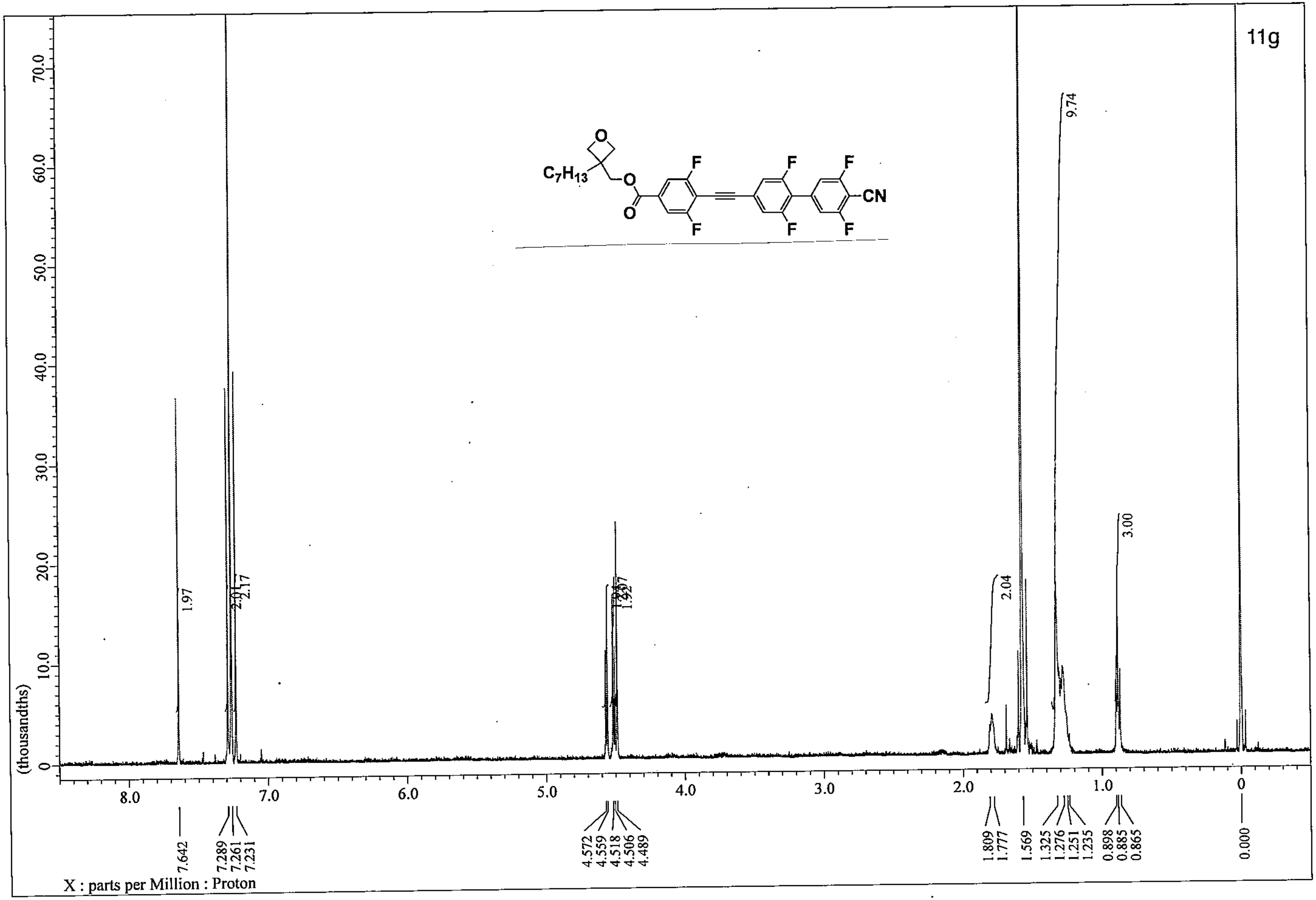
11g
C7H13
O
F
CN
(thousandths)
X : parts per Million : Proton
1.97
2.17
2.04
9.74
3.00
7.642
7.289
7.261
7.231
4.572
4.559
4.518
4.506
4.489
1.809
1.777
1.569
1.325
1.276
1.251
1.235
0.898
0.885
0.865
0.000

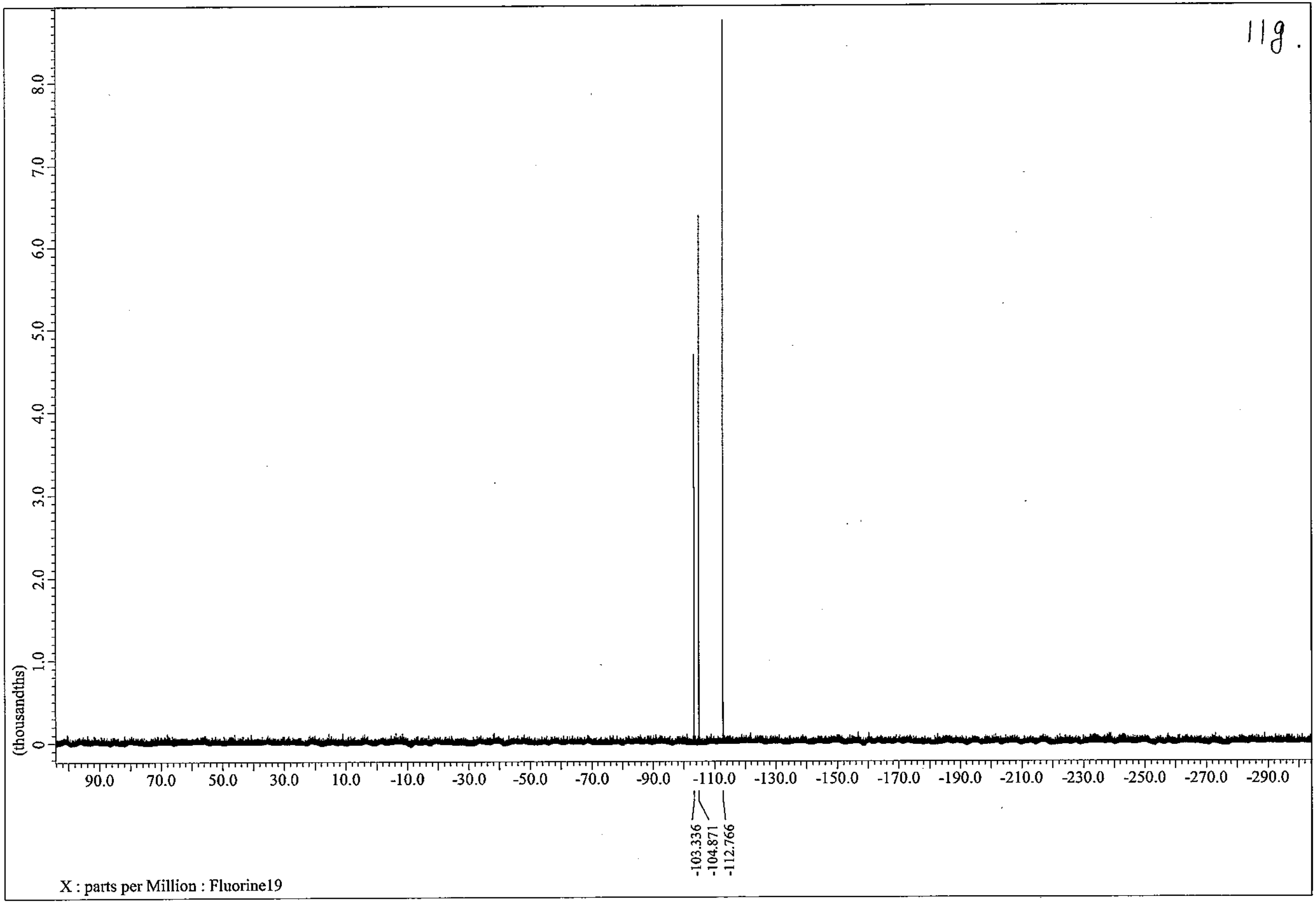

11g.
(thousandths)
0
1.0
2.0
3.0
4.0
5.0
6.0
7.0
8.0
90.0
70.0
50.0
30.0
10.0
-10.0
-30.0
-50.0
-70.0
-90.0
-110.0
-130.0
-150.0
-170.0
-190.0
-210.0
-230.0
-250.0
-270.0
-290.0
-103.336
-104.871
-112.766
X : parts per Million : Fluorine19

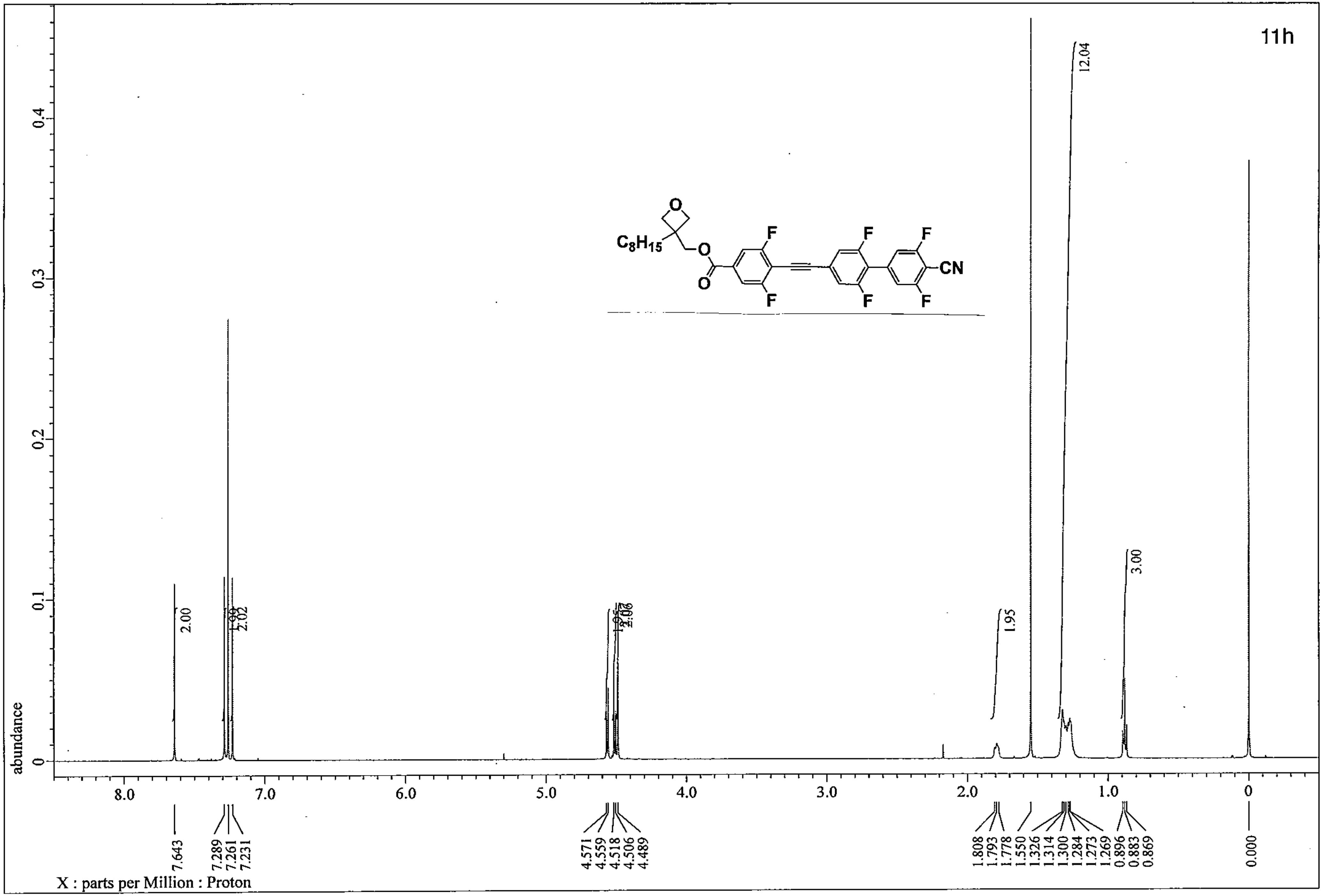

11h
C8H15
O
F
CN
abundance
0.4
0.3
0.2
0.1
0
2.00
1.99
2.02
1.95
12.04
3.00
8.0
7.0
6.0
5.0
4.0
3.0
2.0
1.0
0
7.643
7.289
7.261
7.231
4.571
4.559
4.518
4.506
4.489
1.808
1.793
1.778
1.550
1.326
1.314
1.300
1.284
1.273
1.269
0.896
0.883
0.869
0.000
X : parts per Million : Proton

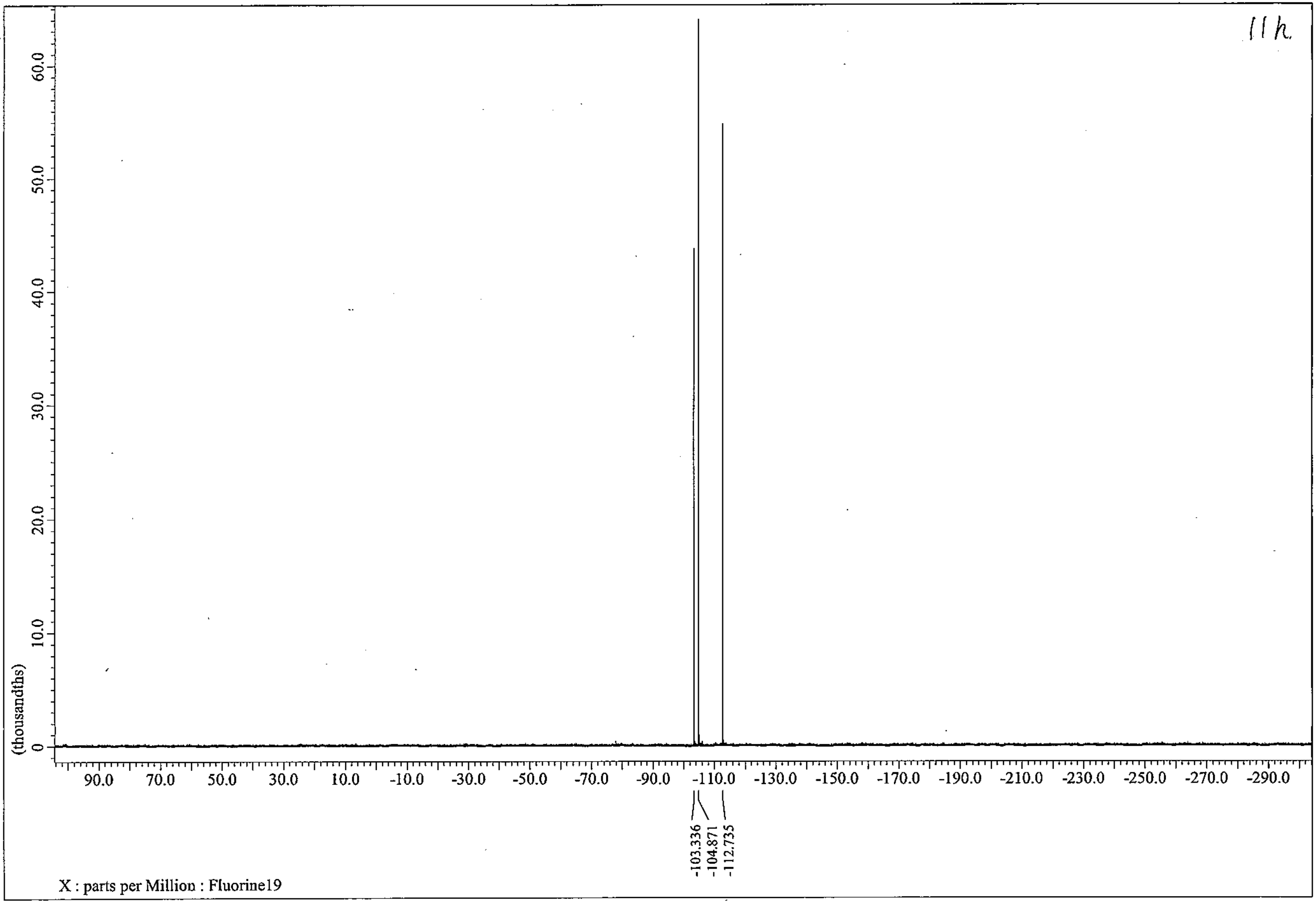
11h
(thousandths)
60.0
50.0
40.0
30.0
20.0
10.0
0
90.0
70.0
50.0
30.0
10.0
-10.0
-30.0
-50.0
-70.0
-90.0
-110.0
-130.0
-150.0
-170.0
-190.0
-210.0
-230.0
-250.0
-270.0
-290.0
-103.336
-104.871
-112.735
X : parts per Million : Fluorine19

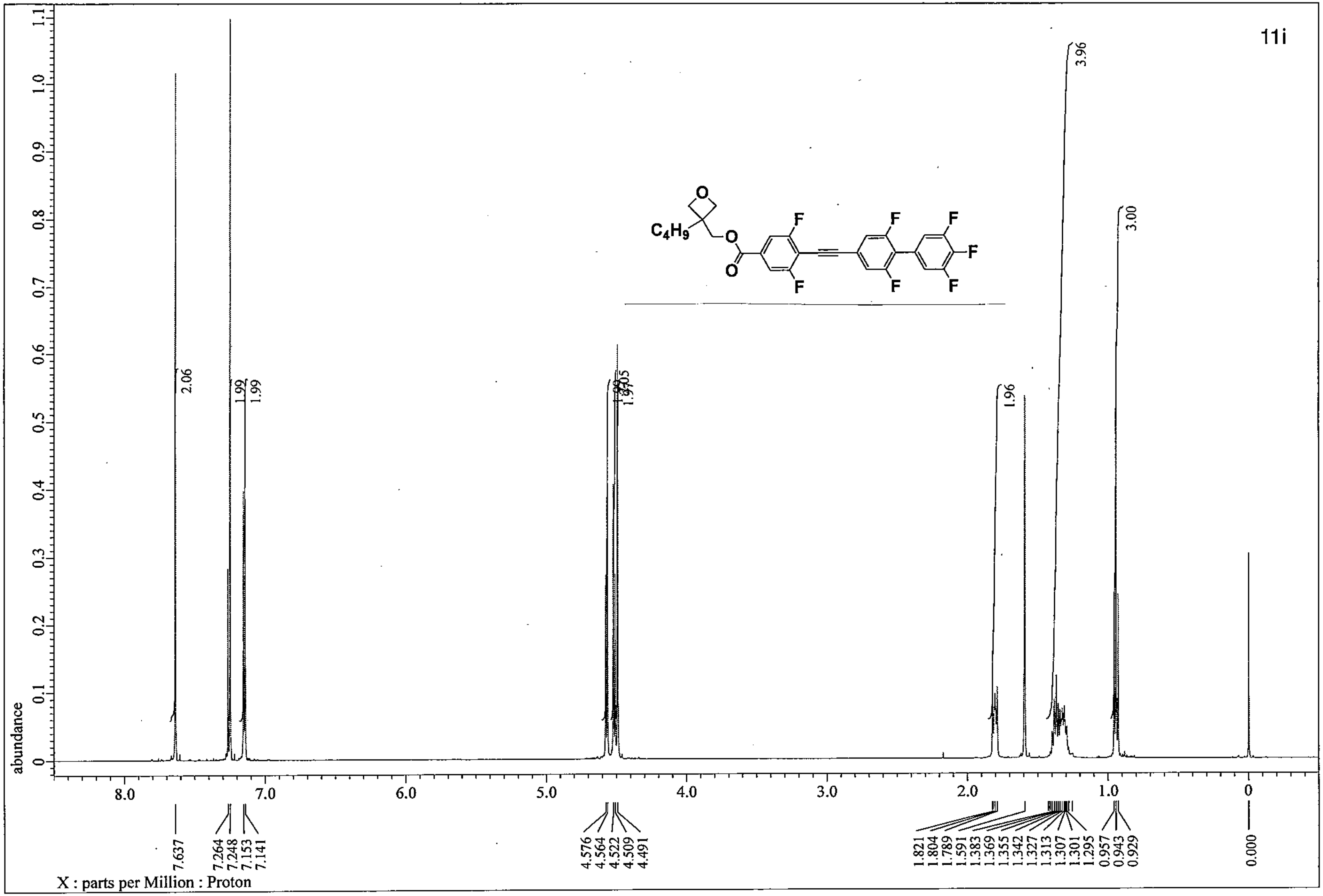

11i
C4H9
O
F
abundance
X : parts per Million : Proton
8.0
7.0
6.0
5.0
4.0
3.0
2.0
1.0
0
2.06
1.99
1.99
1.96
3.96
3.00
7.637
7.264
7.248
7.153
7.141
4.576
4.564
4.522
4.509
4.491
1.821
1.804
1.789
1.591
1.383
1.369
1.355
1.342
1.327
1.313
1.307
1.301
1.295
0.957
0.943
0.929
0.000

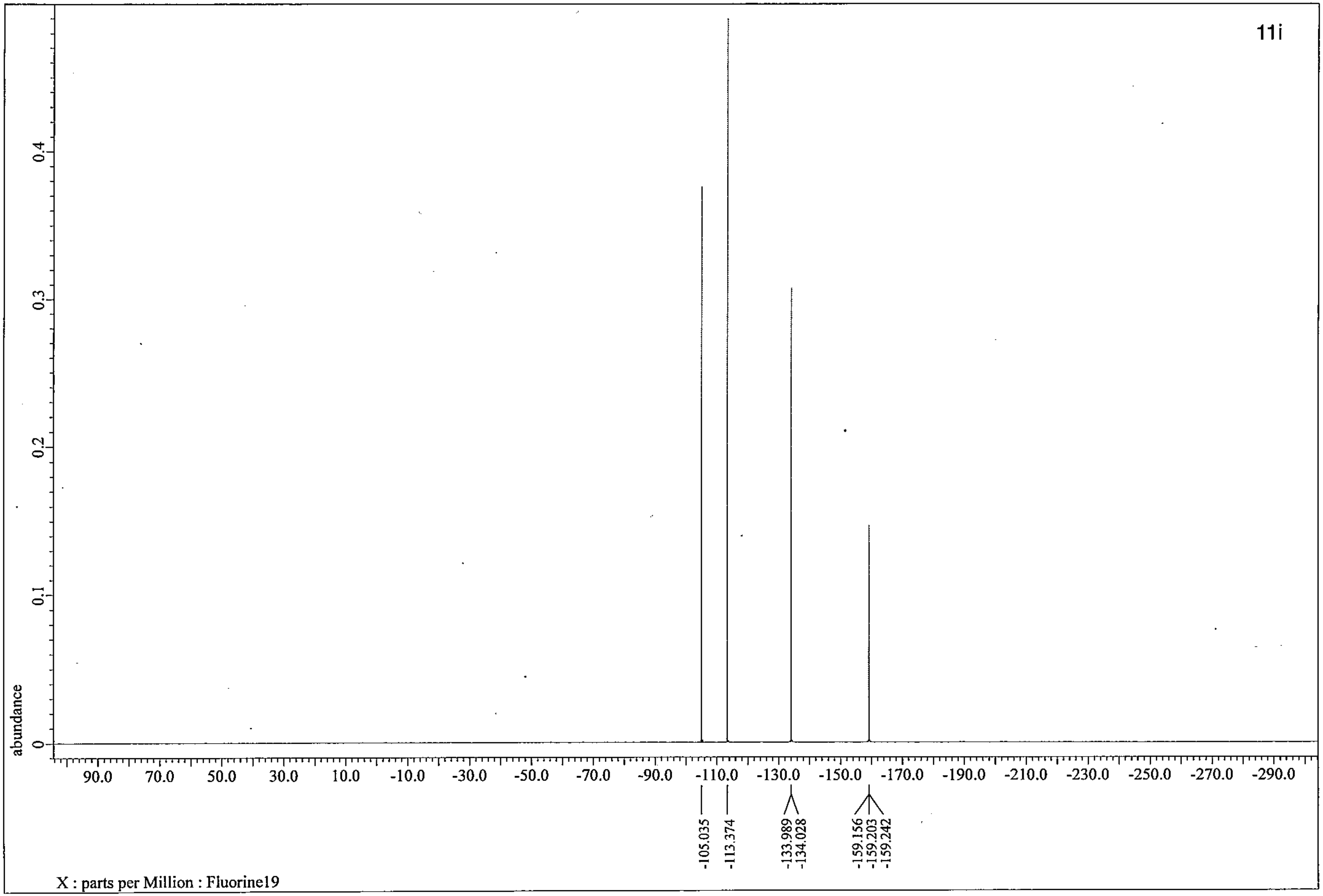

11i
abundance
0.4
0.3
0.2
0.1
0
90.0
70.0
50.0
30.0
10.0
-10.0
-30.0
-50.0
-70.0
-90.0
-110.0
-130.0
-150.0
-170.0
-190.0
-210.0
-230.0
-250.0
-270.0
-290.0
-105.035
-113.374
-133.989
-134.028
-159.156
-159.203
-159.242
X : parts per Million : Fluorine19

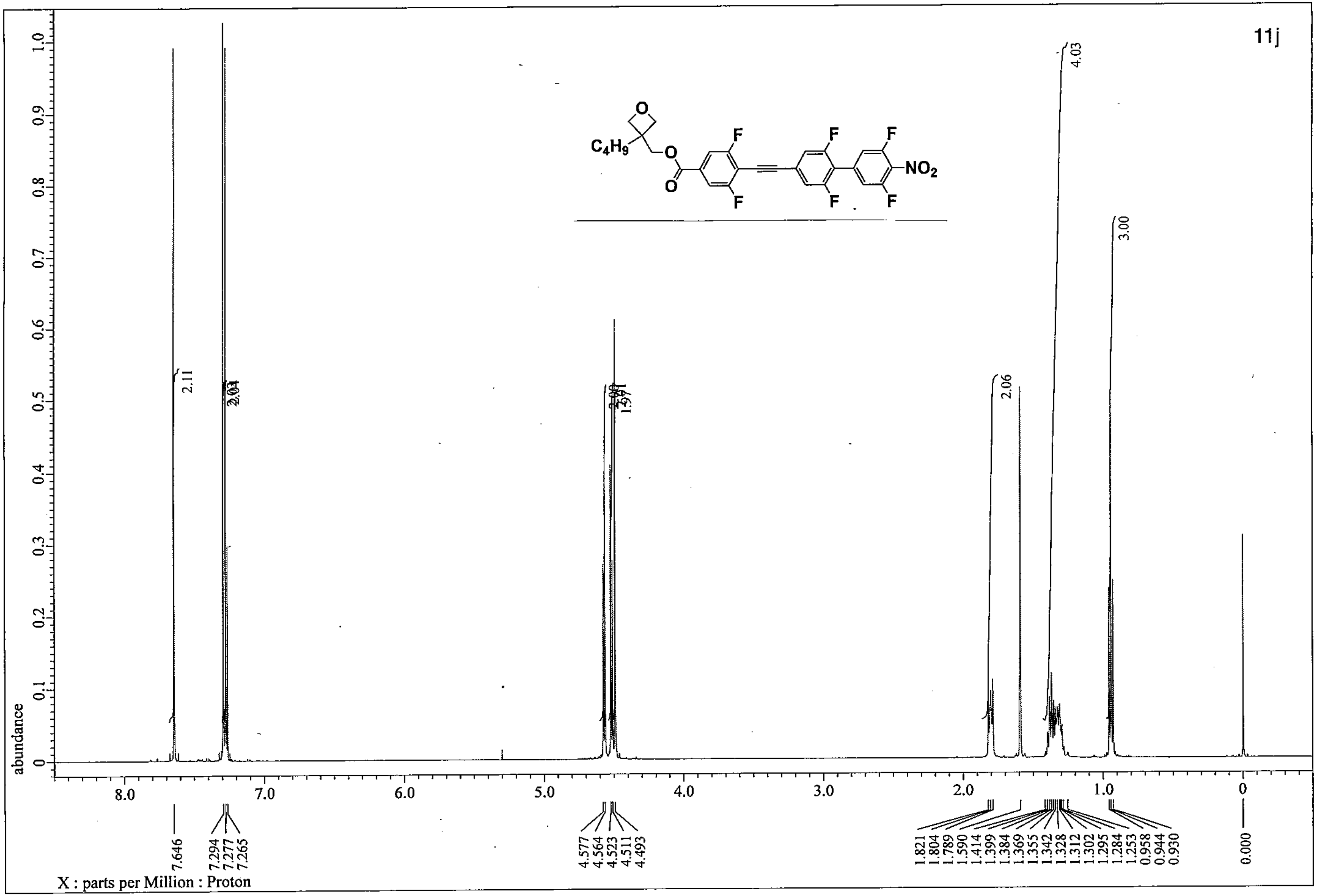
11j
C4H9
O
F
NO2
abundance
X : parts per Million : Proton
8.0
7.0
6.0
5.0
4.0
3.0
2.0
1.0
0
2.11
4.03
3.00
2.06
1.97
7.646
7.294
7.277
7.265
4.577
4.564
4.523
4.511
4.493
1.821
1.804
1.789
1.590
1.414
1.399
1.384
1.369
1.355
1.342
1.328
1.312
1.302
1.295
1.284
1.253
0.958
0.944
0.930
0.000

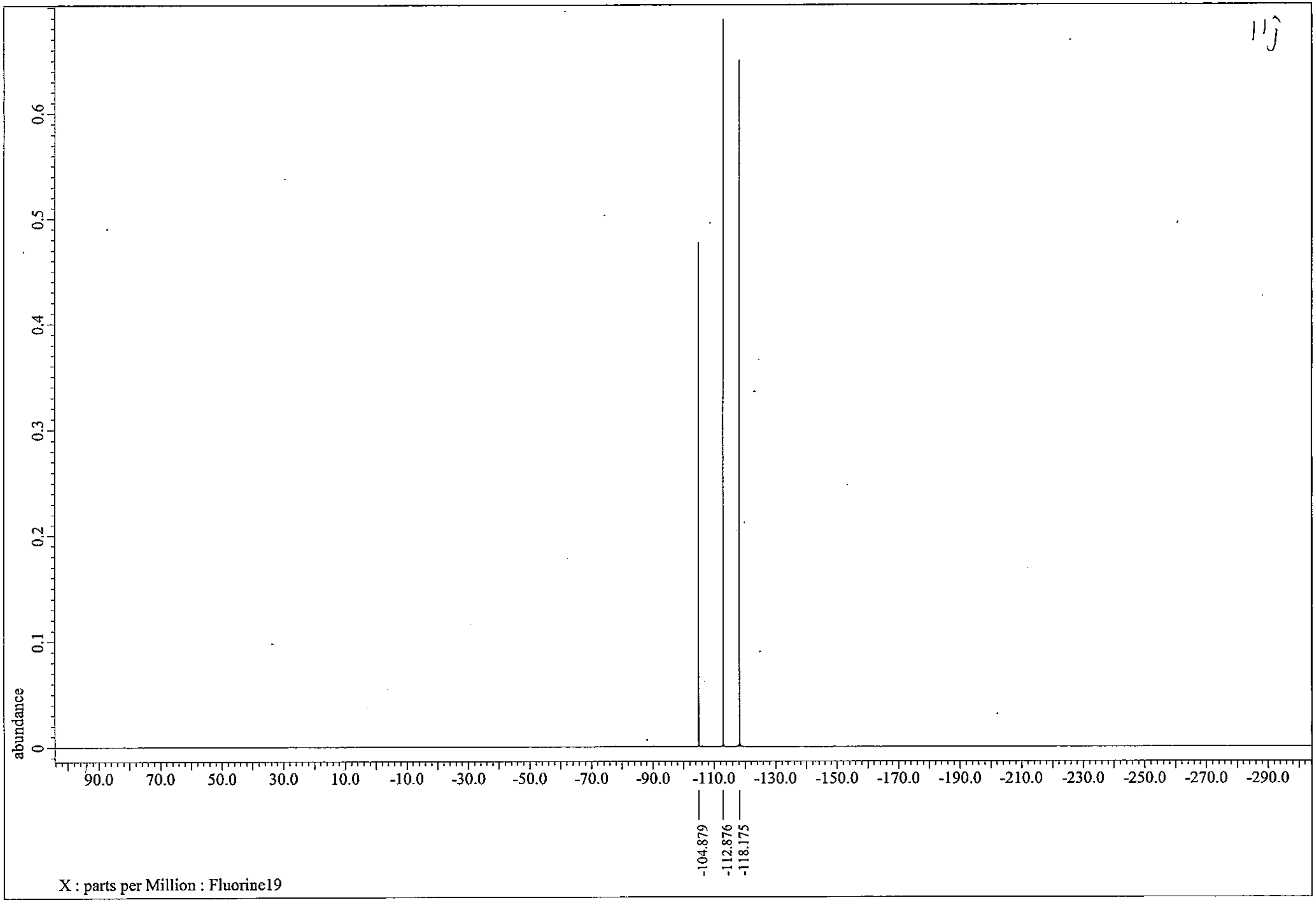

11j
abundance
0.6
0.5
0.4
0.3
0.2
0.1
0
90.0
70.0
50.0
30.0
10.0
-10.0
-30.0
-50.0
-70.0
-90.0
-110.0
-130.0
-150.0
-170.0
-190.0
-210.0
-230.0
-250.0
-270.0
-290.0
-104.879
-112.876
-118.175
X : parts per Million : Fluorine19

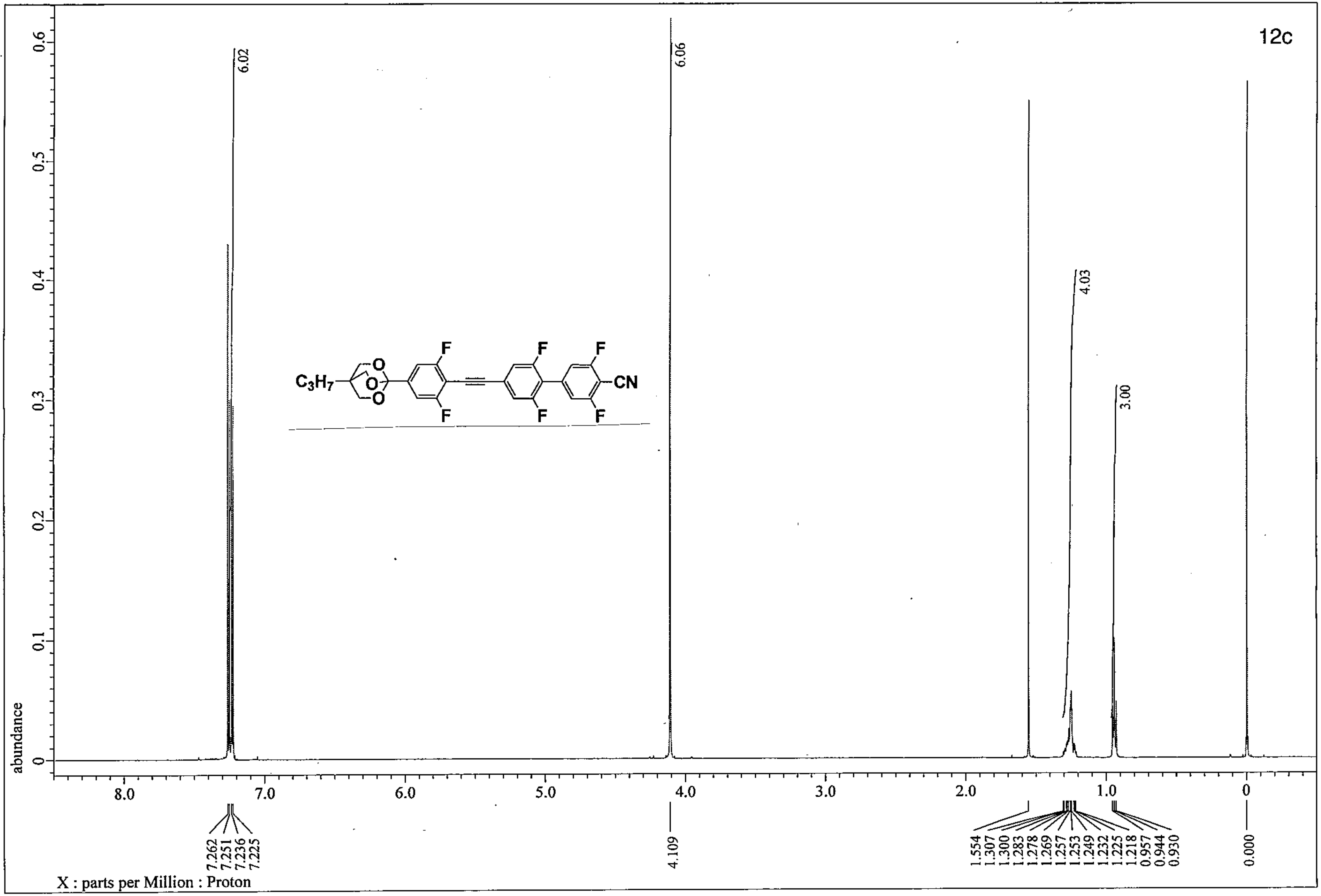
12c
C_3H_7
O
O
O
F
F
F
F
F
F
CN
abundance
0.6
0.5
0.4
0.3
0.2
0.1
0
6.02
6.06
4.03
3.00
8.0
7.0
6.0
5.0
4.0
3.0
2.0
1.0
0
7.262
7.251
7.236
7.225
4.109
1.554
1.307
1.300
1.283
1.278
1.269
1.257
1.253
1.249
1.232
1.225
1.218
0.957
0.944
0.930
0.000
X : parts per Million : Proton

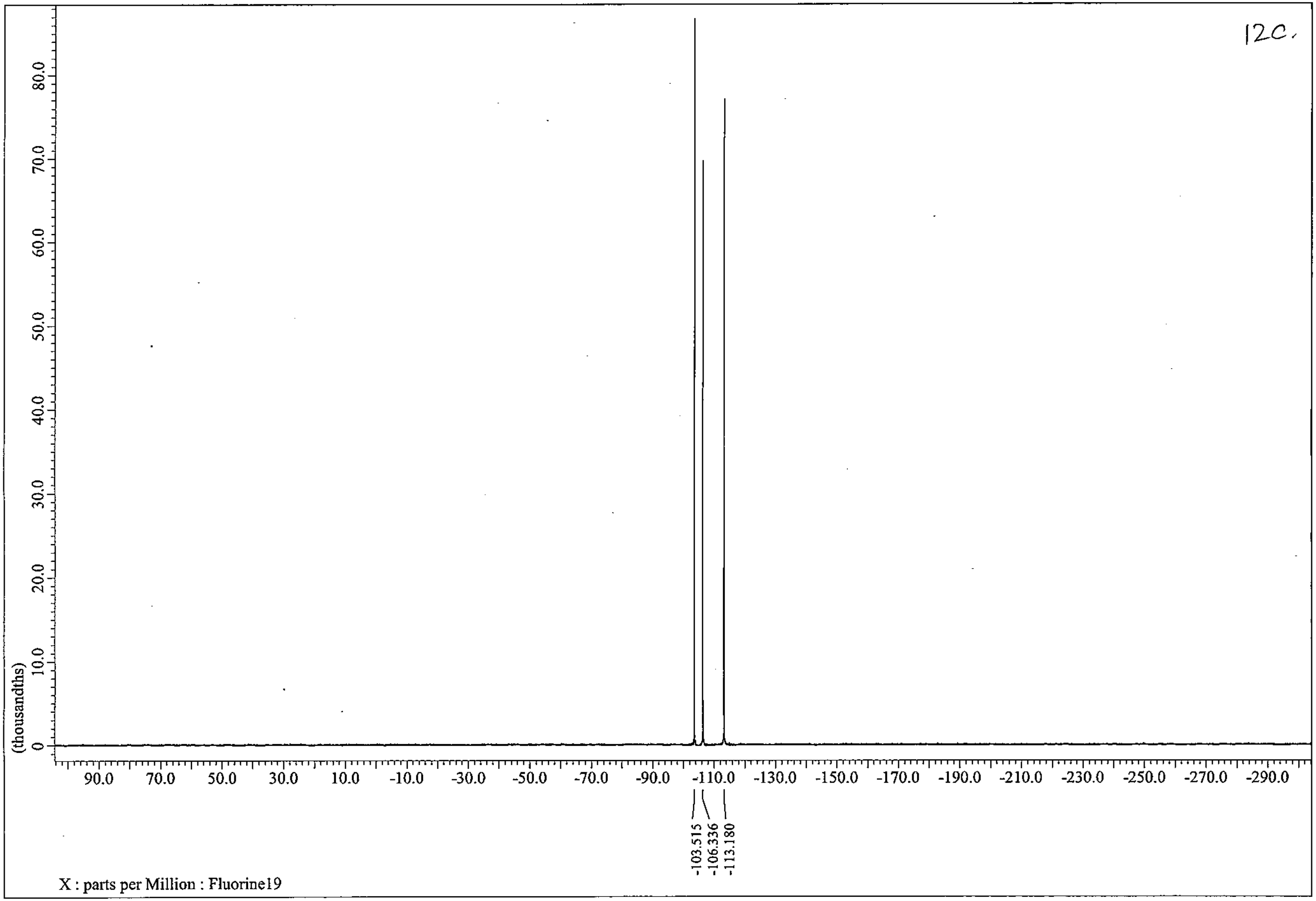

12C.
(thousandths)
80.0
70.0
60.0
50.0
40.0
30.0
20.0
10.0
0
90.0
70.0
50.0
30.0
10.0
-10.0
-30.0
-50.0
-70.0
-90.0
-110.0
-130.0
-150.0
-170.0
-190.0
-210.0
-230.0
-250.0
-270.0
-290.0
-103.515
-106.336
-113.180
X : parts per Million : Fluorine19

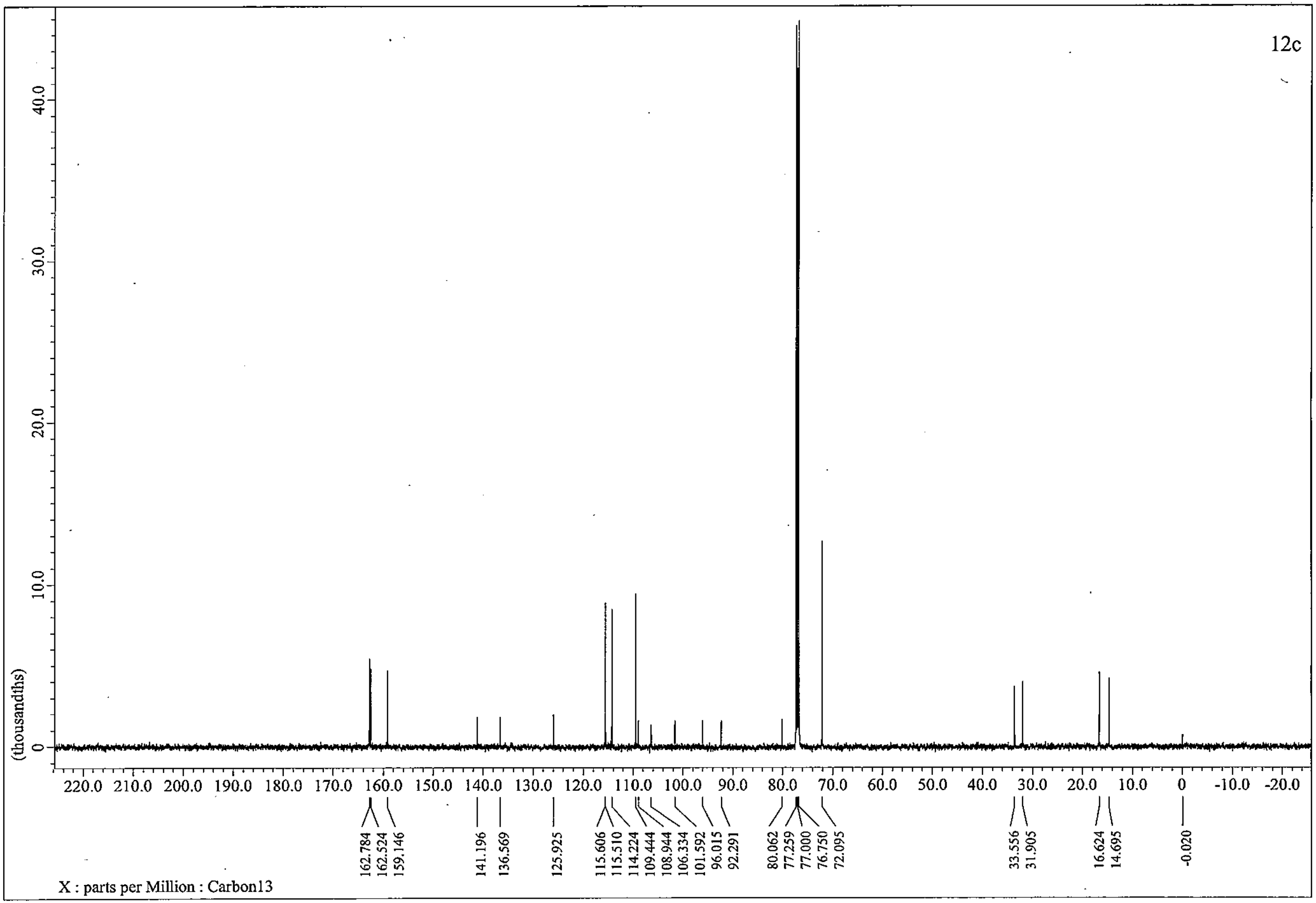
12c
(thousandths)
0
10.0
20.0
30.0
40.0
220.0 210.0 200.0 190.0 180.0 170.0 160.0 150.0 140.0 130.0 120.0 110.0 100.0 90.0 80.0 70.0 60.0 50.0 40.0 30.0 20.0 10.0 0 -10.0 -20.0
162.784
162.524
159.146
141.196
136.569
125.925
115.606
115.510
114.224
109.444
108.944
106.334
101.592
96.015
92.291
80.062
77.259
77.000
76.750
72.095
33.556
31.905
16.624
14.695
-0.020
X : parts per Million : Carbon13

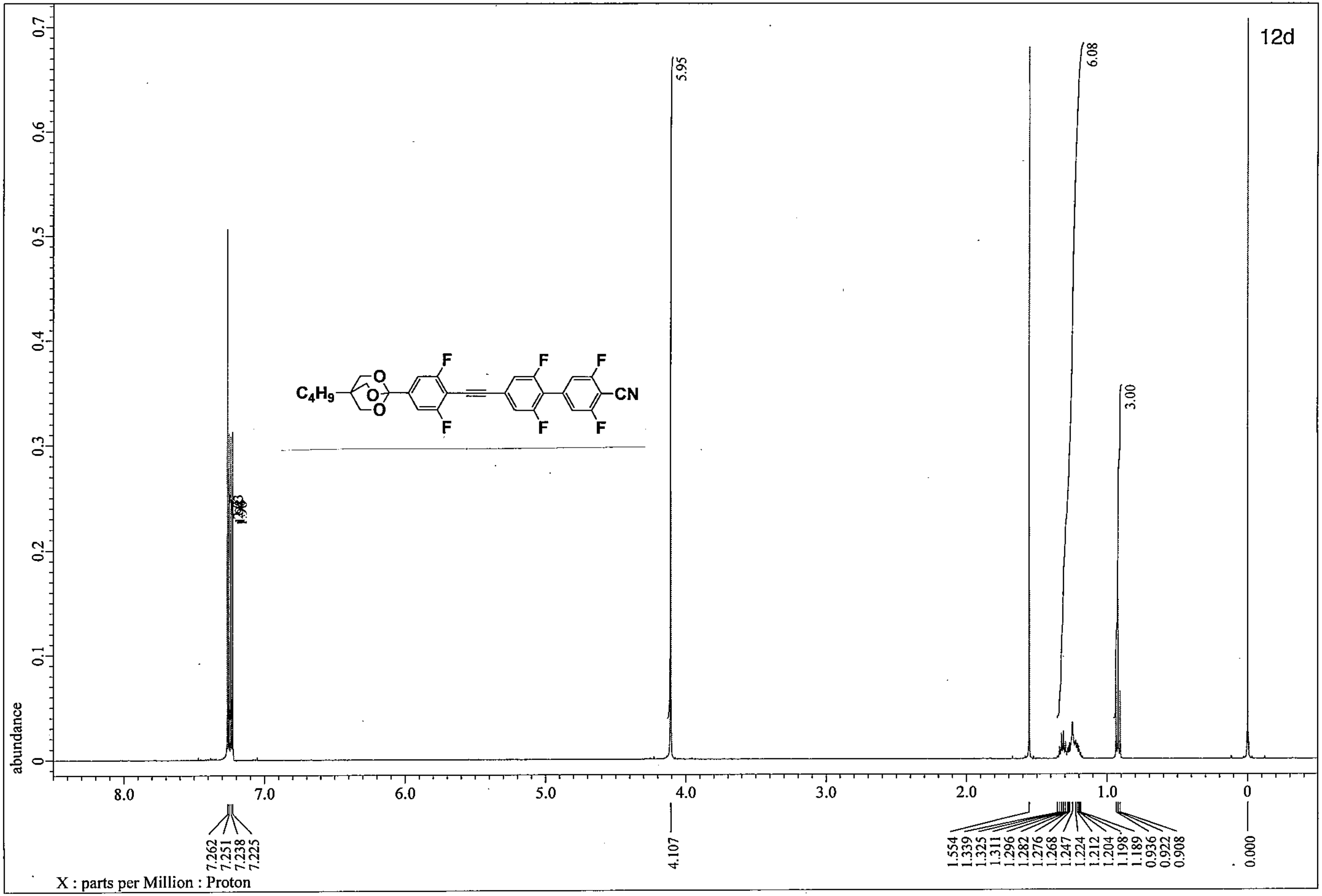
12d
C4H9
O
O
O
F
F
F
F
F
F
CN
5.95
6.08
3.00
abundance
0
0.1
0.2
0.3
0.4
0.5
0.6
0.7
8.0
7.0
6.0
5.0
4.0
3.0
2.0
1.0
0
7.262
7.251
7.238
7.225
4.107
1.554
1.339
1.325
1.311
1.296
1.282
1.276
1.268
1.247
1.224
1.212
1.204
1.198
1.189
0.936
0.922
0.908
0.000
X : parts per Million : Proton

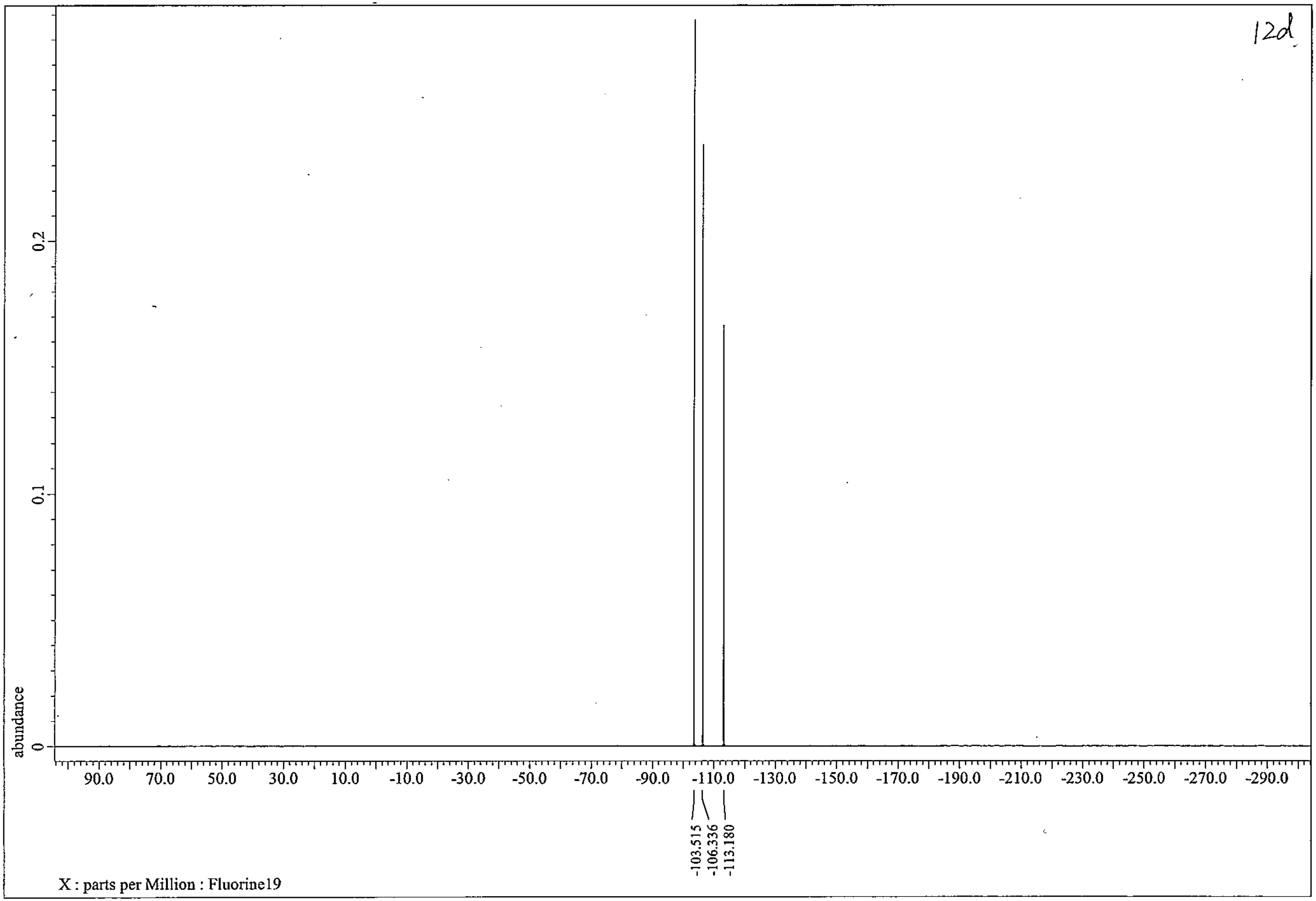

12d
abundance
0
0.1
0.2
90.0
70.0
50.0
30.0
10.0
-10.0
-30.0
-50.0
-70.0
-90.0
-110.0
-130.0
-150.0
-170.0
-190.0
-210.0
-230.0
-250.0
-270.0
-290.0
-103.515
-106.336
-113.180
X : parts per Million : Fluorine19

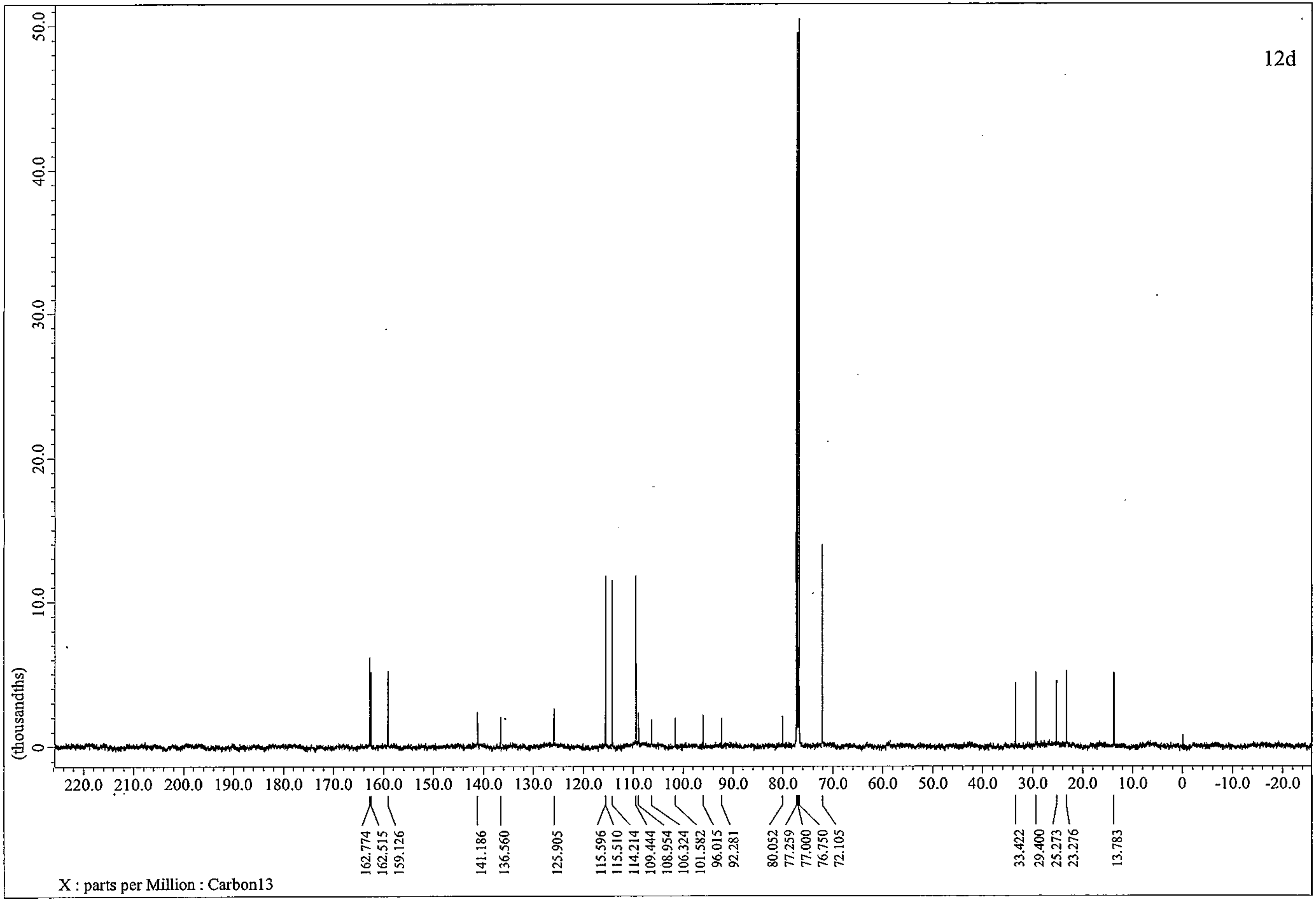

12d
(thousandths)
X : parts per Million : Carbon13
162.774
162.515
159.126
141.186
136.560
125.905
115.596
115.510
114.214
109.444
108.954
106.324
101.582
96.015
92.281
80.052
77.259
77.000
76.750
72.105
33.422
29.400
25.273
23.276
13.783

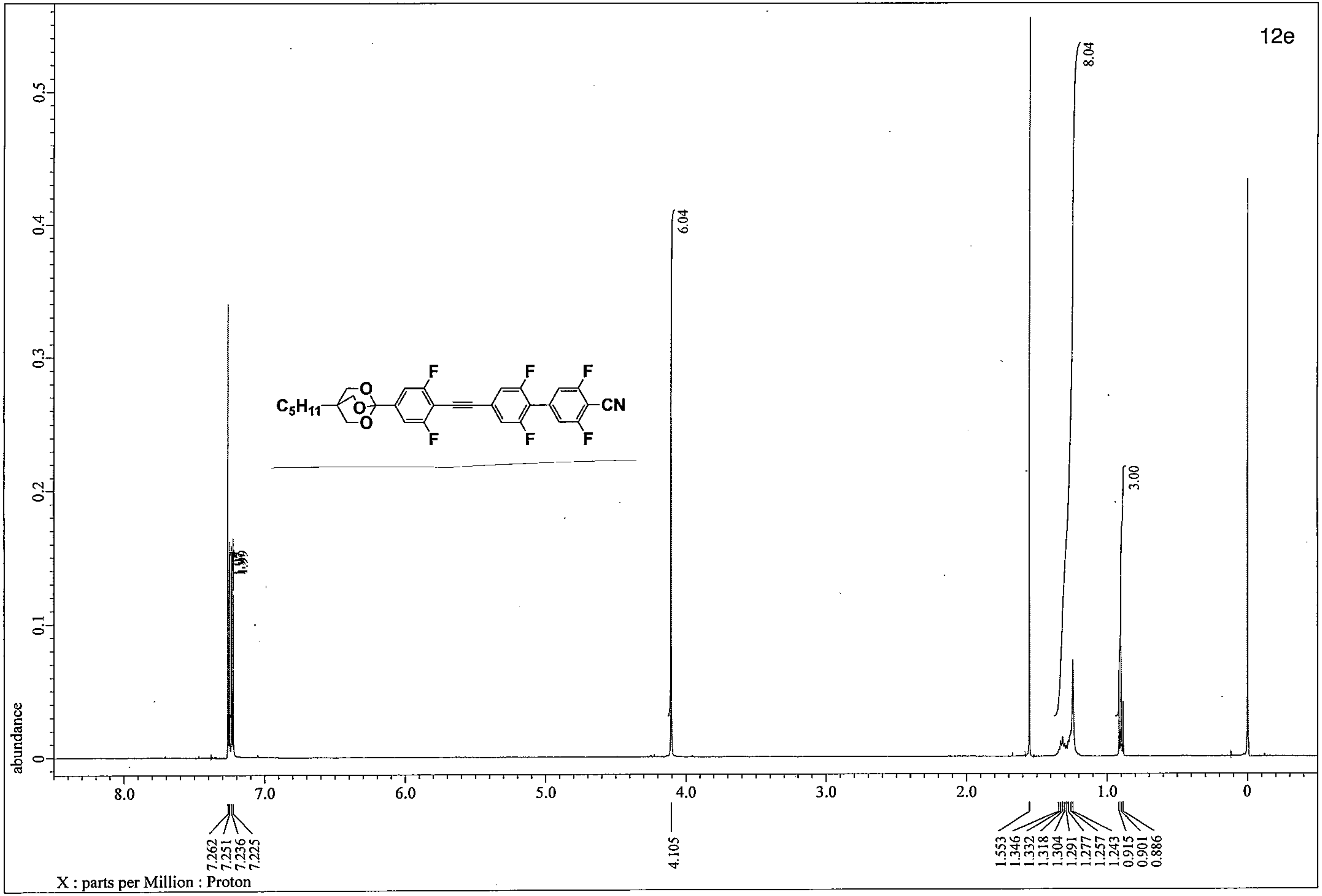

12e
C5H11
F
CN
6.04
8.04
3.00
abundance
0.5
0.4
0.3
0.2
0.1
0
8.0
7.0
6.0
5.0
4.0
3.0
2.0
1.0
0
7.262
7.251
7.236
7.225
4.105
1.553
1.346
1.332
1.318
1.304
1.291
1.277
1.257
1.243
0.915
0.901
0.886
X : parts per Million : Proton

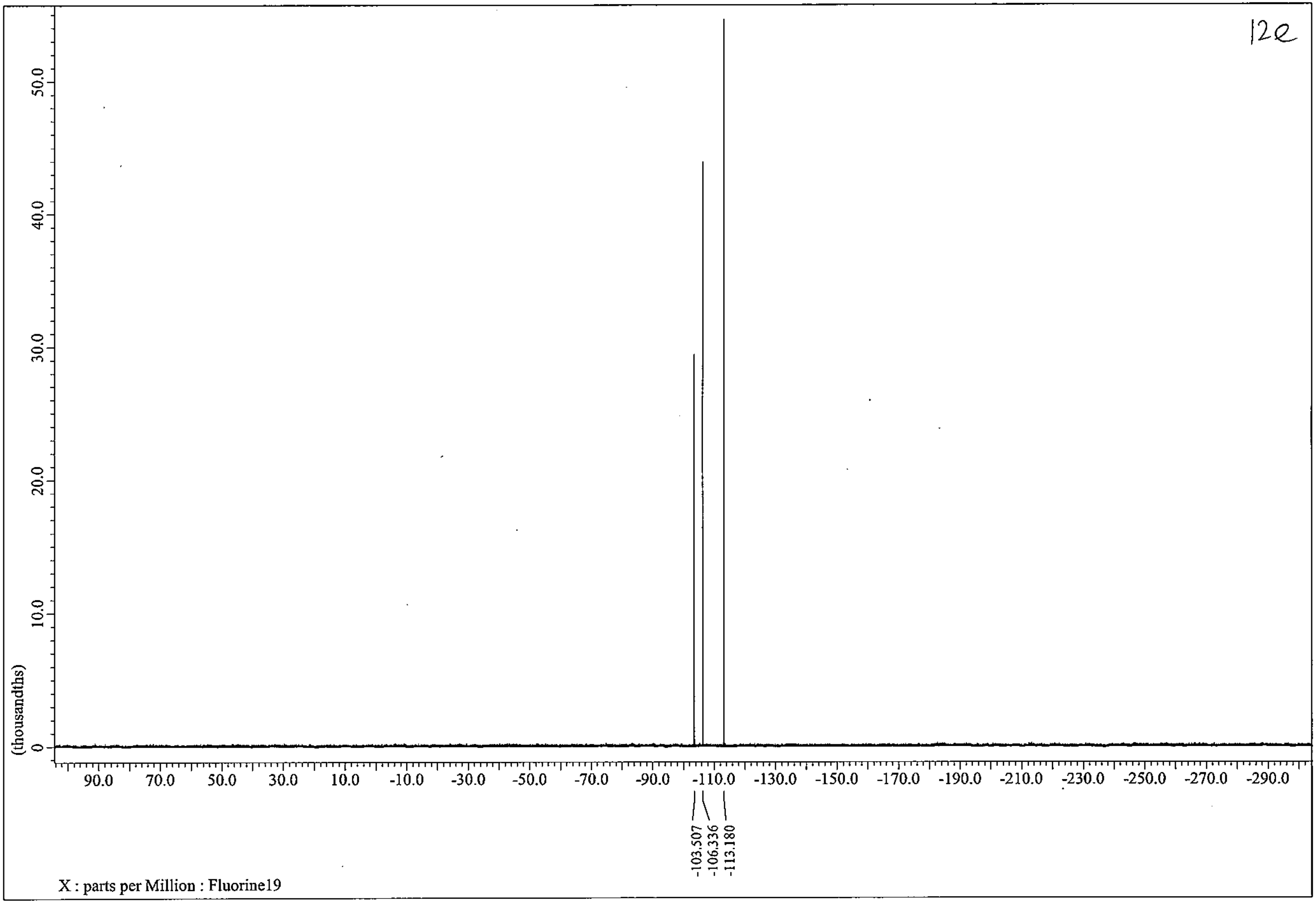

12e
(thousandths)
50.0
40.0
30.0
20.0
10.0
0
90.0
70.0
50.0
30.0
10.0
-10.0
-30.0
-50.0
-70.0
-90.0
-110.0
-130.0
-150.0
-170.0
-190.0
-210.0
-230.0
-250.0
-270.0
-290.0
-103.507
-106.336
-113.180
X : parts per Million : Fluorine19

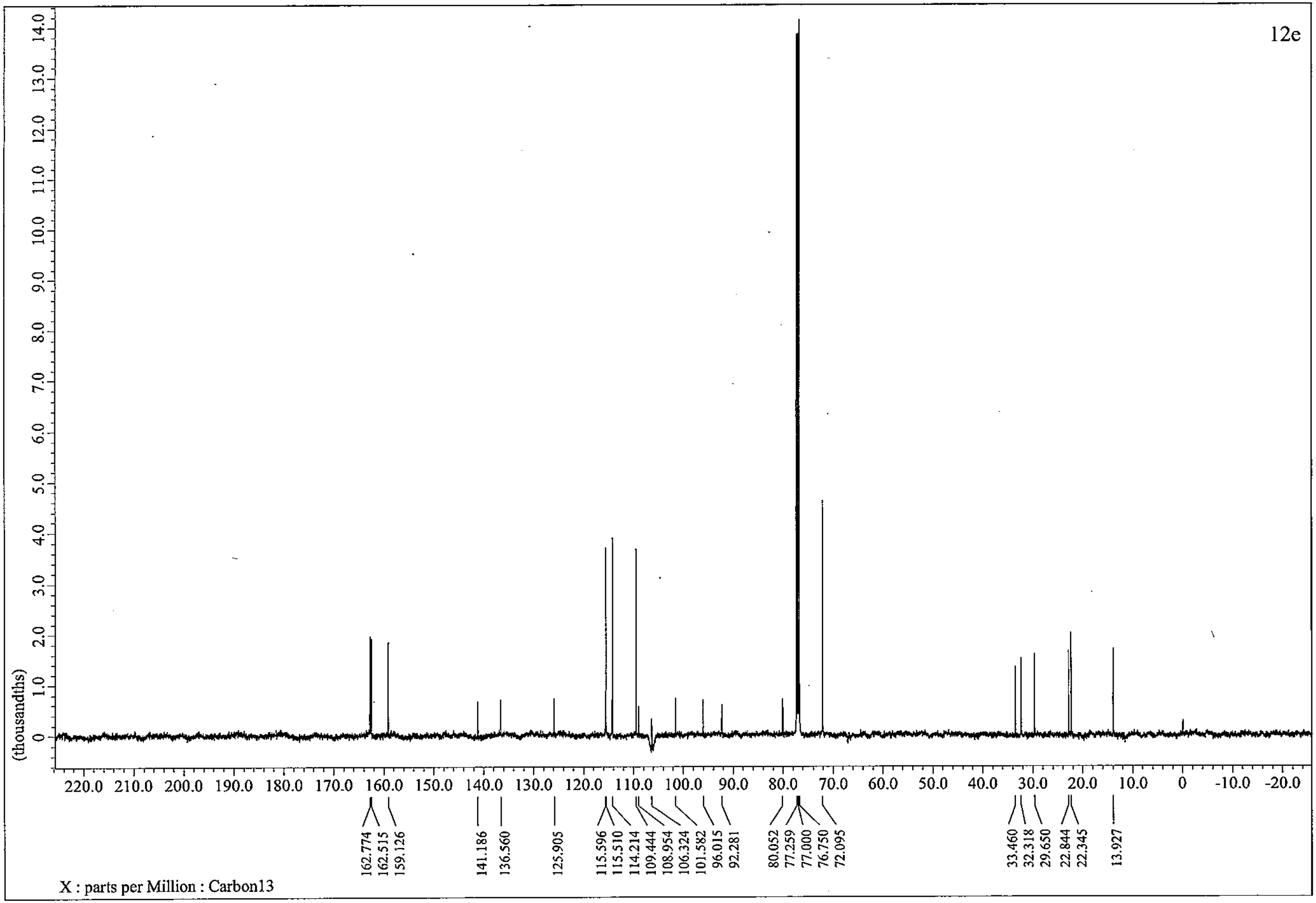
12e
(thousandths)
X : parts per Million : Carbon13
162.774
162.515
159.126
141.186
136.560
125.905
115.596
115.510
114.214
109.444
108.954
106.324
101.582
96.015
92.281
80.052
77.259
77.000
76.750
72.095
33.460
32.318
29.650
22.844
22.345
13.927

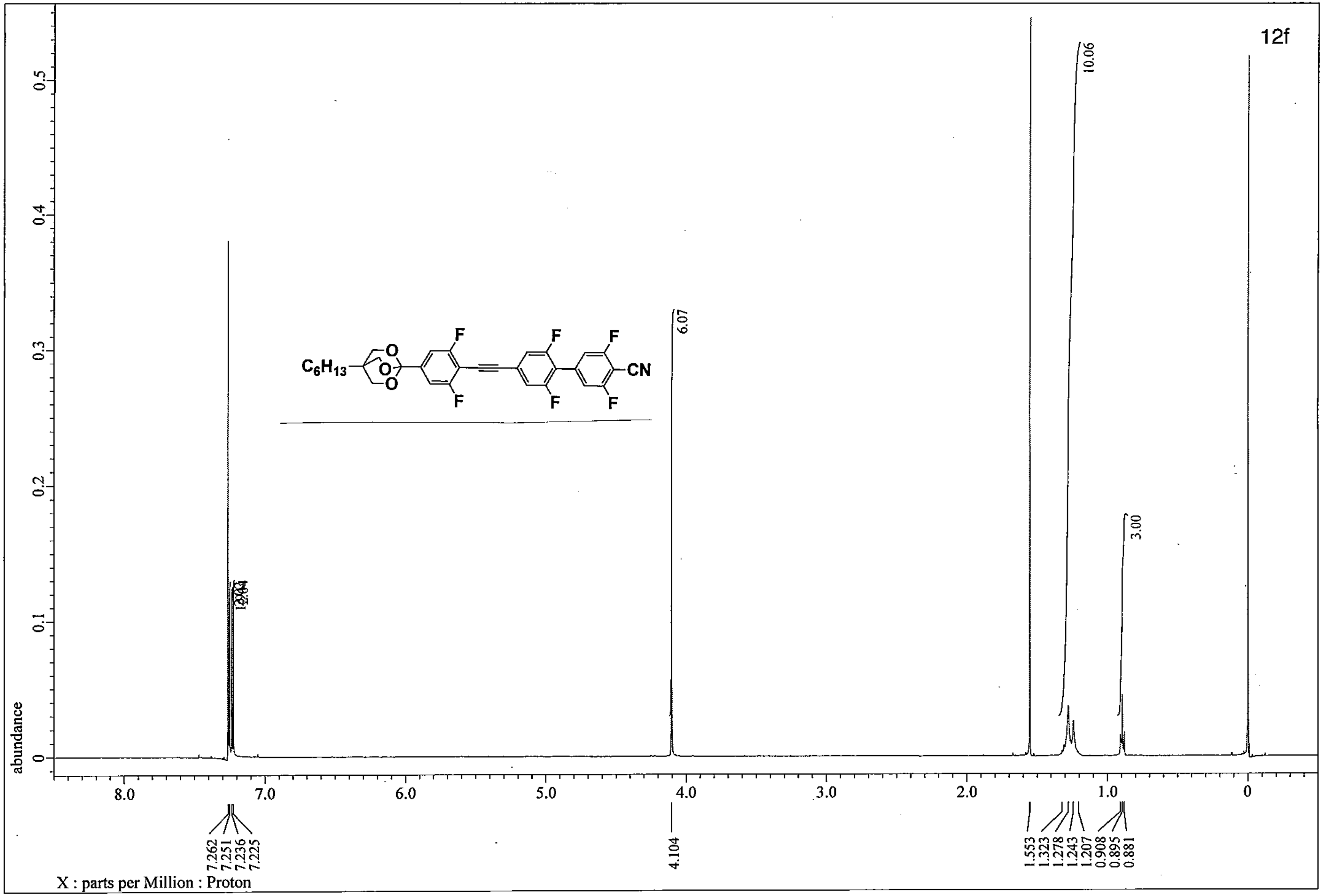
12f
C_6H_{13}
O
O
O
F
F
F
F
F
F
CN
6.07
10.06
3.00
abundance
0.5
0.4
0.3
0.2
0.1
0
8.0
7.0
6.0
5.0
4.0
3.0
2.0
1.0
0
7.262
7.251
7.236
7.225
4.104
1.553
1.323
1.278
1.243
1.207
0.908
0.895
0.881
X : parts per Million : Proton

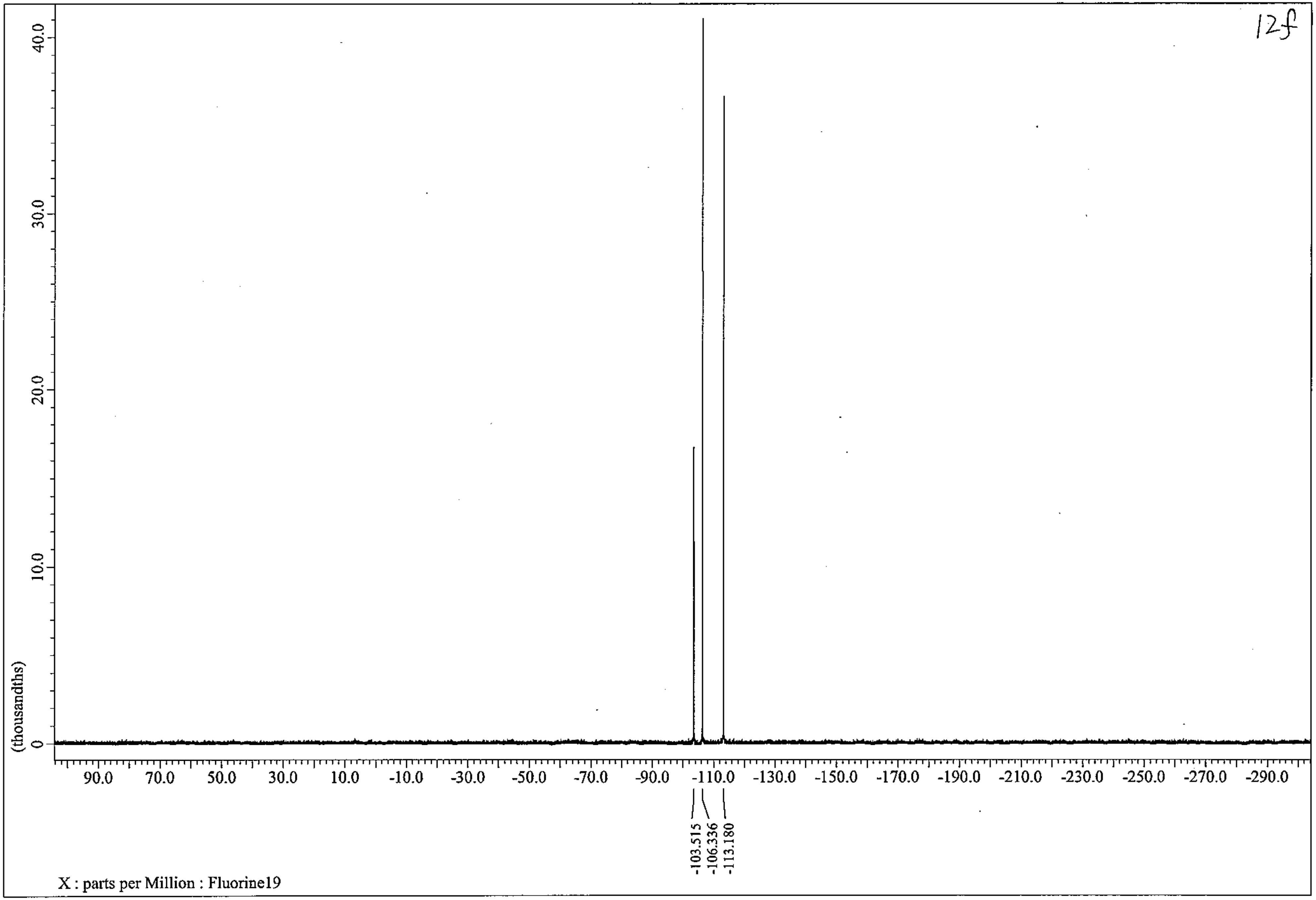
12f
40.0
30.0
20.0
10.0
0
(thousandths)
90.0 70.0 50.0 30.0 10.0 -10.0 -30.0 -50.0 -70.0 -90.0 -110.0 -130.0 -150.0 -170.0 -190.0 -210.0 -230.0 -250.0 -270.0 -290.0
-103.515
-106.336
-113.180
X : parts per Million : Fluorine19

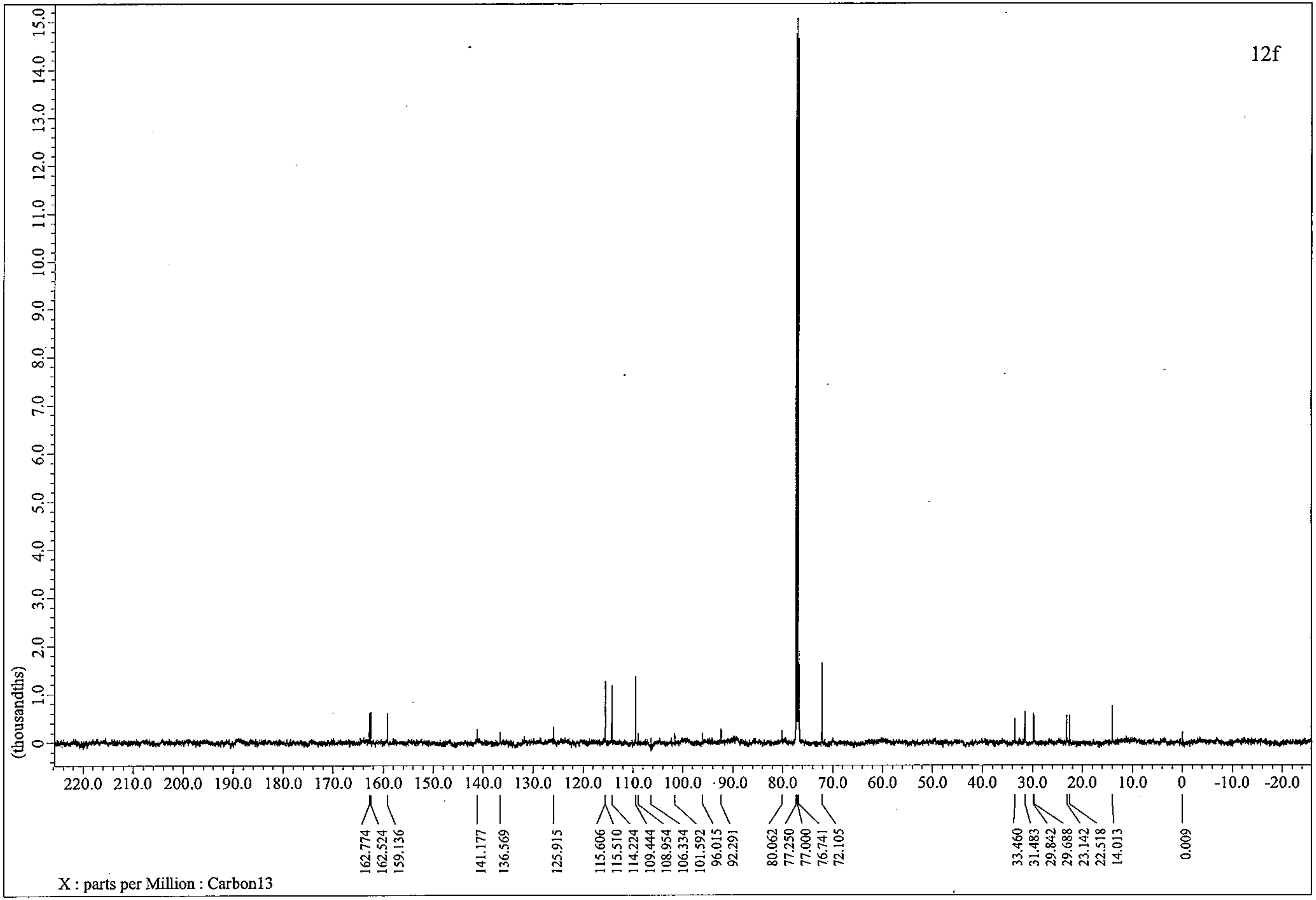
12f
(thousandths)
X : parts per Million : Carbon13
162.774
162.524
159.136
141.177
136.569
125.915
115.606
115.510
114.224
109.444
108.954
106.334
101.592
96.015
92.291
80.062
77.250
77.000
76.741
72.105
33.460
31.483
29.842
29.688
23.142
22.518
14.013
0.009

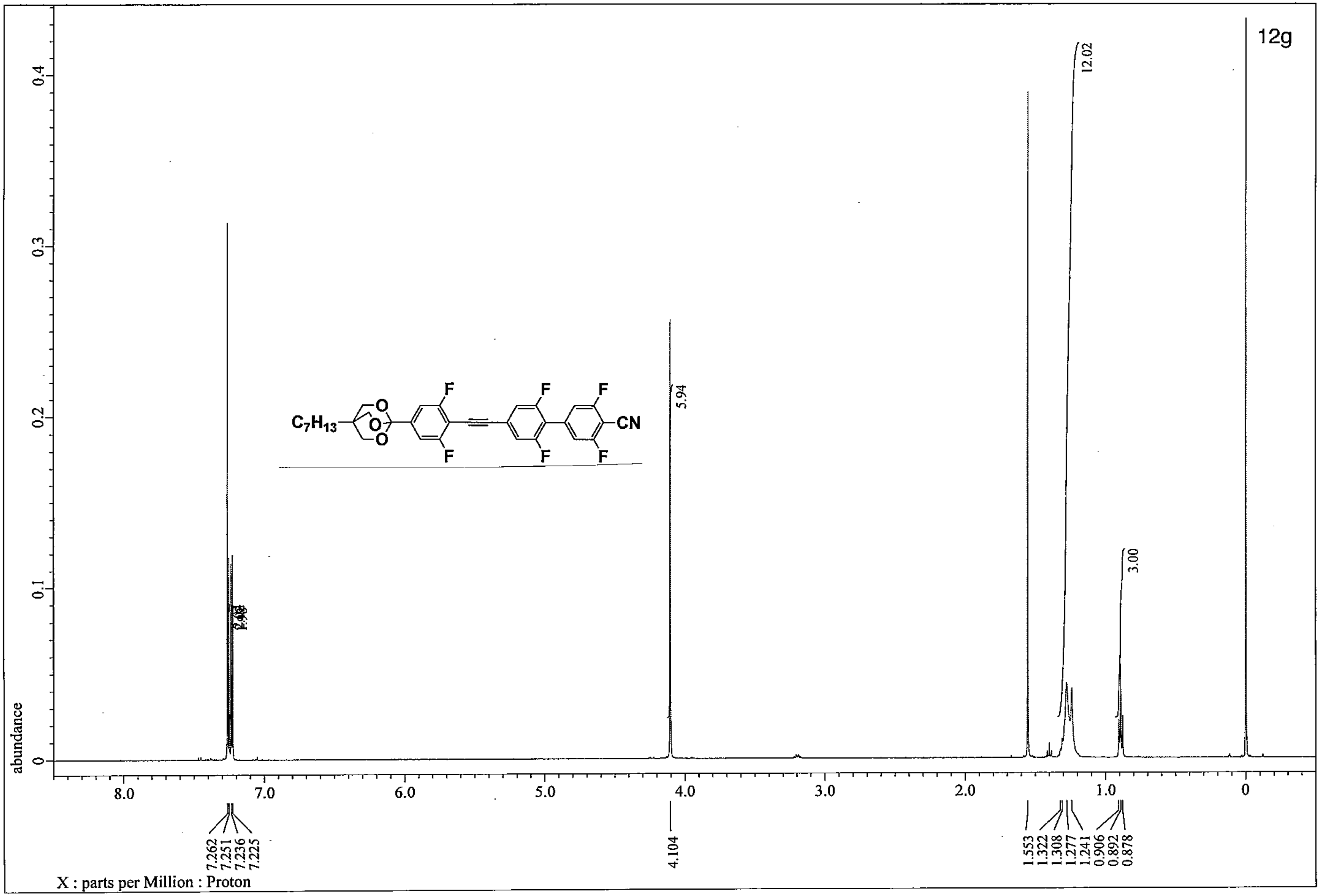

12g
C_7H_{13}
O
O
O
F
F
F
F
F
F
CN
abundance
0
0.1
0.2
0.3
0.4
5.94
12.02
3.00
8.0
7.0
6.0
5.0
4.0
3.0
2.0
1.0
0
7.262
7.251
7.236
7.225
4.104
1.553
1.322
1.308
1.277
1.241
0.906
0.892
0.878
X : parts per Million : Proton

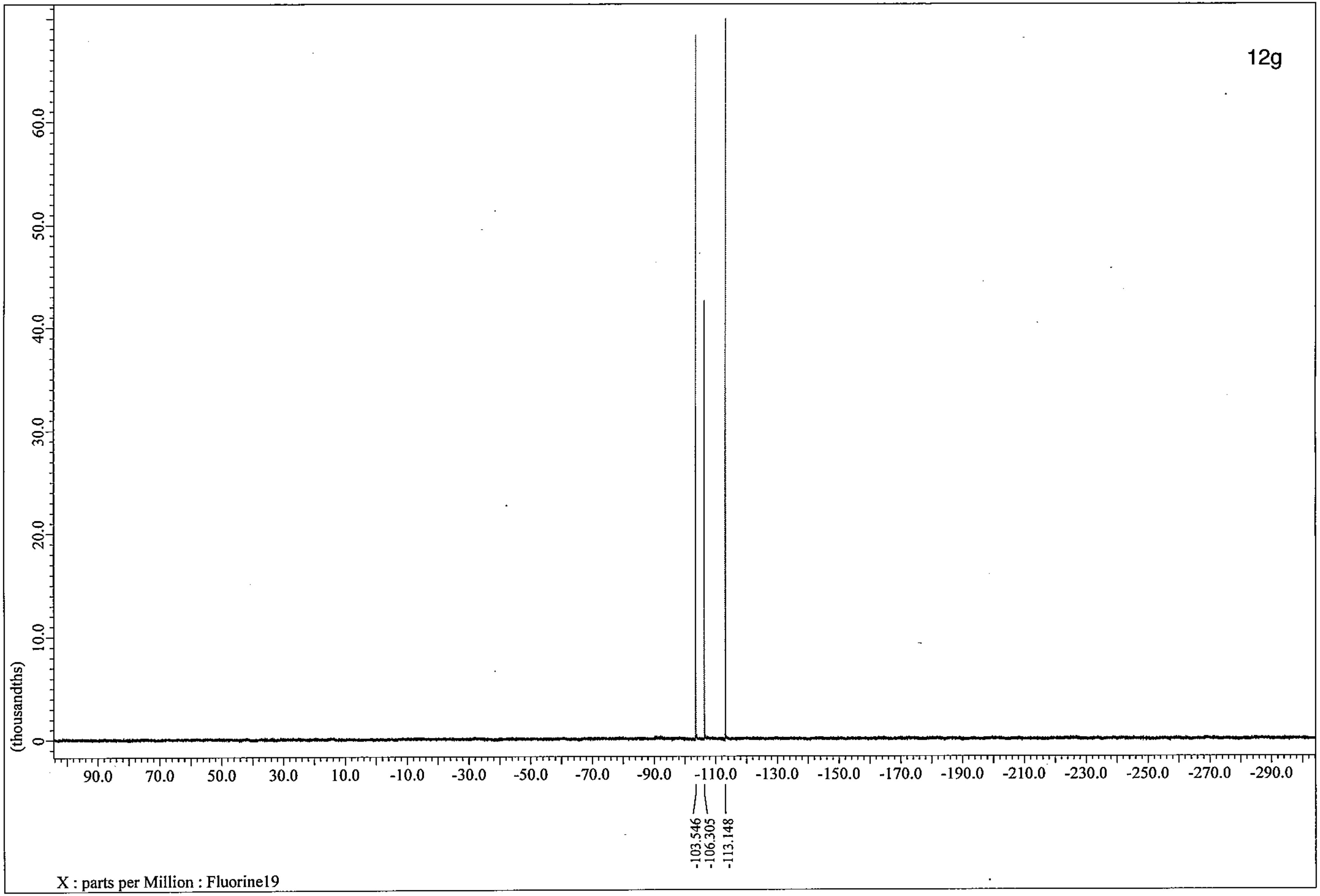
12g
(thousandths)
0
10.0
20.0
30.0
40.0
50.0
60.0
90.0
70.0
50.0
30.0
10.0
-10.0
-30.0
-50.0
-70.0
-90.0
-110.0
-130.0
-150.0
-170.0
-190.0
-210.0
-230.0
-250.0
-270.0
-290.0
-103.546
-106.305
-113.148
X : parts per Million : Fluorine19

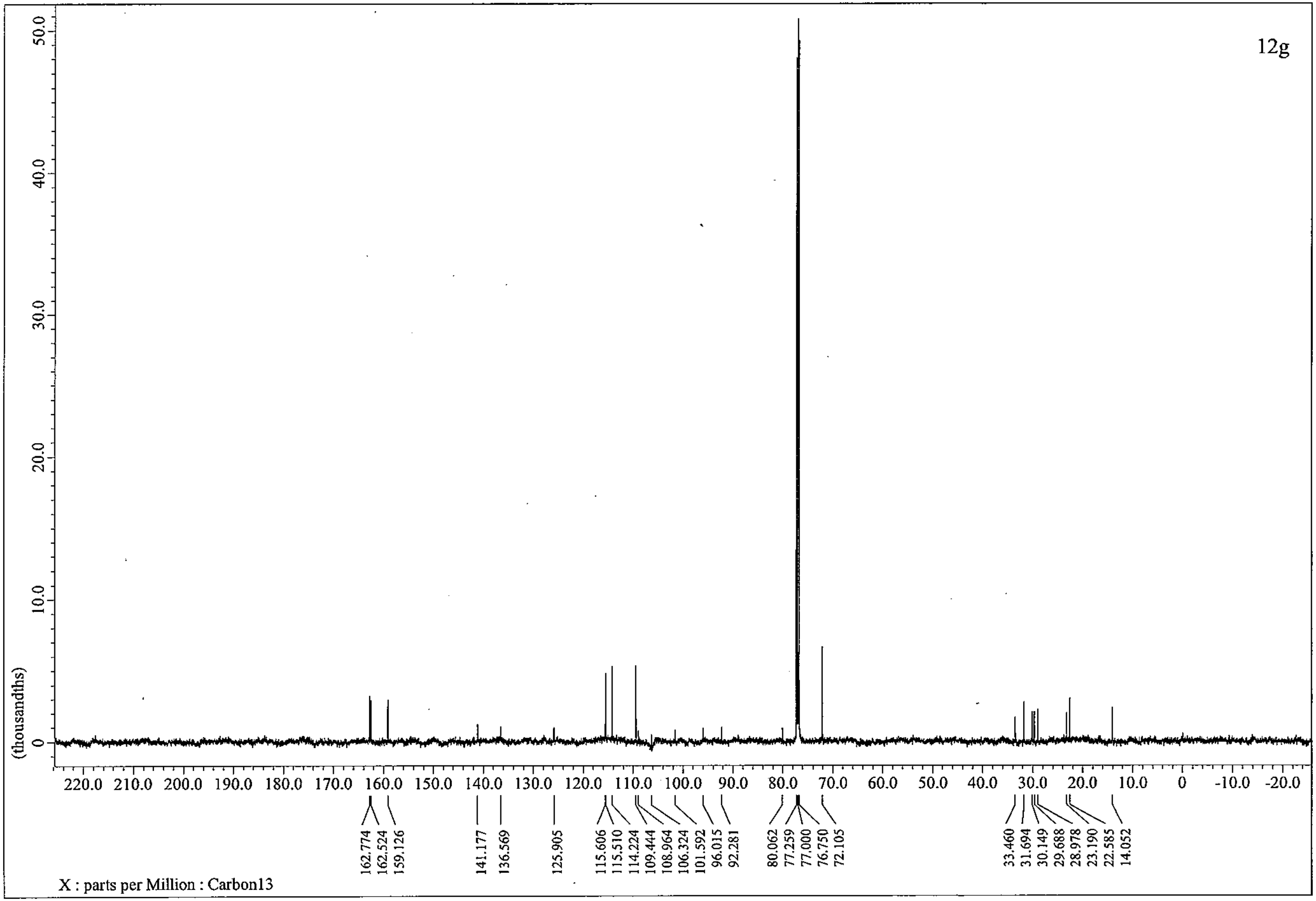
12g
50.0
40.0
30.0
20.0
10.0
0
(thousandths)
220.0 210.0 200.0 190.0 180.0 170.0 160.0 150.0 140.0 130.0 120.0 110.0 100.0 90.0 80.0 70.0 60.0 50.0 40.0 30.0 20.0 10.0 0 -10.0 -20.0
162.774
162.524
159.126
141.177
136.569
125.905
115.606
115.510
114.224
109.444
108.964
106.324
101.592
96.015
92.281
80.062
77.259
77.000
76.750
72.105
33.460
31.694
30.149
29.688
28.978
23.190
22.585
14.052
X : parts per Million : Carbon13

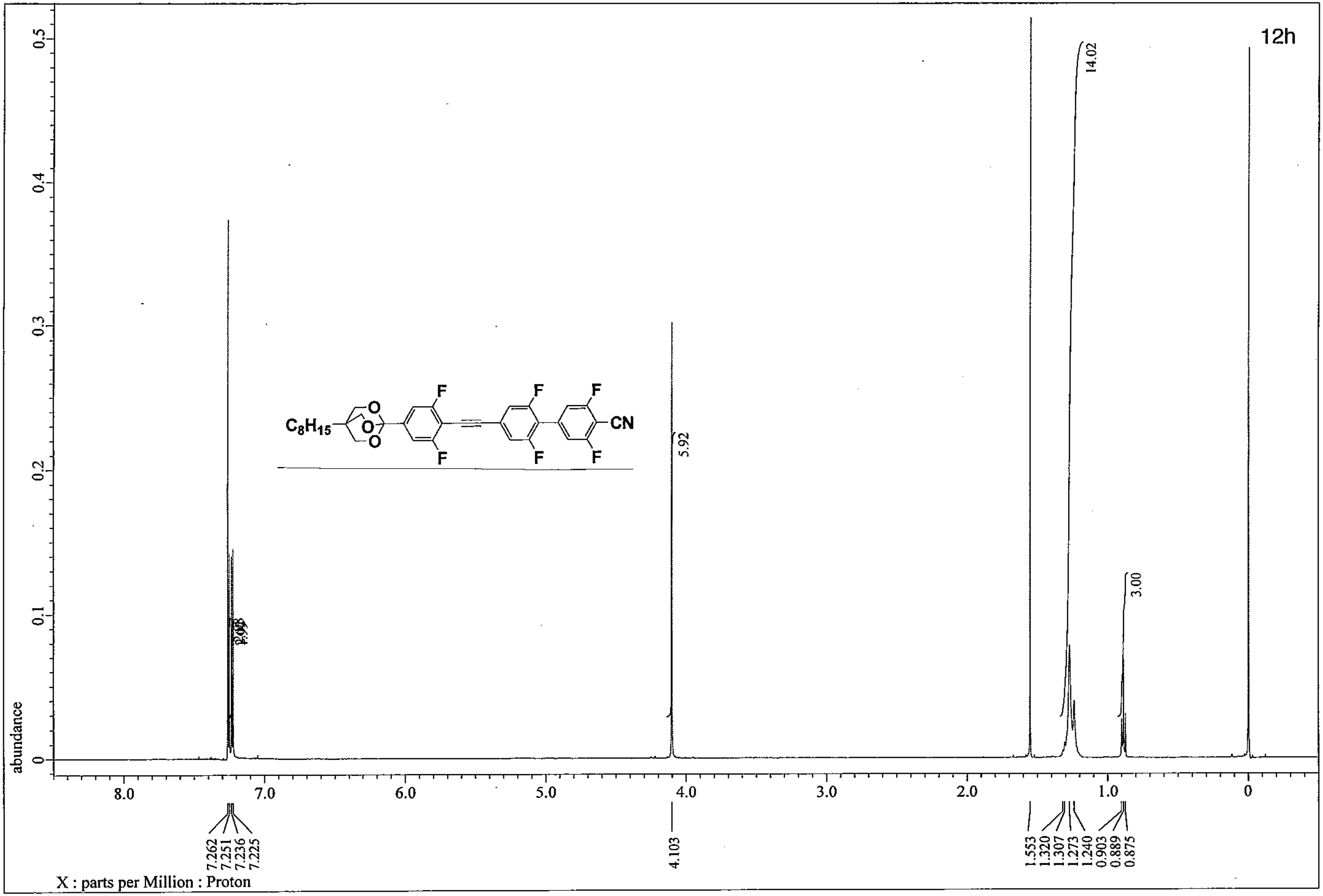
12h
C_8H_{15}
O
O
O
F
F
F
F
F
F
CN
14.02
5.92
3.00
abundance
0
0.1
0.2
0.3
0.4
0.5
8.0
7.0
6.0
5.0
4.0
3.0
2.0
1.0
0
7.262
7.251
7.236
7.225
4.103
1.553
1.320
1.307
1.273
1.240
0.903
0.889
0.875
X : parts per Million : Proton

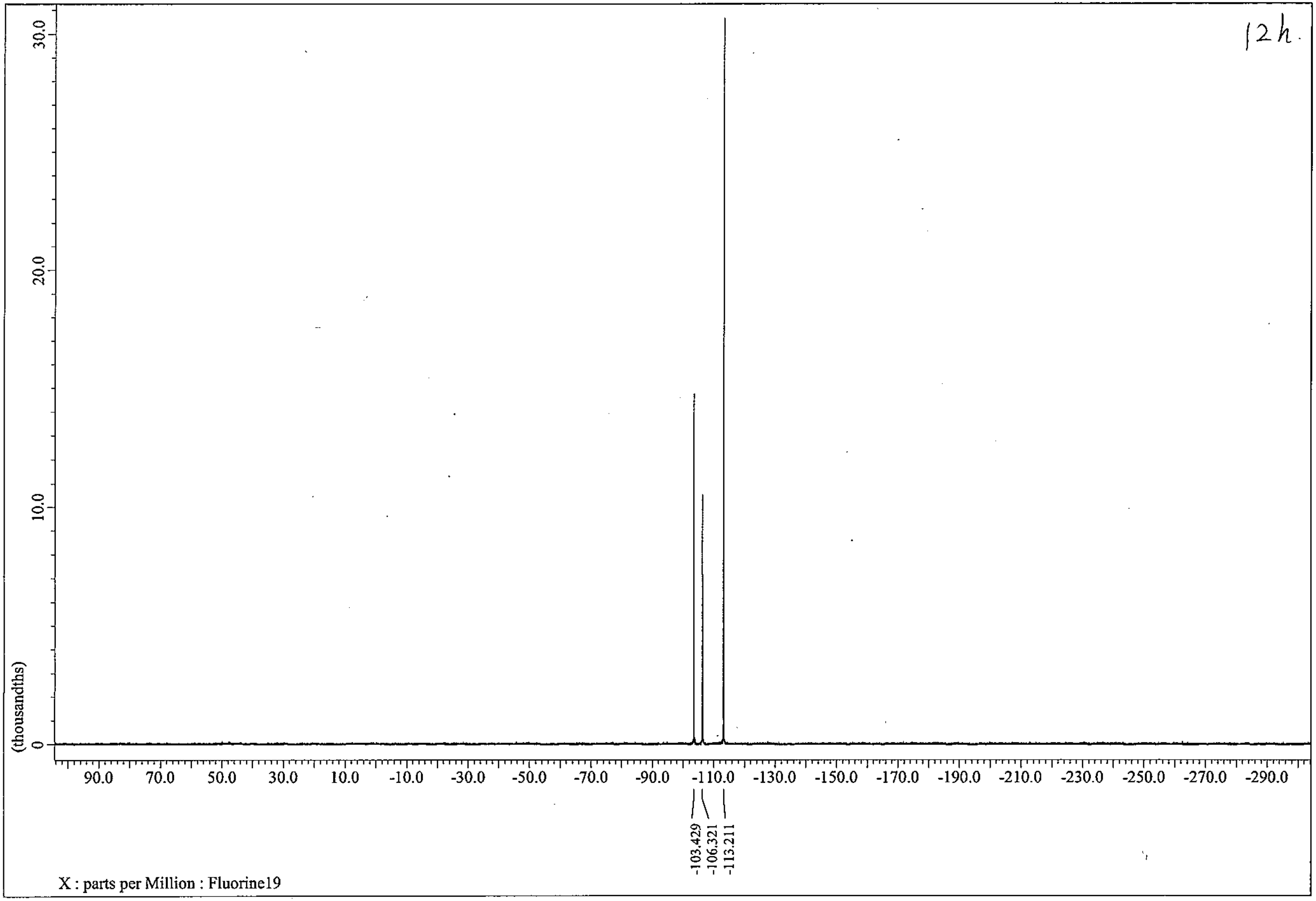
12h
30.0
20.0
10.0
0
(thousandths)
90.0
70.0
50.0
30.0
10.0
-10.0
-30.0
-50.0
-70.0
-90.0
-110.0
-130.0
-150.0
-170.0
-190.0
-210.0
-230.0
-250.0
-270.0
-290.0
-103.429
-106.321
-113.211
X : parts per Million : Fluorine19

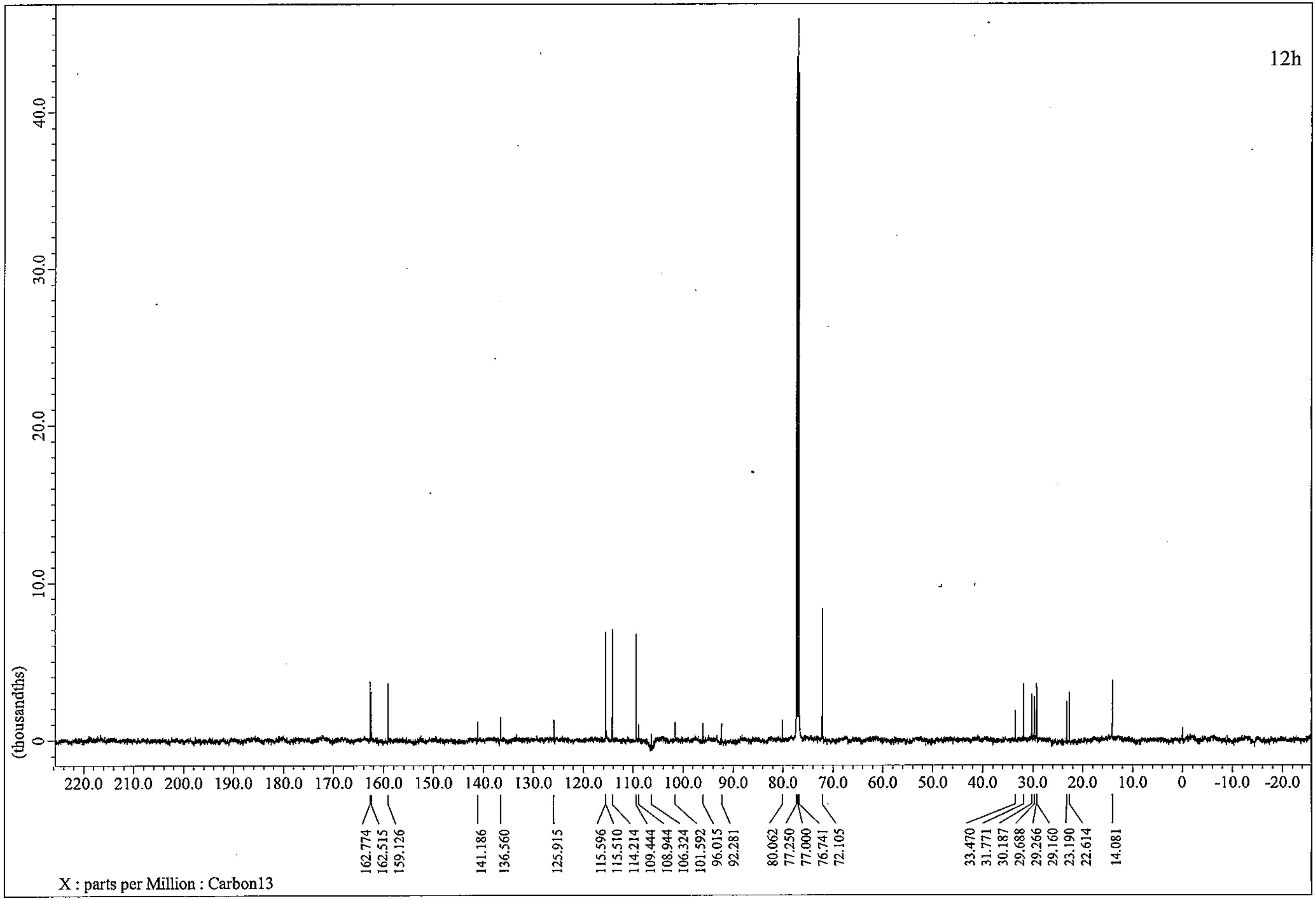
12h
40.0
30.0
20.0
10.0
0
(thousandths)
220.0 210.0 200.0 190.0 180.0 170.0 160.0 150.0 140.0 130.0 120.0 110.0 100.0 90.0 80.0 70.0 60.0 50.0 40.0 30.0 20.0 10.0 0 -10.0 -20.0
162.774
162.515
159.126
141.186
136.560
125.915
115.596
115.510
114.214
109.444
108.944
106.324
101.592
96.015
92.281
80.062
77.250
77.000
76.741
72.105
33.470
31.771
30.187
29.688
29.266
29.160
23.190
22.614
14.081
X : parts per Million : Carbon13

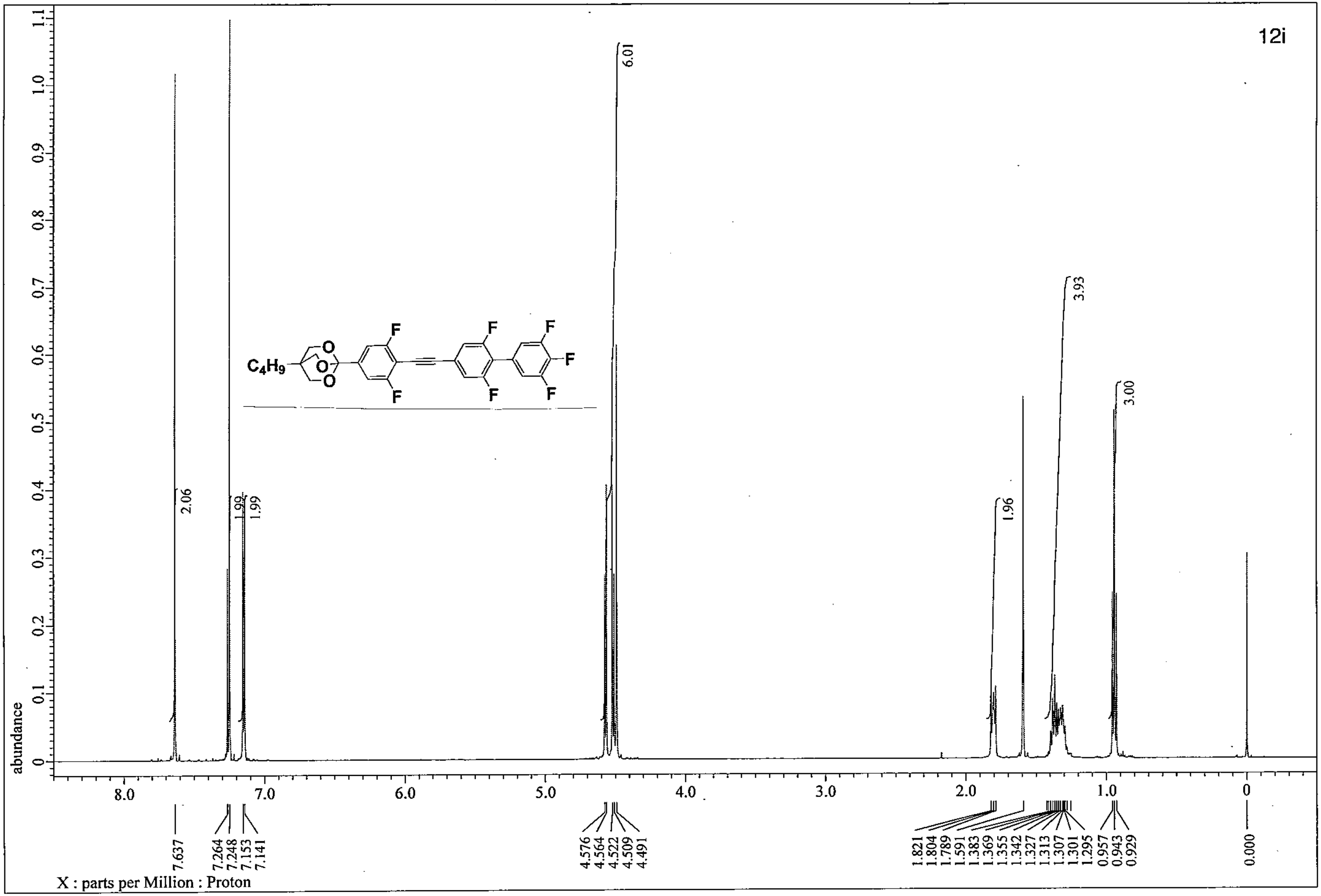
12i
C_4H_9
O
O
O
F
F
F
F
F
F
F
abundance
1.1
1.0
0.9
0.8
0.7
0.6
0.5
0.4
0.3
0.2
0.1
0
2.06
1.99
1.99
6.01
1.96
3.93
3.00
8.0
7.0
6.0
5.0
4.0
3.0
2.0
1.0
0
7.637
7.264
7.248
7.153
7.141
4.576
4.564
4.522
4.509
4.491
1.821
1.804
1.789
1.591
1.383
1.369
1.355
1.342
1.327
1.313
1.307
1.301
1.295
0.957
0.943
0.929
0.000
X : parts per Million : Proton

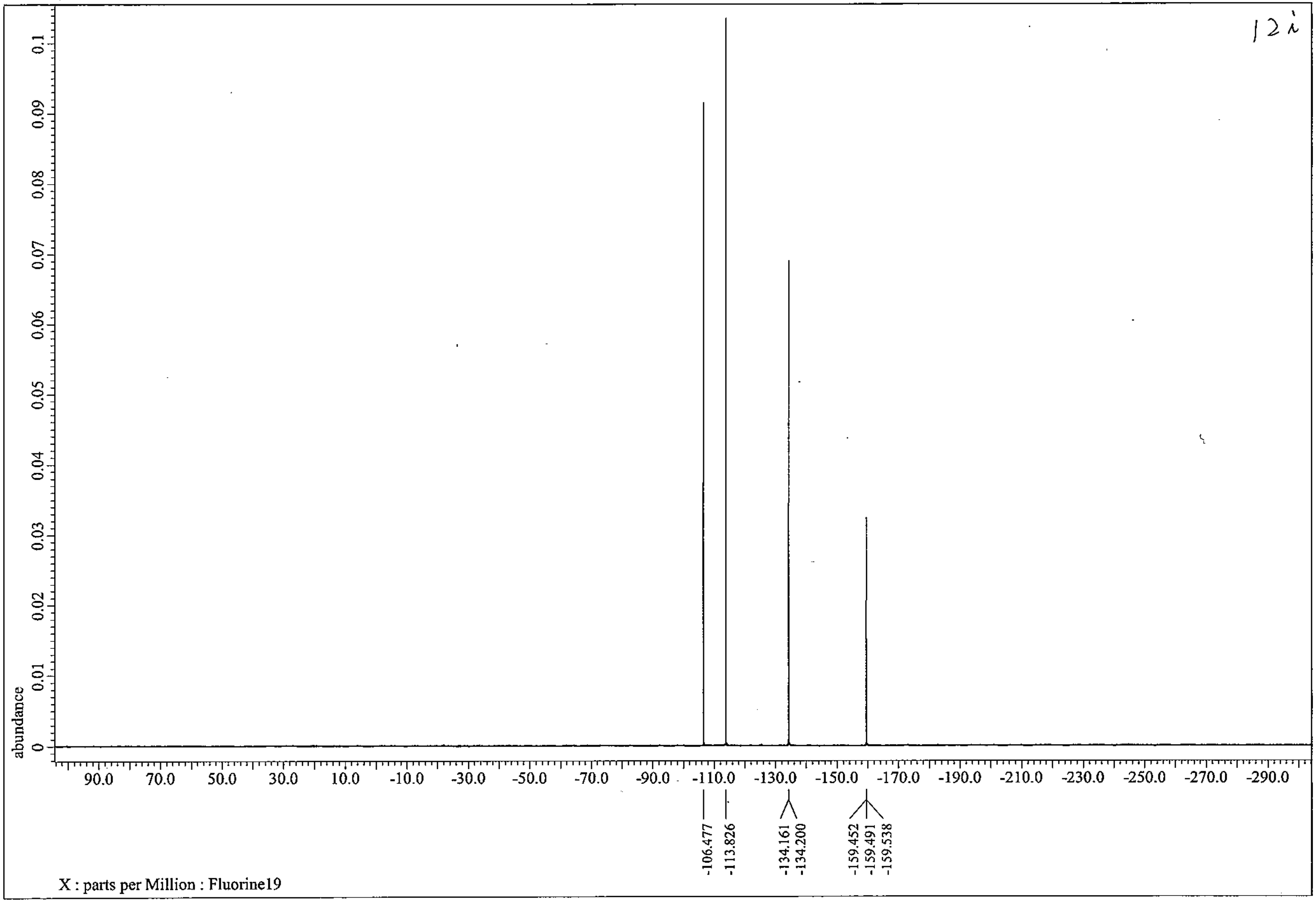
12i
abundance
0.1
0.09
0.08
0.07
0.06
0.05
0.04
0.03
0.02
0.01
0
90.0 70.0 50.0 30.0 10.0 -10.0 -30.0 -50.0 -70.0 -90.0 -110.0 -130.0 -150.0 -170.0 -190.0 -210.0 -230.0 -250.0 -270.0 -290.0
-106.477
-113.826
-134.161
-134.200
-159.452
-159.491
-159.538
X : parts per Million : Fluorine19

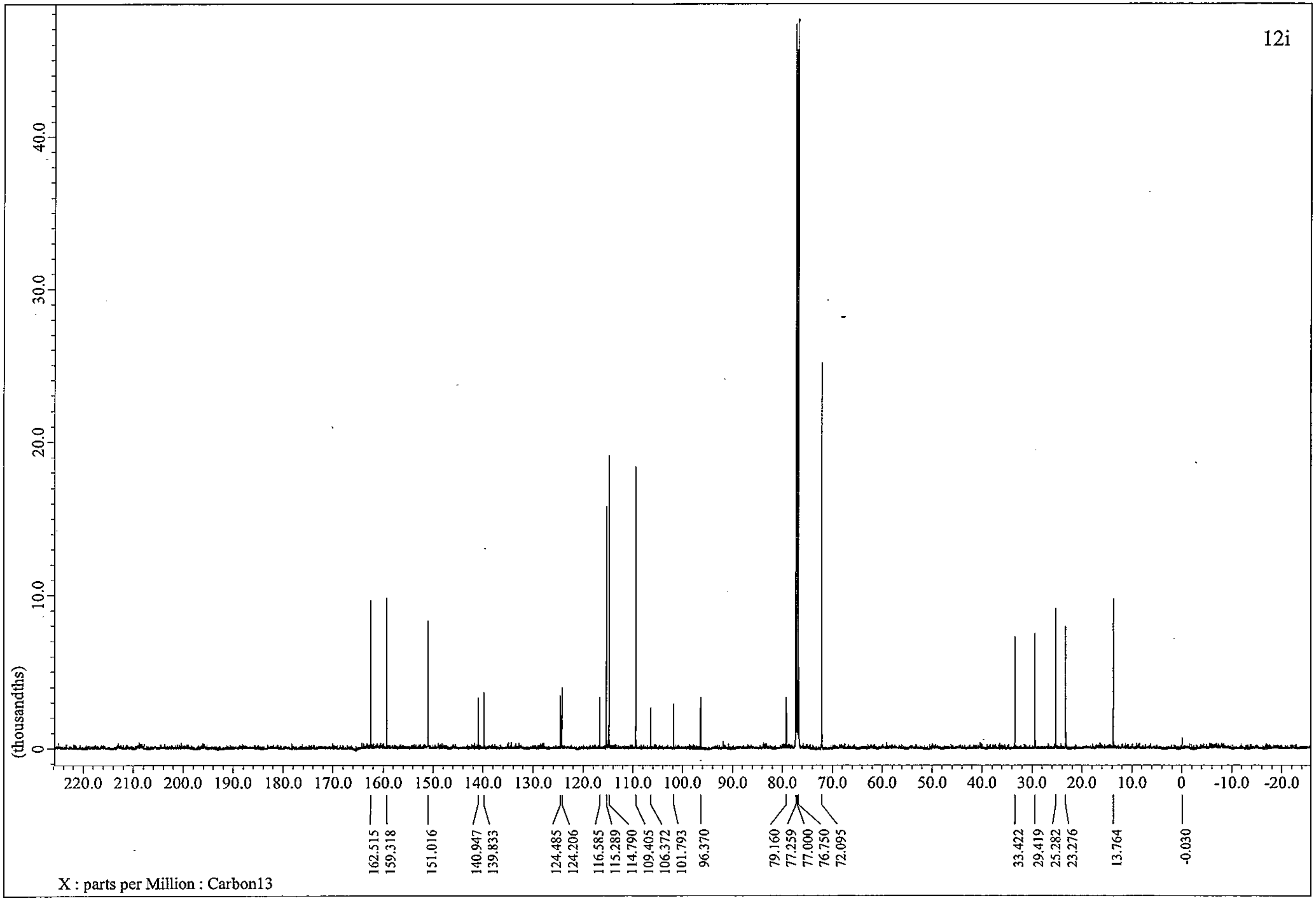

12i
(thousandths)
40.0
30.0
20.0
10.0
0
220.0 210.0 200.0 190.0 180.0 170.0 160.0 150.0 140.0 130.0 120.0 110.0 100.0 90.0 80.0 70.0 60.0 50.0 40.0 30.0 20.0 10.0 0 -10.0 -20.0
162.515
159.318
151.016
140.947
139.833
124.485
124.206
116.585
115.289
114.790
109.405
106.372
101.793
96.370
79.160
77.259
77.000
76.750
72.095
33.422
29.419
25.282
23.276
13.764
-0.030
X : parts per Million : Carbon13

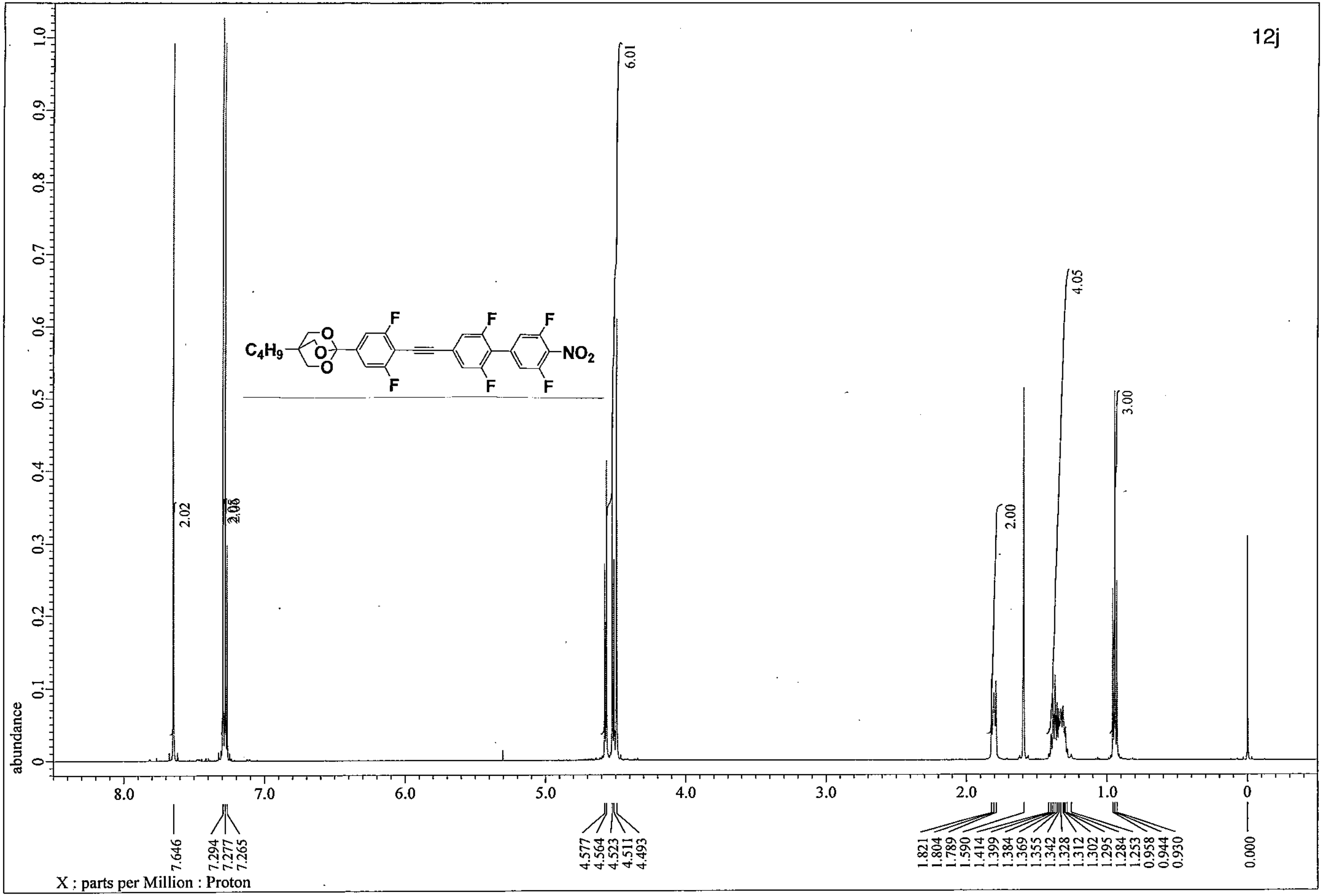

12j
C4H9
NO2
F
abundance
X : parts per Million : Proton
8.0
7.0
6.0
5.0
4.0
3.0
2.0
1.0
0
2.02
6.01
4.05
2.00
3.00
7.646
7.294
7.277
7.265
4.577
4.564
4.523
4.511
4.493
1.821
1.804
1.789
1.590
1.414
1.399
1.384
1.369
1.355
1.342
1.328
1.312
1.302
1.295
1.284
1.253
0.958
0.944
0.930
0.000

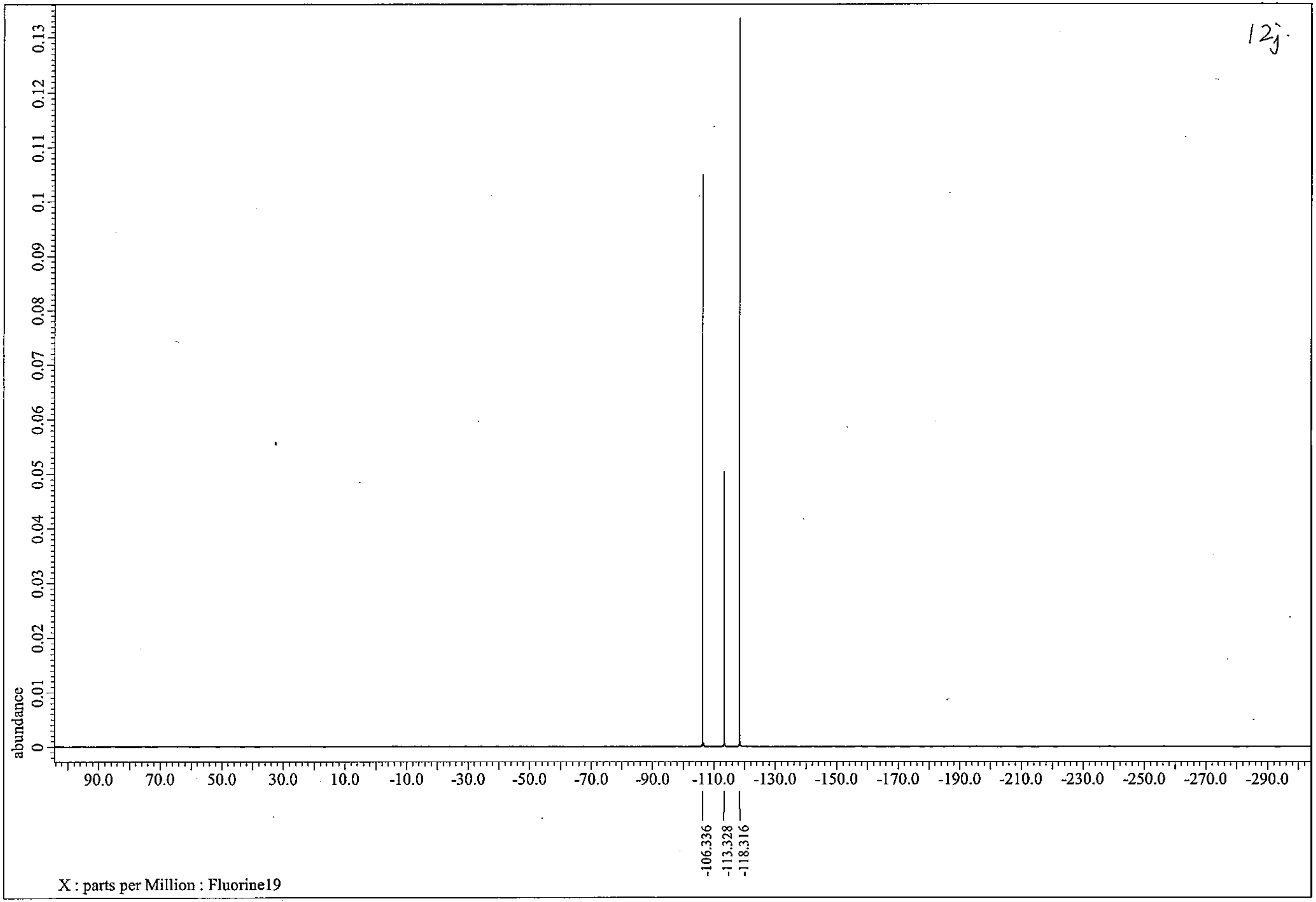
12j
abundance
0
0.01
0.02
0.03
0.04
0.05
0.06
0.07
0.08
0.09
0.1
0.11
0.12
0.13
90.0
70.0
50.0
30.0
10.0
-10.0
-30.0
-50.0
-70.0
-90.0
-110.0
-130.0
-150.0
-170.0
-190.0
-210.0
-230.0
-250.0
-270.0
-290.0
-106.336
-113.328
-118.316
X : parts per Million : Fluorine19

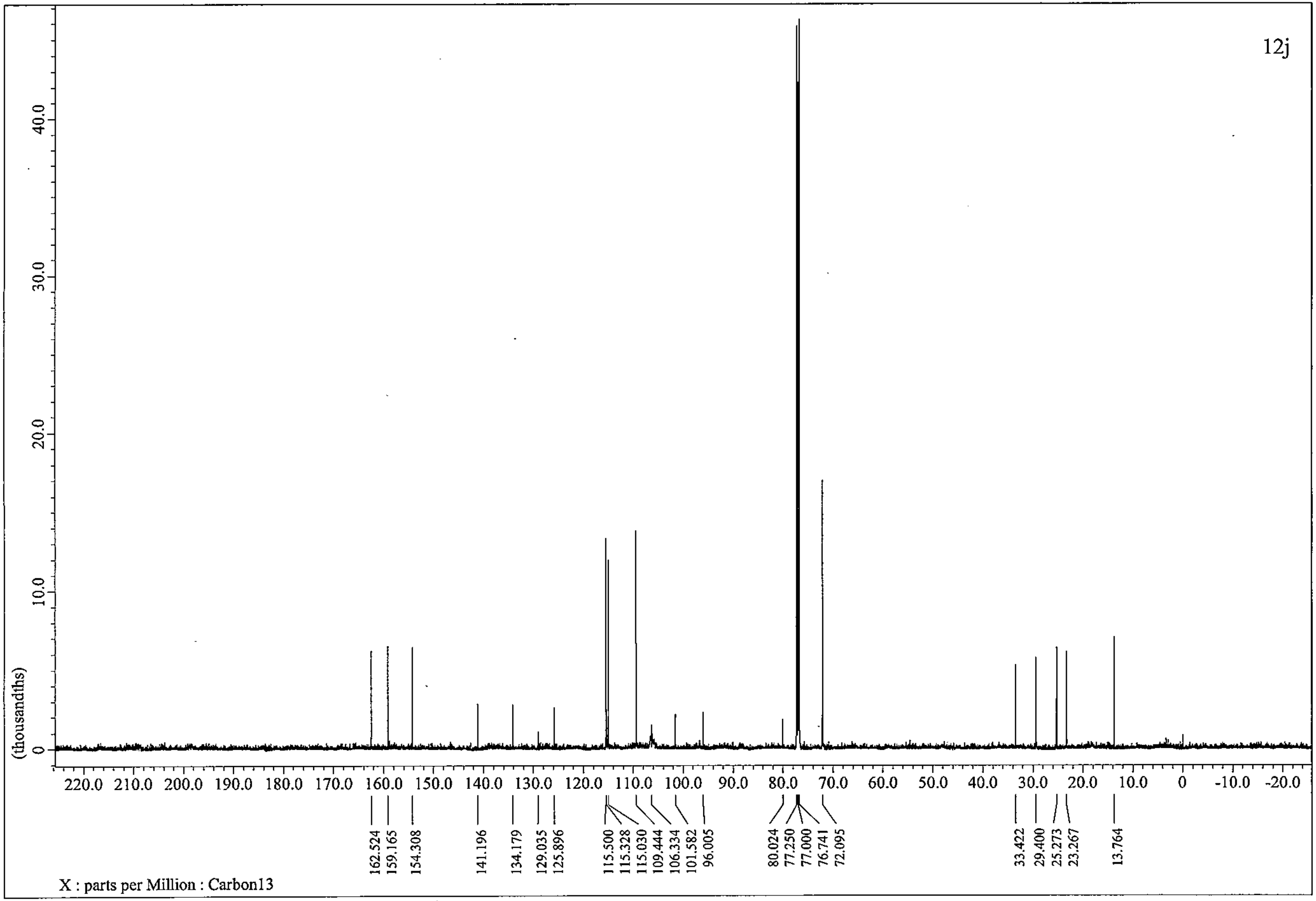
12j
(thousandths)
40.0
30.0
20.0
10.0
0
220.0 210.0 200.0 190.0 180.0 170.0 160.0 150.0 140.0 130.0 120.0 110.0 100.0 90.0 80.0 70.0 60.0 50.0 40.0 30.0 20.0 10.0 0 -10.0 -20.0
162.524
159.165
154.308
141.196
134.179
129.035
125.896
115.500
115.328
115.030
109.444
106.334
101.582
96.005
80.024
77.250
77.000
76.741
72.095
33.422
29.400
25.273
23.267
13.764
X : parts per Million : Carbon13
